# Training Population Design With the Use of Regional Fusarium Head Blight Nurseries to Predict Independent Breeding Lines for FHB Traits

**DOI:** 10.3389/fpls.2020.01083

**Published:** 2020-07-16

**Authors:** Virginia L. Verges, Jeanette Lyerly, Yanhong Dong, David A. Van Sanford

**Affiliations:** ^1^Department of Plant and Soil Sciences, University of Kentucky, Lexington, KY, United States; ^2^Department of Crop and Soil Sciences, North Carolina State University, Raleigh, NC, United States; ^3^Department of Plant Pathology, University of Minnesota, St. Paul, MN, United States

**Keywords:** genomic selection, *Fusarium* head blight, training population, DON content, disease resistance, prediction accuracy

## Abstract

Fusarium head blight (FHB) is a devastating disease in cereals around the world. Because it is quantitatively inherited and technically difficult to reproduce, breeding to increase resistance in wheat germplasm is difficult and slow. Genomic selection (GS) is a form of marker-assisted selection (MAS) that simultaneously estimates all locus, haplotype, or marker effects across the entire genome to calculate genomic estimated breeding values (GEBVs). Since its inception, there have been many studies that demonstrate the utility of GS approaches to breeding for disease resistance in crops. In this study, the Uniform Northern (NUS) and Uniform Southern (SUS) soft red winter wheat scab nurseries (a total 452 lines) were evaluated as possible training populations (TP) to predict FHB traits in breeding lines of the UK (University of Kentucky) wheat breeding program. DON was best predicted by the SUS; Fusarium damaged kernels (FDK), FHB rating, and two indices, DSK index and DK index were best predicted by NUS. The highest prediction accuracies were obtained when the NUS and SUS were combined, reaching up to 0.5 for almost all traits except FHB rating. Highest prediction accuracies were obtained with bigger TP sizes (300–400) and there were not significant effects of TP optimization method for all traits, although at small TP size, the PEVmean algorithm worked better than other methods. To select for lines with tolerance to DON accumulation, a primary breeding target for many breeders, we compared selection based on DON BLUES with selection based on DON GEBVs, DSK GEBVs, and DK GEBVs. At selection intensities (SI) of 30–40%, DSK index showed the best performance with a 4–6% increase over direct selection for DON. Our results confirm the usefulness of regional nurseries as a source of lines to predict GEBVs for local breeding programs, and shows that an index that includes DON, together with FDK and FHB rating could be an excellent choice to identify lines with low DON content and an overall improved FHB resistance.

## Introduction

Fusarium head blight (FHB) is one of the most devastating diseases of bread wheat (*Triticum aestivum* L.) worldwide, which leads to significant losses in grain yield and quality. FHB is particularly aggressive in regions with cropping systems in rotation with maize and high humidity and moisture through heading and maturity. It is primarily caused by *Fusarium graminearum* Schwabe, which infects spikes of wheat leading to the discoloration and deterioration of grain, and the contamination with mycotoxins, mainly deoxynivalenol (DON; Parry et al., 1995; Dexter, 1996; Argyris et al., 2003).

Control of FHB is difficult because of the complexity of the disease and the need for use of different management strategies has been proven (Bai and Shaner, 2004). Breeding for resistant cultivars should be a major part of an integrated approach to reduce the damage from FHB. In this sense, FHB adds complexity to the objective, because resistance is quantitatively inherited with many Quantitative Trait Loci (QTLs) involved (Liu et al., 2005). Breeding for resistance to a quantitative disease is a difficult task that requires multiple cycles of breeding, leading to a gradual improvement of resistance over time (Poland and Rutkoski, 2016). The use of molecular markers to track QTLs of interest in conjunction with phenotypic selection opened a new area, marker-assisted selection (MAS), that has been widely used since the early 2000s (Waldron et al., 1999; Van Sanford et al., 2001; Buerstmayr et al., 2003; Buerstmayr et al., 2009). The value of MAS for improving FHB resistance has been confirmed by many research studies (Miedaner et al., 2006; Anderson et al., 2007; Buerstmayr et al., 2009; Agostinelli et al., 2012; Miedaner and Korzun, 2012; Balut et al., 2013; Liu et al., 2013). However, attempts to improve complex quantitative traits by using QTL-associated markers is not completely successful because of the difficulty of finding the same QTL across multiple environments (due to QTL x environment interactions) or in different genetic backgrounds (Heffner et al., 2009; Bernardo, 2016; Crossa et al., 2017).

Genomic selection (GS) is a form of MAS that simultaneously estimates all locus, haplotype or marker effects across the entire genome to calculate genomic estimated breeding values (GEBVs) (Meuwissen et al., 2001). Since its inception, there have been many studies that demonstrate the utility of GS approaches in breeding for disease resistance in crops (Heffner et al., 2009; Lorenz et al., 2012; Rutkoski et al., 2012; Rutkoski et al., 2014; Poland and Rutkoski, 2016). In wheat, FHB resistance is a challenging breeding target due to the combination of quantitatively inherited resistance and a challenging phenotype that is not easy to reproduce artificially. Thus, GS provides a great opportunity to breed FHB-resistant wheat cultivars. Research evaluating the performance of GS on the prediction of FHB traits in wheat and barley (*Hordeum vulgare* L.) has produced some interesting results. Some studies have predicted GEBVs under a cross validation scheme (Rutkoski et al., 2012; Arruda et al., 2015; Jiang et al., 2015; Mirdita et al., 2015; Hoffstetter et al., 2016; Dong et al., 2018), while others have investigated the application of GS models under a forward selection scheme (Sallam and Smith, 2016; Jiang et al., 2017; Schulthess et al., 2018; Tiede and Smith, 2018; Herter et al., 2019).

While these studies have contributed information on the implementation of GS in a wheat breeding program, little information is found about building the training population with lines coming from regional scab nurseries. These nurseries provide multiyear data sets comprising breeding lines nominated yearly to be included in the Uniform Northern and Uniform Southern soft wheat scab nurseries (https://scabusa.org/publications#pubs_uniform-reports). These nurseries are evaluated in multiple locations every year, and several FHB traits are recorded at every location. Training the GS model with lines belonging to these data sets would enable breeders to rely on multilocation and multiyear data and allow them to predict GEBVs for lines of local programs based on a wider range of germplasm evaluated in many locations. Sarinelli et al. (2019) evaluated the use of a historical USA winter wheat panel to predict yield and agronomic traits under a cross validation scheme, and Dawson et al. (2013) evaluated the overall accuracy of genomic predictions for untested genotypes using an unbalanced dataset to train a genomic prediction model, but none of them included FHB traits as an objective of the research.

Several FHB traits have been under study and estimated with a GS model. The visual evaluation of the disease through FHB rating or FHB index, the product of incidence and severity, is the trait most often evaluated in different studies (Rutkoski et al., 2012; Arruda et al., 2015; Jiang et al., 2015; Mirdita et al., 2015; Hoffstetter et al., 2016; Schulthess et al., 2018; Herter et al., 2019) finding moderate to strong prediction accuracies. Another very important trait, that significantly affects grain quality and commercialization is DON content, a trait that has also received some attention in wheat (Rutkoski et al., 2012; Arruda et al., 2015) and barley (Lorenz et al., 2012; Sallam and Smith, 2016; Tiede and Smith, 2018). DON accumulation is a critical target for wheat breeders, and even though there is a general acceptance that breeding for low DON accumulation is improved by selecting lines based on visually scored traits (Buerstmayr and Lemmens, 2015), the contamination with *Fusarium* and DON on healthy looking grain has been observed and reported (Argyris et al., 2003). Some research has been also done on indirect selection for low DON contamination lines (Rutkoski et al., 2012; Sallam and Smith, 2016) but more research should be done to extend the estimation of GEBVs for indices including DON as part of the index.

The primary objective of this study was to evaluate the use of two unbalanced data sets, the Uniform Northern and Uniform Southern Scab Nurseries, in a forward GS scheme to predict GEBVs for FHB traits in lines from the UK wheat breeding program. As a second objective, we investigated the design of the training population with the regional scab nurseries separated or combined, using different TP sizes and different optimization methods to predict several FHB traits and indices. As a third objective, we evaluated the use of predicted GEBVs for indices to select for low DON content lines in comparison with selecting lines based on GEBVs for DON content.

## Materials and Methods

The plant material in this study comprised lines from the University of Kentucky soft red winter wheat breeding program, and the 2014–2018 Uniform Northern and Uniform Southern soft red winter wheat scab nurseries (NUS and SUS respectively; **Supplementary Table 1**).

We evaluated a population of 306 breeding lines from the University of Kentucky soft red winter wheat breeding program. Lines were derived from multiple F_4:5_ and F_4:6_ families and were evaluated in yield trials as part of the testing program. Two hundred twenty nine lines belonging to the NUS that represented elite germplasm from public and private breeding programs were evaluated in field environments from 2014 to 2018 (**Supplementary Table 1**). The data set was balanced for individual years where the same set of genotypes was evaluated across different locations and unbalanced between years. Another set of 223 lines was evaluated in field environments from 2014 to 2018; these experiments were part of the Uniform Southern scab nursery (SUS) and represented elite germplasm from public and private breeding programs. The data was balanced for individual years where the same set of genotypes was evaluated across different locations and unbalanced between years. A list of locations/year combinations for each regional nursery are shown in **Supplementary Table 1**).

The 306 breeding lines from the University of Kentucky were grown in Lexington, KY during the 2016–2017 growing season. Genotypes were planted in 1.2 m rows long, spaced 30 cm apart. The soil type at the site is a Maury silt loam (fine, mixed, semiactive, mesic typic Paleudalfs). The experiment was planted in a randomized complete block design with two blocks. Two checks, a resistant line (KY02C-3005-25) and a susceptible cultivar (Pioneer Brand 2555) were planted across the experiment. Sixty-six (66) of the total 306 lines that advanced in the breeding program based on grain yield, agronomic and disease profile, were also grown in Lexington, KY during the 2017–2018 growing season in the FHB nursery, under the same protocol explained before.

In both seasons, the FHB Nursery had an overhead mist irrigation system on an automatic timer that started three weeks before heading. The irrigation schedule was as follows: 5 min periods every 15 min from 2,000 to 2,045 h, 2,100 to 2,145 h, 0200 to 0245 h, 0500 to 0530 h, and 0830 h (Balut et al., 2013). The experiment was inoculated with *Fusarium graminearum* –infected corn (*Zea mays* L.) Inoculum comprised 27 isolates taken from scabby wheat seeds collected over the years 2007–2010 from multiple locations across Kentucky (Bec et al., 2015). The inoculum was prepared by allowing corn to imbibe water for approximately 16 h before autoclaving. After autoclaving, a solution of 0.2 g streptomycin in 150 ml sterile water was mixed in the corn to avoid the growth of other microorganisms. The corn was inoculated with potato dextrose agar (PDA) plugs containing *Fusarium graminearum*, covered and incubated for 2 weeks until fully colonized by the fungus. After that, the corn was spread on the floor until dry, and put in storage bags in a freezer until use. Approximately 3 weeks prior to heading, the scabby corn was spread in the rows at a rate of 11.86 g m^-2^ (Balut et al., 2013).

Each nursery cooperator submits his or her breeding materials for evaluation and conducts an inoculated FHB trial at this or her location following the protocols developed by the U.S. Wheat and Barley Scab Initiative (https://scabusa.org/) whose aim is to develop control measures against FHB.

### Phenotypic Evaluation

At 24 days after heading, FHB rating was recorded using a 0–9 scale. FHB rating is a visual estimate of the incidence and severity of the disease ranging from 0 (absence of FHB symptoms) to 9 (≥ 90% of FHB blighted spikelets). Heading date (HD) was recorded when 50% of the spikes in a row had emerged from the flag leaf sheath (in Julian dates; data not shown). Plant height (cm) was measured from the soil surface to the top of the spike, excluding awns (data not shown). Lines were manually harvested using a sickle, mechanically threshed and cleaned. After cleaning, a grain sample of approximately 15 g from each row was further cleaned by hand and evaluated for Fusarium damaged kernels (FDK). The percentage of FDK was estimated by visually comparing samples with known levels of FDK ranging from 10 to 90%. The same sample (15 g) was subsequently sent to the University of Minnesota DON testing laboratory for DON analysis. DON concentration was determined by gas chromatography with mass spectrometry (Mirocha et al., 1998; Dong et al., 2006) Two indices were created:

1. DSK index was created combining FHB rating, FDK percentage and DON content with the formula: FHB*0.2 + FDK* 0.3 + DON*0.5;
2. DK index was obtained combining FDK percentage and DON content with the formula: FDK*0.4+DON*0.6.

Both indices were created to emphasize the importance of kernels traits (FDK, DON) for breeding against FHB. The NUS and SUS data were obtained for every genotype, location, year combination. Lines were planted in a 1.2 m row spaced 30 cm with two blocks. A common check cultivar (Ernie) was planted in the NUS and SUS across years and locations. Historical data consisted of entry mean data for FHB rating, FDK and DON concentration for each combination of genotype/location/year.

### Data Analysis

The following linear mixed model was utilized for the analysis of the FHB traits for which individual row-level was available:

$${\text{Y}}_{\text{lk}}=\mu +{\text{B}}_{\text{k}}+{\text{G}}_{\text{l}}+\epsilon_{\text{kl}}$$

where μ was the mean, Y_lk_ was phenotypic observation of the *l*th genotype at the *k*th block, B_k_ was effect of the block, G_l_ was the effect of the genotype, and ϵ_kl_ represented the residual term. The overall mean and the genotypic effects were considered fixed and block term random. Best Linear Unbiased Estimators (BLUEs) were derived from the model above. For the historical data of the NUS and SUS nurseries, a single value of each line-environment combination was available for the different traits (FHB, FDK, DON). Therefore, the following linear mixed model was used for this data:

$${\text{Y}}_{\text{ijl}}=\mu +{\text{Y}}_{\text{i}}+{\text{L}}_{\text{j}}+{\text{YL}}_{\text{ij}}+{\text{G}}_{\text{l}}+{\text{YG}}_{\text{il}}+{\text{LG}}_{\text{jl}}+\epsilon_{\text{ijl},}$$

where μ was the mean, Y_ijl_ was phenotypic observation of the lth genotype at the ith year in the jth location, Y_i_ was the effect of the year, L_j_ was the effect of the location, G_l_ was the effect of the genotype and YG_il_ and LG_jl_ were the interaction terms year by genotype and location by genotype respectively. E_ijl_ represented the residual term. The overall mean and the genotypic effects were considered fixed and all the remaining terms random. The model above is the one from which BLUEs were derived.

### Genotyping

**Figure 1:**
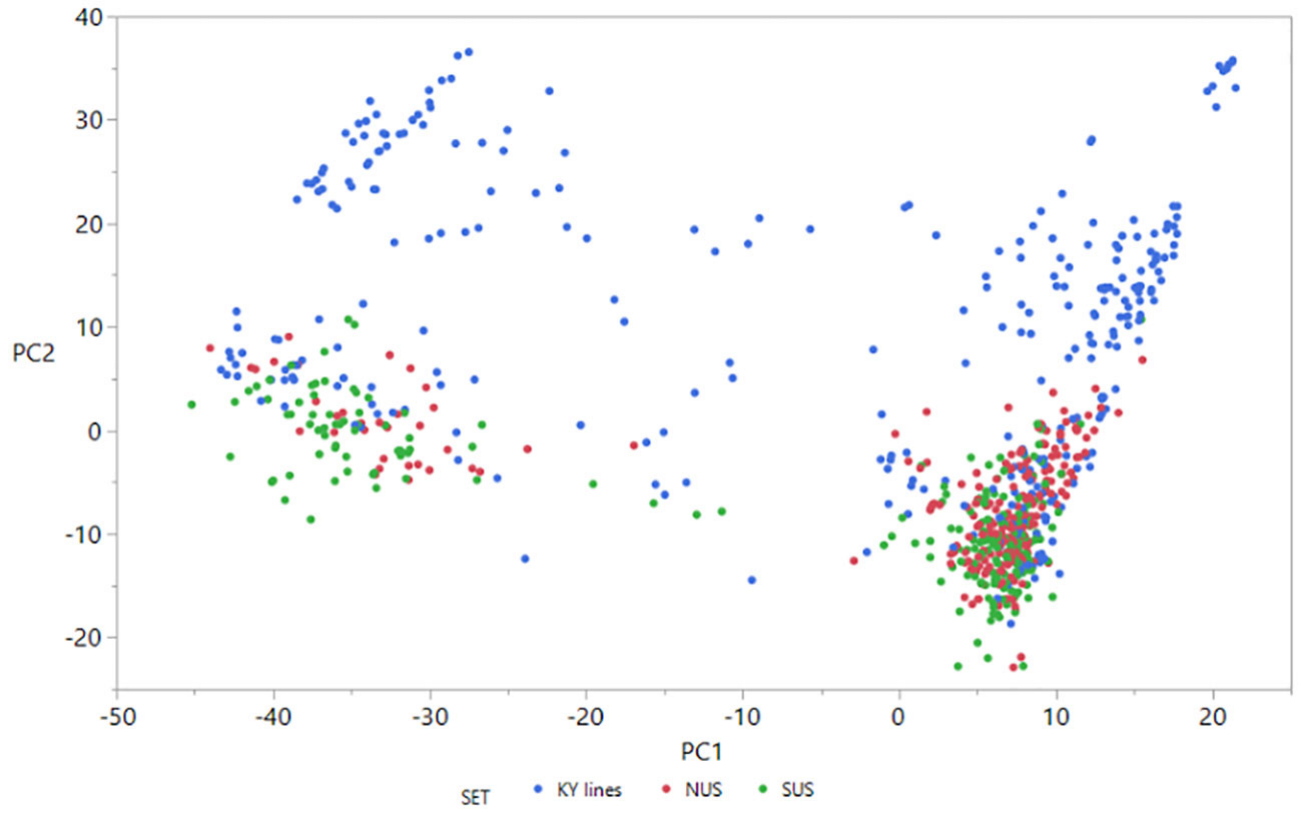
Scatter plot of the first two principal components from analysis of the 758 lines based on the full set of 20,929 SNPs. Different colors represent different sets of germplasm. PC1 = 6.1%; PC2 = 3.2%.

For the 306 breeding lines from the University of Kentucky wheat breeding program, DNA was extracted using the Sbeadex plant kit from BioSearch Technologies; using leaf samples from the F_4:5_ or F_4:6_ lines that were collected by sampling a minimum of eight 7–10 day old seedlings. Genotyping by sequencing (GBS) (Elshire et al., 2011) using the protocol described by (Poland et al., 2012) was conducted for the 758 lines that were phenotyped. Single nucleotide polymorphism (SNP) calling on raw sequence data was done with Tassel-5GBSv2 pipeline version 5.2.35. SNPs with ≤50% missing data, ≥5% minor allele frequency and ≤10% of heterozygous calls per marker locus were retained and imputation performed using Beagle v4.0. The final number of SNPs utilized for analysis was 20,929. With the genome wide marker information, a kinship matrix including the 758 lines was built in Tassel-5GBSv2. Principal components analysis was generated with Tassel-5 and the eigenvalues for PC1 and PC2 were plotted (**Figure 1**).

### Genomic Prediction

GEBVs for FHB rating, FDK, DON, DSK, and DK were estimated using ridge regression best linear unbiased prediction (RR-BLUP) (Meuwissen et al., 2001) with the model

y=Xβ+Zu+e

where y is a vector of BLUEs for one trait for each wheat genotype, β is a vector of fixed effects which includes the overall mean and fixed covariates (major QTL and association mapping markers), u is a vector of random marker effects, X and Z are the design matrices for fixed and random effects, respectively, and e is a vector representing residual terms. The variance–covariance structure associated with the random term was u~N (0, Iσ_u_^2^) and for the residual term was e~N (0, Iσ_e_^2^). The estimates of u were obtained from the mixed.solve function using the package RR-BLUP in R (Endelman and Jannink, 2012). Prediction accuracy was defined as the Pearson correlation between the phenotypic values (BLUEs) and the GEBVs (predicted) values.

### Design of the Training Populations and Validating Populations

To evaluate the NUS and SUS as possible TPs to estimate GEBVs for the UK breeding lines, we established two TP sizes of 100 and 200 lines, and three optimization methods to select the lines: Random, Two tails and Prediction Error Variance (PEVmean) (described below). Under these combinations, the NUS and SUS were used separately as the source of lines for the TP. A second strategy was to combine the lines of the NUS and SUS as training population. Data from the two nurseries comes from the same years in which they have environments in common (15) but no common lines were used from the two nurseries (with the exception of the moderately resistant check cultivar Ernie). For this approach, we evaluated four TP sizes (100, 200, 300, 400), and three different optimization methods: Random, Two tails and PEV. The validating populations (VP) were created by selecting 50 genotypes randomly from the total 306 breeding lines for each validating population, creating a total of 20 validation sets.

### Training Population Optimization Methods

As was described above, the effect of the TP size on the predictive ability of the genomic selection model was assessed using two (100, 200) and four (100, 200, 300, 400) different population sizes. For each population size, we implemented three approaches for comparison of training population selection:

#### Random

For this method, a random sample of genotypes was selected as training population for each TP size, varying the source of lines from which the random sample came. The same random sample was used for all validating populations.

#### Two Tails

To implement this method, fully described by Michel et al. (2017), we selected for each trait individually, the two tails of the phenotypic distribution for the NUS, SUS, or the combination of the two. For all of the TP sizes, we selected lines where 50% had the highest values for the trait, and 50% had the lowest values for the trait.

#### PEVmean

This approach utilized a training population optimization algorithm for each VP that minimized the mean prediction error variance (Rincent et al., 2012; Akdemir et al., 2015). The PEVmean algorithm used genomic information from all genotypes to measure the reliability of the GEBVs for individuals in the validation set. An optimal training population from all genotypes available was selected to minimize the mean prediction error variance in the validation set. We used the approach suggested by (Akdemir et al., 2015) for an efficient approximation to the prediction error variance using the first 100 principal components of the genotypes to estimate the genomic relationship matrix. The PEVmean strategy was implemented using the function “GenAlgForSubsetSelection” from the R package STPGA. Principal components were estimated from genotypic data, and the first 100 principal components were chosen for error variance estimation. The best training population for each of the 20 validation sets for each of the different population sizes and sources of lines to become the training population was selected after 300 iterations of the genetic algorithm parameter, while other parameters in the function were set with default values.

### Cross Validation

We first investigated the predictive ability of the genomic selection model for each of the three traits and two indices calculating the Pearson correlation between the phenotypic values (BLUES) and the GEBVs (predicted) across 100 iterations of cross validation. A random sampling cross validation was conducted, training the model with the NUS, SUS, the combination of both nurseries (NUS+SUS) and the set of KY lines. The cross validation randomly assigned 80% of the total lines to the TP and the other 20% lines to the VP.

### Selection of Lowest DON Content Lines Based on Ranking and Different Selection Intensity

The assessment of the DON content provided by the U Minnesota DON testing laboratory (phenotypic data) and the correlation with GEBVs obtained with GS was performed in this way: for each one of the twenty validating populations a ranking of the lines from lowest to highest DON was made based on BLUEs, and another ranking was made based on the GEBVs for DON content, DSK index and DK index obtained with GS. Different selection intensities were chosen: 20, 30, and 40%. Afterwards we calculated at the different selection intensities the percentage of lines with lowest DON levels that would have been also selected using only the GEBVs for DON, DSK, and DK. This approach was done for the 306 lines evaluated in 2017 and the 66 lines evaluated in both years (2017–2018).

## Results

### Principal Components Analysis (PCA)

The scatter plot of the first two principal components (**Figure 1**) shows that principal component 1 explained only 6.1% and PC2 only 3.2% of the genetic variance. PCA analysis revealed four groups of lines clustered together. Two clusters contain KY breeding lines and also NUS and SUS lines. The other two clusters were more scattered and contained only KY breeding lines. It is interesting that based on the 20,929 SNPs used, the lines belonging to the northern regional nursery grouped together with lines of the southern regional nursery.

### Phenotypic Summary

The sets evaluated in this study consisted of two sets of lines belonging to the NUS and to the SUS and a third set of lines that were breeding lines from the University of Kentucky wheat breeding program. The nurseries historical data comprised five years that were evaluated and curated to be analyzed. The phenotypic information (**Table 1**) for both nurseries, the total set of lines evaluated in Lexington, KY in 2017, and the subset of lines evaluated also in 2018 making two years of phenotype information, showed that good levels of infection were achieved, so that we were able to score genotypes and differentiate resistant and susceptible reactions for the different traits. The means for FHB rating, ranged from 3 in the SUS nursery to 4.5 in the breeding lines (17–18), with a minimum rating of 1–1.5 and a maximum rating scores of 7–8.5 in the four sets. The FDK percentage had an average of 28.3% for the NUS, 31.6% for the SUS, 37.2% for subset breeding lines, and 48.6% on the total KY set, showing an average higher value in 2017 in Kentucky compared with the mean of five years for the regional sets and two years also in KY. The FDK ranged from 8.7 to 62.8% in the NUS, 10.7 to 60.2% in the SUS, from 12 to 90% in the total KY set, and from 8.5 to 72.5% in the subset KY lines. The KY set in 2017 reached a higher maximum value for FDK agreeing with the higher FHB ratings reached. Regarding DON levels, the mean DON content was 8.6 ppm for the NUS, 9.5 ppm for the SUS, 16.6 ppm for the KY subset evaluated in two years, and 24 ppm for the KY set evaluated only in 2017. The DON values ranged from 1.1 to 25 ppm for NUS, 3.5 to 23.9 ppm in the SUS, 6.6 to 33.8 ppm for the KY subset, and a range of 11.1 to 51 ppm in the KY set. The DON levels in Lexington, KY in 2017 got our attention because they were higher than generally occurs. Despite these high values, we still could observe phenotypic variance among the evaluated lines. There was a range of 40 ppm between the lowest and highest values for the KY lines and the NUS and SUS showed a range of 20–24 ppm between the lowest and highest level of DON. DSK index and DK index were calculated based on these traits.

**Table 1:** Summary of the phenotypic information for FHB rating, FDK, and DON for the two regional nurseries (NUS and SUS) and the Kentucky breeding lines. In 2017 breeding lines are a total of 306, the average of 2017 and 2018 field season is for a subset of 66 lines.

	FHB rating (0-9)	FDK (%)	DON (ppm)		FHB rating (0-9)	FDK (%)	DON (ppm)
NUS (2014-2018)				Breeding lines (2017)			
Mean	3.4	28.29	8.56	Mean	4.1	48.62	24.92
Min	1.5	8.70	1.04	Min	1	12.00	11.10
Max	6.8	62.8	25.02	Max	8.5	90.00	51.40
SUS (2014-2018)				Breeding lines (2017-18)			
Mean	3.2	31.59	9.47	Mean	4.5	37.18	16.60
Min	1	10.70	3.50	Min	1.8	8.50	6.65
Max	7.5	60.15	23.90	Max	7.8	72.50	33.88

### Cross Validation

To evaluate the ability of the model to estimate the GEBVs for the different traits, 100 cycles of cross validation were run and prediction accuracy calculated (**Table 2**). Prediction accuracies were moderate for the different traits, except a low prediction accuracy for FHB rating in the SUS. Prediction accuracy for FHB ranged from 0.27 in SUS to 0.49 in the KY set. For FDK percentage, prediction accuracy ranged from 0.46 in the SUS to 0.60 in the NUS. Prediction accuracy for DON ranged from 0.49 in the SUS to 0.63 in the KY lines. Prediction accuracy for DSK index ranged from 0.49 in SUS to 0.64 in NUS and finally DK index ranged from 0.51 in the SUS to 0.64 in the NUS. For all traits, the SUS set obtained with cross validation produced lower prediction accuracies compared to NUS or the KY lines set.

**Table 2:** Mean prediction accuracy and standard deviation for the different traits and indexes with Cross Validation.

	N	FHB rating	FDK	DON	DSK	DK
NUS	229	0.47 ± 0.09	0.6 ± 0.09	0.59 ± 0.09	0.64 ± 0.08	0.64 ± 0.08
SUS	223	0.27 ± 0.12	0.46 ± 0.11	0.49 ± 0.1	0.49 ± 0.09	0.51 ± 0.09
NUS+SUS	452	0.41 ± 0.08	0.57 ± 0.07	0.58 ± 0.06	0.62 ± 0.06	0.63 ± 0.06
KY Lines	306	0.49 ± 0.10	0.51 ± 0.09	0.63 ± 0.06	0.58 ± 0.07	0.55 ± 0.09

N is the set size. FHB rating (0–9), FDK (%), DON (ppm), DSK index, DK index.

### The Regional Nurseries as Training Populations to Predict the Kentucky Breeding Lines

The first question we wanted to investigate with this research was the value of the regional nurseries as source of lines and information to train a model for genomic prediction for local breeding programs (**Tables 3** and **4**). The use of the NUS or the SUS as a TP to calculate GEBVs for the Kentucky breeding lines showed different responses depending on the trait. As a general conclusion from **Table 3**, we obtained positive and moderate prediction accuracies for all traits. The SUS was the best source of lines to estimate GEBVs for DON content, obtaining prediction accuracies of 0.4 with TP size 200 for the three optimization methods. FDK, FHB rating, DSK index, and DK index were better predicted by the NUS; the highest prediction accuracies were reached with a TP size=200 regardless of the optimization method. For FDK, average prediction accuracy was 0.4 and prediction accuracies of 0.33 and 0.46 for FHB rating and DSK index were found respectively.

**Table 3:** Mean prediction accuracy and standard deviation for the different traits and index with two different Training Populations = NUS and SUS; two TP sizes = 100, 200 and three different TP optimization methods = Random, Two Tails and Prediction Error Variance. FHB rating (0–9), FDK (%), DON (ppm), DSK index, DK index.

		NUS		SUS	
		100	200	100	200
	Random	0.25 ± 0.12	0.33 ± 0.13	0.35 ± 0.09	0.4 ± 0.09
DON	TT	0.24 ± 0.12	0.29 ± 0.11	0.37 ± 0.10	0.41 ± 0.12
	PEV	0.38 ± 0.07	0.34 ± 0.11	0.33 ± 0.12	0.44 ± 0.10
	Random	0.37 ± 0.1	0.38 ± 0.13	0.28 ± 0.11	0.29 ± 0.09
FDK	TT	0.33 ± 011	0.4 ± 0.09	0.31 ± 0.10	0.3 ± 0.10
	PEV	0.37 ± 0.10	0.4 ± 0.08	0.25 ± 0.10	0.31 ± 0.10
FHB	Random	0.3 ± 0.13	0.32 ± 0.13	0.26 ± 0.13	0.23 ± 0.12
rating	TT	0.3 ± 0.13	0.33 ± 0.12	0.19 ± 0.12	0.27 ± 0.12
	PEV	0.31 ± 0.12	0.33 ± 0.12	0.19 ± 0.12	0.26 ± 0.12
	Random	0.43 ± 0.11	0.45 ± 0.11	0.46 ± 0.11	0.43 ± 0.09
DSK	TT	0.38 0.13	0.47 ± 0.10	0.45 ± 0.10	0.45 ± 0.10
	PEV	0.46 ± 0.08	0.46 ± 0.09	0.35 ± 0.12	0.43 ± 010
	Random	0.41 ± 0.09	0.42 ± 0.09	0.45 ± 0.10	0.44 ± 0.06
DK	TT	0.36 ± 0.14	0.41 ± 0.11	0.46 ± 0.08	0.43 ± 0.08
	PEV	0.43 ± 0.09	0.44 ± 0.08	0.36 ± 0.11	0.43 ± 0.08

**Table 4:** Mean prediction accuracy and standard deviation for the different traits and index with a combined Training Population = NUS + SUS; four TP sizes = 100, 200, 300, 400 and three different TP optimization methods = Random, Two Tails and Prediction Error Variance. FHB rating (0–9), FDK (%), DON (ppm), DSK index, DK index.

		NUS + SUS			
		100	200	300	400
	Random	0.24 ± 0.13	0.43 ± 0.1	0.43 ± 0.1	0.43 ± 0.09
DON	TT	0.38 ± 0.10	0.4 ± 0.09	0.42 ± 0.09	0.4 ± 0.10
	PEV	0.42 ± 0.12	0.42 ± 0.10	0.42 ± 0.09	0.41 ± 0.10
	Random	0.37 ± 0.10	0.34 ± 0.08	0.39 ± 0.09	0.41 ± 0.09
FDK	TT	0.35 ± 0.10	0.35 ± 0.09	0.37 ± 0.09	0.38 ± 0.09
	PEV	0.35 ± 0.09	0.37 ± 0.09	0.38 ± 0.08	0.38 ± 0.08
FHB	Random	0.23 ± 0.11	0.35 ± 0.11	0.3 ± 0.11	0.35 ± 0.10
rating	TT	0.24 ± 0.10	0.28 ± 0.12	0.35 ± 0.11	0.33 ± 0.10
	PEV	0.27 ± 0.12	0.32 ± 0.11	0.34 ± 0.11	0.35 ± 0.11
	Random	0.42 ± 0.10	0.46 ± 0.08	0.48 ± 0.07	0.49 ± 0.07
DSK	TT	0.36 ± 0.12	0.44 ± 0.1	0.49 ± 0.08	0.49 ± 0.07
	PEV	0.46 ± 0.08	0.46 ± 0.09	0.48 ± 0.07	0.48 ± 0.07
	Random	0.43 ± 0.08	0.46 ± 0.07	0.47 ± 0.06	0.48 ± 0.06
DK	TT	0.36 ± 0.09	0.38 ± 0.1	0.44 ± 0.1	0.47 ± 0.06
	PEV	0.45 ± 0.07	0.44 ± 0.07	0.46 ± 0.06	0.46 ± 0.06

When lines of the NUS were the TP source, TP size showed an effect when increased from 100 to 200 for all traits and optimization methods. Only for DON when the optimization method was PEVmean did we find a decrease in the prediction accuracy as TP size was increased from 100 to 200 TP. All other traits under different optimization methods showed increases in prediction accuracy ranging from 1% (DSK, PEV) to 32% (DON, Random). As an average among traits, the two tails optimization method showed the highest increase: 18% when the TP size increased from 100 to 200 individuals. The random method showed a 10% increase and the PEV optimization method showed a 1% increase. But it is important to mention that at TP=100, PEVmean showed the highest prediction accuracy for all traits. When the SUS lines were the TP source, the TP size had a positive effect only for DON when increased from 100 to 200 for the three optimization methods. We found a positive effect with Random and PEV optimization methods when predicting FDK and a positive effect with Two Tails and PEV optimization methods when predicting FHB rating. On average, by optimization method, PEVmean showed an increase of 26% in prediction accuracy when TP size increased from 100 to 200. Two tails showed an 8% increase and Random did not change when TP size went from 100 to 200.

**Table 4** shows the prediction accuracies for the same traits and index when we combined NUS and SUS as a source of lines for the TP. As an overall conclusion there was a significant effect of the TP size, showing a good response when the TP was increased up to 400 lines. Overall, DSK index had the highest prediction accuracies with 0.49 for two optimization methods, Random and Two Tails, and 0.48 for PEVmean. On the other hand, FHB rating showed the lowest prediction accuracy with TP size =100, for the three optimization methods, with an average of 0.24. For all traits, the increase in TP size showed positive effects in prediction accuracies. FDK, FHB rating, DSK, and DK showed increases in prediction accuracy under the three optimization methods. For FDK the increase was between a 9–11%, for FHB rating ranged from 32 to 53%, for DSK ranged from 6 to 35% and for DK ranged from a 3 to 31%. To predict DON, with the random method there was a big jump from TP 100 to TP 400 (82% increase) but with two tails and PEVmean there is a slight decrease of prediction accuracy: 2% with PEV and 7% with the two tails optimization method.

No significant differences in prediction accuracy were found among TP optimization methods by trait. Despite this result, choosing lines at random showed the highest increase from 100 to 400 TP size; this was especially due to the high increment for DON and FHB rating with prediction accuracies of 0.23–0.24 with a TP size 100 vs. 0.35–0.43 with a size of 400 individuals in the TP. When we looked at averages by TP size, both indices, DSK and DK showed the highest prediction accuracies at the four TP sizes, ranging from 0.41 to 0.49 for DSK and ranging from 0.41 to 0.47 for DK. FHB rating showed the lowest prediction accuracies at the four TP sizes ranging from 0.24 to 0.34. Finally, at the smallest TP size, TP=100, PEVmean showed the highest prediction accuracies for the two traits (FHB rating and DON) and two indices.

### Impact of Different Selection Intensities and Different Predicted Traits on the Identification of Lines With Low DON Accumulation

**Figure 2:**
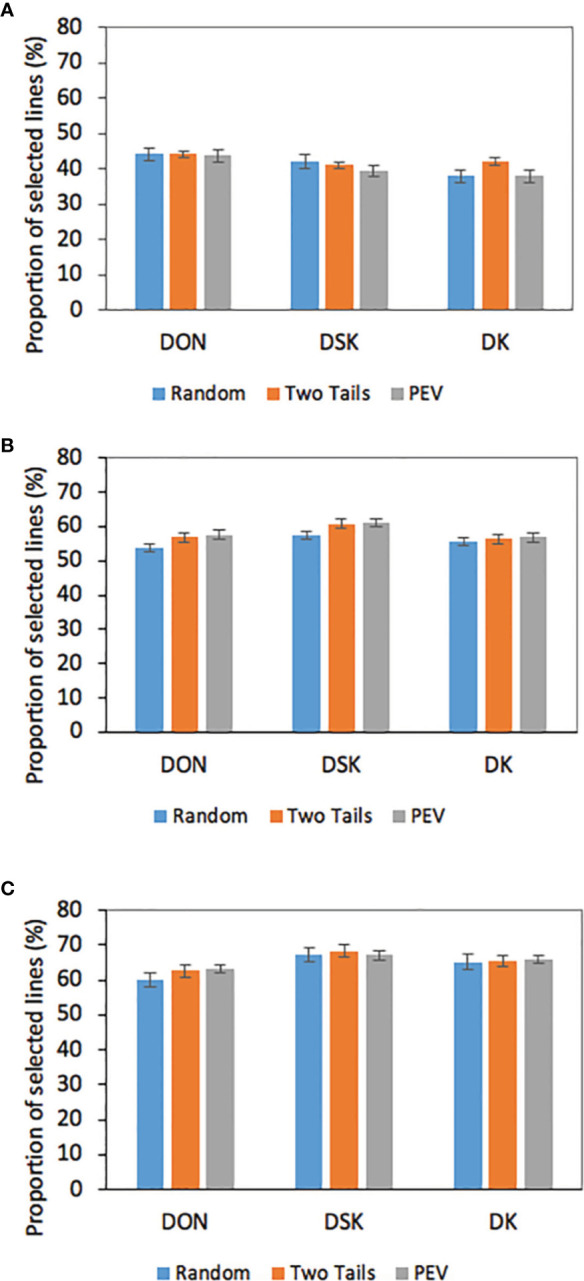
Proportion of correctly selected lines for low DON based on GEBVs for DON, DSK, and DK at different selection intensities (**A** = 20%; **B** = 30%; **C** = 40%) DON BLUES based on 2017 phenotypic data GEBVs calculated with TP = 400 and three different TP optimization methods.

We analyzed the impact of selection based on predicted breeding values for a critical trait, DON content on the 306 breeding lines evaluated in 2017 (**Figure 2**). It has been mentioned before that reducing DON content in wheat is a central objective of the breeding program; genomic estimate breeding values (GEBVs) for tested and untested lines would only add information the breeder could use to make selections and advance lines in the breeding program. We evaluated GEBVs obtained for DON, DSK, and DK with a TP=400 and with the three optimization methods, which yielded the highest prediction accuracies for the three traits (0.40–0.49). The results showed that a selection intensity (SI) of 20% resulted in an average of 44% lines that were correctly selected based on the GEBVs for DON compared with the ones selected based on BLUES; 41% were correctly selected based on the DSK index and a 39% were correctly selected based on DK. With an SI of 30%, being conservative to keep lines for further evaluation, 56% were correctly selected based on GEBVs for DON and DK and a 60% of the lines were correctly selected based on DSK. Finally, with an SI of 40%, 68% of lines were correctly selected based on DSK index, 66% based on DK and 62% based on DON GEBVs.

Our results show that to select for low DON in wheat lines, the breeder should focus on not only GEBVs for DON, but DSK index. In the current study, DSK index was an excellent source of additional information: at selection intensities of 0.3 and 0.4 this index picked up lines with low BLUES for DON at a 60 and 68% average respectively, 4–6% more than selection based on DON GEBVs. In contrast, at 20% SI, 44% of the selected lines based on DON GEBVs were correctly selected, 3% more than selection based on DSK.

**Figure 3:**
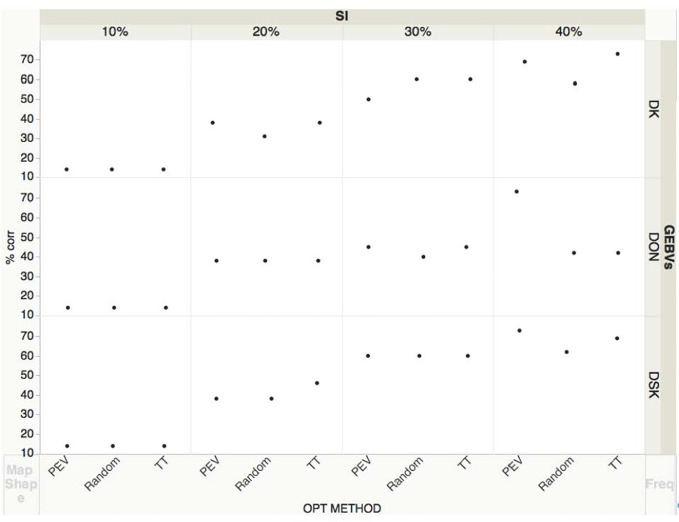
Correlations of line ranks based on DON BLUEs and GEBVs for DON, DK and DSK at different selection intensities (SI= 0.1, 0.2, 0.3, 0.4). DON BLUES based on two years (17–18) phenotypic data for a subset of KY lines. GEBVs obtained with a TP size= 400 and three optimization methods: Random, Two-tails and PEVmean. The three black dots correspond to each optimization method that trained the model.

We also analyzed all predicted traits and correlated them with DON BLUES for the lines evaluated in two years, 17–18 (**Figure 3**). With a SI of 20%, an average of 41% lines were correctly selected based on GEBVs for DON and DSK compared with the ones selected based on BLUES while 36% were correctly selected based on DK. With a SI of 30%, an average of 43% of lines were correctly selected based on GEBVs for DON, 60% of lines were correctly selected based on DSK and 57% of lines based on DK index. With a SI of 40%, an average of 52% lines were correctly selected based on GEBVs for DON, 67% based on DK index and 68% based on DSK index. Shown in **Figure 3**, the three optimization methods performed similarly in terms of accuracy to identify the best performing lines, except for GEBVs obtained for DON, where at SI of 40%, PEV method performed significantly better predicting correctly 73% of lines, compared to 42% with two tails or random. When selecting with highest SI, 10%, the percentage of correctly selected lines is low—only 14% of low DON lines were correctly identified. As an overall conclusion, at higher SI, 10-20%, the three traits DON, DSK, DK performed similarly in identifying the lowest content DON lines based on GEBVs with the proportion of selected lines not exceeding the value expected by the prediction accuracy. At lower SI, 30–40%, DSK and DK indices were promising with up to a 70% of the lines correctly selected.

## Discussion

Genomic selection has become a primary technology for plant breeders looking to accelerate the breeding process. Some of the benefits of GS pursued by breeders include increasing genetic gain per unit time, reduced phenotyping costs, reducing field testing and more accurate selection of parents for crosses. Another big impact of this strategy based on genome wide markers (GWM) is the possibility of breeding for quantitative traits with better outcomes than with marker assisted selection (MAS), because in contrast to MAS, the use of whole-genome prediction models generally has greater power to capture small-effect loci that would be missed by MAS because of limited power for declaring significant marker effects (Heffner et al., 2009).

The ability to improve FHB resistance through genomic selection has been studied and reviewed in recent years (Poland and Rutkoski, 2016; Steiner et al., 2017). A big question that needs more attention is the success of GS when the training population is a sample independent of the validating population. In our study, we tried to shed light on this question, investigating the use of the regional scab nurseries in which breeding lines are submitted every year by breeders from public or private programs. These nurseries are evaluated in multiple environments in the eastern soft red winter wheat region of the United States.

Overall, results from our study show that it is possible to use historical scab nurseries as TP to predict FHB traits. The study also showed encouraging results regarding the use of GEBVs for indices as indirect selection criteria for low DON genotypes. FHB traits are complex, highly polygenic and their expression is under a great environmental influence, which makes the phenotyping more difficult and costly.

The model’s predictive ability, with cross validation, showed moderate prediction accuracies for the different traits, varying from 0.27 to 0.49 for FHB rating, 0.46 to 0.6 for FDK, 0.49 to 0.63 for DON. These results are in agreement with studies that used cross validation in wheat; for FHB rating, (Arruda et al., 2015; Mirdita et al., 2015; Dong et al., 2018) found a 0.5 prediction accuracy, similar to our best estimation of this trait (KY lines). A lower value of 0.37 was found by Hoffstetter et al. (2016) for the same trait. For FDK, our moderate prediction accuracies differed from Arruda et al. (2015) who found a 0.8 prediction accuracy and Rutkoski et al. (2012) who found prediction accuracies ranging from 0.35 to 0.46 for the same trait. Investigators have found low and moderate values for DON, ranging from 0.24 to 0.64 (Rutkoski et al., 2012; Arruda et al., 2015; Dong et al., 2018). Our results fall within this range with prediction accuracies ranging from 0.49 to 0.63.

The two indices created to predict levels of scab resistance yielded moderate prediction accuracies: 0.49 to 0.64 for DSK and 0.51 to 0.64 for DK. Arruda et al. (2015) evaluated two different indexes (FHB index and ISK) finding prediction accuracies of around 0.5 for FHB index and 0.7 for ISK. In another study, Rutkoski et al. (2012) found for ISK prediction accuracies from 0.44 to 0.54. Our indices included DON as part of the index, a critical trait that is important to breeders, farmers, and the entire industry. Many studies have tried to select lines for low DON based on indices with incidence, severity and FDK (Rutkoski et al., 2012), traits that correlate very well with DON accumulation (Buerstmayr and Lemmens, 2015). In our study we calculated GEBVs for DON and two indices that included DON and found very good prediction accuracies for both indices as well as good agreement with DON BLUES (**Figures 2** and **3**).

Training population size can have a critical effect on prediction accuracies and it is a major issue for breeders as it relates to the genotyping and phenotyping efforts and the costs associated with them. In this study, we obtained the numerically highest prediction accuracies with a TP size of 400 although there was not a statistically significant difference between the TP of size 400 and the TP size of 300 for any trait (**Table 4**). The prediction accuracies with this TP size ranged from an average of 0.35 for FHB rating, to 0.49 prediction accuracy for DSK (**Table 4**). Our results agree with other studies regarding TP size, even those studies running cross validation schemes, as opposed to our forward prediction scheme. Our study presents a novel approach, investigating the effect of the TP size with a population of lines (NUS + SUS) independent of the VP, in our case the set of KY breeding lines. Applying forward GS, Lorenz et al. (2012) found the highest prediction accuracies up to 0.7 for DON and FHB rating in barley with a TP size of 300 individuals and Arruda et al. (2015) in a cross validation study found also an increasing prediction accuracy reaching a TP size 224. Herter et al. (2019) obtained for FHB a prediction accuracy of 0.8 with a TP size of 160 running a fivefold cross-validation and for *Septoria tritici* blotch the same study found a prediction accuracy of 0.5 at the same TP size. Sarinelli et al. (2019) found for powdery mildew infection a prediction accuracy of around 0.55 with a cross validation scheme when the TP size was increased from 50 to 350. Other studies also showed achieving highest prediction accuracies with TP sizes of 300–350 (Asoro et al., 2011; Lorenz et al., 2012; Lehermeier et al., 2014; Isidro et al., 2015; Rutkoski et al., 2015; Michel et al., 2017; Sarinelli et al., 2019).

Optimization methods to select the training population have also received attention from breeders and scientists because the lines selected to train the model are critical in obtaining GEBVs accurate enough to be used in the breeding program. We did not find significant differences among any of the three optimization methods; rather we found the highest prediction accuracies at TP size 400 for the three optimization methods. We did observe that at TP100, higher prediction accuracies were obtained with PEV compared to Random and Two-Tails for all traits, showing that at the lowest TP size, combining lines from the two nurseries and using the PEV method allows one to achieve prediction accuracies similar to the ones at TP400. Sarinelli et al. (2019) found similar results at low TP sizes. Selecting at random, prediction accuracies with TP 100 were lower compared with PEV but the most significant increase in prediction accuracies was achieved for all traits and all methods by increasing the TP size to 400 lines. The phenotypic selection of the two tails of the distribution for each trait of interest to define the TP was evaluated by Michel et al. (2017) for grain yield and protein content. They found a slight (5%) increase in prediction accuracy compared to random optimization method for both traits (r=0.39 for grain yield, and r= 0.55 for protein content). This was observed especially at small TP sizes. In our study, two-tails optimization TP method did not show a significant effect on prediction accuracy. On the other hand, a method to design the training population based on reduction of PEV mean of the validation set was more accurate compared with methods that selected individuals at random or by two tails. This is especially true with small training population sizes, because they better accounted for the relationship between the individuals in the training population and the validation set (Habier et al., 2010).

The importance of relatedness between TP and VP has been extensively discussed in the literature, and higher prediction accuracies are always associated with closer relationships between individuals in the TP and VP. In studies where cross validation is performed within populations of sibs or half sib, positive and moderate to high prediction accuracies have been found (Zhao et al., 2012; Hickey et al., 2014; Lehermeier et al., 2014; Herter et al., 2019). When the distance between individuals in the TP and VP is larger, e.g. using panels of lines with some kind of relatedness but not parent-offspring or sibs, using cross validation, only moderate or moderate to low prediction accuracies have been found in different crops especially for complex traits like yield. There are studies applying a forward GS scheme, where TP and VP are independent samples but with related material. In barley, for example, prediction accuracies for DON ranged from 0.14 to 0.67 and for FHB rating ranged from 0.58 to 0.77 (Lorenz et al., 2012; Tiede and Smith, 2018). In wheat, using an independent sample for TP and VP, (Jiang et al., 2017) found prediction accuracy of 0.58 for FHB rating, using a TP and VP evaluated in different years, for a sets of European wheat populations. They found a small difference, only 8% compared with prediction accuracies obtained with cross validation. In another study in wheat, (Hoffstetter et al., 2016) evaluated both cross validation and forward GS for FHB rating, and found that when the TP was predicting the parent lines of that TP, prediction accuracy ranged from 0.14 (unweighted) to 0.47 (weighted). When the VP consisted lines that shared some pedigree relationship to the TP, the prediction accuracy was 0.22 for the same trait. Similarly, (Schulthess et al., 2018) found prediction accuracies ranging from 0.4 (lower relatedness between TP and VP) to 0.8 (higher relatedness between TP and VP) when predicting FHB severity in hybrid wheat. Another study investigating the application of GS in a forward scheme, was performed by Herter et al. (2019) where they evaluated FHB rating and other traits both in a cross validation and across populations for all populations included in the study. When they predicted across populations, where many populations where half sibs, they found prediction accuracies ranging from -0.2 to 0.5 for FHB rating, showing substantial variation among 30 possible combinations. Our results agree with those of Herter et al. (2019) who suggested that for breeding for disease resistant traits the relatedness between TP and VP is critical to achieve good prediction accuracy. Because of the complexity of the traits evaluated in the present study, we surmise that the relatedness among the regional nurseries and the KY material, though being related germplasm due to the exchange between breeders, is not enough to overcome the threshold of 0.5 prediction accuracy we observed. The PCA (**Figure 1**) showed the association between lines that are clustered in four groups, and while we could see good association between lines in the two regional nurseries, all clustered in two groups, we observe for the KY lines more variability and association of lines in four clusters. This point is critical in our study and it is a real situation breeders face. Even though cross validation shows exciting prediction accuracies (Lorenz et al., 2012; Rutkoski et al., 2012; Arruda et al., 2015; Mirdita et al., 2015; Sallam and Smith, 2016; Schulthess et al., 2018); the use of historical data from regional nurseries, as our results confirm, offers breeders an excellent tool to estimate GEBVs for lines that have not been evaluated in the field for a specific trait. This reduces phenotyping costs tremendously because the TP phenotypic data set is generated by a collaborative effort among different breeding programs (Rutkoski et al., 2015; Sarinelli et al., 2019) or international breeding efforts (Dawson et al., 2013). Therefore, material in early generations may be selected or discarded based on GEBVs for DON without a DON content analysis, which allows breeders to reallocate the budget for DON analysis of more advanced material in the breeding program.

When breeding for FHB resistance, all traits are of critical importance and increasing FHB resistance in the germplasm implies improving all of them. Some authors have shown that measuring incidence, severity and FDK in the field, because of the high correlation with DON content, allows selection for these traits with easier evaluation and data recording compared with DON (Rutkoski et al., 2012; Buerstmayr and Lemmens, 2015; Sallam and Smith, 2016). Our study tried to move one step beyond this in understanding the weight DON has in an index, and the results we get after the DON content analysis could be used together with other field recorded traits to obtain the most accurate GEBVs. Both indices were created with the idea that DON is a trait of critical importance with food safety concerns and big economic losses to farmers (Bai and Shaner, 2004) and that evidence showed that DON contamination has been found even in healthy looking grain and that the DON accumulation has been found occurring during grain filling specially during wet grain filling periods (Argyris et al., 2003; Del Ponte et al., 2007; Bianchini et al., 2015). Our results showed that the highest prediction accuracies were obtained for DSK (0.49) and DK (0.47) and for DON (0.42) in third place. In **Figure 2**, we analyzed the impact of these prediction accuracies when selecting lines based on DON BLUEs, and we observed an increase in proportion of lines correctly selected (up to 70%) when reducing the selection intensity, and with an advantage of 4–8% when selecting lines based on DSK index compare to DON index. These results confirm the usefulness of multiple trait indices as a source of information to distinguish the best genotypes for a trait, and also shows again that a prediction accuracy of 0.49 for example should be considered in terms of the percentage of lines “correctly” selected by the GS model, as discussed by Bassi et al. (2015) and Verges and Van Sanford (2020). In our study again with a prediction accuracy of 0.49, a 50 to 70% of the lines are correctly selected at 30–40% SI. In early stages of field testing, when the breeder has many hundreds or even thousands of lines for yield evaluation, the ability to select based on GEBVs for a trait like DON would become an exceptional tool when scab resistance is a critical objective in the breeding program. This early cycle of selection based on GEBVs will allow one to include in the scab nursery in the following year only lines with an acceptable level of resistance to DON accumulation and overall FHB resistance.

Our study validates the use of DON related indices in applying GS for low DON (**Figure 3**). We observed that up to a 70% of lines were correctly selected based on DSK when a 40% selection intensity was used, in comparison to a 52% success rate for lines based on DON GEBVs, using two years of phenotypic data. Further, our results strongly support the use of the regional scab nurseries as a source of lines for training the GS model to predict FHB traits. This strategy can be implemented by breeding programs that belong to the regions where these scab nurseries are planted over years and multi locations data of hundreds of lines together with the possibility to predict GEBVs for expensive traits like DON content.

This study involved a complex scheme for GS, that included forward GS, historical data sets building the TP, three optimization methods, multiple TP sizes and the evaluation of three traits and two indices to improve and hasten breeding for FHB resistance. While these results are encouraging, we conclude that the relatedness between TP and VP becomes a critical issue if one wants to exceed a prediction accuracy of 0.5. In spite of this concern we are optimistic that the use of regional nurseries to predict scab traits will be useful from a breeder’s standpoint and allow one to predict scab resistance prior to actual phenotypic evaluation. For a resource intensive breeding target like scab resistance, this is a huge consideration.

## Data Availability Statement

The raw data supporting the conclusions of this article will be made available by the authors, without undue reservation.

## Author Contributions

VV and DS conceived and designed the experiments. VV performed the experiments. JL generated hapmaps and assisted with R coding. YD perform DON analysis. VV and DS analyzed the data. VV wrote the paper. DS edited it. All authors contributed to the article and approved the submitted version.

## Funding

This work was funded by a grant from the U.S. Department of Agriculture, through the US Wheat and Barley Scab Initiative under agreement no. 59-0206-9-054.

## Disclaimer

The founding sponsors had no role in the design of the study; in the collection, analysis, or interpretation of data; in the writing of the manuscript, and in the decision to publish the results.

## Conflict of Interest

The authors declare that the research was conducted in the absence of any commercial or financial relationships that could be construed as a potential conflict of interest.
