# Supplementary material for: Training Population Design With the Use of Regional Fusarium Head Blight Nurseries to Predict Independent Breeding Lines for FHB Traits
Source: Front Plant Sci. 2020 Jul 16;11:1083. doi: 10.3389/fpls.2020.01083 (PMC7381120; doi:10.3389/fpls.2020.01083)
Supplement: Supplementary file 1 [file DataSheet_1.pdf]

Figure S1: Uniform Northern and Uniform Southern Scab Nursery (NUS-SUS) for 2014-2018. Each environment is a combination of year/location. Severity, FDK and DON data is the average between two replications for each genotype.

NA: Data not recorded

YEAR	TEST	ENV	ENTRY	GENOTYPE	SEVERITY	FDK	DON
2014	NUS	2014_VA_BLA	1	TRUMAN	13.7	2.7	1.2
2014	NUS	2014_VA_BLA	2	ERNIE	20.5	4.5	0.5
2014	NUS	2014_VA_BLA	3	FREEDOM	28.1	15.0	4.6
2014	NUS	2014_VA_BLA	4	PIONEER2545	57.9	18.3	6.3
2014	NUS	2014_VA_BLA	5	NY01016-AN	50.0	12.1	2.7
2014	NUS	2014_VA_BLA	6	NY01066-278	46.0	16.5	7.5
2014	NUS	2014_VA_BLA	7	NY99059-249	42.5	6.3	1.3
2014	NUS	2014_VA_BLA	8	NY99069-249	31.2	7.5	3.1
2014	NUS	2014_VA_BLA	9	NY99069-352	51.2	12.9	4.6
2014	NUS	2014_VA_BLA	10	KWS023	14.0	5.0	0.4
2014	NUS	2014_VA_BLA	11	KWS024	39.7	25.5	4.0
2014	NUS	2014_VA_BLA	12	KWS025	74.5	6.4	3.0
2014	NUS	2014_VA_BLA	13	KWS028	55.2	7.6	1.8
2014	NUS	2014_VA_BLA	14	L29230	18.5	7.5	0.4
2014	NUS	2014_VA_BLA	15	LCS321	41.0	6.7	3.5
2014	NUS	2014_VA_BLA	16	E6012	48.7	2.7	1.9
2014	NUS	2014_VA_BLA	17	F0036R	65.2	18.4	7.1
2014	NUS	2014_VA_BLA	18	F0039	70.5	25.6	7.1
2014	NUS	2014_VA_BLA	19	F1014	20.1	21.0	3.6
2014	NUS	2014_VA_BLA	20	OH07-263-3	22.2	13.7	1.3
2014	NUS	2014_VA_BLA	21	OH08-206-69	64.5	4.5	1.6
2014	NUS	2014_VA_BLA	22	OH08-269-58	41.5	21.2	2.6
2014	NUS	2014_VA_BLA	23	0570A1-2-32-5-1-4	26.9	14.8	1.5
2014	NUS	2014_VA_BLA	24	0762A1-2-8	19.6	5.6	0.7
2014	NUS	2014_VA_BLA	25	08334A1-31	22.0	6.0	0.7
2014	NUS	2014_VA_BLA	26	10641B1-9-11-7	21.8	3.5	0.4
2014	NUS	2014_VA_BLA	27	B08-91993	20.3	1.0	0.8
2014	NUS	2014_VA_BLA	28	B09-900256	45.1	11.5	3.7
2014	NUS	2014_VA_BLA	29	M09L-9547	44.8	7.5	2.6
2014	NUS	2014_VA_BLA	30	M10-1100	29.9	17.0	4.8
2014	NUS	2014_VA_BLA	31	M11-1027	39.1	19.5	3.0
2014	NUS	2014_VA_BLA	32	M11-2298	28.6	8.5	2.1
2014	NUS	2014_VA_BLA	33	GL133	18.5	3.8	3.0
2014	NUS	2014_VA_BLA	34	GL164	24.8	15.0	3.4
2014	NUS	2014_VA_BLA	35	UGRCC2-78	50.1	13.0	4.9
2014	NUS	2014_VA_BLA	36	UGRCC5-116	42.2	19.0	0.6
2014	NUS	2014_VA_BLA	37	IL09-24328	21.4	2.9	0.2
2014	NUS	2014_VA_BLA	38	IL09-3264	12.6	5.0	0.1
2014	NUS	2014_VA_BLA	39	IL10-19464	12.9	3.0	0.7
2014	NUS	2014_VA_BLA	40	IL10-6855	15.9	4.0	1.1
2014	NUS	2014_VA_BLA	41	KY05C-1020-4-6-5	21.8	9.0	1.7
2014	NUS	2014_VA_BLA	42	KY05C-1105-43-6-1	26.9	5.1	1.7

2014	NUS	2014_VA_BLA	43	KY06C-3003-43-13-3	27.7	8.0	3.2
2014	NUS	2014_VA_BLA	44	KY204604	51.9	19.0	5.2
2014	NUS	2014_VA_BLA	45	MD08-22-22-13-4	13.2	2.0	0.7
2014	NUS	2014_VA_BLA	46	MD08-22-22-13-10	11.8	4.1	1.4
2014	NUS	2014_VA_BLA	47	MD09W272-8-4-13-3	27.6	3.5	3.3
2014	NUS	2014_VA_BLA	48	MDC07026-12-28	13.1	2.5	0.1
2014	NUS	2014_VA_BLA	49	MO120194	11.9	7.5	0.4
2014	NUS	2014_VA_BLA	50	MO120452	16.1	7.0	1.2
2014	NUS	2014_VA_BLA	51	MO120794	19.6	3.9	2.1
2014	NUS	2014_VA_BLA	52	MO121183	36.9	5.4	2.4
2014	NUS	2014_VA_BLA	53	NE06545	27.8	19.5	4.1
2014	NUS	2014_VA_BLA	54	NE08499	32.3	13.1	2.4
2014	NUS	2014_VA_BLA	55	NE10478	34.4	7.7	0.5
2014	NUS	2014_VA_BLA	56	NI12702W	47.4	11.0	4.7
2014	NUS	2014_VA_BLA	57	VA10W-140	46.6	4.5	2.0
2014	NUS	2014_VA_BLA	58	VA11W-106	52.2	4.8	2.2
2014	NUS	2014_VA_BLA	59	VA11W-301	50.6	16.4	6.1
2014	NUS	2014_VA_BLA	60	VA12FHB-53	38.7	6.5	1.5
2014	NUS	2014_IL_CHA	1	TRUMAN	12.5	NA	NA
2014	NUS	2014_IL_CHA	2	ERNIE	27.5	NA	NA
2014	NUS	2014_IL_CHA	3	FREEDOM	27.5	NA	NA
2014	NUS	2014_IL_CHA	4	PIONEER2545	45.0	NA	NA
2014	NUS	2014_IL_CHA	5	NY01016-AN	50.0	NA	NA
2014	NUS	2014_IL_CHA	6	NY01066-278	50.0	NA	NA
2014	NUS	2014_IL_CHA	7	NY99059-249	45.0	NA	NA
2014	NUS	2014_IL_CHA	8	NY99069-249	35.0	NA	NA
2014	NUS	2014_IL_CHA	9	NY99069-352	30.0	NA	NA
2014	NUS	2014_IL_CHA	10	KWS023	35.0	NA	NA
2014	NUS	2014_IL_CHA	11	KWS024	35.0	NA	NA
2014	NUS	2014_IL_CHA	12	KWS025	55.0	NA	NA
2014	NUS	2014_IL_CHA	13	KWS028	32.5	NA	NA
2014	NUS	2014_IL_CHA	14	L29230	47.5	NA	NA
2014	NUS	2014_IL_CHA	15	LCS321	32.5	NA	NA
2014	NUS	2014_IL_CHA	16	E6012	27.5	NA	NA
2014	NUS	2014_IL_CHA	17	F0036R	35.0	NA	NA
2014	NUS	2014_IL_CHA	18	F0039	45.0	NA	NA
2014	NUS	2014_IL_CHA	19	F1014	20.0	NA	NA
2014	NUS	2014_IL_CHA	20	OH07-263-3	30.0	NA	NA
2014	NUS	2014_IL_CHA	21	OH08-206-69	30.0	NA	NA
2014	NUS	2014_IL_CHA	22	OH08-269-58	25.0	NA	NA
2014	NUS	2014_IL_CHA	23	0570A1-2-32-5-1-4	20.0	NA	NA
2014	NUS	2014_IL_CHA	24	0762A1-2-8	10.0	NA	NA
2014	NUS	2014_IL_CHA	25	08334A1-31	35.0	NA	NA
2014	NUS	2014_IL_CHA	26	10641B1-9-11-7	20.0	NA	NA
2014	NUS	2014_IL_CHA	27	B08-91993	42.5	NA	NA
2014	NUS	2014_IL_CHA	28	B09-900256	30.0	NA	NA
2014	NUS	2014_IL_CHA	29	M09L-9547	17.5	NA	NA
2014	NUS	2014_IL_CHA	30	M10-1100	20.0	NA	NA
2014	NUS	2014_IL_CHA	31	M11-1027	32.5	NA	NA

2014	NUS	2014_IL_CHA	32	M11-2298	15.0	NA	NA
2014	NUS	2014_IL_CHA	33	GL133	42.5	NA	NA
2014	NUS	2014_IL_CHA	34	GL164	22.5	NA	NA
2014	NUS	2014_IL_CHA	35	UGRCC2-78	50.0	NA	NA
2014	NUS	2014_IL_CHA	36	UGRCC5-116	32.5	NA	NA
2014	NUS	2014_IL_CHA	37	IL09-24328	12.5	NA	NA
2014	NUS	2014_IL_CHA	38	IL09-3264	25.0	NA	NA
2014	NUS	2014_IL_CHA	39	IL10-19464	15.0	NA	NA
2014	NUS	2014_IL_CHA	40	IL10-6855	20.0	NA	NA
2014	NUS	2014_IL_CHA	41	KY05C-1020-4-6-5	5.0	NA	NA
2014	NUS	2014_IL_CHA	42	KY05C-1105-43-6-1	10.0	NA	NA
2014	NUS	2014_IL_CHA	43	KY06C-3003-43-13-3	20.0	NA	NA
2014	NUS	2014_IL_CHA	44	KY204604	60.0	NA	NA
2014	NUS	2014_IL_CHA	45	MD08-22-22-13-4	4.0	NA	NA
2014	NUS	2014_IL_CHA	46	MD08-22-22-13-10	4.0	NA	NA
2014	NUS	2014_IL_CHA	47	MD09W272-8-4-13-3	30.0	NA	NA
2014	NUS	2014_IL_CHA	48	MDC07026-12-28	20.0	NA	NA
2014	NUS	2014_IL_CHA	49	MO120194	7.5	NA	NA
2014	NUS	2014_IL_CHA	50	MO120452	32.5	NA	NA
2014	NUS	2014_IL_CHA	51	MO120794	10.0	NA	NA
2014	NUS	2014_IL_CHA	52	MO121183	7.5	NA	NA
2014	NUS	2014_IL_CHA	53	NE06545	12.5	NA	NA
2014	NUS	2014_IL_CHA	54	NE08499	25.0	NA	NA
2014	NUS	2014_IL_CHA	55	NE10478	25.0	NA	NA
2014	NUS	2014_IL_CHA	56	NI12702W	30.0	NA	NA
2014	NUS	2014_IL_CHA	57	VA10W-140	45.0	NA	NA
2014	NUS	2014_IL_CHA	58	VA11W-106	12.5	NA	NA
2014	NUS	2014_IL_CHA	59	VA11W-301	42.5	NA	NA
2014	NUS	2014_IL_CHA	60	VA12FHB-53	12.5	NA	NA
2014	NUS	2014_MO_COL	1	TRUMAN	10.5	NA	NA
2014	NUS	2014_MO_COL	2	ERNIE	21.7	NA	NA
2014	NUS	2014_MO_COL	3	FREEDOM	25.9	NA	NA
2014	NUS	2014_MO_COL	4	PIONEER2545	65.6	NA	NA
2014	NUS	2014_MO_COL	5	NY01016-AN	14.1	NA	NA
2014	NUS	2014_MO_COL	6	NY01066-278	16.2	NA	NA
2014	NUS	2014_MO_COL	7	NY99059-249	16.2	NA	NA
2014	NUS	2014_MO_COL	8	NY99069-249	67.6	NA	NA
2014	NUS	2014_MO_COL	9	NY99069-352	11.4	NA	NA
2014	NUS	2014_MO_COL	10	KWS023	32.1	NA	NA
2014	NUS	2014_MO_COL	11	KWS024	37.7	NA	NA

2014	NUS	2014_MO_COL	12	KWS025	21.0	NA	NA
2014	NUS	2014_MO_COL	13	KWS028	24.8	NA	NA
2014	NUS	2014_MO_COL	14	L29230	36.3	NA	NA
2014	NUS	2014_MO_COL	15	LCS321	44.6	NA	NA
2014	NUS	2014_MO_COL	16	E6012	17.0	NA	NA
2014	NUS	2014_MO_COL	17	F0036R	11.7	NA	NA
2014	NUS	2014_MO_COL	18	F0039	58.5	NA	NA
2014	NUS	2014_MO_COL	19	F1014	40.5	NA	NA
2014	NUS	2014_MO_COL	20	OH07-263-3	52.6	NA	NA
2014	NUS	2014_MO_COL	21	OH08-206-69	31.6	NA	NA
2014	NUS	2014_MO_COL	22	OH08-269-58	48.6	NA	NA
2014	NUS	2014_MO_COL	23	0570A1-2-32-5-1-4	12.8	NA	NA
2014	NUS	2014_MO_COL	24	0762A1-2-8	10.3	NA	NA
2014	NUS	2014_MO_COL	25	08334A1-31	24.5	NA	NA
2014	NUS	2014_MO_COL	26	10641B1-9-11-7	23.4	NA	NA
2014	NUS	2014_MO_COL	27	B08-91993	47.0	NA	NA
2014	NUS	2014_MO_COL	28	B09-900256	8.4	NA	NA
2014	NUS	2014_MO_COL	29	M09L-9547	37.4	NA	NA
2014	NUS	2014_MO_COL	30	M10-1100	40.4	NA	NA
2014	NUS	2014_MO_COL	31	M11-1027	25.7	NA	NA
2014	NUS	2014_MO_COL	32	M11-2298	32.6	NA	NA
2014	NUS	2014_MO_COL	33	GL133	38.8	NA	NA
2014	NUS	2014_MO_COL	34	GL164	60.8	NA	NA
2014	NUS	2014_MO_COL	35	UGRCC2-78	62.4	NA	NA
2014	NUS	2014_MO_COL	36	UGRCC5-116	37.0	NA	NA
2014	NUS	2014_MO_COL	37	IL09-24328	20.5	NA	NA
2014	NUS	2014_MO_COL	38	IL09-3264	17.2	NA	NA
2014	NUS	2014_MO_COL	39	IL10-19464	26.6	NA	NA

2014	NUS	2014_MO_COL	40	IL10-6855	10.0	NA	NA
2014	NUS	2014_MO_COL	41	KY05C-1020-4-6-5	18.2	NA	NA
2014	NUS	2014_MO_COL	42	KY05C-1105-43-6-1	23.6	NA	NA
2014	NUS	2014_MO_COL	43	KY06C-3003-43-13-3	57.1	NA	NA
2014	NUS	2014_MO_COL	44	KY204604	31.6	NA	NA
2014	NUS	2014_MO_COL	45	MD08-22-22-13-4	31.3	NA	NA
2014	NUS	2014_MO_COL	46	MD08-22-22-13-10	11.0	NA	NA
2014	NUS	2014_MO_COL	47	MD09W272-8-4-13-3	15.9	NA	NA
2014	NUS	2014_MO_COL	48	MDC07026-12-28	14.7	NA	NA
2014	NUS	2014_MO_COL	49	MO120194	18.8	NA	NA
2014	NUS	2014_MO_COL	50	MO120452	31.8	NA	NA
2014	NUS	2014_MO_COL	51	MO120794	27.6	NA	NA
2014	NUS	2014_MO_COL	52	MO121183	18.3	NA	NA
2014	NUS	2014_MO_COL	53	NE06545	51.0	NA	NA
2014	NUS	2014_MO_COL	54	NE08499	49.8	NA	NA
2014	NUS	2014_MO_COL	55	NE10478	35.4	NA	NA
2014	NUS	2014_MO_COL	56	NI12702W	15.6	NA	NA
2014	NUS	2014_MO_COL	57	VA10W-140	34.2	NA	NA
2014	NUS	2014_MO_COL	58	VA11W-106	25.0	NA	NA
2014	NUS	2014_MO_COL	59	VA11W-301	28.1	NA	NA
2014	NUS	2014_MO_COL	60	VA12FHB-53	27.5	NA	NA
2014	NUS	2014_MI_ELA	1	TRUMAN	33.1	26.1	3.0
2014	NUS	2014_MI_ELA	2	ERNIE	59.5	30.0	9.0
2014	NUS	2014_MI_ELA	3	FREEDOM	62.0	38.0	8.1
2014	NUS	2014_MI_ELA	4	PIONEER2545	51.2	26.3	14.8
2014	NUS	2014_MI_ELA	5	NY01016-AN	56.9	43.5	15.1
2014	NUS	2014_MI_ELA	6	NY01066-278	66.1	37.1	18.4
2014	NUS	2014_MI_ELA	7	NY99059-249	52.9	21.8	13.6
2014	NUS	2014_MI_ELA	8	NY99069-249	63.6	21.0	7.9
2014	NUS	2014_MI_ELA	9	NY99069-352	59.4	32.5	10.5
2014	NUS	2014_MI_ELA	10	KWS023	47.9	30.6	6.4
2014	NUS	2014_MI_ELA	11	KWS024	50.5	31.8	6.0
2014	NUS	2014_MI_ELA	12	KWS025	55.2	23.1	6.2

2014	NUS	2014_MI_ELA	13	KWS028	62.3	23.6	8.2
2014	NUS	2014_MI_ELA	14	L29230	58.1	27.0	8.5
2014	NUS	2014_MI_ELA	15	LCS321	54.3	40.5	5.9
2014	NUS	2014_MI_ELA	16	E6012	55.8	27.5	9.5
2014	NUS	2014_MI_ELA	17	F0036R	49.8	34.3	6.4
2014	NUS	2014_MI_ELA	18	F0039	72.6	33.5	14.3
2014	NUS	2014_MI_ELA	19	F1014	35.5	19.2	6.9
2014	NUS	2014_MI_ELA	20	OH07-263-3	57.3	21.6	9.9
2014	NUS	2014_MI_ELA	21	OH08-206-69	44.3	20.0	9.0
2014	NUS	2014_MI_ELA	22	OH08-269-58	67.3	52.5	8.2
2014	NUS	2014_MI_ELA	23	0570A1-2-32-5-1-4	33.6	31.0	7.6
2014	NUS	2014_MI_ELA	24	0762A1-2-8	28.4	13.7	2.9
2014	NUS	2014_MI_ELA	25	08334A1-31	41.9	22.6	2.8
2014	NUS	2014_MI_ELA	26	10641B1-9-11-7	28.5	34.0	5.6
2014	NUS	2014_MI_ELA	27	B08-91993	53.5	31.2	15.2
2014	NUS	2014_MI_ELA	28	B09-900256	64.4	23.5	13.1
2014	NUS	2014_MI_ELA	29	M09L-9547	58.3	39.0	5.3
2014	NUS	2014_MI_ELA	30	M10-1100	52.4	17.4	6.6
2014	NUS	2014_MI_ELA	31	M11-1027	26.7	25.1	2.3
2014	NUS	2014_MI_ELA	32	M11-2298	44.1	18.3	2.9
2014	NUS	2014_MI_ELA	33	GL133	55.1	29.3	5.8
2014	NUS	2014_MI_ELA	34	GL164	68.0	20.2	7.7
2014	NUS	2014_MI_ELA	35	UGRCC2-78	57.0	42.0	9.5
2014	NUS	2014_MI_ELA	36	UGRCC5-116	58.1	32.0	3.2
2014	NUS	2014_MI_ELA	37	IL09-24328	51.3	22.9	3.9
2014	NUS	2014_MI_ELA	38	IL09-3264	43.8	20.5	2.6
2014	NUS	2014_MI_ELA	39	IL10-19464	35.3	28.6	5.8
2014	NUS	2014_MI_ELA	40	IL10-6855	36.1	23.3	5.3
2014	NUS	2014_MI_ELA	41	KY05C-1020-4-6-5	30.4	41.9	7.2
2014	NUS	2014_MI_ELA	42	KY05C-1105-43-6-1	50.9	17.8	1.9
2014	NUS	2014_MI_ELA	43	KY06C-3003-43-13-3	56.9	23.1	3.7
2014	NUS	2014_MI_ELA	44	KY204604	45.4	44.4	8.2
2014	NUS	2014_MI_ELA	45	MD08-22-22-13-4	18.4	15.0	1.5
2014	NUS	2014_MI_ELA	46	MD08-22-22-13-10	33.3	15.5	5.1
2014	NUS	2014_MI_ELA	47	MD09W272-8-4-13-3	44.6	26.2	5.2
2014	NUS	2014_MI_ELA	48	MDC07026-12-28	50.8	15.2	3.3
2014	NUS	2014_MI_ELA	49	MO120194	57.6	19.3	2.8
2014	NUS	2014_MI_ELA	50	MO120452	25.4	22.8	3.5
2014	NUS	2014_MI_ELA	51	MO120794	40.1	21.2	4.3
2014	NUS	2014_MI_ELA	52	MO121183	40.9	25.3	4.5
2014	NUS	2014_MI_ELA	53	NE06545	59.4	33.1	8.0
2014	NUS	2014_MI_ELA	54	NE08499	61.8	26.9	16.6
2014	NUS	2014_MI_ELA	55	NE10478	47.8	21.0	5.8
2014	NUS	2014_MI_ELA	56	NI12702W	53.0	21.2	11.7
2014	NUS	2014_MI_ELA	57	VA10W-140	67.1	30.8	8.7
2014	NUS	2014_MI_ELA	58	VA11W-106	54.6	17.4	7.6
2014	NUS	2014_MI_ELA	59	VA11W-301	56.5	37.5	8.2
2014	NUS	2014_MI_ELA	60	VA12FHB-53	45.2	21.2	5.8
2014	NUS	2014_IN_HIG	1	TRUMAN	35.0	96.1	NA

2014	NUS	2014_IN_HIG	2	ERNIE	55.0	76.2	NA
2014	NUS	2014_IN_HIG	3	FREEDOM	60.0	91.3	NA
2014	NUS	2014_IN_HIG	4	PIONEER2545	70.0	77.0	NA
2014	NUS	2014_IN_HIG	5	NY01016-AN	40.0	100.0	NA
2014	NUS	2014_IN_HIG	6	NY01066-278	65.0	79.7	NA
2014	NUS	2014_IN_HIG	7	NY99059-249	75.0	90.3	NA
2014	NUS	2014_IN_HIG	8	NY99069-249	55.0	82.7	NA
2014	NUS	2014_IN_HIG	9	NY99069-352	70.0	100.0	NA
2014	NUS	2014_IN_HIG	10	KWS023	85.0	91.2	NA
2014	NUS	2014_IN_HIG	11	KWS024	80.0	100.0	NA
2014	NUS	2014_IN_HIG	12	KWS025	45.0	100.0	NA
2014	NUS	2014_IN_HIG	13	KWS028	75.0	60.3	NA
2014	NUS	2014_IN_HIG	14	L29230	80.0	92.9	NA
2014	NUS	2014_IN_HIG	15	LCS321	80.0	74.3	NA
2014	NUS	2014_IN_HIG	16	E6012	80.0	100.0	NA
2014	NUS	2014_IN_HIG	17	F0036R	60.0	88.7	NA
2014	NUS	2014_IN_HIG	18	F0039	75.0	100.0	NA
2014	NUS	2014_IN_HIG	19	F1014	70.0	100.0	NA
2014	NUS	2014_IN_HIG	20	OH07-263-3	55.0	100.0	NA
2014	NUS	2014_IN_HIG	21	OH08-206-69	70.0	94.7	NA
2014	NUS	2014_IN_HIG	22	OH08-269-58	90.0	94.4	NA
2014	NUS	2014_IN_HIG	23	0570A1-2-32-5-1-4	90.0	100.0	NA
2014	NUS	2014_IN_HIG	24	0762A1-2-8	60.0	100.0	NA
2014	NUS	2014_IN_HIG	25	08334A1-31	75.0	99.3	NA
2014	NUS	2014_IN_HIG	26	10641B1-9-11-7	80.0	94.4	NA
2014	NUS	2014_IN_HIG	27	B08-91993	75.0	100.0	NA
2014	NUS	2014_IN_HIG	28	B09-900256	80.0	100.0	NA
2014	NUS	2014_IN_HIG	29	M09L-9547	75.0	100.0	NA
2014	NUS	2014_IN_HIG	30	M10-1100	75.0	87.1	NA
2014	NUS	2014_IN_HIG	31	M11-1027	80.0	90.8	NA
2014	NUS	2014_IN_HIG	32	M11-2298	35.0	80.4	NA
2014	NUS	2014_IN_HIG	33	GL133	70.0	96.1	NA
2014	NUS	2014_IN_HIG	34	GL164	50.0	54.5	NA
2014	NUS	2014_IN_HIG	35	UGRCC2-78	60.0	100.0	NA
2014	NUS	2014_IN_HIG	36	UGRCC5-116	55.0	76.4	NA
2014	NUS	2014_IN_HIG	37	IL09-24328	40.0	82.1	NA
2014	NUS	2014_IN_HIG	38	IL09-3264	50.0	62.1	NA
2014	NUS	2014_IN_HIG	39	IL10-19464	80.0	61.9	NA
2014	NUS	2014_IN_HIG	40	IL10-6855	75.0	40.8	NA
2014	NUS	2014_IN_HIG	41	KY05C-1020-4-6-5	65.0	84.3	NA
2014	NUS	2014_IN_HIG	42	KY05C-1105-43-6-1	75.0	75.1	NA
2014	NUS	2014_IN_HIG	43	KY06C-3003-43-13-3	35.0	100.0	NA
2014	NUS	2014_IN_HIG	44	KY204604	75.0	90.5	NA
2014	NUS	2014_IN_HIG	45	MD08-22-22-13-4	20.0	53.1	NA
2014	NUS	2014_IN_HIG	46	MD08-22-22-13-10	30.0	72.7	NA
2014	NUS	2014_IN_HIG	47	MD09W272-8-4-13-3	75.0	88.3	NA
2014	NUS	2014_IN_HIG	48	MDC07026-12-28	70.0	49.1	NA
2014	NUS	2014_IN_HIG	49	MO120194	65.0	66.6	NA
2014	NUS	2014_IN_HIG	50	MO120452	70.0	62.4	NA

2014	NUS	2014_IN_HIG	51	MO120794	55.0	100.0	NA
2014	NUS	2014_IN_HIG	52	MO121183	75.0	94.8	NA
2014	NUS	2014_IN_HIG	53	NE06545	80.0	89.3	NA
2014	NUS	2014_IN_HIG	54	NE08499	85.0	84.2	NA
2014	NUS	2014_IN_HIG	55	NE10478	75.0	100.0	NA
2014	NUS	2014_IN_HIG	56	NI12702W	85.0	75.0	NA
2014	NUS	2014_IN_HIG	57	VA10W-140	70.0	97.1	NA
2014	NUS	2014_IN_HIG	58	VA11W-106	80.0	95.1	NA
2014	NUS	2014_IN_HIG	59	VA11W-301	80.0	100.0	NA
2014	NUS	2014_IN_HIG	60	VA12FHB-53	60.0	90.4	NA
2014	NUS	2014_NY_ITH	1	TRUMAN	12.4	NA	NA
2014	NUS	2014_NY_ITH	2	ERNIE	12.2	NA	NA
2014	NUS	2014_NY_ITH	3	FREEDOM	16.0	NA	NA
2014	NUS	2014_NY_ITH	4	PIONEER2545	14.8	NA	NA
2014	NUS	2014_NY_ITH	5	NY01016-AN	8.6	NA	NA
2014	NUS	2014_NY_ITH	6	NY01066-278	28.0	NA	NA
2014	NUS	2014_NY_ITH	7	NY99059-249	12.6	NA	NA
2014	NUS	2014_NY_ITH	8	NY99069-249	14.2	NA	NA
2014	NUS	2014_NY_ITH	9	NY99069-352	15.6	NA	NA
2014	NUS	2014_NY_ITH	10	KWS023	16.6	NA	NA
2014	NUS	2014_NY_ITH	11	KWS024	15.0	NA	NA
2014	NUS	2014_NY_ITH	12	KWS025	30.4	NA	NA
2014	NUS	2014_NY_ITH	13	KWS028	10.4	NA	NA
2014	NUS	2014_NY_ITH	14	L29230	24.0	NA	NA
2014	NUS	2014_NY_ITH	15	LCS321	8.6	NA	NA
2014	NUS	2014_NY_ITH	16	E6012	11.4	NA	NA
2014	NUS	2014_NY_ITH	17	F0036R	14.8	NA	NA
2014	NUS	2014_NY_ITH	18	F0039	55.5	NA	NA
2014	NUS	2014_NY_ITH	19	F1014	16.8	NA	NA
2014	NUS	2014_NY_ITH	20	OH07-263-3	15.8	NA	NA
2014	NUS	2014_NY_ITH	21	OH08-206-69	16.4	NA	NA
2014	NUS	2014_NY_ITH	22	OH08-269-58	11.2	NA	NA
2014	NUS	2014_NY_ITH	23	0570A1-2-32-5-1-4	15.6	NA	NA
2014	NUS	2014_NY_ITH	24	0762A1-2-8	9.8	NA	NA
2014	NUS	2014_NY_ITH	25	08334A1-31	14.4	NA	NA
2014	NUS	2014_NY_ITH	26	10641B1-9-11-7	8.6	NA	NA
2014	NUS	2014_NY_ITH	27	B08-91993	13.2	NA	NA
2014	NUS	2014_NY_ITH	28	B09-900256	17.2	NA	NA
2014	NUS	2014_NY_ITH	29	M09L-9547	7.2	NA	NA
2014	NUS	2014_NY_ITH	30	M10-1100	17.4	NA	NA
2014	NUS	2014_NY_ITH	31	M11-1027	11.8	NA	NA
2014	NUS	2014_NY_ITH	32	M11-2298	13.6	NA	NA
2014	NUS	2014_NY_ITH	33	GL133	18.4	NA	NA
2014	NUS	2014_NY_ITH	34	GL164	16.0	NA	NA
2014	NUS	2014_NY_ITH	35	UGRCC2-78	20.2	NA	NA
2014	NUS	2014_NY_ITH	36	UGRCC5-116	12.8	NA	NA
2014	NUS	2014_NY_ITH	37	IL09-24328	12.0	NA	NA
2014	NUS	2014_NY_ITH	38	IL09-3264	16.0	NA	NA
2014	NUS	2014_NY_ITH	39	IL10-19464	9.2	NA	NA

2014	NUS	2014_NY_ITH	40	IL10-6855	7.6	NA	NA
2014	NUS	2014_NY_ITH	41	KY05C-1020-4-6-5	9.2	NA	NA
2014	NUS	2014_NY_ITH	42	KY05C-1105-43-6-1	10.2	NA	NA
2014	NUS	2014_NY_ITH	43	KY06C-3003-43-13-3	7.0	NA	NA
2014	NUS	2014_NY_ITH	44	KY204604	25.2	NA	NA
2014	NUS	2014_NY_ITH	45	MD08-22-22-13-4	8.6	NA	NA
2014	NUS	2014_NY_ITH	46	MD08-22-22-13-10	9.0	NA	NA
2014	NUS	2014_NY_ITH	47	MD09W272-8-4-13-3	9.6	NA	NA
2014	NUS	2014_NY_ITH	48	MDC07026-12-28	6.8	NA	NA
2014	NUS	2014_NY_ITH	49	MO120194	10.2	NA	NA
2014	NUS	2014_NY_ITH	50	MO120452	11.4	NA	NA
2014	NUS	2014_NY_ITH	51	MO120794	8.0	NA	NA
2014	NUS	2014_NY_ITH	52	MO121183	15.0	NA	NA
2014	NUS	2014_NY_ITH	53	NE06545	13.8	NA	NA
2014	NUS	2014_NY_ITH	54	NE08499	10.0	NA	NA
2014	NUS	2014_NY_ITH	55	NE10478	16.4	NA	NA
2014	NUS	2014_NY_ITH	56	NI12702W	27.6	NA	NA
2014	NUS	2014_NY_ITH	57	VA10W-140	15.4	NA	NA
2014	NUS	2014_NY_ITH	58	VA11W-106	14.2	NA	NA
2014	NUS	2014_NY_ITH	59	VA11W-301	23.0	NA	NA
2014	NUS	2014_NY_ITH	60	VA12FHB-53	9.6	NA	NA
2014	NUS	2014_KY_LEX	1	TRUMAN	38.2	11.5	16.1
2014	NUS	2014_KY_LEX	2	ERNIE	29.0	27.6	14.7
2014	NUS	2014_KY_LEX	3	FREEDOM	45.9	33.2	16.5
2014	NUS	2014_KY_LEX	4	PIONEER2545	57.8	45.9	16.9
2014	NUS	2014_KY_LEX	5	NY01016-AN	41.2	21.9	15.7
2014	NUS	2014_KY_LEX	6	NY01066-278	61.0	41.2	14.9
2014	NUS	2014_KY_LEX	7	NY99059-249	40.0	20.3	13.8
2014	NUS	2014_KY_LEX	8	NY99069-249	43.2	31.7	12.7
2014	NUS	2014_KY_LEX	9	NY99069-352	50.1	38.8	22.7
2014	NUS	2014_KY_LEX	10	KWS023	47.8	27.4	11.6
2014	NUS	2014_KY_LEX	11	KWS024	26.3	27.2	12.5
2014	NUS	2014_KY_LEX	12	KWS025	46.7	32.4	14.2
2014	NUS	2014_KY_LEX	13	KWS028	46.7	29.1	13.9
2014	NUS	2014_KY_LEX	14	L29230	51.6	36.7	12.8
2014	NUS	2014_KY_LEX	15	LCS321	41.7	27.9	21.8
2014	NUS	2014_KY_LEX	16	E6012	51.2	28.3	16.3
2014	NUS	2014_KY_LEX	17	F0036R	39.9	34.6	13.1
2014	NUS	2014_KY_LEX	18	F0039	65.5	43.4	19.8
2014	NUS	2014_KY_LEX	19	F1014	53.9	28.2	17.9
2014	NUS	2014_KY_LEX	20	OH07-263-3	34.5	22.6	15.1
2014	NUS	2014_KY_LEX	21	OH08-206-69	35.1	30.5	11.6
2014	NUS	2014_KY_LEX	22	OH08-269-58	49.1	33.2	16.1
2014	NUS	2014_KY_LEX	23	0570A1-2-32-5-1-4	34.0	21.1	21.6
2014	NUS	2014_KY_LEX	24	0762A1-2-8	18.2	22.3	7.7
2014	NUS	2014_KY_LEX	25	08334A1-31	26.2	26.0	12.2
2014	NUS	2014_KY_LEX	26	10641B1-9-11-7	22.4	18.2	13.4
2014	NUS	2014_KY_LEX	27	B08-91993	37.8	21.8	11.3
2014	NUS	2014_KY_LEX	28	B09-900256	23.1	33.7	17.3

2014	NUS	2014_KY_LEX	29	M09L-9547	31.1	26.3	15.7
2014	NUS	2014_KY_LEX	30	M10-1100	37.8	28.9	10.8
2014	NUS	2014_KY_LEX	31	M11-1027	28.9	27.7	11.9
2014	NUS	2014_KY_LEX	32	M11-2298	21.7	23.8	12.4
2014	NUS	2014_KY_LEX	33	GL133	40.8	36.6	15.9
2014	NUS	2014_KY_LEX	34	GL164	29.2	29.2	13.4
2014	NUS	2014_KY_LEX	35	UGRCC2-78	44.9	40.5	15.5
2014	NUS	2014_KY_LEX	36	UGRCC5-116	39.8	29.1	9.5
2014	NUS	2014_KY_LEX	37	IL09-24328	22.9	14.5	7.5
2014	NUS	2014_KY_LEX	38	IL09-3264	14.3	14.6	6.2
2014	NUS	2014_KY_LEX	39	IL10-19464	26.1	15.7	8.1
2014	NUS	2014_KY_LEX	40	IL10-6855	17.2	8.6	6.3
2014	NUS	2014_KY_LEX	41	KY05C-1020-4-6-5	25.6	22.7	15.4
2014	NUS	2014_KY_LEX	42	KY05C-1105-43-6-1	30.1	27.7	12.2
2014	NUS	2014_KY_LEX	43	KY06C-3003-43-13-3	28.9	19.9	12.1
2014	NUS	2014_KY_LEX	44	KY204604	48.3	34.3	17.2
2014	NUS	2014_KY_LEX	45	MD08-22-22-13-4	19.6	9.8	6.7
2014	NUS	2014_KY_LEX	46	MD08-22-22-13-10	20.7	13.0	6.8
2014	NUS	2014_KY_LEX	47	MD09W272-8-4-13-3	39.4	22.9	9.6
2014	NUS	2014_KY_LEX	48	MDC07026-12-28	20.1	23.8	6.3
2014	NUS	2014_KY_LEX	49	MO120194	32.8	23.3	13.8
2014	NUS	2014_KY_LEX	50	MO120452	37.6	14.0	11.0
2014	NUS	2014_KY_LEX	51	MO120794	35.0	27.4	13.4
2014	NUS	2014_KY_LEX	52	MO121183	33.8	27.8	7.3
2014	NUS	2014_KY_LEX	53	NE06545	46.9	36.4	11.0
2014	NUS	2014_KY_LEX	54	NE08499	34.7	29.2	13.6
2014	NUS	2014_KY_LEX	55	NE10478	39.4	44.4	11.6
2014	NUS	2014_KY_LEX	56	NI12702W	40.9	32.7	19.3
2014	NUS	2014_KY_LEX	57	VA10W-140	48.8	22.8	9.3
2014	NUS	2014_KY_LEX	58	VA11W-106	44.0	26.3	13.1
2014	NUS	2014_KY_LEX	59	VA11W-301	31.5	31.0	12.1
2014	NUS	2014_KY_LEX	60	VA12FHB-53	32.6	28.4	10.1
2014	NUS	2014_NE_MEA	1	TRUMAN	0.0	17.0	NA
2014	NUS	2014_NE_MEA	2	ERNIE	14.0	17.0	NA
2014	NUS	2014_NE_MEA	3	FREEDOM	NA	NA	NA
2014	NUS	2014_NE_MEA	4	PIONEER2545	16.3	90.0	NA
2014	NUS	2014_NE_MEA	5	NY01016-AN	0.0	100.0	NA
2014	NUS	2014_NE_MEA	6	NY01066-278	7.0	NA	NA
2014	NUS	2014_NE_MEA	7	NY99059-249	0.0	8.0	NA
2014	NUS	2014_NE_MEA	8	NY99069-249	8.0	68.0	NA
2014	NUS	2014_NE_MEA	9	NY99069-352	0.0	NA	NA
2014	NUS	2014_NE_MEA	10	KWS023	11.1	28.0	NA
2014	NUS	2014_NE_MEA	11	KWS024	NA	21.0	NA
2014	NUS	2014_NE_MEA	12	KWS025	12.5	11.0	NA
2014	NUS	2014_NE_MEA	13	KWS028	6.7	69.0	NA
2014	NUS	2014_NE_MEA	14	L29230	10.8	30.0	NA
2014	NUS	2014_NE_MEA	15	LCS321	3.1	32.0	NA
2014	NUS	2014_NE_MEA	16	E6012	8.7	36.0	NA
2014	NUS	2014_NE_MEA	17	F0036R	5.0	28.0	NA

2014	NUS	2014_NE_MEA	18	F0039	NA	NA	NA
2014	NUS	2014_NE_MEA	19	F1014	NA	NA	NA
2014	NUS	2014_NE_MEA	20	OH07-263-3	20.3	41.0	NA
2014	NUS	2014_NE_MEA	21	OH08-206-69	11.1	92.0	NA
2014	NUS	2014_NE_MEA	22	OH08-269-58	23.1	40.0	NA
2014	NUS	2014_NE_MEA	23	0570A1-2-32-5-1-4	NA	NA	NA
2014	NUS	2014_NE_MEA	24	0762A1-2-8	7.5	NA	NA
2014	NUS	2014_NE_MEA	25	08334A1-31	12.0	NA	NA
2014	NUS	2014_NE_MEA	26	10641B1-9-11-7	NA	NA	NA
2014	NUS	2014_NE_MEA	27	B08-91993	10.0	23.0	NA
2014	NUS	2014_NE_MEA	28	B09-900256	15.0	20.0	NA
2014	NUS	2014_NE_MEA	29	M09L-9547	9.1	8.0	NA
2014	NUS	2014_NE_MEA	30	M10-1100	11.7	44.0	NA
2014	NUS	2014_NE_MEA	31	M11-1027	9.7	5.0	NA
2014	NUS	2014_NE_MEA	32	M11-2298	6.3	3.0	NA
2014	NUS	2014_NE_MEA	33	GL133	33.3	4.0	NA
2014	NUS	2014_NE_MEA	34	GL164	0.0	2.0	NA
2014	NUS	2014_NE_MEA	35	UGRCC2-78	12.5	4.0	NA
2014	NUS	2014_NE_MEA	36	UGRCC5-116	13.4	3.0	NA
2014	NUS	2014_NE_MEA	37	IL09-24328	17.1	5.0	NA
2014	NUS	2014_NE_MEA	38	IL09-3264	21.8	3.0	NA
2014	NUS	2014_NE_MEA	39	IL10-19464	15.9	3.0	NA
2014	NUS	2014_NE_MEA	40	IL10-6855	20.9	6.0	NA
2014	NUS	2014_NE_MEA	41	KY05C-1020-4-6-5	6.3	6.0	NA
2014	NUS	2014_NE_MEA	42	KY05C-1105-43-6-1	15.0	20.0	NA
2014	NUS	2014_NE_MEA	43	KY06C-3003-43-13-3	3.3	16.0	NA
2014	NUS	2014_NE_MEA	44	KY204604	9.7	11.0	NA
2014	NUS	2014_NE_MEA	45	MD08-22-22-13-4	17.6	0.0	NA
2014	NUS	2014_NE_MEA	46	MD08-22-22-13-10	0.0	4.0	NA
2014	NUS	2014_NE_MEA	47	MD09W272-8-4-13-3	8.0	NA	NA
2014	NUS	2014_NE_MEA	48	MDC07026-12-28	30.3	5.0	NA
2014	NUS	2014_NE_MEA	49	MO120194	18.8	49.0	NA
2014	NUS	2014_NE_MEA	50	MO120452	23.8	7.0	NA
2014	NUS	2014_NE_MEA	51	MO120794	2.5	6.0	NA
2014	NUS	2014_NE_MEA	52	MO121183	24.7	14.0	NA
2014	NUS	2014_NE_MEA	53	NE06545	20.0	14.0	NA
2014	NUS	2014_NE_MEA	54	NE08499	13.1	6.0	NA
2014	NUS	2014_NE_MEA	55	NE10478	7.9	55.0	NA
2014	NUS	2014_NE_MEA	56	NI12702W	14.5	5.0	NA
2014	NUS	2014_NE_MEA	57	VA10W-140	2.5	NA	NA
2014	NUS	2014_NE_MEA	58	VA11W-106	5.8	NA	NA
2014	NUS	2014_NE_MEA	59	VA11W-301	15.0	6.0	NA
2014	NUS	2014_NE_MEA	60	VA12FHB-53	5.0	20.0	NA
2014	NUS	2014_ON_RID	1	TRUMAN	6.7	NA	NA
2014	NUS	2014_ON_RID	2	ERNIE	30.0	NA	NA
2014	NUS	2014_ON_RID	3	FREEDOM	31.7	NA	NA
2014	NUS	2014_ON_RID	4	PIONEER2545	55.0	NA	NA
2014	NUS	2014_ON_RID	5	NY01016-AN	23.3	NA	NA
2014	NUS	2014_ON_RID	6	NY01066-278	48.3	NA	NA

2014	NUS	2014_ON_RID	7	NY99059-249	28.3	NA	NA
2014	NUS	2014_ON_RID	8	NY99069-249	30.0	NA	NA
2014	NUS	2014_ON_RID	9	NY99069-352	25.0	NA	NA
2014	NUS	2014_ON_RID	10	KWS023	23.3	NA	NA
2014	NUS	2014_ON_RID	11	KWS024	46.7	NA	NA
2014	NUS	2014_ON_RID	12	KWS025	20.0	NA	NA
2014	NUS	2014_ON_RID	13	KWS028	20.0	NA	NA
2014	NUS	2014_ON_RID	14	L29230	46.7	NA	NA
2014	NUS	2014_ON_RID	15	LCS321	21.7	NA	NA
2014	NUS	2014_ON_RID	16	E6012	36.7	NA	NA
2014	NUS	2014_ON_RID	17	F0036R	33.3	NA	NA
2014	NUS	2014_ON_RID	18	F0039	66.7	NA	NA
2014	NUS	2014_ON_RID	19	F1014	30.0	NA	NA
2014	NUS	2014_ON_RID	20	OH07-263-3	35.0	NA	NA
2014	NUS	2014_ON_RID	21	OH08-206-69	33.3	NA	NA
2014	NUS	2014_ON_RID	22	OH08-269-58	60.0	NA	NA
2014	NUS	2014_ON_RID	23	0570A1-2-32-5-1-4	10.0	NA	NA
2014	NUS	2014_ON_RID	24	0762A1-2-8	23.3	NA	NA
2014	NUS	2014_ON_RID	25	08334A1-31	33.3	NA	NA
2014	NUS	2014_ON_RID	26	10641B1-9-11-7	25.0	NA	NA
2014	NUS	2014_ON_RID	27	B08-91993	38.3	NA	NA
2014	NUS	2014_ON_RID	28	B09-900256	48.3	NA	NA
2014	NUS	2014_ON_RID	29	M09L-9547	20.0	NA	NA
2014	NUS	2014_ON_RID	30	M10-1100	20.0	NA	NA
2014	NUS	2014_ON_RID	31	M11-1027	10.0	NA	NA
2014	NUS	2014_ON_RID	32	M11-2298	38.3	NA	NA
2014	NUS	2014_ON_RID	33	GL133	73.3	NA	NA
2014	NUS	2014_ON_RID	34	GL164	43.3	NA	NA
2014	NUS	2014_ON_RID	35	UGRCC2-78	20.0	NA	NA
2014	NUS	2014_ON_RID	36	UGRCC5-116	17.5	NA	NA
2014	NUS	2014_ON_RID	37	IL09-24328	18.3	NA	NA
2014	NUS	2014_ON_RID	38	IL09-3264	20.0	NA	NA
2014	NUS	2014_ON_RID	39	IL10-19464	18.3	NA	NA
2014	NUS	2014_ON_RID	40	IL10-6855	36.7	NA	NA
2014	NUS	2014_ON_RID	41	KY05C-1020-4-6-5	25.0	NA	NA
2014	NUS	2014_ON_RID	42	KY05C-1105-43-6-1	28.3	NA	NA
2014	NUS	2014_ON_RID	43	KY06C-3003-43-13-3	25.0	NA	NA
2014	NUS	2014_ON_RID	44	KY204604	35.0	NA	NA
2014	NUS	2014_ON_RID	45	MD08-22-22-13-4	20.0	NA	NA
2014	NUS	2014_ON_RID	46	MD08-22-22-13-10	13.3	NA	NA
2014	NUS	2014_ON_RID	47	MD09W272-8-4-13-3	16.7	NA	NA
2014	NUS	2014_ON_RID	48	MDC07026-12-28	20.0	NA	NA
2014	NUS	2014_ON_RID	49	MO120194	16.7	NA	NA
2014	NUS	2014_ON_RID	50	MO120452	28.3	NA	NA
2014	NUS	2014_ON_RID	51	MO120794	33.3	NA	NA
2014	NUS	2014_ON_RID	52	MO121183	30.0	NA	NA
2014	NUS	2014_ON_RID	53	NE06545	15.0	NA	NA
2014	NUS	2014_ON_RID	54	NE08499	26.7	NA	NA
2014	NUS	2014_ON_RID	55	NE10478	30.0	NA	NA

2014	NUS	2014_ON_RID	56	NI12702W	23.3	NA	NA
2014	NUS	2014_ON_RID	57	VA10W-140	23.3	NA	NA
2014	NUS	2014_ON_RID	58	VA11W-106	18.3	NA	NA
2014	NUS	2014_ON_RID	59	VA11W-301	48.3	NA	NA
2014	NUS	2014_ON_RID	60	VA12FHB-53	31.7	NA	NA
2014	NUS	2014_IL_URB	1	TRUMAN	21.2	22.5	14.8
2014	NUS	2014_IL_URB	2	ERNIE	42.8	40.0	13.8
2014	NUS	2014_IL_URB	3	FREEDOM	68.6	55.0	22.3
2014	NUS	2014_IL_URB	4	PIONEER2545	78.3	57.5	44.1
2014	NUS	2014_IL_URB	5	NY01016-AN	75.5	50.0	35.2
2014	NUS	2014_IL_URB	6	NY01066-278	84.8	72.5	36.3
2014	NUS	2014_IL_URB	7	NY99059-249	70.6	30.0	15.7
2014	NUS	2014_IL_URB	8	NY99069-249	63.6	50.0	22.7
2014	NUS	2014_IL_URB	9	NY99069-352	44.9	27.5	20.6
2014	NUS	2014_IL_URB	10	KWS023	72.0	57.5	14.3
2014	NUS	2014_IL_URB	11	KWS024	43.8	42.5	12.0
2014	NUS	2014_IL_URB	12	KWS025	78.0	40.0	10.3
2014	NUS	2014_IL_URB	13	KWS028	62.9	20.0	8.6
2014	NUS	2014_IL_URB	14	L29230	48.3	37.5	11.5
2014	NUS	2014_IL_URB	15	LCS321	41.9	37.5	17.8
2014	NUS	2014_IL_URB	16	E6012	58.6	30.0	14.8
2014	NUS	2014_IL_URB	17	F0036R	66.0	52.5	32.0
2014	NUS	2014_IL_URB	18	F0039	83.7	60.0	46.5
2014	NUS	2014_IL_URB	19	F1014	20.5	50.0	40.6
2014	NUS	2014_IL_URB	20	OH07-263-3	35.9	17.5	7.1
2014	NUS	2014_IL_URB	21	OH08-206-69	50.7	65.0	12.8
2014	NUS	2014_IL_URB	22	OH08-269-58	55.2	25.0	7.9
2014	NUS	2014_IL_URB	23	0570A1-2-32-5-1-4	32.6	64.3	30.4
2014	NUS	2014_IL_URB	24	0762A1-2-8	12.8	30.0	7.5
2014	NUS	2014_IL_URB	25	08334A1-31	37.0	45.0	7.4
2014	NUS	2014_IL_URB	26	10641B1-9-11-7	19.5	72.5	20.7
2014	NUS	2014_IL_URB	27	B08-91993	40.6	25.0	9.0
2014	NUS	2014_IL_URB	28	B09-900256	68.9	67.5	18.5
2014	NUS	2014_IL_URB	29	M09L-9547	34.7	37.5	10.0
2014	NUS	2014_IL_URB	30	M10-1100	53.6	27.5	15.1
2014	NUS	2014_IL_URB	31	M11-1027	24.3	30.0	12.0
2014	NUS	2014_IL_URB	32	M11-2298	35.8	15.0	8.7
2014	NUS	2014_IL_URB	33	GL133	46.2	42.5	10.5
2014	NUS	2014_IL_URB	34	GL164	43.7	27.5	6.2
2014	NUS	2014_IL_URB	35	UGRCC2-78	67.1	27.5	9.0
2014	NUS	2014_IL_URB	36	UGRCC5-116	50.9	22.5	6.9
2014	NUS	2014_IL_URB	37	IL09-24328	27.7	12.5	4.0
2014	NUS	2014_IL_URB	38	IL09-3264	32.0	17.5	5.1
2014	NUS	2014_IL_URB	39	IL10-19464	18.6	15.0	6.5
2014	NUS	2014_IL_URB	40	IL10-6855	17.0	15.0	5.9
2014	NUS	2014_IL_URB	41	KY05C-1020-4-6-5	16.2	40.0	13.6
2014	NUS	2014_IL_URB	42	KY05C-1105-43-6-1	17.0	17.5	8.0
2014	NUS	2014_IL_URB	43	KY06C-3003-43-13-3	42.5	15.0	7.4
2014	NUS	2014_IL_URB	44	KY204604	76.5	57.5	33.8

2014	NUS	2014_IL_URB	45	MD08-22-22-13-4	10.3	12.5	3.4
2014	NUS	2014_IL_URB	46	MD08-22-22-13-10	10.3	37.5	7.4
2014	NUS	2014_IL_URB	47	MD09W272-8-4-13-3	16.5	67.5	18.6
2014	NUS	2014_IL_URB	48	MDC07026-12-28	18.2	62.5	10.1
2014	NUS	2014_IL_URB	49	MO120194	20.5	12.5	4.4
2014	NUS	2014_IL_URB	50	MO120452	18.6	25.0	11.4
2014	NUS	2014_IL_URB	51	MO120794	40.2	17.5	14.2
2014	NUS	2014_IL_URB	52	MO121183	52.6	32.5	14.4
2014	NUS	2014_IL_URB	53	NE06545	51.7	37.5	12.0
2014	NUS	2014_IL_URB	54	NE08499	49.9	35.0	10.7
2014	NUS	2014_IL_URB	55	NE10478	35.5	57.5	18.5
2014	NUS	2014_IL_URB	56	NI12702W	71.7	32.5	15.6
2014	NUS	2014_IL_URB	57	VA10W-140	62.8	45.0	11.4
2014	NUS	2014_IL_URB	58	VA11W-106	45.8	40.0	18.7
2014	NUS	2014_IL_URB	59	VA11W-301	69.9	60.0	24.0
2014	NUS	2014_IL_URB	60	VA12FHB-53	36.3	50.6	18.0
2014	NUS	2014_IN_WLA	1	TRUMAN	NA	7.5	0.7
2014	NUS	2014_IN_WLA	2	ERNIE	NA	15.5	2.8
2014	NUS	2014_IN_WLA	3	FREEDOM	NA	12.5	1.4
2014	NUS	2014_IN_WLA	4	PIONEER2545	NA	3.0	0.4
2014	NUS	2014_IN_WLA	5	NY01016-AN	NA	10.5	1.5
2014	NUS	2014_IN_WLA	6	NY01066-278	NA	10.0	1.3
2014	NUS	2014_IN_WLA	7	NY99059-249	NA	20.0	0.9
2014	NUS	2014_IN_WLA	8	NY99069-249	NA	15.5	1.6
2014	NUS	2014_IN_WLA	9	NY99069-352	NA	12.5	1.7
2014	NUS	2014_IN_WLA	10	KWS023	NA	5.0	1.5
2014	NUS	2014_IN_WLA	11	KWS024	NA	10.5	2.4
2014	NUS	2014_IN_WLA	12	KWS025	NA	20.5	1.4
2014	NUS	2014_IN_WLA	13	KWS028	NA	20.5	1.7
2014	NUS	2014_IN_WLA	14	L29230	NA	12.5	2.1
2014	NUS	2014_IN_WLA	15	LCS321	NA	10.5	1.1
2014	NUS	2014_IN_WLA	16	E6012	NA	3.0	0.4
2014	NUS	2014_IN_WLA	17	F0036R	NA	7.5	1.1
2014	NUS	2014_IN_WLA	18	F0039	NA	17.5	2.1
2014	NUS	2014_IN_WLA	19	F1014	NA	10.5	0.8
2014	NUS	2014_IN_WLA	20	OH07-263-3	NA	7.5	1.5
2014	NUS	2014_IN_WLA	21	OH08-206-69	NA	1.0	1.8
2014	NUS	2014_IN_WLA	22	OH08-269-58	NA	15.0	4.2
2014	NUS	2014_IN_WLA	23	0570A1-2-32-5-1-4	NA	20.0	3.7
2014	NUS	2014_IN_WLA	24	0762A1-2-8	NA	7.5	1.4
2014	NUS	2014_IN_WLA	25	08334A1-31	NA	20.0	2.8
2014	NUS	2014_IN_WLA	26	10641B1-9-11-7	NA	12.5	1.5
2014	NUS	2014_IN_WLA	27	B08-91993	NA	20.0	2.1
2014	NUS	2014_IN_WLA	28	B09-900256	NA	5.5	1.0
2014	NUS	2014_IN_WLA	29	M09L-9547	NA	5.0	1.0
2014	NUS	2014_IN_WLA	30	M10-1100	NA	15.5	2.1
2014	NUS	2014_IN_WLA	31	M11-1027	NA	1.0	0.6
2014	NUS	2014_IN_WLA	32	M11-2298	NA	15.5	2.2
2014	NUS	2014_IN_WLA	33	GL133	NA	3.0	1.2

2014	NUS	2014_IN_WLA	34	GL164	NA	20.5	1.8
2014	NUS	2014_IN_WLA	35	UGRCC2-78	NA	20.5	3.1
2014	NUS	2014_IN_WLA	36	UGRCC5-116	NA	10.5	1.6
2014	NUS	2014_IN_WLA	37	IL09-24328	NA	40.0	3.6
2014	NUS	2014_IN_WLA	38	IL09-3264	NA	15.5	3.0
2014	NUS	2014_IN_WLA	39	IL10-19464	NA	5.5	0.6
2014	NUS	2014_IN_WLA	40	IL10-6855	NA	20.0	2.0
2014	NUS	2014_IN_WLA	41	KY05C-1020-4-6-5	NA	5.5	1.3
2014	NUS	2014_IN_WLA	42	KY05C-1105-43-6-1	NA	10.5	2.6
2014	NUS	2014_IN_WLA	43	KY06C-3003-43-13-3	NA	15.5	3.2
2014	NUS	2014_IN_WLA	44	KY204604	NA	10.5	2.1
2014	NUS	2014_IN_WLA	45	MD08-22-22-13-4	NA	10.0	1.3
2014	NUS	2014_IN_WLA	46	MD08-22-22-13-10	NA	3.0	1.5
2014	NUS	2014_IN_WLA	47	MD09W272-8-4-13-3	NA	10.5	1.7
2014	NUS	2014_IN_WLA	48	MDC07026-12-28	NA	17.5	0.8
2014	NUS	2014_IN_WLA	49	MO120194	NA	3.0	0.8
2014	NUS	2014_IN_WLA	50	MO120452	NA	15.5	1.8
2014	NUS	2014_IN_WLA	51	MO120794	NA	5.0	1.7
2014	NUS	2014_IN_WLA	52	MO121183	NA	17.5	2.1
2014	NUS	2014_IN_WLA	53	NE06545	NA	17.5	1.9
2014	NUS	2014_IN_WLA	54	NE08499	NA	17.5	1.9
2014	NUS	2014_IN_WLA	55	NE10478	NA	10.5	3.3
2014	NUS	2014_IN_WLA	56	NI12702W	NA	12.5	1.9
2014	NUS	2014_IN_WLA	57	VA10W-140	NA	25.5	3.3
2014	NUS	2014_IN_WLA	58	VA11W-106	NA	20.0	2.3
2014	NUS	2014_IN_WLA	59	VA11W-301	NA	10.5	0.8
2014	NUS	2014_IN_WLA	60	VA12FHB-53	NA	10.0	0.7
2014	NUS	2014_OH_WOO	1	TRUMAN	33.7	40.0	18.8
2014	NUS	2014_OH_WOO	2	ERNIE	31.0	20.0	16.5
2014	NUS	2014_OH_WOO	3	FREEDOM	42.0	80.0	19.8
2014	NUS	2014_OH_WOO	4	PIONEER2545	74.8	85.0	24.2
2014	NUS	2014_OH_WOO	5	NY01016-AN	54.9	40.0	21.6
2014	NUS	2014_OH_WOO	6	NY01066-278	74.8	50.0	21.0
2014	NUS	2014_OH_WOO	7	NY99059-249	59.7	50.0	19.4
2014	NUS	2014_OH_WOO	8	NY99069-249	62.3	45.0	24.5
2014	NUS	2014_OH_WOO	9	NY99069-352	71.3	65.0	31.3
2014	NUS	2014_OH_WOO	10	KWS023	51.0	50.0	14.8
2014	NUS	2014_OH_WOO	11	KWS024	37.8	60.0	24.3
2014	NUS	2014_OH_WOO	12	KWS025	65.2	40.0	16.7

2014	NUS	2014_OH_WOO	13	KWS028	61.6	65.0	24.4
2014	NUS	2014_OH_WOO	14	L29230	55.5	55.0	19.1
2014	NUS	2014_OH_WOO	15	LCS321	53.8	50.0	26.0
2014	NUS	2014_OH_WOO	16	E6012	65.3	40.0	20.9
2014	NUS	2014_OH_WOO	17	F0036R	74.4	75.0	17.4
2014	NUS	2014_OH_WOO	18	F0039	76.5	95.0	26.0
2014	NUS	2014_OH_WOO	19	F1014	55.7	75.0	29.9
2014	NUS	2014_OH_WOO	20	OH07-263-3	30.0	30.0	16.3
2014	NUS	2014_OH_WOO	21	OH08-206-69	34.0	40.0	15.5
2014	NUS	2014_OH_WOO	22	OH08-269-58	58.7	50.0	13.9
2014	NUS	2014_OH_WOO	23	0570A1-2-32-5-1-4	20.1	40.0	20.6
2014	NUS	2014_OH_WOO	24	0762A1-2-8	17.2	25.0	13.7
2014	NUS	2014_OH_WOO	25	08334A1-31	24.0	25.0	14.0
2014	NUS	2014_OH_WOO	26	10641B1-9-11-7	14.4	75.0	41.1
2014	NUS	2014_OH_WOO	27	B08-91993	47.7	60.0	17.3
2014	NUS	2014_OH_WOO	28	B09-900256	69.8	70.0	14.6
2014	NUS	2014_OH_WOO	29	M09L-9547	42.8	80.0	26.9
2014	NUS	2014_OH_WOO	30	M10-1100	54.0	70.0	16.8
2014	NUS	2014_OH_WOO	31	M11-1027	54.0	70.0	23.3
2014	NUS	2014_OH_WOO	32	M11-2298	35.9	70.0	32.1
2014	NUS	2014_OH_WOO	33	GL133	63.3	80.0	25.8
2014	NUS	2014_OH_WOO	34	GL164	37.1	70.0	27.5
2014	NUS	2014_OH_WOO	35	UGRCC2-78	80.8	80.0	36.1
2014	NUS	2014_OH_WOO	36	UGRCC5-116	57.9	50.0	21.7
2014	NUS	2014_OH_WOO	37	IL09-24328	16.8	20.0	13.4
2014	NUS	2014_OH_WOO	38	IL09-3264	15.8	25.0	12.3
2014	NUS	2014_OH_WOO	39	IL10-19464	17.3	15.0	12.6
2014	NUS	2014_OH_WOO	40	IL10-6855	25.2	25.0	18.0

2014	NUS	2014_OH_WOO	41	KY05C-1020-4-6-5	44.8	70.0	41.1
2014	NUS	2014_OH_WOO	42	KY05C-1105-43-6-1	21.2	50.0	18.2
2014	NUS	2014_OH_WOO	43	KY06C-3003-43-13-3	40.9	40.0	23.3
2014	NUS	2014_OH_WOO	44	KY204604	73.4	60.0	35.0
2014	NUS	2014_OH_WOO	45	MD08-22-22-13-4	17.8	20.0	16.1
2014	NUS	2014_OH_WOO	46	MD08-22-22-13-10	14.5	25.0	17.4
2014	NUS	2014_OH_WOO	47	MD09W272-8-4-13-3	27.0	40.0	19.5
2014	NUS	2014_OH_WOO	48	MDC07026-12-28	33.3	40.0	21.6
2014	NUS	2014_OH_WOO	49	MO120194	12.5	25.0	14.0
2014	NUS	2014_OH_WOO	50	MO120452	29.8	20.0	18.8
2014	NUS	2014_OH_WOO	51	MO120794	34.6	30.0	28.8
2014	NUS	2014_OH_WOO	52	MO121183	47.1	35.0	15.9
2014	NUS	2014_OH_WOO	53	NE06545	43.5	80.0	26.1
2014	NUS	2014_OH_WOO	54	NE08499	40.4	80.0	29.1
2014	NUS	2014_OH_WOO	55	NE10478	44.0	85.0	22.8
2014	NUS	2014_OH_WOO	56	NI12702W	62.5	85.0	34.7
2014	NUS	2014_OH_WOO	57	VA10W-140	62.3	50.0	20.1
2014	NUS	2014_OH_WOO	58	VA11W-106	48.8	60.0	29.5
2014	NUS	2014_OH_WOO	59	VA11W-301	75.2	85.0	37.4
2014	NUS	2014_OH_WOO	60	VA12FHB-53	45.8	75.0	34.9
2015	NUS	2015_VA_BLA	1	TRUMAN	9.1	NA	NA
2015	NUS	2015_VA_BLA	2	ERNIE	26.1	NA	NA
2015	NUS	2015_VA_BLA	3	FREEDOM	9.8	NA	NA
2015	NUS	2015_VA_BLA	4	PIONEER2545	41.7	NA	NA
2015	NUS	2015_VA_BLA	5	NY99056-161	30.5	NA	NA
2015	NUS	2015_VA_BLA	6	NY09067-2-69-1097	21.3	NA	NA
2015	NUS	2015_VA_BLA	7	NY05152-818	24.9	NA	NA
2015	NUS	2015_VA_BLA	8	NY05152-825	8.5	NA	NA
2015	NUS	2015_VA_BLA	9	NY05152-821	46.9	NA	NA
2015	NUS	2015_VA_BLA	10	KWS050	18.5	NA	NA
2015	NUS	2015_VA_BLA	11	KWS051	14.0	NA	NA
2015	NUS	2015_VA_BLA	12	KWS052	14.1	NA	NA
2015	NUS	2015_VA_BLA	13	KWS036	22.4	NA	NA
2015	NUS	2015_VA_BLA	14	ES12-3030	10.5	NA	NA

2015	NUS	2015_VA_BLA	15	ES12-1358	9.0	NA	NA
2015	NUS	2015_VA_BLA	16	ES12-1275	11.8	NA	NA
2015	NUS	2015_VA_BLA	17	F1014	14.8	NA	NA
2015	NUS	2015_VA_BLA	18	E6012	23.5	NA	NA
2015	NUS	2015_VA_BLA	19	OH09-207-24	42.0	NA	NA
2015	NUS	2015_VA_BLA	20	OH09-281-10	11.4	NA	NA
2015	NUS	2015_VA_BLA	21	OH10-200-49	22.5	NA	NA
2015	NUS	2015_VA_BLA	22	10641B1-9-11-7	16.8	NA	NA
2015	NUS	2015_VA_BLA	23	0762A1-2-8	7.0	NA	NA
2015	NUS	2015_VA_BLA	24	08334A1-31	15.9	NA	NA
2015	NUS	2015_VA_BLA	25	0566A1-3-1-6	22.3	NA	NA
2015	NUS	2015_VA_BLA	26	10512RA1-8	9.6	NA	NA
2015	NUS	2015_VA_BLA	27	M11-2024	13.5	NA	NA
2015	NUS	2015_VA_BLA	28	M12-3312CW	20.9	NA	NA
2015	NUS	2015_VA_BLA	29	M12-3301	15.0	NA	NA
2015	NUS	2015_VA_BLA	30	M12-2036	9.3	NA	NA
2015	NUS	2015_VA_BLA	31	M12-2031	20.3	NA	NA
2015	NUS	2015_VA_BLA	32	CA9-72	8.2	NA	NA
2015	NUS	2015_VA_BLA	33	CA9-76	26.4	NA	NA
2015	NUS	2015_VA_BLA	34	DH5-15	24.0	NA	NA
2015	NUS	2015_VA_BLA	35	CA13-53	15.5	NA	NA
2015	NUS	2015_VA_BLA	36	CA13-63	15.0	NA	NA
2015	NUS	2015_VA_BLA	37	IL10-19464	14.7	NA	NA
2015	NUS	2015_VA_BLA	38	IL10-21934	9.9	NA	NA
2015	NUS	2015_VA_BLA	39	IL10-21937	7.0	NA	NA
2015	NUS	2015_VA_BLA	40	IL11-36131	16.4	NA	NA
2015	NUS	2015_VA_BLA	41	IL11-27667	13.5	NA	NA
2015	NUS	2015_VA_BLA	42	KY06C-1195-37-2-5	24.4	NA	NA
2015	NUS	2015_VA_BLA	43	KY06C-1201-18-6-3	29.9	NA	NA
2015	NUS	2015_VA_BLA	44	KY06C-1107-7-2-5	25.8	NA	NA
2015	NUS	2015_VA_BLA	45	KY06C-2020-10-5-3	18.6	NA	NA
2015	NUS	2015_VA_BLA	46	KY06C-2020-11-12-1	23.0	NA	NA
2015	NUS	2015_VA_BLA	47	MO122246	7.0	NA	NA
2015	NUS	2015_VA_BLA	48	MO130203	12.0	NA	NA
2015	NUS	2015_VA_BLA	49	MO130765	8.8	NA	NA
2015	NUS	2015_VA_BLA	50	MO131838	8.8	NA	NA
2015	NUS	2015_VA_BLA	51	NE05548	19.8	NA	NA
2015	NUS	2015_VA_BLA	52	NE10589	48.4	NA	NA
2015	NUS	2015_VA_BLA	53	NW13455	24.8	NA	NA
2015	NUS	2015_VA_BLA	54	NE13511	20.3	NA	NA
2015	NUS	2015_VA_BLA	55	NE06545	16.9	NA	NA
2015	NUS	2015_VA_BLA	56	VA11W-108	32.9	NA	NA
2015	NUS	2015_VA_BLA	57	VA11W-182	36.0	NA	NA
2015	NUS	2015_VA_BLA	58	VA12W-150	13.9	NA	NA
2015	NUS	2015_VA_BLA	59	VA12FHB-4	10.5	NA	NA
2015	NUS	2015_VA_BLA	60	VA12FHB-55	8.0	NA	NA
2015	NUS	2015_IL_CHA	1	TRUMAN	15.0	NA	NA
2015	NUS	2015_IL_CHA	2	ERNIE	25.0	NA	NA
2015	NUS	2015_IL_CHA	3	FREEDOM	45.0	NA	NA

2015	NUS	2015_IL_CHA	4	PIONEER2545	85.0	NA	NA
2015	NUS	2015_IL_CHA	5	NY99056-161	27.5	NA	NA
2015	NUS	2015_IL_CHA	6	NY09067-2-69-1097	40.0	NA	NA
2015	NUS	2015_IL_CHA	7	NY05152-818	20.0	NA	NA
2015	NUS	2015_IL_CHA	8	NY05152-825	15.0	NA	NA
2015	NUS	2015_IL_CHA	9	NY05152-821	15.0	NA	NA
2015	NUS	2015_IL_CHA	10	KWS050	45.0	NA	NA
2015	NUS	2015_IL_CHA	11	KWS051	70.0	NA	NA
2015	NUS	2015_IL_CHA	12	KWS052	50.0	NA	NA
2015	NUS	2015_IL_CHA	13	KWS036	65.0	NA	NA
2015	NUS	2015_IL_CHA	14	ES12-3030	40.0	NA	NA
2015	NUS	2015_IL_CHA	15	ES12-1358	35.0	NA	NA
2015	NUS	2015_IL_CHA	16	ES12-1275	22.5	NA	NA
2015	NUS	2015_IL_CHA	17	F1014	22.5	NA	NA
2015	NUS	2015_IL_CHA	18	E6012	30.0	NA	NA
2015	NUS	2015_IL_CHA	19	OH09-207-24	17.5	NA	NA
2015	NUS	2015_IL_CHA	20	OH09-281-10	45.0	NA	NA
2015	NUS	2015_IL_CHA	21	OH10-200-49	22.5	NA	NA
2015	NUS	2015_IL_CHA	22	10641B1-9-11-7	15.0	NA	NA
2015	NUS	2015_IL_CHA	23	0762A1-2-8	30.0	NA	NA
2015	NUS	2015_IL_CHA	24	08334A1-31	20.0	NA	NA
2015	NUS	2015_IL_CHA	25	0566A1-3-1-6	35.0	NA	NA
2015	NUS	2015_IL_CHA	26	10512RA1-8	15.0	NA	NA
2015	NUS	2015_IL_CHA	27	M11-2024	75.0	NA	NA
2015	NUS	2015_IL_CHA	28	M12-3312CW	15.0	NA	NA
2015	NUS	2015_IL_CHA	29	M12-3301	20.0	NA	NA
2015	NUS	2015_IL_CHA	30	M12-2036	NA	NA	NA
2015	NUS	2015_IL_CHA	31	M12-2031	NA	NA	NA
2015	NUS	2015_IL_CHA	32	CA9-72	NA	NA	NA
2015	NUS	2015_IL_CHA	33	CA9-76	30.0	NA	NA
2015	NUS	2015_IL_CHA	34	DH5-15	65.0	NA	NA
2015	NUS	2015_IL_CHA	35	CA13-53	27.5	NA	NA
2015	NUS	2015_IL_CHA	36	CA13-63	25.0	NA	NA
2015	NUS	2015_IL_CHA	37	IL10-19464	20.0	NA	NA
2015	NUS	2015_IL_CHA	38	IL10-21934	22.5	NA	NA
2015	NUS	2015_IL_CHA	39	IL10-21937	20.0	NA	NA
2015	NUS	2015_IL_CHA	40	IL11-36131	35.0	NA	NA
2015	NUS	2015_IL_CHA	41	IL11-27667	25.0	NA	NA
2015	NUS	2015_IL_CHA	42	KY06C-1195-37-2-5	20.0	NA	NA
2015	NUS	2015_IL_CHA	43	KY06C-1201-18-6-3	25.0	NA	NA
2015	NUS	2015_IL_CHA	44	KY06C-1107-7-2-5	35.0	NA	NA
2015	NUS	2015_IL_CHA	45	KY06C-2020-10-5-3	40.0	NA	NA
2015	NUS	2015_IL_CHA	46	KY06C-2020-11-12-1	15.0	NA	NA
2015	NUS	2015_IL_CHA	47	MO122246	20.0	NA	NA
2015	NUS	2015_IL_CHA	48	MO130203	20.0	NA	NA
2015	NUS	2015_IL_CHA	49	MO130765	35.0	NA	NA
2015	NUS	2015_IL_CHA	50	MO131838	17.5	NA	NA
2015	NUS	2015_IL_CHA	51	NE05548	60.0	NA	NA
2015	NUS	2015_IL_CHA	52	NE10589	65.0	NA	NA

2015	NUS	2015_IL_CHA	53	NW13455	35.0	NA	NA
2015	NUS	2015_IL_CHA	54	NE13511	35.0	NA	NA
2015	NUS	2015_IL_CHA	55	NE06545	30.0	NA	NA
2015	NUS	2015_IL_CHA	56	VA11W-108	17.5	NA	NA
2015	NUS	2015_IL_CHA	57	VA11W-182	27.5	NA	NA
2015	NUS	2015_IL_CHA	58	VA12W-150	15.0	NA	NA
2015	NUS	2015_IL_CHA	59	VA12FHB-4	40.0	NA	NA
2015	NUS	2015_IL_CHA	60	VA12FHB-55	20.0	NA	NA
2015	NUS	2015_MO_COL	1	TRUMAN	65.0	12.5	4.4
2015	NUS	2015_MO_COL	2	ERNIE	70.0	35.0	4.2
2015	NUS	2015_MO_COL	3	FREEDOM	87.5	50.0	5.0
2015	NUS	2015_MO_COL	4	PIONEER2545	95.0	85.0	11.1
2015	NUS	2015_MO_COL	5	NY99056-161	90.0	55.0	16.7
2015	NUS	2015_MO_COL	6	NY09067-2-69-1097	80.0	62.5	7.3
2015	NUS	2015_MO_COL	7	NY05152-818	100.0	75.0	9.5
2015	NUS	2015_MO_COL	8	NY05152-825	92.5	35.0	5.0
2015	NUS	2015_MO_COL	9	NY05152-821	100.0	87.5	8.5
2015	NUS	2015_MO_COL	10	KWS050	70.0	60.0	5.4
2015	NUS	2015_MO_COL	11	KWS051	70.0	45.0	4.4
2015	NUS	2015_MO_COL	12	KWS052	67.5	42.5	7.0
2015	NUS	2015_MO_COL	13	KWS036	60.0	50.0	27.6
2015	NUS	2015_MO_COL	14	ES12-3030	90.0	25.0	10.5
2015	NUS	2015_MO_COL	15	ES12-1358	85.0	15.0	2.8
2015	NUS	2015_MO_COL	16	ES12-1275	60.0	30.0	4.8
2015	NUS	2015_MO_COL	17	F1014	95.0	42.5	9.9
2015	NUS	2015_MO_COL	18	E6012	62.5	40.0	23.8
2015	NUS	2015_MO_COL	19	OH09-207-24	62.5	25.0	2.9
2015	NUS	2015_MO_COL	20	OH09-281-10	80.0	85.0	14.2
2015	NUS	2015_MO_COL	21	OH10-200-49	70.0	25.0	5.9
2015	NUS	2015_MO_COL	22	10641B1-9-11-7	87.5	10.0	3.9
2015	NUS	2015_MO_COL	23	0762A1-2-8	80.0	17.5	2.4

2015	NUS	2015_MO_COL	24	08334A1-31	90.0	60.0	5.0
2015	NUS	2015_MO_COL	25	0566A1-3-1-6	77.5	22.5	6.9
2015	NUS	2015_MO_COL	26	10512RA1-8	90.0	65.0	6.9
2015	NUS	2015_MO_COL	27	M11-2024	52.5	40.0	7.4
2015	NUS	2015_MO_COL	28	M12-3312CW	67.5	40.0	34.9
2015	NUS	2015_MO_COL	29	M12-3301	72.5	27.5	4.6
2015	NUS	2015_MO_COL	30	M12-2036	55.0	70.0	10.3
2015	NUS	2015_MO_COL	31	M12-2031	72.5	55.0	16.0
2015	NUS	2015_MO_COL	32	CA9-72	85.0	35.0	5.8
2015	NUS	2015_MO_COL	33	CA9-76	70.0	40.0	6.6
2015	NUS	2015_MO_COL	34	DH5-15	77.5	32.5	8.9
2015	NUS	2015_MO_COL	35	CA13-53	100.0	80.0	14.1
2015	NUS	2015_MO_COL	36	CA13-63	72.5	55.0	9.5
2015	NUS	2015_MO_COL	37	IL10-19464	75.0	7.5	3.7
2015	NUS	2015_MO_COL	38	IL10-21934	65.0	12.5	5.3
2015	NUS	2015_MO_COL	39	IL10-21937	35.0	7.5	3.4
2015	NUS	2015_MO_COL	40	IL11-36131	27.5	7.5	3.0
2015	NUS	2015_MO_COL	41	IL11-27667	77.5	45.0	2.6
2015	NUS	2015_MO_COL	42	KY06C-1195-37-2-5	85.0	35.0	4.9
2015	NUS	2015_MO_COL	43	KY06C-1201-18-6-3	62.5	35.0	4.1
2015	NUS	2015_MO_COL	44	KY06C-1107-7-2-5	77.5	30.0	4.5
2015	NUS	2015_MO_COL	45	KY06C-2020-10-5-3	55.0	10.0	6.3
2015	NUS	2015_MO_COL	46	KY06C-2020-11-12-1	77.5	20.0	3.3
2015	NUS	2015_MO_COL	47	MO122246	87.5	15.0	5.2
2015	NUS	2015_MO_COL	48	MO130203	95.0	12.5	6.9
2015	NUS	2015_MO_COL	49	MO130765	85.0	40.0	6.9
2015	NUS	2015_MO_COL	50	MO131838	75.0	30.0	9.1
2015	NUS	2015_MO_COL	51	NE05548	100.0	85.0	6.8

2015	NUS	2015_MO_COL	52	NE10589	97.5	80.0	11.9
2015	NUS	2015_MO_COL	53	NW13455	95.0	50.0	20.3
2015	NUS	2015_MO_COL	54	NE13511	97.5	55.0	7.7
2015	NUS	2015_MO_COL	55	NE06545	90.0	25.0	4.8
2015	NUS	2015_MO_COL	56	VA11W-108	87.5	40.0	9.1
2015	NUS	2015_MO_COL	57	VA11W-182	70.0	65.0	4.8
2015	NUS	2015_MO_COL	58	VA12W-150	75.0	40.0	7.1
2015	NUS	2015_MO_COL	59	VA12FHB-4	77.5	12.5	6.3
2015	NUS	2015_MO_COL	60	VA12FHB-55	87.5	25.0	2.1
2015	NUS	2015_MI_ELA	1	TRUMAN	26.5	NA	NA
2015	NUS	2015_MI_ELA	2	ERNIE	31.7	NA	NA
2015	NUS	2015_MI_ELA	3	FREEDOM	24.4	NA	NA
2015	NUS	2015_MI_ELA	4	PIONEER2545	42.7	NA	NA
2015	NUS	2015_MI_ELA	5	NY99056-161	27.6	NA	NA
2015	NUS	2015_MI_ELA	6	NY09067-2-69-1097	16.7	NA	NA
2015	NUS	2015_MI_ELA	7	NY05152-818	20.9	NA	NA
2015	NUS	2015_MI_ELA	8	NY05152-825	20.8	NA	NA
2015	NUS	2015_MI_ELA	9	NY05152-821	34.5	NA	NA
2015	NUS	2015_MI_ELA	10	KWS050	21.9	NA	NA
2015	NUS	2015_MI_ELA	11	KWS051	19.8	NA	NA
2015	NUS	2015_MI_ELA	12	KWS052	22.2	NA	NA
2015	NUS	2015_MI_ELA	13	KWS036	33.2	NA	NA
2015	NUS	2015_MI_ELA	14	ES12-3030	23.4	NA	NA
2015	NUS	2015_MI_ELA	15	ES12-1358	20.4	NA	NA
2015	NUS	2015_MI_ELA	16	ES12-1275	14.2	NA	NA
2015	NUS	2015_MI_ELA	17	F1014	26.0	NA	NA
2015	NUS	2015_MI_ELA	18	E6012	26.6	NA	NA
2015	NUS	2015_MI_ELA	19	OH09-207-24	16.3	NA	NA
2015	NUS	2015_MI_ELA	20	OH09-281-10	17.9	NA	NA
2015	NUS	2015_MI_ELA	21	OH10-200-49	28.5	NA	NA
2015	NUS	2015_MI_ELA	22	10641B1-9-11-7	14.1	NA	NA
2015	NUS	2015_MI_ELA	23	0762A1-2-8	13.9	NA	NA
2015	NUS	2015_MI_ELA	24	08334A1-31	21.4	NA	NA
2015	NUS	2015_MI_ELA	25	0566A1-3-1-6	28.6	NA	NA
2015	NUS	2015_MI_ELA	26	10512RA1-8	19.1	NA	NA
2015	NUS	2015_MI_ELA	27	M11-2024	16.6	NA	NA
2015	NUS	2015_MI_ELA	28	M12-3312CW	17.5	NA	NA
2015	NUS	2015_MI_ELA	29	M12-3301	23.6	NA	NA
2015	NUS	2015_MI_ELA	30	M12-2036	24.5	NA	NA
2015	NUS	2015_MI_ELA	31	M12-2031	18.4	NA	NA
2015	NUS	2015_MI_ELA	32	CA9-72	30.5	NA	NA
2015	NUS	2015_MI_ELA	33	CA9-76	23.5	NA	NA

2015	NUS	2015_MI_ELA	34	DH5-15	31.6	NA	NA
2015	NUS	2015_MI_ELA	35	CA13-53	18.1	NA	NA
2015	NUS	2015_MI_ELA	36	CA13-63	25.9	NA	NA
2015	NUS	2015_MI_ELA	37	IL10-19464	11.8	NA	NA
2015	NUS	2015_MI_ELA	38	IL10-21934	17.7	NA	NA
2015	NUS	2015_MI_ELA	39	IL10-21937	21.0	NA	NA
2015	NUS	2015_MI_ELA	40	IL11-36131	23.7	NA	NA
2015	NUS	2015_MI_ELA	41	IL11-27667	22.3	NA	NA
2015	NUS	2015_MI_ELA	42	KY06C-1195-37-2-5	21.5	NA	NA
2015	NUS	2015_MI_ELA	43	KY06C-1201-18-6-3	22.9	NA	NA
2015	NUS	2015_MI_ELA	44	KY06C-1107-7-2-5	14.9	NA	NA
2015	NUS	2015_MI_ELA	45	KY06C-2020-10-5-3	23.5	NA	NA
2015	NUS	2015_MI_ELA	46	KY06C-2020-11-12-1	15.9	NA	NA
2015	NUS	2015_MI_ELA	47	MO122246	11.7	NA	NA
2015	NUS	2015_MI_ELA	48	MO130203	26.6	NA	NA
2015	NUS	2015_MI_ELA	49	MO130765	26.7	NA	NA
2015	NUS	2015_MI_ELA	50	MO131838	28.5	NA	NA
2015	NUS	2015_MI_ELA	51	NE05548	28.4	NA	NA
2015	NUS	2015_MI_ELA	52	NE10589	34.3	NA	NA
2015	NUS	2015_MI_ELA	53	NW13455	32.0	NA	NA
2015	NUS	2015_MI_ELA	54	NE13511	31.0	NA	NA
2015	NUS	2015_MI_ELA	55	NE06545	19.4	NA	NA
2015	NUS	2015_MI_ELA	56	VA11W-108	24.2	NA	NA
2015	NUS	2015_MI_ELA	57	VA11W-182	23.4	NA	NA
2015	NUS	2015_MI_ELA	58	VA12W-150	18.8	NA	NA
2015	NUS	2015_MI_ELA	59	VA12FHB-4	15.9	NA	NA
2015	NUS	2015_MI_ELA	60	VA12FHB-55	14.8	NA	NA
2015	NUS	2015_IL_HIG	1	TRUMAN	10.0	15.0	NA
2015	NUS	2015_IL_HIG	2	ERNIE	65.0	4.0	NA
2015	NUS	2015_IL_HIG	3	FREEDOM	30.0	77.5	NA
2015	NUS	2015_IL_HIG	4	PIONEER2545	55.0	82.5	NA
2015	NUS	2015_IL_HIG	5	NY99056-161	45.0	32.5	NA
2015	NUS	2015_IL_HIG	6	NY09067-2-69-1097	30.0	67.5	NA
2015	NUS	2015_IL_HIG	7	NY05152-818	20.0	52.5	NA
2015	NUS	2015_IL_HIG	8	NY05152-825	20.0	75.0	NA
2015	NUS	2015_IL_HIG	9	NY05152-821	15.0	27.5	NA
2015	NUS	2015_IL_HIG	10	KWS050	30.0	27.5	NA
2015	NUS	2015_IL_HIG	11	KWS051	45.0	27.5	NA
2015	NUS	2015_IL_HIG	12	KWS052	45.0	62.5	NA
2015	NUS	2015_IL_HIG	13	KWS036	30.0	70.0	NA
2015	NUS	2015_IL_HIG	14	ES12-3030	45.0	25.0	NA
2015	NUS	2015_IL_HIG	15	ES12-1358	30.0	11.5	NA
2015	NUS	2015_IL_HIG	16	ES12-1275	40.0	17.5	NA
2015	NUS	2015_IL_HIG	17	F1014	10.0	32.5	NA
2015	NUS	2015_IL_HIG	18	E6012	30.0	35.0	NA
2015	NUS	2015_IL_HIG	19	OH09-207-24	25.0	10.0	NA
2015	NUS	2015_IL_HIG	20	OH09-281-10	20.0	50.0	NA
2015	NUS	2015_IL_HIG	21	OH10-200-49	10.0	6.5	NA
2015	NUS	2015_IL_HIG	22	10641B1-9-11-7	35.0	15.0	NA

2015	NUS	2015_IL_HIG	23	0762A1-2-8	10.0	40.0	NA
2015	NUS	2015_IL_HIG	24	08334A1-31	25.0	27.5	NA
2015	NUS	2015_IL_HIG	25	0566A1-3-1-6	45.0	25.0	NA
2015	NUS	2015_IL_HIG	26	10512RA1-8	10.0	50.0	NA
2015	NUS	2015_IL_HIG	27	M11-2024	35.0	14.0	NA
2015	NUS	2015_IL_HIG	28	M12-3312CW	45.0	6.5	NA
2015	NUS	2015_IL_HIG	29	M12-3301	10.0	6.5	NA
2015	NUS	2015_IL_HIG	30	M12-2036	25.0	73.5	NA
2015	NUS	2015_IL_HIG	31	M12-2031	20.0	37.5	NA
2015	NUS	2015_IL_HIG	32	CA9-72	65.0	88.5	NA
2015	NUS	2015_IL_HIG	33	CA9-76	60.0	45.0	NA
2015	NUS	2015_IL_HIG	34	DH5-15	35.0	7.5	NA
2015	NUS	2015_IL_HIG	35	CA13-53	15.0	35.0	NA
2015	NUS	2015_IL_HIG	36	CA13-63	20.0	25.0	NA
2015	NUS	2015_IL_HIG	37	IL10-19464	30.0	10.0	NA
2015	NUS	2015_IL_HIG	38	IL10-21934	25.0	15.0	NA
2015	NUS	2015_IL_HIG	39	IL10-21937	50.0	15.0	NA
2015	NUS	2015_IL_HIG	40	IL11-36131	30.0	15.0	NA
2015	NUS	2015_IL_HIG	41	IL11-27667	35.0	7.5	NA
2015	NUS	2015_IL_HIG	42	KY06C-1195-37-2-5	25.0	15.0	NA
2015	NUS	2015_IL_HIG	43	KY06C-1201-18-6-3	40.0	50.0	NA
2015	NUS	2015_IL_HIG	44	KY06C-1107-7-2-5	55.0	11.5	NA
2015	NUS	2015_IL_HIG	45	KY06C-2020-10-5-3	30.0	55.0	NA
2015	NUS	2015_IL_HIG	46	KY06C-2020-11-12-1	25.0	3.0	NA
2015	NUS	2015_IL_HIG	47	MO122246	20.0	27.5	NA
2015	NUS	2015_IL_HIG	48	MO130203	30.0	4.0	NA
2015	NUS	2015_IL_HIG	49	MO130765	10.0	39.0	NA
2015	NUS	2015_IL_HIG	50	MO131838	10.0	30.0	NA
2015	NUS	2015_IL_HIG	51	NE05548	80.0	77.5	NA
2015	NUS	2015_IL_HIG	52	NE10589	45.0	80.0	NA
2015	NUS	2015_IL_HIG	53	NW13455	30.0	55.0	NA
2015	NUS	2015_IL_HIG	54	NE13511	50.0	75.0	NA
2015	NUS	2015_IL_HIG	55	NE06545	15.0	25.0	NA
2015	NUS	2015_IL_HIG	56	VA11W-108	45.0	40.0	NA
2015	NUS	2015_IL_HIG	57	VA11W-182	30.0	32.5	NA
2015	NUS	2015_IL_HIG	58	VA12W-150	50.0	22.5	NA
2015	NUS	2015_IL_HIG	59	VA12FHB-4	25.0	7.5	NA
2015	NUS	2015_IL_HIG	60	VA12FHB-55	20.0	10.0	NA
2015	NUS	2015_NY_ITH	2	ERNIE	10.4	45.0	2.4
2015	NUS	2015_NY_ITH	3	FREEDOM	21.0	70.0	4.0
2015	NUS	2015_NY_ITH	4	PIONEER2545	15.4	90.0	8.6
2015	NUS	2015_NY_ITH	5	NY99056-161	15.8	35.0	2.9
2015	NUS	2015_NY_ITH	6	NY09067-2-69-1097	16.8	70.0	3.1
2015	NUS	2015_NY_ITH	7	NY05152-818	13.0	45.0	3.0
2015	NUS	2015_NY_ITH	8	NY05152-825	9.6	30.0	1.2
2015	NUS	2015_NY_ITH	9	NY05152-821	9.6	60.0	3.2
2015	NUS	2015_NY_ITH	10	KWS050	19.8	45.0	3.7
2015	NUS	2015_NY_ITH	11	KWS051	14.8	55.0	1.2
2015	NUS	2015_NY_ITH	12	KWS052	16.4	70.0	1.1

2015	NUS	2015_NY_ITH	13	KWS036	22.4	55.0	9.2
2015	NUS	2015_NY_ITH	14	ES12-3030	10.0	55.0	3.5
2015	NUS	2015_NY_ITH	15	ES12-1358	7.8	25.0	1.4
2015	NUS	2015_NY_ITH	16	ES12-1275	9.0	65.0	1.0
2015	NUS	2015_NY_ITH	17	F1014	13.8	45.0	4.7
2015	NUS	2015_NY_ITH	18	E6012	13.2	45.0	4.9
2015	NUS	2015_NY_ITH	19	OH09-207-24	10.8	50.0	1.9
2015	NUS	2015_NY_ITH	20	OH09-281-10	17.8	70.0	3.7
2015	NUS	2015_NY_ITH	21	OH10-200-49	10.2	35.0	2.0
2015	NUS	2015_NY_ITH	22	10641B1-9-11-7	11.0	55.0	1.9
2015	NUS	2015_NY_ITH	23	0762A1-2-8	11.6	70.0	1.5
2015	NUS	2015_NY_ITH	24	08334A1-31	13.4	60.0	2.2
2015	NUS	2015_NY_ITH	25	0566A1-3-1-6	14.0	45.0	1.1
2015	NUS	2015_NY_ITH	26	10512RA1-8	10.4	70.0	2.4
2015	NUS	2015_NY_ITH	27	M11-2024	19.8	55.0	3.7
2015	NUS	2015_NY_ITH	28	M12-3312CW	15.8	45.0	4.9
2015	NUS	2015_NY_ITH	29	M12-3301	11.6	45.0	1.4
2015	NUS	2015_NY_ITH	30	M12-2036	8.2	30.0	2.8
2015	NUS	2015_NY_ITH	31	M12-2031	11.0	70.0	3.8
2015	NUS	2015_NY_ITH	32	CA9-72	23.0	60.0	5.6
2015	NUS	2015_NY_ITH	33	CA9-76	17.6	85.0	4.7
2015	NUS	2015_NY_ITH	34	DH5-15	15.6	75.0	4.9
2015	NUS	2015_NY_ITH	35	CA13-53	17.2	75.0	5.7
2015	NUS	2015_NY_ITH	36	CA13-63	8.6	45.0	2.3
2015	NUS	2015_NY_ITH	37	IL10-19464	14.4	30.0	2.3
2015	NUS	2015_NY_ITH	38	IL10-21934	19.4	30.0	2.2
2015	NUS	2015_NY_ITH	39	IL10-21937	12.4	50.0	2.2
2015	NUS	2015_NY_ITH	40	IL11-36131	7.0	25.0	0.9
2015	NUS	2015_NY_ITH	41	IL11-27667	9.2	10.0	0.5
2015	NUS	2015_NY_ITH	42	KY06C-1195-37-2-5	13.0	25.0	4.2
2015	NUS	2015_NY_ITH	43	KY06C-1201-18-6-3	15.4	40.0	2.0
2015	NUS	2015_NY_ITH	44	KY06C-1107-7-2-5	11.0	40.0	3.1
2015	NUS	2015_NY_ITH	45	KY06C-2020-10-5-3	16.6	50.0	2.4
2015	NUS	2015_NY_ITH	46	KY06C-2020-11-12-1	17.8	25.0	1.8
2015	NUS	2015_NY_ITH	47	MO122246	16.8	25.0	1.8
2015	NUS	2015_NY_ITH	48	MO130203	12.8	40.0	2.6
2015	NUS	2015_NY_ITH	49	MO130765	12.6	50.0	2.2
2015	NUS	2015_NY_ITH	50	MO131838	14.6	45.0	2.1
2015	NUS	2015_NY_ITH	51	NE05548	27.2	65.0	3.5
2015	NUS	2015_NY_ITH	52	NE10589	23.6	60.0	4.5
2015	NUS	2015_NY_ITH	53	NW13455	10.6	40.0	2.8
2015	NUS	2015_NY_ITH	54	NE13511	17.4	55.0	6.5
2015	NUS	2015_NY_ITH	55	NE06545	17.4	55.0	4.5
2015	NUS	2015_NY_ITH	56	VA11W-108	11.4	65.0	2.5
2015	NUS	2015_NY_ITH	57	VA11W-182	16.8	70.0	1.1
2015	NUS	2015_NY_ITH	58	VA12W-150	17.6	55.0	3.4
2015	NUS	2015_NY_ITH	59	VA12FHB-4	12.2	55.0	3.5
2015	NUS	2015_NY_ITH	60	VA12FHB-55	7.2	35.0	1.7
2015	NUS	2015_KY_LEX	1	TRUMAN	39.0	3.7	3.6

2015	NUS	2015_KY_LEX	2	ERNIE	40.3	9.9	8.6
2015	NUS	2015_KY_LEX	3	FREEDOM	45.1	9.5	5.1
2015	NUS	2015_KY_LEX	4	PIONEER2545	60.6	23.1	11.2
2015	NUS	2015_KY_LEX	5	NY99056-161	28.5	8.3	11.0
2015	NUS	2015_KY_LEX	6	NY09067-2-69-1097	41.6	13.7	11.4
2015	NUS	2015_KY_LEX	7	NY05152-818	32.7	6.4	6.7
2015	NUS	2015_KY_LEX	8	NY05152-825	39.9	8.7	4.8
2015	NUS	2015_KY_LEX	9	NY05152-821	32.8	11.2	9.0
2015	NUS	2015_KY_LEX	10	KWS050	37.2	14.5	7.2
2015	NUS	2015_KY_LEX	11	KWS051	29.9	6.8	3.9
2015	NUS	2015_KY_LEX	12	KWS052	27.8	14.5	6.3
2015	NUS	2015_KY_LEX	13	KWS036	44.7	12.7	17.4
2015	NUS	2015_KY_LEX	14	ES12-3030	48.5	8.2	5.2
2015	NUS	2015_KY_LEX	15	ES12-1358	30.8	3.3	1.9
2015	NUS	2015_KY_LEX	16	ES12-1275	17.2	3.6	5.9
2015	NUS	2015_KY_LEX	17	F1014	67.1	15.4	14.0
2015	NUS	2015_KY_LEX	18	E6012	54.6	7.3	14.8
2015	NUS	2015_KY_LEX	19	OH09-207-24	26.3	6.7	7.3
2015	NUS	2015_KY_LEX	20	OH09-281-10	33.4	14.7	10.7
2015	NUS	2015_KY_LEX	21	OH10-200-49	37.0	7.2	4.2
2015	NUS	2015_KY_LEX	22	10641B1-9-11-7	19.2	6.4	6.1
2015	NUS	2015_KY_LEX	23	0762A1-2-8	21.3	7.7	4.5
2015	NUS	2015_KY_LEX	24	08334A1-31	35.0	8.8	5.1
2015	NUS	2015_KY_LEX	25	0566A1-3-1-6	22.0	8.5	3.3
2015	NUS	2015_KY_LEX	26	10512RA1-8	27.2	13.4	9.0
2015	NUS	2015_KY_LEX	27	M11-2024	26.3	7.3	8.6
2015	NUS	2015_KY_LEX	28	M12-3312CW	26.5	6.8	10.3
2015	NUS	2015_KY_LEX	29	M12-3301	37.0	8.2	5.8
2015	NUS	2015_KY_LEX	30	M12-2036	23.3	9.5	7.1
2015	NUS	2015_KY_LEX	31	M12-2031	28.7	13.8	8.0
2015	NUS	2015_KY_LEX	32	CA9-72	29.4	15.9	7.4
2015	NUS	2015_KY_LEX	33	CA9-76	45.5	15.0	10.6
2015	NUS	2015_KY_LEX	34	DH5-15	46.3	12.6	12.0
2015	NUS	2015_KY_LEX	35	CA13-53	38.5	14.2	7.4
2015	NUS	2015_KY_LEX	36	CA13-63	30.4	7.7	3.7
2015	NUS	2015_KY_LEX	37	IL10-19464	30.5	8.2	5.4
2015	NUS	2015_KY_LEX	38	IL10-21934	22.6	3.3	2.6
2015	NUS	2015_KY_LEX	39	IL10-21937	17.5	4.8	3.6
2015	NUS	2015_KY_LEX	40	IL11-36131	20.2	4.2	3.1
2015	NUS	2015_KY_LEX	41	IL11-27667	37.1	8.2	5.9
2015	NUS	2015_KY_LEX	42	KY06C-1195-37-2-5	38.0	5.5	8.7
2015	NUS	2015_KY_LEX	43	KY06C-1201-18-6-3	19.1	6.1	4.2
2015	NUS	2015_KY_LEX	44	KY06C-1107-7-2-5	38.5	7.6	6.6
2015	NUS	2015_KY_LEX	45	KY06C-2020-10-5-3	56.6	15.8	8.2
2015	NUS	2015_KY_LEX	46	KY06C-2020-11-12-1	36.2	7.7	4.1
2015	NUS	2015_KY_LEX	47	MO122246	18.6	5.1	2.7
2015	NUS	2015_KY_LEX	48	MO130203	44.5	5.1	4.2
2015	NUS	2015_KY_LEX	49	MO130765	44.5	6.1	3.8
2015	NUS	2015_KY_LEX	50	MO131838	23.1	6.9	3.9

2015	NUS	2015_KY_LEX	51	NE05548	41.2	8.6	5.8
2015	NUS	2015_KY_LEX	52	NE10589	47.9	16.3	12.7
2015	NUS	2015_KY_LEX	53	NW13455	32.6	10.9	9.9
2015	NUS	2015_KY_LEX	54	NE13511	31.5	12.3	8.0
2015	NUS	2015_KY_LEX	55	NE06545	32.8	12.6	6.9
2015	NUS	2015_KY_LEX	56	VA11W-108	34.5	11.6	6.7
2015	NUS	2015_KY_LEX	57	VA11W-182	41.3	19.3	10.4
2015	NUS	2015_KY_LEX	58	VA12W-150	48.4	10.1	6.7
2015	NUS	2015_KY_LEX	59	VA12FHB-4	24.1	8.4	3.6
2015	NUS	2015_KY_LEX	60	VA12FHB-55	22.9	7.4	4.8
2015	NUS	2015_NE_MEA	1	TRUMAN	6.0	25.0	15.3
2015	NUS	2015_NE_MEA	2	ERNIE	23.7	19.0	NA
2015	NUS	2015_NE_MEA	3	FREEDOM	20.7	25.0	NA
2015	NUS	2015_NE_MEA	4	PIONEER2545	29.3	NA	36.9
2015	NUS	2015_NE_MEA	5	NY99056-161	13.7	30.0	12.8
2015	NUS	2015_NE_MEA	6	NY09067-2-69-1097	25.3	30.0	30.5
2015	NUS	2015_NE_MEA	7	NY05152-818	8.3	29.0	15.2
2015	NUS	2015_NE_MEA	8	NY05152-825	11.7	17.0	NA
2015	NUS	2015_NE_MEA	9	NY05152-821	16.7	11.0	NA
2015	NUS	2015_NE_MEA	10	KWS050	22.3	33.0	15.3
2015	NUS	2015_NE_MEA	11	KWS051	26.3	31.0	NA
2015	NUS	2015_NE_MEA	12	KWS052	42.7	15.0	13.1
2015	NUS	2015_NE_MEA	13	KWS036	26.7	40.0	32.9
2015	NUS	2015_NE_MEA	14	ES12-3030	40.3	NA	22.1
2015	NUS	2015_NE_MEA	15	ES12-1358	22.0	NA	10.2
2015	NUS	2015_NE_MEA	16	ES12-1275	18.0	14.0	NA
2015	NUS	2015_NE_MEA	17	F1014	10.7	28.0	18.9
2015	NUS	2015_NE_MEA	18	E6012	34.7	40.0	22.3
2015	NUS	2015_NE_MEA	19	OH09-207-24	18.3	24.0	NA
2015	NUS	2015_NE_MEA	20	OH09-281-10	40.7	26.0	21.6
2015	NUS	2015_NE_MEA	21	OH10-200-49	11.7	NA	11.5
2015	NUS	2015_NE_MEA	22	10641B1-9-11-7	17.3	30.0	9.7
2015	NUS	2015_NE_MEA	23	0762A1-2-8	13.3	NA	11.9
2015	NUS	2015_NE_MEA	24	08334A1-31	22.3	31.0	NA
2015	NUS	2015_NE_MEA	25	0566A1-3-1-6	37.7	NA	11.9
2015	NUS	2015_NE_MEA	26	10512RA1-8	17.0	30.0	19.4
2015	NUS	2015_NE_MEA	27	M11-2024	33.7	NA	21.6
2015	NUS	2015_NE_MEA	28	M12-3312CW	13.3	65.0	21.6
2015	NUS	2015_NE_MEA	29	M12-3301	18.3	NA	21.0
2015	NUS	2015_NE_MEA	30	M12-2036	12.4	NA	17.4
2015	NUS	2015_NE_MEA	31	M12-2031	13.0	NA	15.0
2015	NUS	2015_NE_MEA	32	CA9-72	35.3	33.0	NA
2015	NUS	2015_NE_MEA	33	CA9-76	44.3	NA	14.0
2015	NUS	2015_NE_MEA	34	DH5-15	29.3	29.0	19.4
2015	NUS	2015_NE_MEA	35	CA13-53	21.6	27.0	22.9
2015	NUS	2015_NE_MEA	36	CA13-63	19.0	NA	8.2
2015	NUS	2015_NE_MEA	37	IL10-19464	29.3	29.0	14.8
2015	NUS	2015_NE_MEA	38	IL10-21934	27.7	NA	NA
2015	NUS	2015_NE_MEA	39	IL10-21937	32.3	NA	NA

2015	NUS	2015_NE_MEA	40	IL11-36131	25.3	18.0	NA
2015	NUS	2015_NE_MEA	41	IL11-27667	25.3	NA	8.2
2015	NUS	2015_NE_MEA	42	KY06C-1195-37-2-5	36.0	NA	14.8
2015	NUS	2015_NE_MEA	43	KY06C-1201-18-6-3	20.3	20.0	7.8
2015	NUS	2015_NE_MEA	44	KY06C-1107-7-2-5	24.0	30.0	15.7
2015	NUS	2015_NE_MEA	45	KY06C-2020-10-5-3	24.0	29.0	16.8
2015	NUS	2015_NE_MEA	46	KY06C-2020-11-12-1	17.0	20.0	9.8
2015	NUS	2015_NE_MEA	47	MO122246	14.0	NA	13.7
2015	NUS	2015_NE_MEA	48	MO130203	21.7	NA	14.4
2015	NUS	2015_NE_MEA	49	MO130765	14.3	21.0	10.9
2015	NUS	2015_NE_MEA	50	MO131838	16.7	20.0	9.7
2015	NUS	2015_NE_MEA	51	NE05548	27.0	32.0	18.9
2015	NUS	2015_NE_MEA	52	NE10589	37.0	45.0	NA
2015	NUS	2015_NE_MEA	53	NW13455	29.3	NA	28.7
2015	NUS	2015_NE_MEA	54	NE13511	28.7	31.0	25.8
2015	NUS	2015_NE_MEA	55	NE06545	38.0	NA	24.9
2015	NUS	2015_NE_MEA	56	VA11W-108	45.0	NA	10.0
2015	NUS	2015_NE_MEA	57	VA11W-182	40.0	29.0	16.6
2015	NUS	2015_NE_MEA	58	VA12W-150	31.3	NA	12.7
2015	NUS	2015_NE_MEA	59	VA12FHB-4	27.3	NA	11.3
2015	NUS	2015_NE_MEA	60	VA12FHB-55	46.3	17.0	NA
2015	NUS	2015_ON_RID	1	TRUMAN	66.0	NA	NA
2015	NUS	2015_ON_RID	2	ERNIE	54.0	NA	NA
2015	NUS	2015_ON_RID	3	FREEDOM	55.0	NA	NA
2015	NUS	2015_ON_RID	4	PIONEER2545	79.6	NA	NA
2015	NUS	2015_ON_RID	5	NY99056-161	65.0	NA	NA
2015	NUS	2015_ON_RID	6	NY09067-2-69-1097	59.3	NA	NA
2015	NUS	2015_ON_RID	7	NY05152-818	85.3	NA	NA
2015	NUS	2015_ON_RID	8	NY05152-825	66.6	NA	NA
2015	NUS	2015_ON_RID	9	NY05152-821	58.6	NA	NA
2015	NUS	2015_ON_RID	10	KWS050	79.0	NA	NA
2015	NUS	2015_ON_RID	11	KWS051	66.0	NA	NA
2015	NUS	2015_ON_RID	12	KWS052	34.7	NA	NA
2015	NUS	2015_ON_RID	13	KWS036	79.0	NA	NA
2015	NUS	2015_ON_RID	14	ES12-3030	34.7	NA	NA
2015	NUS	2015_ON_RID	15	ES12-1358	38.0	NA	NA
2015	NUS	2015_ON_RID	16	ES12-1275	49.7	NA	NA
2015	NUS	2015_ON_RID	17	F1014	79.0	NA	NA
2015	NUS	2015_ON_RID	18	E6012	65.0	NA	NA
2015	NUS	2015_ON_RID	19	OH09-207-24	78.3	NA	NA
2015	NUS	2015_ON_RID	20	OH09-281-10	70.3	NA	NA
2015	NUS	2015_ON_RID	21	OH10-200-49	70.3	NA	NA
2015	NUS	2015_ON_RID	22	10641B1-9-11-7	59.7	NA	NA
2015	NUS	2015_ON_RID	23	0762A1-2-8	22.7	NA	NA
2015	NUS	2015_ON_RID	24	08334A1-31	44.3	NA	NA
2015	NUS	2015_ON_RID	25	0566A1-3-1-6	44.3	NA	NA
2015	NUS	2015_ON_RID	26	10512RA1-8	45.7	NA	NA
2015	NUS	2015_ON_RID	27	M11-2024	44.0	NA	NA
2015	NUS	2015_ON_RID	28	M12-3312CW	44.3	NA	NA

2015	NUS	2015_ON_RID	29	M12-3301	32.3	NA	NA
2015	NUS	2015_ON_RID	30	M12-2036	34.7	NA	NA
2015	NUS	2015_ON_RID	31	M12-2031	49.7	NA	NA
2015	NUS	2015_ON_RID	32	CA9-72	70.3	NA	NA
2015	NUS	2015_ON_RID	33	CA9-76	79.0	NA	NA
2015	NUS	2015_ON_RID	34	DH5-15	82.0	NA	NA
2015	NUS	2015_ON_RID	35	CA13-53	70.3	NA	NA
2015	NUS	2015_ON_RID	36	CA13-63	44.3	NA	NA
2015	NUS	2015_ON_RID	37	IL10-19464	48.3	NA	NA
2015	NUS	2015_ON_RID	38	IL10-21934	65.0	NA	NA
2015	NUS	2015_ON_RID	39	IL10-21937	49.7	NA	NA
2015	NUS	2015_ON_RID	40	IL11-36131	38.7	NA	NA
2015	NUS	2015_ON_RID	41	IL11-27667	30.7	NA	NA
2015	NUS	2015_ON_RID	42	KY06C-1195-37-2-5	68.7	NA	NA
2015	NUS	2015_ON_RID	43	KY06C-1201-18-6-3	40.0	NA	NA
2015	NUS	2015_ON_RID	44	KY06C-1107-7-2-5	63.3	NA	NA
2015	NUS	2015_ON_RID	45	KY06C-2020-10-5-3	55.0	NA	NA
2015	NUS	2015_ON_RID	46	KY06C-2020-11-12-1	44.3	NA	NA
2015	NUS	2015_ON_RID	47	MO122246	34.7	NA	NA
2015	NUS	2015_ON_RID	48	MO130203	59.3	NA	NA
2015	NUS	2015_ON_RID	49	MO130765	55.3	NA	NA
2015	NUS	2015_ON_RID	50	MO131838	60.7	NA	NA
2015	NUS	2015_ON_RID	51	NE05548	59.3	NA	NA
2015	NUS	2015_ON_RID	52	NE10589	63.0	NA	NA
2015	NUS	2015_ON_RID	53	NW13455	58.7	NA	NA
2015	NUS	2015_ON_RID	54	NE13511	78.3	NA	NA
2015	NUS	2015_ON_RID	55	NE06545	60.7	NA	NA
2015	NUS	2015_ON_RID	56	VA11W-108	44.0	NA	NA
2015	NUS	2015_ON_RID	57	VA11W-182	60.7	NA	NA
2015	NUS	2015_ON_RID	58	VA12W-150	59.3	NA	NA
2015	NUS	2015_ON_RID	59	VA12FHB-4	22.7	NA	NA
2015	NUS	2015_ON_RID	60	VA12FHB-55	44.3	NA	NA
2015	NUS	2015_IL_URB	1	TRUMAN	33.5	6.7	4.4
2015	NUS	2015_IL_URB	2	ERNIE	32.0	23.3	4.6
2015	NUS	2015_IL_URB	3	FREEDOM	43.0	21.7	2.7
2015	NUS	2015_IL_URB	4	PIONEER2545	80.0	58.3	12.3
2015	NUS	2015_IL_URB	5	NY99056-161	22.7	21.7	4.6
2015	NUS	2015_IL_URB	6	NY09067-2-69-1097	50.4	15.0	2.6
2015	NUS	2015_IL_URB	7	NY05152-818	38.4	11.7	2.9
2015	NUS	2015_IL_URB	8	NY05152-825	41.4	21.7	2.9
2015	NUS	2015_IL_URB	9	NY05152-821	26.5	16.7	3.5
2015	NUS	2015_IL_URB	10	KWS050	35.3	18.3	4.1
2015	NUS	2015_IL_URB	11	KWS051	56.1	23.3	3.8
2015	NUS	2015_IL_URB	12	KWS052	77.6	13.3	2.6
2015	NUS	2015_IL_URB	13	KWS036	75.3	16.7	9.9
2015	NUS	2015_IL_URB	14	ES12-3030	34.3	26.7	2.5
2015	NUS	2015_IL_URB	15	ES12-1358	36.5	8.3	1.5
2015	NUS	2015_IL_URB	16	ES12-1275	30.6	6.7	1.6
2015	NUS	2015_IL_URB	17	F1014	53.6	33.3	7.8

2015	NUS	2015_IL_URB	18	E6012	76.2	13.3	7.5
2015	NUS	2015_IL_URB	19	OH09-207-24	37.0	10.0	2.7
2015	NUS	2015_IL_URB	20	OH09-281-10	36.3	38.3	2.0
2015	NUS	2015_IL_URB	21	OH10-200-49	28.9	5.3	1.5
2015	NUS	2015_IL_URB	22	10641B1-9-11-7	15.7	38.3	7.4
2015	NUS	2015_IL_URB	23	0762A1-2-8	18.1	20.0	0.7
2015	NUS	2015_IL_URB	24	08334A1-31	38.7	6.7	1.7
2015	NUS	2015_IL_URB	25	0566A1-3-1-6	42.3	10.0	2.2
2015	NUS	2015_IL_URB	26	10512RA1-8	16.5	30.0	5.3
2015	NUS	2015_IL_URB	27	M11-2024	61.5	16.7	6.2
2015	NUS	2015_IL_URB	28	M12-3312CW	16.8	11.7	7.5
2015	NUS	2015_IL_URB	29	M12-3301	55.5	8.3	1.8
2015	NUS	2015_IL_URB	30	M12-2036	26.8	13.3	5.6
2015	NUS	2015_IL_URB	31	M12-2031	35.9	15.0	5.8
2015	NUS	2015_IL_URB	32	CA9-72	74.1	18.3	7.2
2015	NUS	2015_IL_URB	33	CA9-76	43.3	11.7	4.7
2015	NUS	2015_IL_URB	34	DH5-15	22.6	18.3	3.0
2015	NUS	2015_IL_URB	35	CA13-53	31.0	16.7	2.6
2015	NUS	2015_IL_URB	36	CA13-63	21.0	11.7	2.5
2015	NUS	2015_IL_URB	37	IL10-19464	34.5	3.7	1.6
2015	NUS	2015_IL_URB	38	IL10-21934	26.1	5.3	1.8
2015	NUS	2015_IL_URB	39	IL10-21937	25.9	5.3	2.8
2015	NUS	2015_IL_URB	40	IL11-36131	23.0	3.7	1.5
2015	NUS	2015_IL_URB	41	IL11-27667	31.8	3.7	1.4
2015	NUS	2015_IL_URB	42	KY06C-1195-37-2-5	42.0	11.7	3.0
2015	NUS	2015_IL_URB	43	KY06C-1201-18-6-3	30.2	10.0	1.8
2015	NUS	2015_IL_URB	44	KY06C-1107-7-2-5	30.6	13.3	2.8
2015	NUS	2015_IL_URB	45	KY06C-2020-10-5-3	80.3	8.3	2.5
2015	NUS	2015_IL_URB	46	KY06C-2020-11-12-1	37.8	8.3	1.0
2015	NUS	2015_IL_URB	47	MO122246	12.1	6.7	1.6
2015	NUS	2015_IL_URB	48	MO130203	44.7	8.3	2.8
2015	NUS	2015_IL_URB	49	MO130765	40.8	8.3	2.3
2015	NUS	2015_IL_URB	50	MO131838	19.0	5.0	1.7
2015	NUS	2015_IL_URB	51	NE05548	52.0	18.3	4.4
2015	NUS	2015_IL_URB	52	NE10589	62.7	30.0	7.6
2015	NUS	2015_IL_URB	53	NW13455	44.4	13.3	9.0
2015	NUS	2015_IL_URB	54	NE13511	43.7	16.7	3.2
2015	NUS	2015_IL_URB	55	NE06545	54.0	21.7	3.2
2015	NUS	2015_IL_URB	56	VA11W-108	70.9	23.3	3.4
2015	NUS	2015_IL_URB	57	VA11W-182	50.8	26.7	5.6
2015	NUS	2015_IL_URB	58	VA12W-150	68.7	15.0	3.5
2015	NUS	2015_IL_URB	59	VA12FHB-4	28.3	8.3	3.1
2015	NUS	2015_IL_URB	60	VA12FHB-55	20.1	5.0	1.9
2015	NUS	2015_OH_WOO	1	TRUMAN	NA	7.2	6.4
2015	NUS	2015_OH_WOO	2	ERNIE	NA	7.5	4.0
2015	NUS	2015_OH_WOO	3	FREEDOM	NA	8.3	4.0

2015	NUS	2015_OH_WOO	4	PIONEER2545	NA	19.2	27.2
2015	NUS	2015_OH_WOO	5	NY99056-161	NA	8.9	24.3
2015	NUS	2015_OH_WOO	6	NY09067-2-69-1097	NA	15.0	8.3
2015	NUS	2015_OH_WOO	7	NY05152-818	NA	17.5	16.6
2015	NUS	2015_OH_WOO	8	NY05152-825	NA	22.5	12.7
2015	NUS	2015_OH_WOO	9	NY05152-821	NA	21.9	22.0
2015	NUS	2015_OH_WOO	10	KWS050	NA	18.9	16.6
2015	NUS	2015_OH_WOO	11	KWS051	NA	18.3	5.8
2015	NUS	2015_OH_WOO	12	KWS052	NA	9.7	6.5
2015	NUS	2015_OH_WOO	13	KWS036	NA	10.8	31.8
2015	NUS	2015_OH_WOO	14	ES12-3030	NA	10.3	5.8
2015	NUS	2015_OH_WOO	15	ES12-1358	NA	8.3	3.0
2015	NUS	2015_OH_WOO	16	ES12-1275	NA	10.0	4.3
2015	NUS	2015_OH_WOO	17	F1014	NA	6.4	21.0
2015	NUS	2015_OH_WOO	18	E6012	NA	7.9	23.3
2015	NUS	2015_OH_WOO	19	OH09-207-24	NA	13.9	7.1
2015	NUS	2015_OH_WOO	20	OH09-281-10	NA	5.4	7.6
2015	NUS	2015_OH_WOO	21	OH10-200-49	NA	4.6	6.1
2015	NUS	2015_OH_WOO	22	10641B1-9-11-7	NA	7.5	5.6
2015	NUS	2015_OH_WOO	23	0762A1-2-8	NA	4.4	3.1
2015	NUS	2015_OH_WOO	24	08334A1-31	NA	9.7	6.0
2015	NUS	2015_OH_WOO	25	0566A1-3-1-6	NA	6.7	4.4
2015	NUS	2015_OH_WOO	26	10512RA1-8	NA	3.9	9.6
2015	NUS	2015_OH_WOO	27	M11-2024	NA	6.1	14.9
2015	NUS	2015_OH_WOO	28	M12-3312CW	NA	5.8	24.5
2015	NUS	2015_OH_WOO	29	M12-3301	NA	4.4	5.6
2015	NUS	2015_OH_WOO	30	M12-2036	NA	4.4	17.4
2015	NUS	2015_OH_WOO	31	M12-2031	NA	5.3	26.7

2015	NUS	2015_OH_WOO	32	CA9-72	NA	14.4	17.1
2015	NUS	2015_OH_WOO	33	CA9-76	NA	11.7	11.3
2015	NUS	2015_OH_WOO	34	DH5-15	NA	10.4	14.7
2015	NUS	2015_OH_WOO	35	CA13-53	NA	22.5	15.1
2015	NUS	2015_OH_WOO	36	CA13-63	NA	17.2	14.0
2015	NUS	2015_OH_WOO	37	IL10-19464	NA	10.1	4.6
2015	NUS	2015_OH_WOO	38	IL10-21934	NA	7.5	5.1
2015	NUS	2015_OH_WOO	39	IL10-21937	NA	7.5	4.8
2015	NUS	2015_OH_WOO	40	IL11-36131	NA	3.8	4.3
2015	NUS	2015_OH_WOO	41	IL11-27667	NA	3.3	2.0
2015	NUS	2015_OH_WOO	42	KY06C-1195-37-2-5	NA	11.9	6.2
2015	NUS	2015_OH_WOO	43	KY06C-1201-18-6-3	NA	11.7	7.7
2015	NUS	2015_OH_WOO	44	KY06C-1107-7-2-5	NA	9.6	6.6
2015	NUS	2015_OH_WOO	45	KY06C-2020-10-5-3	NA	16.7	6.0
2015	NUS	2015_OH_WOO	46	KY06C-2020-11-12-1	NA	5.8	1.4
2015	NUS	2015_OH_WOO	47	MO122246	NA	9.7	3.5
2015	NUS	2015_OH_WOO	48	MO130203	NA	8.6	4.3
2015	NUS	2015_OH_WOO	49	MO130765	NA	9.2	6.1
2015	NUS	2015_OH_WOO	50	MO131838	NA	5.8	5.3
2015	NUS	2015_OH_WOO	51	NE05548	NA	20.8	7.8
2015	NUS	2015_OH_WOO	52	NE10589	NA	23.9	17.6
2015	NUS	2015_OH_WOO	53	NW13455	NA	9.4	14.7
2015	NUS	2015_OH_WOO	54	NE13511	NA	18.6	9.4
2015	NUS	2015_OH_WOO	55	NE06545	NA	10.4	11.1
2015	NUS	2015_OH_WOO	56	VA11W-108	NA	8.6	10.0
2015	NUS	2015_OH_WOO	57	VA11W-182	NA	17.2	10.4
2015	NUS	2015_OH_WOO	58	VA12W-150	NA	14.4	10.7
2015	NUS	2015_OH_WOO	59	VA12FHB-4	NA	7.5	4.0

2015	NUS	2015_OH_WOO	60	VA12FHB-55	NA	11.4	1.7
2016	NUS	2016_IL_CHA	1	TRUMAN	20.0	NA	NA
2016	NUS	2016_IL_CHA	2	FREEDOM	25.0	NA	NA
2016	NUS	2016_IL_CHA	3	ERNIE	60.0	NA	NA
2016	NUS	2016_IL_CHA	4	PIONEER2545	50.0	NA	NA
2016	NUS	2016_IL_CHA	5	OH10-219-65	60.0	NA	NA
2016	NUS	2016_IL_CHA	6	OH09-207-68	30.0	NA	NA
2016	NUS	2016_IL_CHA	7	OH08-206-69	30.0	NA	NA
2016	NUS	2016_IL_CHA	8	OH11-118-18	10.0	NA	NA
2016	NUS	2016_IL_CHA	9	KWS060	80.0	NA	NA
2016	NUS	2016_IL_CHA	10	KWS072	45.0	NA	NA
2016	NUS	2016_IL_CHA	11	KWS074	35.0	NA	NA
2016	NUS	2016_IL_CHA	12	KWS078	20.0	NA	NA
2016	NUS	2016_IL_CHA	13	KY09C-0052-26-12-3	35.0	NA	NA
2016	NUS	2016_IL_CHA	14	X08C-1070-73-18-1	30.0	NA	NA
2016	NUS	2016_IL_CHA	15	X09-0187-112-14-1	30.0	NA	NA
2016	NUS	2016_IL_CHA	16	KY09C-1024-96-1-3	10.0	NA	NA
2016	NUS	2016_IL_CHA	17	KY09C-0601-39-8-1	20.0	NA	NA
2016	NUS	2016_IL_CHA	18	NY05158-833	20.0	NA	NA
2016	NUS	2016_IL_CHA	19	NY05158-864	20.0	NA	NA
2016	NUS	2016_IL_CHA	20	NY05158-841	20.0	NA	NA
2016	NUS	2016_IL_CHA	21	NY05158-859	15.0	NA	NA
2016	NUS	2016_IL_CHA	22	NY99069-352	85.0	NA	NA
2016	NUS	2016_IL_CHA	23	VA14W-6	55.0	NA	NA
2016	NUS	2016_IL_CHA	24	VA10W-21BSR124	90.0	NA	NA
2016	NUS	2016_IL_CHA	25	VA14FHB-22	45.0	NA	NA
2016	NUS	2016_IL_CHA	26	VA14FHB-31	35.0	NA	NA
2016	NUS	2016_IL_CHA	27	MI14R0233	25.0	NA	NA
2016	NUS	2016_IL_CHA	28	MI14R0082	10.0	NA	NA
2016	NUS	2016_IL_CHA	29	MI14W0217	35.0	NA	NA
2016	NUS	2016_IL_CHA	30	MI14R0080	25.0	NA	NA
2016	NUS	2016_IL_CHA	31	MI14R0109	25.0	NA	NA
2016	NUS	2016_IL_CHA	32	0762A1-2-8	10.0	NA	NA
2016	NUS	2016_IL_CHA	33	05247A1-7-3-108-2	25.0	NA	NA
2016	NUS	2016_IL_CHA	34	0566A1-3-1-52	30.0	NA	NA
2016	NUS	2016_IL_CHA	35	05247A1-7-3-120	45.0	NA	NA
2016	NUS	2016_IL_CHA	36	04620A1-1-7-4-17	45.0	NA	NA
2016	NUS	2016_IL_CHA	37	ES14-0937	45.0	NA	NA
2016	NUS	2016_IL_CHA	38	ES14-1398	55.0	NA	NA
2016	NUS	2016_IL_CHA	39	ES14-1860	20.0	NA	NA
2016	NUS	2016_IL_CHA	40	ES14-1847	25.0	NA	NA
2016	NUS	2016_IL_CHA	41	IL10-21934	45.0	NA	NA
2016	NUS	2016_IL_CHA	42	IL10-21937	15.0	NA	NA
2016	NUS	2016_IL_CHA	43	IL11-6543	25.0	NA	NA
2016	NUS	2016_IL_CHA	44	IL11-28222	10.0	NA	NA
2016	NUS	2016_IL_CHA	45	IL12-5110	30.0	NA	NA
2016	NUS	2016_IL_CHA	46	NE13625	30.0	NA	NA
2016	NUS	2016_IL_CHA	47	NE13515	50.0	NA	NA
2016	NUS	2016_IL_CHA	48	NE05548	70.0	NA	NA

2016	NUS	2016_IL_CHA	49	NE13604	15.0	NA	NA
2016	NUS	2016_IL_CHA	50	NI12702W	45.0	NA	NA
2016	NUS	2016_IL_HIG	1	TRUMAN	5.0	NA	NA
2016	NUS	2016_IL_HIG	2	FREEDOM	20.0	NA	NA
2016	NUS	2016_IL_HIG	3	ERNIE	50.0	NA	NA
2016	NUS	2016_IL_HIG	4	PIONEER2545	65.0	NA	NA
2016	NUS	2016_IL_HIG	5	OH10-219-65	12.5	NA	NA
2016	NUS	2016_IL_HIG	6	OH09-207-68	15.0	NA	NA
2016	NUS	2016_IL_HIG	7	OH08-206-69	22.5	NA	NA
2016	NUS	2016_IL_HIG	8	OH11-118-18	25.0	NA	NA
2016	NUS	2016_IL_HIG	9	KWS060	45.0	NA	NA
2016	NUS	2016_IL_HIG	10	KWS072	45.0	NA	NA
2016	NUS	2016_IL_HIG	11	KWS074	45.0	NA	NA
2016	NUS	2016_IL_HIG	12	KWS078	32.5	NA	NA
2016	NUS	2016_IL_HIG	13	KY09C-0052-26-12-3	30.0	NA	NA
2016	NUS	2016_IL_HIG	14	X08C-1070-73-18-1	97.5	NA	NA
2016	NUS	2016_IL_HIG	15	X09-0187-112-14-1	10.5	NA	NA
2016	NUS	2016_IL_HIG	16	KY09C-1024-96-1-3	17.5	NA	NA
2016	NUS	2016_IL_HIG	17	KY09C-0601-39-8-1	22.5	NA	NA
2016	NUS	2016_IL_HIG	18	NY05158-833	12.5	NA	NA
2016	NUS	2016_IL_HIG	19	NY05158-864	10.0	NA	NA
2016	NUS	2016_IL_HIG	20	NY05158-841	27.5	NA	NA
2016	NUS	2016_IL_HIG	21	NY05158-859	15.0	NA	NA
2016	NUS	2016_IL_HIG	22	NY99069-352	45.0	NA	NA
2016	NUS	2016_IL_HIG	23	VA14W-6	27.5	NA	NA
2016	NUS	2016_IL_HIG	24	VA10W-21BSR124	45.0	NA	NA
2016	NUS	2016_IL_HIG	25	VA14FHB-22	27.5	NA	NA
2016	NUS	2016_IL_HIG	26	VA14FHB-31	50.0	NA	NA
2016	NUS	2016_IL_HIG	27	MI14R0233	7.5	NA	NA
2016	NUS	2016_IL_HIG	28	MI14R0082	3.0	NA	NA
2016	NUS	2016_IL_HIG	29	MI14W0217	22.5	NA	NA
2016	NUS	2016_IL_HIG	30	MI14R0080	45.0	NA	NA
2016	NUS	2016_IL_HIG	31	MI14R0109	15.0	NA	NA
2016	NUS	2016_IL_HIG	32	0762A1-2-8	2.5	NA	NA
2016	NUS	2016_IL_HIG	33	05247A1-7-3-108-2	7.5	NA	NA
2016	NUS	2016_IL_HIG	34	0566A1-3-1-52	27.5	NA	NA
2016	NUS	2016_IL_HIG	35	05247A1-7-3-120	12.5	NA	NA
2016	NUS	2016_IL_HIG	36	04620A1-1-7-4-17	25.0	NA	NA
2016	NUS	2016_IL_HIG	37	ES14-0937	20.0	NA	NA
2016	NUS	2016_IL_HIG	38	ES14-1398	27.5	NA	NA
2016	NUS	2016_IL_HIG	39	ES14-1860	22.5	NA	NA
2016	NUS	2016_IL_HIG	40	ES14-1847	45.0	NA	NA
2016	NUS	2016_IL_HIG	41	IL10-21934	45.0	NA	NA
2016	NUS	2016_IL_HIG	42	IL10-21937	27.5	NA	NA
2016	NUS	2016_IL_HIG	43	IL11-6543	35.0	NA	NA
2016	NUS	2016_IL_HIG	44	IL11-28222	10.0	NA	NA
2016	NUS	2016_IL_HIG	45	IL12-5110	10.0	NA	NA
2016	NUS	2016_IL_HIG	46	NE13625	62.5	NA	NA
2016	NUS	2016_IL_HIG	47	NE13515	45.0	NA	NA

2016	NUS	2016_IL_HIG	48	NE05548	30.0	NA	NA
2016	NUS	2016_IL_HIG	49	NE13604	15.0	NA	NA
2016	NUS	2016_IL_HIG	50	NI12702W	17.5	NA	NA
2016	NUS	2016_IL_URB	1	TRUMAN	36.2	15.0	0.8
2016	NUS	2016_IL_URB	2	FREEDOM	44.4	50.0	1.9
2016	NUS	2016_IL_URB	3	ERNIE	55.7	53.3	2.6
2016	NUS	2016_IL_URB	4	PIONEER2545	72.9	80.0	7.1
2016	NUS	2016_IL_URB	5	OH10-219-65	52.5	28.3	1.8
2016	NUS	2016_IL_URB	6	OH09-207-68	22.7	36.7	1.4
2016	NUS	2016_IL_URB	7	OH08-206-69	36.4	53.3	1.2
2016	NUS	2016_IL_URB	8	OH11-118-18	13.3	30.0	0.4
2016	NUS	2016_IL_URB	9	KWS060	55.4	33.3	2.0
2016	NUS	2016_IL_URB	10	KWS072	37.4	23.3	4.5
2016	NUS	2016_IL_URB	11	KWS074	34.3	23.3	0.8
2016	NUS	2016_IL_URB	12	KWS078	48.2	26.7	1.1
2016	NUS	2016_IL_URB	13	KY09C-0052-26-12-3	19.0	6.7	0.8
2016	NUS	2016_IL_URB	14	X08C-1070-73-18-1	13.7	11.7	0.9
2016	NUS	2016_IL_URB	15	X09-0187-112-14-1	42.0	40.0	1.8
2016	NUS	2016_IL_URB	16	KY09C-1024-96-1-3	57.3	26.7	1.3
2016	NUS	2016_IL_URB	17	KY09C-0601-39-8-1	65.4	38.3	0.5
2016	NUS	2016_IL_URB	18	NY05158-833	34.6	23.3	1.2
2016	NUS	2016_IL_URB	19	NY05158-864	51.1	23.3	1.9
2016	NUS	2016_IL_URB	20	NY05158-841	57.9	26.7	1.4
2016	NUS	2016_IL_URB	21	NY05158-859	45.2	30.0	1.5
2016	NUS	2016_IL_URB	22	NY99069-352	79.4	8.3	2.0
2016	NUS	2016_IL_URB	23	VA14W-6	35.7	23.3	3.5
2016	NUS	2016_IL_URB	24	VA10W-21BSR124	65.5	53.3	3.4
2016	NUS	2016_IL_URB	25	VA14FHB-22	77.6	16.7	2.0
2016	NUS	2016_IL_URB	26	VA14FHB-31	33.9	40.0	3.6
2016	NUS	2016_IL_URB	27	MI14R0233	62.5	10.0	1.2
2016	NUS	2016_IL_URB	28	MI14R0082	66.9	20.0	0.2
2016	NUS	2016_IL_URB	29	MI14W0217	47.7	6.7	1.5
2016	NUS	2016_IL_URB	30	MI14R0080	66.5	66.7	2.3
2016	NUS	2016_IL_URB	31	MI14R0109	34.8	20.0	2.8
2016	NUS	2016_IL_URB	32	0762A1-2-8	15.1	16.7	1.3
2016	NUS	2016_IL_URB	33	05247A1-7-3-108-2	21.9	20.0	2.1
2016	NUS	2016_IL_URB	34	0566A1-3-1-52	38.2	33.3	1.6
2016	NUS	2016_IL_URB	35	05247A1-7-3-120	56.6	16.7	1.9
2016	NUS	2016_IL_URB	36	04620A1-1-7-4-17	62.2	13.3	2.0
2016	NUS	2016_IL_URB	37	ES14-0937	45.8	30.0	1.0
2016	NUS	2016_IL_URB	38	ES14-1398	39.5	15.0	1.3
2016	NUS	2016_IL_URB	39	ES14-1860	17.0	6.7	0.5
2016	NUS	2016_IL_URB	40	ES14-1847	58.5	36.7	3.4
2016	NUS	2016_IL_URB	41	IL10-21934	27.6	5.0	0.8
2016	NUS	2016_IL_URB	42	IL10-21937	23.8	15.0	1.0
2016	NUS	2016_IL_URB	43	IL11-6543	36.9	20.0	0.6
2016	NUS	2016_IL_URB	44	IL11-28222	14.4	8.3	0.7
2016	NUS	2016_IL_URB	45	IL12-5110	42.3	15.0	1.1
2016	NUS	2016_IL_URB	46	NE13625	53.0	6.7	1.6

2016	NUS	2016_IL_URB	47	NE13515	68.9	36.7	3.1
2016	NUS	2016_IL_URB	48	NE05548	79.9	40.0	2.0
2016	NUS	2016_IL_URB	49	NE13604	35.7	10.0	0.5
2016	NUS	2016_IL_URB	50	NI12702W	47.9	6.7	0.9
2016	NUS	2016_IN_WLA	1	TRUMAN	23.5	NA	NA
2016	NUS	2016_IN_WLA	2	FREEDOM	NA	NA	NA
2016	NUS	2016_IN_WLA	3	ERNIE	26.2	NA	NA
2016	NUS	2016_IN_WLA	4	PIONEER2545	27.9	NA	NA
2016	NUS	2016_IN_WLA	5	OH10-219-65	18.4	NA	NA
2016	NUS	2016_IN_WLA	6	OH09-207-68	21.7	NA	NA
2016	NUS	2016_IN_WLA	7	OH08-206-69	19.8	NA	NA
2016	NUS	2016_IN_WLA	8	OH11-118-18	14.5	NA	NA
2016	NUS	2016_IN_WLA	9	KWS060	22.9	NA	NA
2016	NUS	2016_IN_WLA	10	KWS072	36.1	NA	NA
2016	NUS	2016_IN_WLA	11	KWS074	14.1	NA	NA
2016	NUS	2016_IN_WLA	12	KWS078	22.7	NA	NA
2016	NUS	2016_IN_WLA	13	KY09C-0052-26-12-3	22.7	NA	NA
2016	NUS	2016_IN_WLA	14	X08C-1070-73-18-1	14.1	NA	NA
2016	NUS	2016_IN_WLA	15	X09-0187-112-14-1	27.7	NA	NA
2016	NUS	2016_IN_WLA	16	KY09C-1024-96-1-3	8.5	NA	NA
2016	NUS	2016_IN_WLA	17	KY09C-0601-39-8-1	15.4	NA	NA
2016	NUS	2016_IN_WLA	18	NY05158-833	30.7	NA	NA
2016	NUS	2016_IN_WLA	19	NY05158-864	21.6	NA	NA
2016	NUS	2016_IN_WLA	20	NY05158-841	21.9	NA	NA
2016	NUS	2016_IN_WLA	21	NY05158-859	28.2	NA	NA
2016	NUS	2016_IN_WLA	22	NY99069-352	21.7	NA	NA
2016	NUS	2016_IN_WLA	23	VA14W-6	21.5	NA	NA
2016	NUS	2016_IN_WLA	24	VA10W-21BSR124	22.2	NA	NA
2016	NUS	2016_IN_WLA	25	VA14FHB-22	24.6	NA	NA
2016	NUS	2016_IN_WLA	26	VA14FHB-31	24.0	NA	NA
2016	NUS	2016_IN_WLA	27	MI14R0233	18.2	NA	NA
2016	NUS	2016_IN_WLA	28	MI14R0082	9.0	NA	NA
2016	NUS	2016_IN_WLA	29	MI14W0217	20.4	NA	NA
2016	NUS	2016_IN_WLA	30	MI14R0080	27.1	NA	NA
2016	NUS	2016_IN_WLA	31	MI14R0109	11.8	NA	NA
2016	NUS	2016_IN_WLA	32	0762A1-2-8	9.8	NA	NA
2016	NUS	2016_IN_WLA	33	05247A1-7-3-108-2	14.6	NA	NA
2016	NUS	2016_IN_WLA	34	0566A1-3-1-52	20.5	NA	NA
2016	NUS	2016_IN_WLA	35	05247A1-7-3-120	24.6	NA	NA
2016	NUS	2016_IN_WLA	36	04620A1-1-7-4-17	15.1	NA	NA
2016	NUS	2016_IN_WLA	37	ES14-0937	20.1	NA	NA
2016	NUS	2016_IN_WLA	38	ES14-1398	22.3	NA	NA
2016	NUS	2016_IN_WLA	39	ES14-1860	19.6	NA	NA
2016	NUS	2016_IN_WLA	40	ES14-1847	20.6	NA	NA
2016	NUS	2016_IN_WLA	41	IL10-21934	15.5	NA	NA
2016	NUS	2016_IN_WLA	42	IL10-21937	9.9	NA	NA
2016	NUS	2016_IN_WLA	43	IL11-6543	18.0	NA	NA
2016	NUS	2016_IN_WLA	44	IL11-28222	15.4	NA	NA
2016	NUS	2016_IN_WLA	45	IL12-5110	16.5	NA	NA

2016	NUS	2016_IN_WLA	46	NE13625	17.6	NA	NA
2016	NUS	2016_IN_WLA	47	NE13515	20.5	NA	NA
2016	NUS	2016_IN_WLA	48	NE05548	32.8	NA	NA
2016	NUS	2016_IN_WLA	49	NE13604	27.2	NA	NA
2016	NUS	2016_IN_WLA	50	NI12702W	28.2	NA	NA
2016	NUS	2016_KY_LEX	1	TRUMAN	10.5	5.0	10.1
2016	NUS	2016_KY_LEX	2	FREEDOM	NA	NA	NA
2016	NUS	2016_KY_LEX	3	ERNIE	18.0	7.0	15.9
2016	NUS	2016_KY_LEX	4	PIONEER2545	32.3	12.8	26.8
2016	NUS	2016_KY_LEX	5	OH10-219-65	19.7	12.0	12.3
2016	NUS	2016_KY_LEX	6	OH09-207-68	14.0	12.3	15.9
2016	NUS	2016_KY_LEX	7	OH08-206-69	22.1	6.7	16.3
2016	NUS	2016_KY_LEX	8	OH11-118-18	9.5	2.7	8.5
2016	NUS	2016_KY_LEX	9	KWS060	22.0	4.6	12.3
2016	NUS	2016_KY_LEX	10	KWS072	28.6	8.9	19.5
2016	NUS	2016_KY_LEX	11	KWS074	20.5	11.2	18.5
2016	NUS	2016_KY_LEX	12	KWS078	26.2	5.1	19.9
2016	NUS	2016_KY_LEX	13	KY09C-0052-26-12-3	13.4	4.9	6.7
2016	NUS	2016_KY_LEX	14	X08C-1070-73-18-1	14.5	3.0	6.9
2016	NUS	2016_KY_LEX	15	X09-0187-112-14-1	16.7	7.7	16.0
2016	NUS	2016_KY_LEX	16	KY09C-1024-96-1-3	16.6	2.7	12.3
2016	NUS	2016_KY_LEX	17	KY09C-0601-39-8-1	15.4	3.5	7.7
2016	NUS	2016_KY_LEX	18	NY05158-833	32.6	13.5	17.9
2016	NUS	2016_KY_LEX	19	NY05158-864	27.8	7.7	18.1
2016	NUS	2016_KY_LEX	20	NY05158-841	22.0	3.1	17.1
2016	NUS	2016_KY_LEX	21	NY05158-859	28.2	5.6	16.5
2016	NUS	2016_KY_LEX	22	NY99069-352	23.6	6.8	38.5
2016	NUS	2016_KY_LEX	23	VA14W-6	27.3	5.6	23.3
2016	NUS	2016_KY_LEX	24	VA10W-21BSR124	29.8	8.9	24.6
2016	NUS	2016_KY_LEX	25	VA14FHB-22	23.9	5.6	11.6
2016	NUS	2016_KY_LEX	26	VA14FHB-31	35.2	6.3	20.4
2016	NUS	2016_KY_LEX	27	MI14R0233	21.4	9.6	18.7
2016	NUS	2016_KY_LEX	28	MI14R0082	15.0	3.3	7.5
2016	NUS	2016_KY_LEX	29	MI14W0217	14.7	4.3	20.8
2016	NUS	2016_KY_LEX	30	MI14R0080	15.4	7.1	15.7
2016	NUS	2016_KY_LEX	31	MI14R0109	18.1	6.3	22.0
2016	NUS	2016_KY_LEX	32	0762A1-2-8	12.0	5.8	6.5
2016	NUS	2016_KY_LEX	33	05247A1-7-3-108-2	16.2	5.2	16.1
2016	NUS	2016_KY_LEX	34	0566A1-3-1-52	17.8	7.6	10.6
2016	NUS	2016_KY_LEX	35	05247A1-7-3-120	16.8	4.3	13.3
2016	NUS	2016_KY_LEX	36	04620A1-1-7-4-17	16.2	4.4	14.4
2016	NUS	2016_KY_LEX	37	ES14-0937	15.7	3.2	7.3
2016	NUS	2016_KY_LEX	38	ES14-1398	18.5	5.3	9.5
2016	NUS	2016_KY_LEX	39	ES14-1860	14.9	2.5	5.7
2016	NUS	2016_KY_LEX	40	ES14-1847	28.3	12.2	21.3
2016	NUS	2016_KY_LEX	41	IL10-21934	17.4	4.5	9.9
2016	NUS	2016_KY_LEX	42	IL10-21937	18.9	5.0	13.7
2016	NUS	2016_KY_LEX	43	IL11-6543	14.6	3.2	7.7
2016	NUS	2016_KY_LEX	44	IL11-28222	11.3	2.1	5.6

2016	NUS	2016_KY_LEX	45	IL12-5110	12.9	2.6	9.9
2016	NUS	2016_KY_LEX	46	NE13625	17.8	5.9	16.3
2016	NUS	2016_KY_LEX	47	NE13515	31.0	16.1	40.8
2016	NUS	2016_KY_LEX	48	NE05548	30.7	18.9	32.1
2016	NUS	2016_KY_LEX	49	NE13604	19.6	10.3	33.7
2016	NUS	2016_KY_LEX	50	NI12702W	20.0	8.6	21.1
2016	NUS	2016_MI_ELA	1	TRUMAN	28.8	NA	NA
2016	NUS	2016_MI_ELA	2	FREEDOM	29.1	NA	NA
2016	NUS	2016_MI_ELA	3	ERNIE	16.9	NA	NA
2016	NUS	2016_MI_ELA	4	PIONEER2545	60.5	NA	NA
2016	NUS	2016_MI_ELA	5	OH10-219-65	37.1	NA	NA
2016	NUS	2016_MI_ELA	6	OH09-207-68	44.3	NA	NA
2016	NUS	2016_MI_ELA	7	OH08-206-69	13.2	NA	NA
2016	NUS	2016_MI_ELA	8	OH11-118-18	30.1	NA	NA
2016	NUS	2016_MI_ELA	9	KWS060	32.2	NA	NA
2016	NUS	2016_MI_ELA	10	KWS072	58.7	NA	NA
2016	NUS	2016_MI_ELA	11	KWS074	47.2	NA	NA
2016	NUS	2016_MI_ELA	12	KWS078	45.0	NA	NA
2016	NUS	2016_MI_ELA	13	KY09C-0052-26-12-3	51.8	NA	NA
2016	NUS	2016_MI_ELA	14	X08C-1070-73-18-1	54.6	NA	NA
2016	NUS	2016_MI_ELA	15	X09-0187-112-14-1	14.0	NA	NA
2016	NUS	2016_MI_ELA	16	KY09C-1024-96-1-3	43.3	NA	NA
2016	NUS	2016_MI_ELA	17	KY09C-0601-39-8-1	37.9	NA	NA
2016	NUS	2016_MI_ELA	18	NY05158-833	47.5	NA	NA
2016	NUS	2016_MI_ELA	19	NY05158-864	71.5	NA	NA
2016	NUS	2016_MI_ELA	20	NY05158-841	55.7	NA	NA
2016	NUS	2016_MI_ELA	21	NY05158-859	52.7	NA	NA
2016	NUS	2016_MI_ELA	22	NY99069-352	17.8	NA	NA
2016	NUS	2016_MI_ELA	23	VA14W-6	46.8	NA	NA
2016	NUS	2016_MI_ELA	24	VA10W-21BSR124	43.9	NA	NA
2016	NUS	2016_MI_ELA	25	VA14FHB-22	36.2	NA	NA
2016	NUS	2016_MI_ELA	26	VA14FHB-31	60.4	NA	NA
2016	NUS	2016_MI_ELA	27	MI14R0233	50.2	NA	NA
2016	NUS	2016_MI_ELA	28	MI14R0082	41.3	NA	NA
2016	NUS	2016_MI_ELA	29	MI14W0217	46.7	NA	NA
2016	NUS	2016_MI_ELA	30	MI14R0080	50.9	NA	NA
2016	NUS	2016_MI_ELA	31	MI14R0109	43.2	NA	NA
2016	NUS	2016_MI_ELA	32	0762A1-2-8	20.2	NA	NA
2016	NUS	2016_MI_ELA	33	05247A1-7-3-108-2	26.6	NA	NA
2016	NUS	2016_MI_ELA	34	0566A1-3-1-52	27.9	NA	NA
2016	NUS	2016_MI_ELA	35	05247A1-7-3-120	41.7	NA	NA
2016	NUS	2016_MI_ELA	36	04620A1-1-7-4-17	9.8	NA	NA
2016	NUS	2016_MI_ELA	37	ES14-0937	30.7	NA	NA
2016	NUS	2016_MI_ELA	38	ES14-1398	43.8	NA	NA
2016	NUS	2016_MI_ELA	39	ES14-1860	40.9	NA	NA
2016	NUS	2016_MI_ELA	40	ES14-1847	43.6	NA	NA
2016	NUS	2016_MI_ELA	41	IL10-21934	20.2	NA	NA
2016	NUS	2016_MI_ELA	42	IL10-21937	50.5	NA	NA
2016	NUS	2016_MI_ELA	43	IL11-6543	29.7	NA	NA

2016	NUS	2016_MI_ELA	44	IL11-28222	31.2	NA	NA
2016	NUS	2016_MI_ELA	45	IL12-5110	30.2	NA	NA
2016	NUS	2016_MI_ELA	46	NE13625	26.2	NA	NA
2016	NUS	2016_MI_ELA	47	NE13515	30.8	NA	NA
2016	NUS	2016_MI_ELA	48	NE05548	35.2	NA	NA
2016	NUS	2016_MI_ELA	49	NE13604	25.2	NA	NA
2016	NUS	2016_MI_ELA	50	NI12702W	20.7	NA	NA
2016	NUS	2016_MO_COL	1	TRUMAN	11.2	22.5	NA
2016	NUS	2016_MO_COL	2	FREEDOM	NA	NA	NA
2016	NUS	2016_MO_COL	3	ERNIE	25.2	30.0	NA
2016	NUS	2016_MO_COL	4	PIONEER2545	29.2	37.5	NA
2016	NUS	2016_MO_COL	5	OH10-219-65	19.9	60.0	NA
2016	NUS	2016_MO_COL	6	OH09-207-68	18.8	27.5	NA
2016	NUS	2016_MO_COL	7	OH08-206-69	16.2	35.0	NA
2016	NUS	2016_MO_COL	8	OH11-118-18	10.4	17.5	NA
2016	NUS	2016_MO_COL	9	KWS060	18.2	37.5	NA
2016	NUS	2016_MO_COL	10	KWS072	24.5	35.0	NA
2016	NUS	2016_MO_COL	11	KWS074	20.2	30.0	NA
2016	NUS	2016_MO_COL	12	KWS078	8.7	17.5	NA
2016	NUS	2016_MO_COL	13	KY09C-0052-26-12-3	11.7	25.0	NA
2016	NUS	2016_MO_COL	14	X08C-1070-73-18-1	14.2	25.0	NA
2016	NUS	2016_MO_COL	15	X09-0187-112-14-1	12.6	22.5	NA
2016	NUS	2016_MO_COL	16	KY09C-1024-96-1-3	19.4	47.5	NA
2016	NUS	2016_MO_COL	17	KY09C-0601-39-8-1	12.2	30.0	NA
2016	NUS	2016_MO_COL	18	NY05158-833	17.4	32.5	NA
2016	NUS	2016_MO_COL	19	NY05158-864	14.0	35.0	NA
2016	NUS	2016_MO_COL	20	NY05158-841	12.6	22.5	NA
2016	NUS	2016_MO_COL	21	NY05158-859	23.5	50.0	NA
2016	NUS	2016_MO_COL	22	NY99069-352	24.1	60.0	NA
2016	NUS	2016_MO_COL	23	VA14W-6	15.3	52.5	NA
2016	NUS	2016_MO_COL	24	VA10W-21BSR124	27.5	57.5	NA

2016	NUS	2016_MO_COL	25	VA14FHB-22	22.8	40.0	NA
2016	NUS	2016_MO_COL	26	VA14FHB-31	15.1	22.5	NA
2016	NUS	2016_MO_COL	27	MI14R0233	11.0	32.5	NA
2016	NUS	2016_MO_COL	28	MI14R0082	10.4	30.0	NA
2016	NUS	2016_MO_COL	29	MI14W0217	11.0	12.5	NA
2016	NUS	2016_MO_COL	30	MI14R0080	16.5	45.0	NA
2016	NUS	2016_MO_COL	31	MI14R0109	12.6	37.5	NA
2016	NUS	2016_MO_COL	32	0762A1-2-8	10.1	20.0	NA
2016	NUS	2016_MO_COL	33	05247A1-7-3-108-2	7.9	15.0	NA
2016	NUS	2016_MO_COL	34	0566A1-3-1-52	7.8	15.0	NA
2016	NUS	2016_MO_COL	35	05247A1-7-3-120	9.2	20.0	NA
2016	NUS	2016_MO_COL	36	04620A1-1-7-4-17	10.5	17.5	NA
2016	NUS	2016_MO_COL	37	ES14-0937	17.2	25.0	NA
2016	NUS	2016_MO_COL	38	ES14-1398	19.0	45.0	NA
2016	NUS	2016_MO_COL	39	ES14-1860	15.3	37.5	NA
2016	NUS	2016_MO_COL	40	ES14-1847	14.5	35.0	NA
2016	NUS	2016_MO_COL	41	IL10-21934	11.0	15.0	NA
2016	NUS	2016_MO_COL	42	IL10-21937	8.0	17.5	NA
2016	NUS	2016_MO_COL	43	IL11-6543	8.4	7.5	NA
2016	NUS	2016_MO_COL	44	IL11-28222	21.0	22.5	NA
2016	NUS	2016_MO_COL	45	IL12-5110	12.5	37.5	NA
2016	NUS	2016_MO_COL	46	NE13625	13.2	15.0	NA
2016	NUS	2016_MO_COL	47	NE13515	16.5	35.0	NA
2016	NUS	2016_MO_COL	48	NE05548	18.8	60.0	NA
2016	NUS	2016_MO_COL	49	NE13604	21.2	25.0	NA
2016	NUS	2016_MO_COL	50	NI12702W	26.1	45.0	NA
2016	NUS	2016_NE_MEA	1	TRUMAN	11.3	22.0	NA
2016	NUS	2016_NE_MEA	2	FREEDOM	15.7	24.0	NA
2016	NUS	2016_NE_MEA	3	ERNIE	19.7	12.0	NA

2016	NUS	2016_NE_MEA	4	PIONEER2545	27.0	28.0	NA
2016	NUS	2016_NE_MEA	5	OH10-219-65	15.0	13.0	NA
2016	NUS	2016_NE_MEA	6	OH09-207-68	10.7	12.0	NA
2016	NUS	2016_NE_MEA	7	OH08-206-69	13.0	16.0	NA
2016	NUS	2016_NE_MEA	8	OH11-118-18	9.0	25.0	NA
2016	NUS	2016_NE_MEA	9	KWS060	12.0	15.0	NA
2016	NUS	2016_NE_MEA	10	KWS072	26.7	14.0	NA
2016	NUS	2016_NE_MEA	11	KWS074	18.3	15.0	NA
2016	NUS	2016_NE_MEA	12	KWS078	14.0	7.0	NA
2016	NUS	2016_NE_MEA	13	KY09C-0052-26-12-3	19.7	19.0	NA
2016	NUS	2016_NE_MEA	14	X08C-1070-73-18-1	16.0	14.0	NA
2016	NUS	2016_NE_MEA	15	X09-0187-112-14-1	27.3	24.0	NA
2016	NUS	2016_NE_MEA	16	KY09C-1024-96-1-3	17.0	10.0	NA
2016	NUS	2016_NE_MEA	17	KY09C-0601-39-8-1	15.7	5.0	NA
2016	NUS	2016_NE_MEA	18	NY05158-833	18.0	6.0	NA
2016	NUS	2016_NE_MEA	19	NY05158-864	21.7	8.0	NA
2016	NUS	2016_NE_MEA	20	NY05158-841	29.3	11.0	NA
2016	NUS	2016_NE_MEA	21	NY05158-859	31.7	12.0	NA
2016	NUS	2016_NE_MEA	22	NY99069-352	20.0	11.0	NA
2016	NUS	2016_NE_MEA	23	VA14W-6	25.7	12.0	NA
2016	NUS	2016_NE_MEA	24	VA10W-21BSR124	24.3	18.0	NA
2016	NUS	2016_NE_MEA	25	VA14FHB-22	17.3	14.0	NA
2016	NUS	2016_NE_MEA	26	VA14FHB-31	14.7	28.0	NA
2016	NUS	2016_NE_MEA	27	MI14R0233	18.7	22.0	NA
2016	NUS	2016_NE_MEA	28	MI14R0082	14.7	29.0	NA
2016	NUS	2016_NE_MEA	29	MI14W0217	11.3	12.0	NA
2016	NUS	2016_NE_MEA	30	MI14R0080	17.0	19.0	NA
2016	NUS	2016_NE_MEA	31	MI14R0109	16.7	17.0	NA
2016	NUS	2016_NE_MEA	32	0762A1-2-8	17.3	25.0	NA
2016	NUS	2016_NE_MEA	33	05247A1-7-3-108-2	14.3	20.0	NA
2016	NUS	2016_NE_MEA	34	0566A1-3-1-52	15.3	19.0	NA
2016	NUS	2016_NE_MEA	35	05247A1-7-3-120	23.3	21.0	NA
2016	NUS	2016_NE_MEA	36	04620A1-1-7-4-17	22.0	21.0	NA
2016	NUS	2016_NE_MEA	37	ES14-0937	15.0	40.0	NA
2016	NUS	2016_NE_MEA	38	ES14-1398	14.0	18.0	NA
2016	NUS	2016_NE_MEA	39	ES14-1860	19.7	26.0	NA
2016	NUS	2016_NE_MEA	40	ES14-1847	22.4	23.0	NA
2016	NUS	2016_NE_MEA	41	IL10-21934	22.7	18.0	NA
2016	NUS	2016_NE_MEA	42	IL10-21937	13.7	10.0	NA
2016	NUS	2016_NE_MEA	43	IL11-6543	18.3	19.0	NA
2016	NUS	2016_NE_MEA	44	IL11-28222	20.3	31.0	NA
2016	NUS	2016_NE_MEA	45	IL12-5110	20.3	26.0	NA
2016	NUS	2016_NE_MEA	46	NE13625	8.7	8.0	NA
2016	NUS	2016_NE_MEA	47	NE13515	18.0	8.0	NA
2016	NUS	2016_NE_MEA	48	NE05548	16.0	18.0	NA
2016	NUS	2016_NE_MEA	49	NE13604	15.0	10.0	NA
2016	NUS	2016_NE_MEA	50	NI12702W	14.0	2.0	NA
2016	NUS	2016_NY_ITH	1	TRUMAN	NA	10.0	NA
2016	NUS	2016_NY_ITH	2	FREEDOM	NA	25.0	NA

2016	NUS	2016_NY_ITH	3	ERNIE	NA	15.0	NA
2016	NUS	2016_NY_ITH	4	PIONEER2545	NA	60.0	NA
2016	NUS	2016_NY_ITH	5	OH10-219-65	NA	10.0	NA
2016	NUS	2016_NY_ITH	6	OH09-207-68	NA	20.0	NA
2016	NUS	2016_NY_ITH	7	OH08-206-69	NA	25.0	NA
2016	NUS	2016_NY_ITH	8	OH11-118-18	NA	25.0	NA
2016	NUS	2016_NY_ITH	9	KWS060	NA	25.0	NA
2016	NUS	2016_NY_ITH	10	KWS072	NA	15.0	NA
2016	NUS	2016_NY_ITH	11	KWS074	NA	25.0	NA
2016	NUS	2016_NY_ITH	12	KWS078	NA	10.0	NA
2016	NUS	2016_NY_ITH	13	KY09C-0052-26-12-3	NA	10.0	NA
2016	NUS	2016_NY_ITH	14	X08C-1070-73-18-1	NA	20.0	NA
2016	NUS	2016_NY_ITH	15	X09-0187-112-14-1	NA	25.0	NA
2016	NUS	2016_NY_ITH	16	KY09C-1024-96-1-3	NA	10.0	NA
2016	NUS	2016_NY_ITH	17	KY09C-0601-39-8-1	NA	15.0	NA
2016	NUS	2016_NY_ITH	18	NY05158-833	NA	5.0	NA
2016	NUS	2016_NY_ITH	19	NY05158-864	NA	10.0	NA
2016	NUS	2016_NY_ITH	20	NY05158-841	NA	5.0	NA
2016	NUS	2016_NY_ITH	21	NY05158-859	NA	10.0	NA
2016	NUS	2016_NY_ITH	22	NY99069-352	NA	30.0	NA
2016	NUS	2016_NY_ITH	23	VA14W-6	NA	40.0	NA
2016	NUS	2016_NY_ITH	24	VA10W-21BSR124	NA	50.0	NA
2016	NUS	2016_NY_ITH	25	VA14FHB-22	NA	25.0	NA
2016	NUS	2016_NY_ITH	26	VA14FHB-31	NA	45.0	NA
2016	NUS	2016_NY_ITH	27	MI14R0233	NA	15.0	NA
2016	NUS	2016_NY_ITH	28	MI14R0082	NA	50.0	NA
2016	NUS	2016_NY_ITH	29	MI14W0217	NA	10.0	NA
2016	NUS	2016_NY_ITH	30	MI14R0080	NA	10.0	NA
2016	NUS	2016_NY_ITH	31	MI14R0109	NA	10.0	NA
2016	NUS	2016_NY_ITH	32	0762A1-2-8	NA	45.0	NA
2016	NUS	2016_NY_ITH	33	05247A1-7-3-108-2	NA	50.0	NA
2016	NUS	2016_NY_ITH	34	0566A1-3-1-52	NA	55.0	NA
2016	NUS	2016_NY_ITH	35	05247A1-7-3-120	NA	40.0	NA
2016	NUS	2016_NY_ITH	36	04620A1-1-7-4-17	NA	25.0	NA
2016	NUS	2016_NY_ITH	37	ES14-0937	NA	15.0	NA
2016	NUS	2016_NY_ITH	38	ES14-1398	NA	25.0	NA
2016	NUS	2016_NY_ITH	39	ES14-1860	NA	10.0	NA
2016	NUS	2016_NY_ITH	40	ES14-1847	NA	65.0	NA
2016	NUS	2016_NY_ITH	41	IL10-21934	NA	5.0	NA
2016	NUS	2016_NY_ITH	42	IL10-21937	NA	5.0	NA
2016	NUS	2016_NY_ITH	43	IL11-6543	NA	5.0	NA
2016	NUS	2016_NY_ITH	44	IL11-28222	NA	0.0	NA
2016	NUS	2016_NY_ITH	45	IL12-5110	NA	5.0	NA
2016	NUS	2016_NY_ITH	46	NE13625	NA	5.0	NA
2016	NUS	2016_NY_ITH	47	NE13515	NA	10.0	NA
2016	NUS	2016_NY_ITH	48	NE05548	NA	10.0	NA
2016	NUS	2016_NY_ITH	49	NE13604	NA	20.0	NA
2016	NUS	2016_NY_ITH	50	NI12702W	NA	10.0	NA
2016	NUS	2016_OH_WOO	1	TRUMAN	NA	15.0	11.9

2016	NUS	2016_OH_WOO	2	FREEDOM	NA	40.0	12.8
2016	NUS	2016_OH_WOO	3	ERNIE	NA	28.0	32.1
2016	NUS	2016_OH_WOO	4	PIONEER2545	NA	58.0	53.7
2016	NUS	2016_OH_WOO	5	OH10-219-65	NA	50.0	28.6
2016	NUS	2016_OH_WOO	6	OH09-207-68	NA	20.0	11.7
2016	NUS	2016_OH_WOO	7	OH08-206-69	NA	30.0	25.2
2016	NUS	2016_OH_WOO	8	OH11-118-18	NA	8.0	6.5
2016	NUS	2016_OH_WOO	9	KWS060	NA	55.0	39.3
2016	NUS	2016_OH_WOO	10	KWS072	NA	20.0	10.2
2016	NUS	2016_OH_WOO	11	KWS074	NA	25.0	16.6
2016	NUS	2016_OH_WOO	12	KWS078	NA	18.0	21.1
2016	NUS	2016_OH_WOO	13	KY09C-0052-26-12-3	NA	8.0	10.1
2016	NUS	2016_OH_WOO	14	X08C-1070-73-18-1	NA	25.0	33.0
2016	NUS	2016_OH_WOO	15	X09-0187-112-14-1	NA	25.0	28.9
2016	NUS	2016_OH_WOO	16	KY09C-1024-96-1-3	NA	10.0	15.4
2016	NUS	2016_OH_WOO	17	KY09C-0601-39-8-1	NA	8.0	14.8
2016	NUS	2016_OH_WOO	18	NY05158-833	NA	15.0	20.5
2016	NUS	2016_OH_WOO	19	NY05158-864	NA	10.0	27.0
2016	NUS	2016_OH_WOO	20	NY05158-841	NA	15.0	16.6
2016	NUS	2016_OH_WOO	21	NY05158-859	NA	10.0	15.0
2016	NUS	2016_OH_WOO	22	NY99069-352	NA	28.0	33.6
2016	NUS	2016_OH_WOO	23	VA14W-6	NA	55.0	24.6
2016	NUS	2016_OH_WOO	24	VA10W-21BSR124	NA	70.0	20.9
2016	NUS	2016_OH_WOO	25	VA14FHB-22	NA	50.0	28.2
2016	NUS	2016_OH_WOO	26	VA14FHB-31	NA	15.0	8.7
2016	NUS	2016_OH_WOO	27	MI14R0233	NA	18.0	19.3
2016	NUS	2016_OH_WOO	28	MI14R0082	NA	5.0	8.0
2016	NUS	2016_OH_WOO	29	MI14W0217	NA	20.0	24.1

2016	NUS	2016_OH_WOO	30	MI14R0080	NA	20.0	13.5
2016	NUS	2016_OH_WOO	31	MI14R0109	NA	18.0	9.7
2016	NUS	2016_OH_WOO	32	0762A1-2-8	NA	15.0	9.7
2016	NUS	2016_OH_WOO	33	05247A1-7-3-108-2	NA	20.0	21.6
2016	NUS	2016_OH_WOO	34	0566A1-3-1-52	NA	18.0	11.6
2016	NUS	2016_OH_WOO	35	05247A1-7-3-120	NA	15.0	23.5
2016	NUS	2016_OH_WOO	36	04620A1-1-7-4-17	NA	30.0	26.1
2016	NUS	2016_OH_WOO	37	ES14-0937	NA	15.0	16.1
2016	NUS	2016_OH_WOO	38	ES14-1398	NA	25.0	15.0
2016	NUS	2016_OH_WOO	39	ES14-1860	NA	15.0	9.5
2016	NUS	2016_OH_WOO	40	ES14-1847	NA	70.0	43.2
2016	NUS	2016_OH_WOO	41	IL10-21934	NA	15.0	13.4
2016	NUS	2016_OH_WOO	42	IL10-21937	NA	25.0	17.3
2016	NUS	2016_OH_WOO	43	IL11-6543	NA	5.0	6.1
2016	NUS	2016_OH_WOO	44	IL11-28222	NA	8.0	8.3
2016	NUS	2016_OH_WOO	45	IL12-5110	NA	8.0	12.1
2016	NUS	2016_OH_WOO	46	NE13625	NA	40.0	27.5
2016	NUS	2016_OH_WOO	47	NE13515	NA	35.0	28.8
2016	NUS	2016_OH_WOO	48	NE05548	NA	50.0	44.5
2016	NUS	2016_OH_WOO	49	NE13604	NA	40.0	25.7
2016	NUS	2016_OH_WOO	50	NI12702W	NA	65.0	NA
2016	NUS	2016_VA_BLA	1	TRUMAN	7.7	22.5	4.8
2016	NUS	2016_VA_BLA	2	FREEDOM	14.3	40.5	9.5
2016	NUS	2016_VA_BLA	3	ERNIE	13.5	46.0	6.0
2016	NUS	2016_VA_BLA	4	PIONEER2545	40.4	52.0	13.0
2016	NUS	2016_VA_BLA	5	OH10-219-65	15.8	31.0	4.8
2016	NUS	2016_VA_BLA	6	OH09-207-68	10.4	47.0	4.8
2016	NUS	2016_VA_BLA	7	OH08-206-69	11.3	37.0	6.8
2016	NUS	2016_VA_BLA	8	OH11-118-18	9.8	21.5	4.9
2016	NUS	2016_VA_BLA	9	KWS060	30.8	51.5	3.0
2016	NUS	2016_VA_BLA	10	KWS072	10.2	48.2	2.7
2016	NUS	2016_VA_BLA	11	KWS074	14.7	31.0	7.8
2016	NUS	2016_VA_BLA	12	KWS078	14.3	23.5	4.0

2016	NUS	2016_VA_BLA	13	KY09C-0052-26-12-3	7.6	34.1	1.2
2016	NUS	2016_VA_BLA	14	X08C-1070-73-18-1	9.1	23.0	3.3
2016	NUS	2016_VA_BLA	15	X09-0187-112-14-1	14.1	38.5	5.6
2016	NUS	2016_VA_BLA	16	KY09C-1024-96-1-3	10.5	27.0	5.0
2016	NUS	2016_VA_BLA	17	KY09C-0601-39-8-1	12.4	15.3	4.0
2016	NUS	2016_VA_BLA	18	NY05158-833	12.8	50.0	5.6
2016	NUS	2016_VA_BLA	19	NY05158-864	18.7	31.3	8.4
2016	NUS	2016_VA_BLA	20	NY05158-841	16.4	42.0	3.0
2016	NUS	2016_VA_BLA	21	NY05158-859	14.6	47.5	7.6
2016	NUS	2016_VA_BLA	22	NY99069-352	22.4	54.5	6.9
2016	NUS	2016_VA_BLA	23	VA14W-6	21.4	44.0	8.6
2016	NUS	2016_VA_BLA	24	VA10W-21BSR124	25.4	41.0	4.1
2016	NUS	2016_VA_BLA	25	VA14FHB-22	16.2	34.0	6.6
2016	NUS	2016_VA_BLA	26	VA14FHB-31	22.3	52.0	7.1
2016	NUS	2016_VA_BLA	27	MI14R0233	16.5	39.0	8.6
2016	NUS	2016_VA_BLA	28	MI14R0082	7.0	19.0	2.9
2016	NUS	2016_VA_BLA	29	MI14W0217	7.7	34.4	5.2
2016	NUS	2016_VA_BLA	30	MI14R0080	14.5	28.8	2.8
2016	NUS	2016_VA_BLA	31	MI14R0109	13.2	32.0	3.3
2016	NUS	2016_VA_BLA	32	0762A1-2-8	8.8	46.0	3.4
2016	NUS	2016_VA_BLA	33	05247A1-7-3-108-2	10.7	45.4	10.8
2016	NUS	2016_VA_BLA	34	0566A1-3-1-52	11.3	75.6	5.3
2016	NUS	2016_VA_BLA	35	05247A1-7-3-120	14.4	41.0	7.3
2016	NUS	2016_VA_BLA	36	04620A1-1-7-4-17	13.8	38.6	6.0
2016	NUS	2016_VA_BLA	37	ES14-0937	10.3	34.0	4.6
2016	NUS	2016_VA_BLA	38	ES14-1398	17.2	41.0	3.3
2016	NUS	2016_VA_BLA	39	ES14-1860	10.4	24.3	1.4
2016	NUS	2016_VA_BLA	40	ES14-1847	34.2	65.5	12.7
2016	NUS	2016_VA_BLA	41	IL10-21934	8.4	31.4	3.3
2016	NUS	2016_VA_BLA	42	IL10-21937	12.9	31.5	4.4
2016	NUS	2016_VA_BLA	43	IL11-6543	8.8	19.5	2.0
2016	NUS	2016_VA_BLA	44	IL11-28222	7.0	18.5	1.7
2016	NUS	2016_VA_BLA	45	IL12-5110	11.7	25.5	3.3
2016	NUS	2016_VA_BLA	46	NE13625	12.2	30.0	6.3
2016	NUS	2016_VA_BLA	47	NE13515	21.1	53.5	7.7
2016	NUS	2016_VA_BLA	48	NE05548	19.9	40.1	8.6
2016	NUS	2016_VA_BLA	49	NE13604	19.6	50.0	12.6
2016	NUS	2016_VA_BLA	50	NI12702W	19.1	61.5	14.2
2017	NUS	2017_OH_WOO	1	TRUMAN	NA	12.0	7.3
2017	NUS	2017_OH_WOO	2	ERNIE	NA	24.0	14.4
2017	NUS	2017_OH_WOO	3	FREEDOM	NA	37.0	18.0
2017	NUS	2017_OH_WOO	4	PIONEER2545	NA	56.0	39.9
2017	NUS	2017_OH_WOO	5	VA11W-108PA	NA	16.0	10.7
2017	NUS	2017_OH_WOO	6	VA09MAS1-12-5-1	NA	15.0	13.7

2017	NUS	2017_OH_WOO	7	VA14FHB-29	NA	15.0	12.8
2017	NUS	2017_OH_WOO	8	VA09MAS8-34-5-2	NA	16.0	12.3
2017	NUS	2017_OH_WOO	9	VA09MAS3-34-2-1	NA	21.0	9.7
2017	NUS	2017_OH_WOO	10	KY09C-0052-26-12-3	NA	11.0	2.4
2017	NUS	2017_OH_WOO	11	X08C-1070-74-20-1	NA	10.0	5.3
2017	NUS	2017_OH_WOO	12	KY09C-1245-100-1-3	NA	12.0	7.0
2017	NUS	2017_OH_WOO	13	KY09C-0267-45-16-3	NA	9.0	5.8
2017	NUS	2017_OH_WOO	14	X08C-1090-51-12-5	NA	12.0	14.3
2017	NUS	2017_OH_WOO	15	OH12-195-22	NA	17.0	13.1
2017	NUS	2017_OH_WOO	16	OH11-118-18	NA	8.0	4.3
2017	NUS	2017_OH_WOO	17	OH12-133-74	NA	17.0	8.9
2017	NUS	2017_OH_WOO	18	OH12-194-24	NA	17.0	14.8
2017	NUS	2017_OH_WOO	19	OH13-16-25	NA	15.0	10.3
2017	NUS	2017_OH_WOO	20	NE14538	NA	17.0	11.7
2017	NUS	2017_OH_WOO	21	NE14606	NA	26.0	21.6
2017	NUS	2017_OH_WOO	22	NE14696	NA	87.0	21.3
2017	NUS	2017_OH_WOO	23	NI12702W	NA	72.0	31.1
2017	NUS	2017_OH_WOO	24	NE15545	NA	73.0	23.3
2017	NUS	2017_OH_WOO	25	KWS095	NA	26.0	7.9
2017	NUS	2017_OH_WOO	26	KWS103	NA	34.0	10.1
2017	NUS	2017_OH_WOO	27	KWS122	NA	17.0	6.1
2017	NUS	2017_OH_WOO	28	KWS127	NA	47.0	15.9
2017	NUS	2017_OH_WOO	29	KWS141	NA	14.0	3.7
2017	NUS	2017_OH_WOO	30	IL09-3264-T2	NA	24.0	6.0
2017	NUS	2017_OH_WOO	31	IL12-21235	NA	11.0	4.1
2017	NUS	2017_OH_WOO	32	IL12-26004	NA	6.0	3.0
2017	NUS	2017_OH_WOO	33	IL13-451	NA	18.0	6.5
2017	NUS	2017_OH_WOO	34	IL13-20616	NA	11.0	5.1

2017	NUS	2017_OH_WOO	35	NY99056-161	NA	18.0	20.0
2017	NUS	2017_OH_WOO	36	NYWHATFORD/7388-39-693	NA	22.0	13.7
2017	NUS	2017_OH_WOO	37	NY09087-15-69-1124	NA	9.0	3.0
2017	NUS	2017_OH_WOO	38	NY01016-AN	NA	17.0	29.8
2017	NUS	2017_OH_WOO	39	NY09125-16-1034	NA	13.0	12.1
2017	NUS	2017_OH_WOO	40	MO140304	NA	13.0	7.5
2017	NUS	2017_OH_WOO	41	MO151323	NA	12.0	3.2
2017	NUS	2017_OH_WOO	42	MO151031	NA	19.0	11.4
2017	NUS	2017_OH_WOO	43	MO151826	NA	11.0	4.4
2017	NUS	2017_OH_WOO	44	MO150133	NA	10.0	3.2
2017	NUS	2017_OH_WOO	45	0566A1-3-1-1-63	NA	22.0	6.8
2017	NUS	2017_OH_WOO	46	04620A1-1-7-4-10	NA	25.0	7.9
2017	NUS	2017_OH_WOO	47	0527A1-9-14-4-3-3	NA	15.0	2.9
2017	NUS	2017_OH_WOO	48	0762A1-2-8	NA	19.0	3.2
2017	NUS	2017_OH_WOO	49	07419A1-16-1-1-16-1-1	NA	70.0	19.3
2017	NUS	2017_OH_WOO	50	LES15-5199	NA	46.0	10.3
2017	NUS	2017_OH_WOO	51	LES15-7011	NA	26.0	4.3
2017	NUS	2017_OH_WOO	52	LES15-5540	NA	57.0	15.1
2017	NUS	2017_OH_WOO	53	LES15-5605	NA	32.0	8.4
2017	NUS	2017_OH_WOO	54	LES15-7004	NA	37.0	9.6
2017	NUS	2017_OH_WOO	55	U6714-B-041	NA	42.0	28.9
2017	NUS	2017_OH_WOO	56	MI14R0008	NA	26.0	7.2
2017	NUS	2017_OH_WOO	57	MI14R0009	NA	17.0	9.7
2017	NUS	2017_OH_WOO	58	MI14R0421	NA	47.0	15.0
2017	NUS	2017_OH_WOO	59	MI14R0267	NA	32.0	9.6
2017	NUS	2017_OH_WOO	60	OH09-207-68	NA	29.0	6.8
2017	NUS	2017_IL_CHA	1	TRUMAN	2.5	NA	NA
2017	NUS	2017_IL_CHA	2	ERNIE	25.0	NA	NA
2017	NUS	2017_IL_CHA	3	FREEDOM	20.0	NA	NA

2017	NUS	2017_IL_CHA	4	PIONEER2545	50.0	NA	NA
2017	NUS	2017_IL_CHA	5	VA11W-108PA	10.0	NA	NA
2017	NUS	2017_IL_CHA	6	VA09MAS1-12-5-1	12.5	NA	NA
2017	NUS	2017_IL_CHA	7	VA14FHB-29	25.0	NA	NA
2017	NUS	2017_IL_CHA	8	VA09MAS8-34-5-2	45.0	NA	NA
2017	NUS	2017_IL_CHA	9	VA09MAS3-34-2-1	12.5	NA	NA
2017	NUS	2017_IL_CHA	10	KY09C-0052-26-12-3	5.0	NA	NA
2017	NUS	2017_IL_CHA	11	X08C-1070-74-20-1	7.5	NA	NA
2017	NUS	2017_IL_CHA	12	KY09C-1245-100-1-3	7.5	NA	NA
2017	NUS	2017_IL_CHA	13	KY09C-0267-45-16-3	5.0	NA	NA
2017	NUS	2017_IL_CHA	14	X08C-1090-51-12-5	15.0	NA	NA
2017	NUS	2017_IL_CHA	15	OH12-195-22	40.0	NA	NA
2017	NUS	2017_IL_CHA	16	OH11-118-18	2.5	NA	NA
2017	NUS	2017_IL_CHA	17	OH12-133-74	25.0	NA	NA
2017	NUS	2017_IL_CHA	18	OH12-194-24	40.0	NA	NA
2017	NUS	2017_IL_CHA	19	OH13-16-25	12.5	NA	NA
2017	NUS	2017_IL_CHA	20	NE14538	22.5	NA	NA
2017	NUS	2017_IL_CHA	21	NE14606	17.5	NA	NA
2017	NUS	2017_IL_CHA	22	NE14696	2.5	NA	NA
2017	NUS	2017_IL_CHA	23	NI12702W	15.0	NA	NA
2017	NUS	2017_IL_CHA	24	NE15545	12.5	NA	NA
2017	NUS	2017_IL_CHA	25	KWS095	22.5	NA	NA
2017	NUS	2017_IL_CHA	26	KWS103	15.0	NA	NA
2017	NUS	2017_IL_CHA	27	KWS122	5.0	NA	NA
2017	NUS	2017_IL_CHA	28	KWS127	42.5	NA	NA
2017	NUS	2017_IL_CHA	29	KWS141	15.0	NA	NA
2017	NUS	2017_IL_CHA	30	IL09-3264-T2	5.0	NA	NA
2017	NUS	2017_IL_CHA	31	IL12-21235	0.0	NA	NA
2017	NUS	2017_IL_CHA	32	IL12-26004	0.0	NA	NA
2017	NUS	2017_IL_CHA	33	IL13-451	2.5	NA	NA
2017	NUS	2017_IL_CHA	34	IL13-20616	0.0	NA	NA
2017	NUS	2017_IL_CHA	35	NY99056-161	22.5	NA	NA
2017	NUS	2017_IL_CHA	36	NYWHATFORD/7388-39-693	6.0	NA	NA
2017	NUS	2017_IL_CHA	37	NY09087-15-69-1124	0.0	NA	NA
2017	NUS	2017_IL_CHA	38	NY01016-AN	20.0	NA	NA
2017	NUS	2017_IL_CHA	39	NY09125-16-1034	12.5	NA	NA
2017	NUS	2017_IL_CHA	40	MO140304	15.0	NA	NA
2017	NUS	2017_IL_CHA	41	MO151323	5.0	NA	NA
2017	NUS	2017_IL_CHA	42	MO151031	12.5	NA	NA
2017	NUS	2017_IL_CHA	43	MO151826	3.5	NA	NA
2017	NUS	2017_IL_CHA	44	MO150133	2.5	NA	NA
2017	NUS	2017_IL_CHA	45	0566A1-3-1-1-63	7.5	NA	NA
2017	NUS	2017_IL_CHA	46	04620A1-1-7-4-10	2.5	NA	NA
2017	NUS	2017_IL_CHA	47	0527A1-9-14-4-3-3	12.5	NA	NA
2017	NUS	2017_IL_CHA	48	0762A1-2-8	2.5	NA	NA
2017	NUS	2017_IL_CHA	49	07419A1-16-1-1-16-1-1	32.5	NA	NA
2017	NUS	2017_IL_CHA	50	LES15-5199	10.0	NA	NA
2017	NUS	2017_IL_CHA	51	LES15-7011	5.0	NA	NA
2017	NUS	2017_IL_CHA	52	LES15-5540	10.0	NA	NA

2017	NUS	2017_IL_CHA	53	LES15-5605	20.0	NA	NA
2017	NUS	2017_IL_CHA	54	LES15-7004	20.0	NA	NA
2017	NUS	2017_IL_CHA	55	U6714-B-041	35.0	NA	NA
2017	NUS	2017_IL_CHA	56	MI14R0008	45.0	NA	NA
2017	NUS	2017_IL_CHA	57	MI14R0009	50.0	NA	NA
2017	NUS	2017_IL_CHA	58	MI14R0421	5.0	NA	NA
2017	NUS	2017_IL_CHA	59	MI14R0267	20.0	NA	NA
2017	NUS	2017_IL_CHA	60	OH09-207-68	25.0	NA	NA
2017	NUS	2017_MI_ELA	1	TRUMAN	19.6	NA	NA
2017	NUS	2017_MI_ELA	2	ERNIE	51.0	NA	NA
2017	NUS	2017_MI_ELA	3	FREEDOM	35.1	NA	NA
2017	NUS	2017_MI_ELA	4	PIONEER2545	52.1	NA	NA
2017	NUS	2017_MI_ELA	5	VA11W-108PA	43.3	NA	NA
2017	NUS	2017_MI_ELA	6	VA09MAS1-12-5-1	25.2	NA	NA
2017	NUS	2017_MI_ELA	7	VA14FHB-29	28.3	NA	NA
2017	NUS	2017_MI_ELA	8	VA09MAS8-34-5-2	53.6	NA	NA
2017	NUS	2017_MI_ELA	9	VA09MAS3-34-2-1	54.4	NA	NA
2017	NUS	2017_MI_ELA	10	KY09C-0052-26-12-3	52.0	NA	NA
2017	NUS	2017_MI_ELA	11	X08C-1070-74-20-1	46.0	NA	NA
2017	NUS	2017_MI_ELA	12	KY09C-1245-100-1-3	42.5	NA	NA
2017	NUS	2017_MI_ELA	13	KY09C-0267-45-16-3	24.7	NA	NA
2017	NUS	2017_MI_ELA	14	X08C-1090-51-12-5	48.7	NA	NA
2017	NUS	2017_MI_ELA	15	OH12-195-22	44.8	NA	NA
2017	NUS	2017_MI_ELA	16	OH11-118-18	26.6	NA	NA
2017	NUS	2017_MI_ELA	17	OH12-133-74	28.6	NA	NA
2017	NUS	2017_MI_ELA	18	OH12-194-24	50.9	NA	NA
2017	NUS	2017_MI_ELA	19	OH13-16-25	58.8	NA	NA
2017	NUS	2017_MI_ELA	20	NE14538	26.7	NA	NA
2017	NUS	2017_MI_ELA	21	NE14606	32.0	NA	NA
2017	NUS	2017_MI_ELA	22	NE14696	27.8	NA	NA
2017	NUS	2017_MI_ELA	23	NI12702W	32.4	NA	NA
2017	NUS	2017_MI_ELA	24	NE15545	60.5	NA	NA
2017	NUS	2017_MI_ELA	25	KWS095	38.3	NA	NA
2017	NUS	2017_MI_ELA	26	KWS103	59.6	NA	NA
2017	NUS	2017_MI_ELA	27	KWS122	NA	NA	NA
2017	NUS	2017_MI_ELA	28	KWS127	NA	NA	NA
2017	NUS	2017_MI_ELA	29	KWS141	NA	NA	NA
2017	NUS	2017_MI_ELA	30	IL09-3264-T2	54.5	NA	NA
2017	NUS	2017_MI_ELA	31	IL12-21235	53.7	NA	NA
2017	NUS	2017_MI_ELA	32	IL12-26004	68.9	NA	NA
2017	NUS	2017_MI_ELA	33	IL13-451	43.8	NA	NA
2017	NUS	2017_MI_ELA	34	IL13-20616	46.1	NA	NA
2017	NUS	2017_MI_ELA	35	NY99056-161	22.9	NA	NA
2017	NUS	2017_MI_ELA	36	NYWHATFORD/7388-39-693	15.5	NA	NA
2017	NUS	2017_MI_ELA	37	NY09087-15-69-1124	14.2	NA	NA
2017	NUS	2017_MI_ELA	38	NY01016-AN	43.7	NA	NA
2017	NUS	2017_MI_ELA	39	NY09125-16-1034	52.8	NA	NA
2017	NUS	2017_MI_ELA	40	MO140304	26.6	NA	NA
2017	NUS	2017_MI_ELA	41	MO151323	32.0	NA	NA

2017	NUS	2017_MI_ELA	42	MO151031	53.1	NA	NA
2017	NUS	2017_MI_ELA	43	MO151826	41.9	NA	NA
2017	NUS	2017_MI_ELA	44	MO150133	32.2	NA	NA
2017	NUS	2017_MI_ELA	45	0566A1-3-1-1-63	53.2	NA	NA
2017	NUS	2017_MI_ELA	46	04620A1-1-7-4-10	36.7	NA	NA
2017	NUS	2017_MI_ELA	47	0527A1-9-14-4-3-3	41.0	NA	NA
2017	NUS	2017_MI_ELA	48	0762A1-2-8	21.6	NA	NA
2017	NUS	2017_MI_ELA	49	07419A1-16-1-1-16-1-1	43.8	NA	NA
2017	NUS	2017_MI_ELA	50	LES15-5199	45.2	NA	NA
2017	NUS	2017_MI_ELA	51	LES15-7011	57.1	NA	NA
2017	NUS	2017_MI_ELA	52	LES15-5540	50.0	NA	NA
2017	NUS	2017_MI_ELA	53	LES15-5605	61.2	NA	NA
2017	NUS	2017_MI_ELA	54	LES15-7004	64.5	NA	NA
2017	NUS	2017_MI_ELA	55	U6714-B-041	60.2	NA	NA
2017	NUS	2017_MI_ELA	56	MI14R0008	51.3	NA	NA
2017	NUS	2017_MI_ELA	57	MI14R0009	44.2	NA	NA
2017	NUS	2017_MI_ELA	58	MI14R0421	41.9	NA	NA
2017	NUS	2017_MI_ELA	59	MI14R0267	31.8	NA	NA
2017	NUS	2017_MI_ELA	60	OH09-207-68	40.2	NA	NA
2017	NUS	2017_VA_MTH	1	TRUMAN	10.0	31.5	0.8
2017	NUS	2017_VA_MTH	2	ERNIE	17.1	21.0	1.4
2017	NUS	2017_VA_MTH	3	FREEDOM	22.5	38.5	1.4
2017	NUS	2017_VA_MTH	4	PIONEER2545	39.9	34.5	5.9
2017	NUS	2017_VA_MTH	5	VA11W-108PA	33.7	38.5	2.1
2017	NUS	2017_VA_MTH	6	VA09MAS1-12-5-1	37.6	17.0	1.6
2017	NUS	2017_VA_MTH	7	VA14FHB-29	28.8	26.5	0.9
2017	NUS	2017_VA_MTH	8	VA09MAS8-34-5-2	36.2	37.0	3.2
2017	NUS	2017_VA_MTH	9	VA09MAS3-34-2-1	35.0	21.0	1.7
2017	NUS	2017_VA_MTH	10	KY09C-0052-26-12-3	17.7	17.5	1.0
2017	NUS	2017_VA_MTH	11	X08C-1070-74-20-1	18.7	22.5	0.9
2017	NUS	2017_VA_MTH	12	KY09C-1245-100-1-3	31.7	17.0	1.0
2017	NUS	2017_VA_MTH	13	KY09C-0267-45-16-3	11.7	18.0	1.4
2017	NUS	2017_VA_MTH	14	X08C-1090-51-12-5	33.8	26.5	1.4
2017	NUS	2017_VA_MTH	15	OH12-195-22	24.4	16.5	1.5
2017	NUS	2017_VA_MTH	16	OH11-118-18	8.6	44.5	1.0
2017	NUS	2017_VA_MTH	17	OH12-133-74	29.6	47.5	1.7

2017	NUS	2017_VA_MTH	18	OH12-194-24	31.8	26.5	1.7
2017	NUS	2017_VA_MTH	19	OH13-16-25	45.4	26.5	1.8
2017	NUS	2017_VA_MTH	20	NE14538	10.6	41.0	1.5
2017	NUS	2017_VA_MTH	21	NE14606	11.6	57.0	4.9
2017	NUS	2017_VA_MTH	22	NE14696	9.5	41.5	2.6
2017	NUS	2017_VA_MTH	23	NI12702W	13.9	53.0	4.0
2017	NUS	2017_VA_MTH	24	NE15545	18.7	43.5	3.7
2017	NUS	2017_VA_MTH	25	KWS095	26.2	32.0	1.5
2017	NUS	2017_VA_MTH	26	KWS103	37.8	27.5	2.2
2017	NUS	2017_VA_MTH	27	KWS122	22.4	26.5	0.6
2017	NUS	2017_VA_MTH	28	KWS127	33.0	23.0	3.0
2017	NUS	2017_VA_MTH	29	KWS141	15.8	27.5	1.3
2017	NUS	2017_VA_MTH	30	IL09-3264-T2	34.2	32.5	1.1
2017	NUS	2017_VA_MTH	31	IL12-21235	19.0	24.5	1.0
2017	NUS	2017_VA_MTH	32	IL12-26004	7.4	19.5	0.6
2017	NUS	2017_VA_MTH	33	IL13-451	16.0	19.5	0.2
2017	NUS	2017_VA_MTH	34	IL13-20616	9.2	27.0	0.6
2017	NUS	2017_VA_MTH	35	NY99056-161	18.3	53.0	5.5
2017	NUS	2017_VA_MTH	36	NYWHATFORD/7388-39-693	10.3	51.0	2.2
2017	NUS	2017_VA_MTH	37	NY09087-15-69-1124	8.5	25.0	0.6
2017	NUS	2017_VA_MTH	38	NY01016-AN	27.3	31.5	4.2
2017	NUS	2017_VA_MTH	39	NY09125-16-1034	15.4	46.0	1.9
2017	NUS	2017_VA_MTH	40	MO140304	10.0	14.5	1.3
2017	NUS	2017_VA_MTH	41	MO151323	7.6	21.5	0.8
2017	NUS	2017_VA_MTH	42	MO151031	17.5	20.0	0.9
2017	NUS	2017_VA_MTH	43	MO151826	11.7	20.5	0.7
2017	NUS	2017_VA_MTH	44	MO150133	13.8	20.0	0.3
2017	NUS	2017_VA_MTH	45	0566A1-3-1-1-63	27.5	20.0	1.9

2017	NUS	2017_VA_MTH	46	04620A1-1-7-4-10	16.9	27.5	1.7
2017	NUS	2017_VA_MTH	47	0527A1-9-14-4-3-3	15.8	18.5	0.6
2017	NUS	2017_VA_MTH	48	0762A1-2-8	6.8	22.5	0.4
2017	NUS	2017_VA_MTH	49	07419A1-16-1-1-16-1-1	34.4	28.5	2.4
2017	NUS	2017_VA_MTH	50	LES15-5199	28.5	39.0	2.3
2017	NUS	2017_VA_MTH	51	LES15-7011	26.9	26.5	1.1
2017	NUS	2017_VA_MTH	52	LES15-5540	24.4	40.0	1.6
2017	NUS	2017_VA_MTH	53	LES15-5605	41.4	25.5	1.1
2017	NUS	2017_VA_MTH	54	LES15-7004	17.8	13.5	0.9
2017	NUS	2017_VA_MTH	55	U6714-B-041	15.4	44.0	7.7
2017	NUS	2017_VA_MTH	56	MI14R0008	51.0	26.0	2.4
2017	NUS	2017_VA_MTH	57	MI14R0009	62.7	23.5	2.9
2017	NUS	2017_VA_MTH	58	MI14R0421	24.1	35.0	1.2
2017	NUS	2017_VA_MTH	59	MI14R0267	21.1	20.5	2.3
2017	NUS	2017_VA_MTH	60	OH09-207-68	27.0	32.5	1.3
2017	NUS	2017_KY_LEX	1	TRUMAN	32.0	10.0	5.5
2017	NUS	2017_KY_LEX	2	ERNIE	49.7	42.5	15.5
2017	NUS	2017_KY_LEX	3	FREEDOM	42.2	65.0	23.1
2017	NUS	2017_KY_LEX	4	PIONEER2545	43.7	65.0	26.9
2017	NUS	2017_KY_LEX	5	VA11W-108PA	25.8	60.0	21.3
2017	NUS	2017_KY_LEX	6	VA09MAS1-12-5-1	26.0	20.0	15.1
2017	NUS	2017_KY_LEX	7	VA14FHB-29	45.3	52.5	21.8
2017	NUS	2017_KY_LEX	8	VA09MAS8-34-5-2	17.8	47.5	17.3
2017	NUS	2017_KY_LEX	9	VA09MAS3-34-2-1	21.9	45.0	20.1
2017	NUS	2017_KY_LEX	10	KY09C-0052-26-12-3	26.0	17.5	9.5
2017	NUS	2017_KY_LEX	11	X08C-1070-74-20-1	31.8	12.5	7.2
2017	NUS	2017_KY_LEX	12	KY09C-1245-100-1-3	36.4	40.0	11.3
2017	NUS	2017_KY_LEX	13	KY09C-0267-45-16-3	35.9	27.5	14.1
2017	NUS	2017_KY_LEX	14	X08C-1090-51-12-5	43.8	30.0	13.0
2017	NUS	2017_KY_LEX	15	OH12-195-22	35.8	37.5	15.6
2017	NUS	2017_KY_LEX	16	OH11-118-18	35.9	7.5	3.4
2017	NUS	2017_KY_LEX	17	OH12-133-74	35.4	30.0	9.8
2017	NUS	2017_KY_LEX	18	OH12-194-24	38.8	47.5	22.3
2017	NUS	2017_KY_LEX	19	OH13-16-25	23.1	37.5	15.3
2017	NUS	2017_KY_LEX	20	NE14538	25.7	55.0	16.6
2017	NUS	2017_KY_LEX	21	NE14606	35.5	37.5	12.7
2017	NUS	2017_KY_LEX	22	NE14696	52.8	32.5	17.7
2017	NUS	2017_KY_LEX	23	NI12702W	26.8	45.0	15.9

2017	NUS	2017_KY_LEX	24	NE15545	28.2	42.5	20.2
2017	NUS	2017_KY_LEX	25	KWS095	39.2	35.0	8.8
2017	NUS	2017_KY_LEX	26	KWS103	30.5	57.5	12.8
2017	NUS	2017_KY_LEX	27	KWS122	36.7	20.0	10.4
2017	NUS	2017_KY_LEX	28	KWS127	40.5	65.0	25.2
2017	NUS	2017_KY_LEX	29	KWS141	45.5	42.5	20.1
2017	NUS	2017_KY_LEX	30	IL09-3264-T2	47.5	32.5	9.4
2017	NUS	2017_KY_LEX	31	IL12-21235	29.2	12.5	12.0
2017	NUS	2017_KY_LEX	32	IL12-26004	36.1	7.5	7.3
2017	NUS	2017_KY_LEX	33	IL13-451	33.8	40.0	10.3
2017	NUS	2017_KY_LEX	34	IL13-20616	36.4	25.0	9.5
2017	NUS	2017_KY_LEX	35	NY99056-161	32.7	17.5	14.2
2017	NUS	2017_KY_LEX	36	NYWHATFORD/7388-39-693	29.2	7.5	6.1
2017	NUS	2017_KY_LEX	37	NY09087-15-69-1124	21.2	12.5	4.2
2017	NUS	2017_KY_LEX	38	NY01016-AN	25.3	37.5	24.2
2017	NUS	2017_KY_LEX	39	NY09125-16-1034	41.2	37.5	19.5
2017	NUS	2017_KY_LEX	40	MO140304	40.9	22.5	14.6
2017	NUS	2017_KY_LEX	41	MO151323	44.7	12.5	7.4
2017	NUS	2017_KY_LEX	42	MO151031	35.4	42.5	14.8
2017	NUS	2017_KY_LEX	43	MO151826	29.0	12.5	11.0
2017	NUS	2017_KY_LEX	44	MO150133	35.1	12.5	5.7
2017	NUS	2017_KY_LEX	45	0566A1-3-1-1-63	28.7	32.5	14.0
2017	NUS	2017_KY_LEX	46	04620A1-1-7-4-10	49.3	37.5	14.4
2017	NUS	2017_KY_LEX	47	0527A1-9-14-4-3-3	39.0	42.5	7.7
2017	NUS	2017_KY_LEX	48	0762A1-2-8	31.6	20.0	9.6
2017	NUS	2017_KY_LEX	49	07419A1-16-1-1-16-1-1	48.4	57.5	18.3
2017	NUS	2017_KY_LEX	50	LES15-5199	37.4	65.0	20.4
2017	NUS	2017_KY_LEX	51	LES15-7011	26.7	32.5	16.9
2017	NUS	2017_KY_LEX	52	LES15-5540	47.6	37.5	14.4
2017	NUS	2017_KY_LEX	53	LES15-5605	35.8	40.0	11.5
2017	NUS	2017_KY_LEX	54	LES15-7004	48.2	37.5	16.3
2017	NUS	2017_KY_LEX	55	U6714-B-041	24.7	77.5	47.1
2017	NUS	2017_KY_LEX	56	MI14R0008	33.8	25.0	11.4
2017	NUS	2017_KY_LEX	57	MI14R0009	33.9	17.5	9.4
2017	NUS	2017_KY_LEX	58	MI14R0421	35.1	47.5	12.9
2017	NUS	2017_KY_LEX	59	MI14R0267	24.9	15.0	15.3
2017	NUS	2017_KY_LEX	60	OH09-207-68	37.4	35.0	13.9
2017	NUS	2017_NY_ITH	1	TRUMAN	6.4	15.0	1.9
2017	NUS	2017_NY_ITH	2	ERNIE	10.2	50.0	9.5
2017	NUS	2017_NY_ITH	3	FREEDOM	10.0	60.0	6.8
2017	NUS	2017_NY_ITH	4	PIONEER2545	19.0	90.0	25.9
2017	NUS	2017_NY_ITH	5	VA11W-108PA	13.4	55.0	8.7
2017	NUS	2017_NY_ITH	6	VA09MAS1-12-5-1	14.6	30.0	7.7
2017	NUS	2017_NY_ITH	7	VA14FHB-29	15.8	50.0	10.6
2017	NUS	2017_NY_ITH	8	VA09MAS8-34-5-2	12.0	90.0	11.5
2017	NUS	2017_NY_ITH	9	VA09MAS3-34-2-1	11.4	40.0	9.6
2017	NUS	2017_NY_ITH	10	KY09C-0052-26-12-3	9.6	10.0	3.4
2017	NUS	2017_NY_ITH	11	X08C-1070-74-20-1	8.8	45.0	8.5
2017	NUS	2017_NY_ITH	12	KY09C-1245-100-1-3	10.6	40.0	5.2

2017	NUS	2017_NY_ITH	13	KY09C-0267-45-16-3	6.8	30.0	7.3
2017	NUS	2017_NY_ITH	14	X08C-1090-51-12-5	7.8	25.0	5.5
2017	NUS	2017_NY_ITH	15	OH12-195-22	11.0	75.0	9.7
2017	NUS	2017_NY_ITH	16	OH11-118-18	1.2	10.0	2.4
2017	NUS	2017_NY_ITH	17	OH12-133-74	11.0	65.0	5.9
2017	NUS	2017_NY_ITH	18	OH12-194-24	20.8	80.0	12.1
2017	NUS	2017_NY_ITH	19	OH13-16-25	10.0	55.0	14.3
2017	NUS	2017_NY_ITH	20	NE14538	10.0	55.0	11.2
2017	NUS	2017_NY_ITH	21	NE14606	11.4	40.0	11.7
2017	NUS	2017_NY_ITH	22	NE14696	11.4	45.0	7.9
2017	NUS	2017_NY_ITH	23	NI12702W	6.4	25.0	10.6
2017	NUS	2017_NY_ITH	24	NE15545	8.0	40.0	4.6
2017	NUS	2017_NY_ITH	25	KWS095	14.2	45.0	6.7
2017	NUS	2017_NY_ITH	26	KWS103	20.6	45.0	9.4
2017	NUS	2017_NY_ITH	27	KWS122	13.6	20.0	4.6
2017	NUS	2017_NY_ITH	28	KWS127	10.6	55.0	12.4
2017	NUS	2017_NY_ITH	29	KWS141	8.0	30.0	6.2
2017	NUS	2017_NY_ITH	30	IL09-3264-T2	9.2	30.0	5.8
2017	NUS	2017_NY_ITH	31	IL12-21235	11.2	10.0	4.6
2017	NUS	2017_NY_ITH	32	IL12-26004	11.4	35.0	5.2
2017	NUS	2017_NY_ITH	33	IL13-451	7.8	15.0	2.2
2017	NUS	2017_NY_ITH	34	IL13-20616	9.0	20.0	2.5
2017	NUS	2017_NY_ITH	35	NY99056-161	13.4	25.0	8.1
2017	NUS	2017_NY_ITH	36	NYWHATFORD/7388-39-693	7.0	35.0	15.2
2017	NUS	2017_NY_ITH	37	NY09087-15-69-1124	6.4	10.0	1.9
2017	NUS	2017_NY_ITH	38	NY01016-AN	17.0	60.0	25.4
2017	NUS	2017_NY_ITH	39	NY09125-16-1034	8.0	50.0	9.4
2017	NUS	2017_NY_ITH	40	MO140304	11.2	25.0	4.8
2017	NUS	2017_NY_ITH	41	MO151323	4.8	10.0	1.7
2017	NUS	2017_NY_ITH	42	MO151031	13.0	25.0	5.2
2017	NUS	2017_NY_ITH	43	MO151826	5.8	20.0	3.1
2017	NUS	2017_NY_ITH	44	MO150133	7.6	10.0	2.8
2017	NUS	2017_NY_ITH	45	0566A1-3-1-1-63	12.0	90.0	12.0
2017	NUS	2017_NY_ITH	46	04620A1-1-7-4-10	8.2	20.0	5.0
2017	NUS	2017_NY_ITH	47	0527A1-9-14-4-3-3	10.8	55.0	2.8
2017	NUS	2017_NY_ITH	48	0762A1-2-8	6.0	50.0	3.3
2017	NUS	2017_NY_ITH	49	07419A1-16-1-1-16-1-1	16.0	80.0	25.5
2017	NUS	2017_NY_ITH	50	LES15-5199	10.2	55.0	8.7
2017	NUS	2017_NY_ITH	51	LES15-7011	7.2	30.0	3.4
2017	NUS	2017_NY_ITH	52	LES15-5540	10.4	25.0	6.7
2017	NUS	2017_NY_ITH	53	LES15-5605	15.8	35.0	5.2
2017	NUS	2017_NY_ITH	54	LES15-7004	13.4	50.0	6.7
2017	NUS	2017_NY_ITH	55	U6714-B-041	11.4	65.0	19.4
2017	NUS	2017_NY_ITH	56	MI14R0008	13.6	60.0	17.2
2017	NUS	2017_NY_ITH	57	MI14R0009	20.8	60.0	16.9
2017	NUS	2017_NY_ITH	58	MI14R0421	10.6	40.0	7.7
2017	NUS	2017_NY_ITH	59	MI14R0267	11.8	35.0	9.2
2017	NUS	2017_NY_ITH	60	OH09-207-68	10.6	50.0	9.4

2017	NUS	2017_MO_COL	1	TRUMAN	7.9	7.5	NA
2017	NUS	2017_MO_COL	2	ERNIE	9.9	12.5	NA
2017	NUS	2017_MO_COL	3	FREEDOM	20.0	7.5	NA
2017	NUS	2017_MO_COL	4	PIONEER2545	14.3	7.5	NA
2017	NUS	2017_MO_COL	5	VA11W-108PA	4.8	15.0	NA
2017	NUS	2017_MO_COL	6	VA09MAS1-12-5-1	8.8	7.5	NA
2017	NUS	2017_MO_COL	7	VA14FHB-29	7.6	10.0	NA
2017	NUS	2017_MO_COL	8	VA09MAS8-34-5-2	12.7	20.0	NA
2017	NUS	2017_MO_COL	9	VA09MAS3-34-2-1	12.5	17.5	NA
2017	NUS	2017_MO_COL	10	KY09C-0052-26-12-3	13.8	12.5	NA
2017	NUS	2017_MO_COL	11	X08C-1070-74-20-1	10.8	7.5	NA
2017	NUS	2017_MO_COL	12	KY09C-1245-100-1-3	11.3	10.0	NA
2017	NUS	2017_MO_COL	13	KY09C-0267-45-16-3	3.2	20.0	NA
2017	NUS	2017_MO_COL	14	X08C-1090-51-12-5	3.7	5.0	NA
2017	NUS	2017_MO_COL	15	OH12-195-22	10.2	5.0	NA
2017	NUS	2017_MO_COL	16	OH11-118-18	12.4	10.0	NA
2017	NUS	2017_MO_COL	17	OH12-133-74	5.4	15.0	NA
2017	NUS	2017_MO_COL	18	OH12-194-24	7.5	15.0	NA
2017	NUS	2017_MO_COL	19	OH13-16-25	7.5	7.5	NA
2017	NUS	2017_MO_COL	20	NE14538	9.5	10.0	NA
2017	NUS	2017_MO_COL	21	NE14606	8.4	5.0	NA
2017	NUS	2017_MO_COL	22	NE14696	10.4	10.0	NA
2017	NUS	2017_MO_COL	23	NI12702W	9.6	10.0	NA
2017	NUS	2017_MO_COL	24	NE15545	6.8	10.0	NA
2017	NUS	2017_MO_COL	25	KWS095	7.6	20.0	NA
2017	NUS	2017_MO_COL	26	KWS103	7.3	5.0	NA
2017	NUS	2017_MO_COL	27	KWS122	12.5	7.5	NA
2017	NUS	2017_MO_COL	28	KWS127	7.6	17.5	NA

2017	NUS	2017_MO_COL	29	KWS141	12.8	5.0	NA
2017	NUS	2017_MO_COL	30	IL09-3264-T2	7.7	12.5	NA
2017	NUS	2017_MO_COL	31	IL12-21235	7.5	5.0	NA
2017	NUS	2017_MO_COL	32	IL12-26004	10.4	10.0	NA
2017	NUS	2017_MO_COL	33	IL13-451	14.0	5.0	NA
2017	NUS	2017_MO_COL	34	IL13-20616	8.9	10.0	NA
2017	NUS	2017_MO_COL	35	NY99056-161	7.4	5.0	NA
2017	NUS	2017_MO_COL	36	NYWHATFORD/7388-39-693	8.2	5.0	NA
2017	NUS	2017_MO_COL	37	NY09087-15-69-1124	6.1	7.5	NA
2017	NUS	2017_MO_COL	38	NY01016-AN	8.9	5.0	NA
2017	NUS	2017_MO_COL	39	NY09125-16-1034	6.2	15.0	NA
2017	NUS	2017_MO_COL	40	MO140304	9.6	12.5	NA
2017	NUS	2017_MO_COL	41	MO151323	10.0	7.5	NA
2017	NUS	2017_MO_COL	42	MO151031	6.9	5.0	NA
2017	NUS	2017_MO_COL	43	MO151826	4.9	7.5	NA
2017	NUS	2017_MO_COL	44	MO150133	13.4	12.5	NA
2017	NUS	2017_MO_COL	45	0566A1-3-1-1-63	9.3	20.0	NA
2017	NUS	2017_MO_COL	46	04620A1-1-7-4-10	10.4	5.0	NA
2017	NUS	2017_MO_COL	47	0527A1-9-14-4-3-3	18.0	10.0	NA
2017	NUS	2017_MO_COL	48	0762A1-2-8	8.8	22.5	NA
2017	NUS	2017_MO_COL	49	07419A1-16-1-1-16-1-1	16.7	15.0	NA
2017	NUS	2017_MO_COL	50	LES15-5199	13.5	20.0	NA
2017	NUS	2017_MO_COL	51	LES15-7011	8.5	10.0	NA
2017	NUS	2017_MO_COL	52	LES15-5540	6.7	15.0	NA
2017	NUS	2017_MO_COL	53	LES15-5605	11.5	10.0	NA
2017	NUS	2017_MO_COL	54	LES15-7004	13.2	7.5	NA
2017	NUS	2017_MO_COL	55	U6714-B-041	22.3	5.0	NA
2017	NUS	2017_MO_COL	56	MI14R0008	13.1	7.5	NA

2017	NUS	2017_MO_COL	57	MI14R0009	10.9	10.0	NA
2017	NUS	2017_MO_COL	58	MI14R0421	13.3	12.5	NA
2017	NUS	2017_MO_COL	59	MI14R0267	9.0	5.0	NA
2017	NUS	2017_MO_COL	60	OH09-207-68	7.7	10.0	NA
2017	NUS	2017_NE_MEA	1	TRUMAN	17.4	NA	NA
2017	NUS	2017_NE_MEA	2	ERNIE	37.9	NA	NA
2017	NUS	2017_NE_MEA	3	FREEDOM	13.9	NA	NA
2017	NUS	2017_NE_MEA	4	PIONEER2545	32.6	NA	NA
2017	NUS	2017_NE_MEA	5	VA11W-108PA	37.8	NA	NA
2017	NUS	2017_NE_MEA	6	VA09MAS1-12-5-1	20.1	NA	NA
2017	NUS	2017_NE_MEA	7	VA14FHB-29	13.2	NA	NA
2017	NUS	2017_NE_MEA	8	VA09MAS8-34-5-2	22.1	NA	NA
2017	NUS	2017_NE_MEA	9	VA09MAS3-34-2-1	43.4	NA	NA
2017	NUS	2017_NE_MEA	10	KY09C-0052-26-12-3	36.1	NA	NA
2017	NUS	2017_NE_MEA	11	X08C-1070-74-20-1	25.8	NA	NA
2017	NUS	2017_NE_MEA	12	KY09C-1245-100-1-3	45.3	NA	NA
2017	NUS	2017_NE_MEA	13	KY09C-0267-45-16-3	24.9	NA	NA
2017	NUS	2017_NE_MEA	14	X08C-1090-51-12-5	16.7	NA	NA
2017	NUS	2017_NE_MEA	15	OH12-195-22	23.4	NA	NA
2017	NUS	2017_NE_MEA	16	OH11-118-18	10.7	NA	NA
2017	NUS	2017_NE_MEA	17	OH12-133-74	19.5	NA	NA
2017	NUS	2017_NE_MEA	18	OH12-194-24	29.9	NA	NA
2017	NUS	2017_NE_MEA	19	OH13-16-25	36.0	NA	NA
2017	NUS	2017_NE_MEA	20	NE14538	14.2	NA	NA
2017	NUS	2017_NE_MEA	21	NE14606	27.2	NA	NA
2017	NUS	2017_NE_MEA	22	NE14696	7.4	NA	NA
2017	NUS	2017_NE_MEA	23	NI12702W	10.7	NA	NA
2017	NUS	2017_NE_MEA	24	NE15545	12.2	NA	NA
2017	NUS	2017_NE_MEA	25	KWS095	21.3	NA	NA
2017	NUS	2017_NE_MEA	26	KWS103	27.9	NA	NA
2017	NUS	2017_NE_MEA	27	KWS122	18.8	NA	NA
2017	NUS	2017_NE_MEA	28	KWS127	28.6	NA	NA
2017	NUS	2017_NE_MEA	29	KWS141	33.1	NA	NA
2017	NUS	2017_NE_MEA	30	IL09-3264-T2	26.6	NA	NA
2017	NUS	2017_NE_MEA	31	IL12-21235	57.4	NA	NA
2017	NUS	2017_NE_MEA	32	IL12-26004	36.9	NA	NA
2017	NUS	2017_NE_MEA	33	IL13-451	38.7	NA	NA
2017	NUS	2017_NE_MEA	34	IL13-20616	44.0	NA	NA
2017	NUS	2017_NE_MEA	35	NY99056-161	18.8	NA	NA
2017	NUS	2017_NE_MEA	36	NYWHATFORD/7388-39-693	11.9	NA	NA
2017	NUS	2017_NE_MEA	37	NY09087-15-69-1124	22.8	NA	NA
2017	NUS	2017_NE_MEA	38	NY01016-AN	22.7	NA	NA
2017	NUS	2017_NE_MEA	39	NY09125-16-1034	34.6	NA	NA
2017	NUS	2017_NE_MEA	40	MO140304	22.1	NA	NA
2017	NUS	2017_NE_MEA	41	MO151323	34.3	NA	NA
2017	NUS	2017_NE_MEA	42	MO151031	24.7	NA	NA

2017	NUS	2017_NE_MEA	43	MO151826	17.7	NA	NA
2017	NUS	2017_NE_MEA	44	MO150133	41.7	NA	NA
2017	NUS	2017_NE_MEA	45	0566A1-3-1-1-63	44.7	NA	NA
2017	NUS	2017_NE_MEA	46	04620A1-1-7-4-10	30.3	NA	NA
2017	NUS	2017_NE_MEA	47	0527A1-9-14-4-3-3	36.1	NA	NA
2017	NUS	2017_NE_MEA	48	0762A1-2-8	51.6	NA	NA
2017	NUS	2017_NE_MEA	49	07419A1-16-1-1-16-1-1	35.3	NA	NA
2017	NUS	2017_NE_MEA	50	LES15-5199	35.8	NA	NA
2017	NUS	2017_NE_MEA	51	LES15-7011	62.2	NA	NA
2017	NUS	2017_NE_MEA	52	LES15-5540	51.2	NA	NA
2017	NUS	2017_NE_MEA	53	LES15-5605	66.5	NA	NA
2017	NUS	2017_NE_MEA	54	LES15-7004	43.3	NA	NA
2017	NUS	2017_NE_MEA	55	U6714-B-041	39.8	NA	NA
2017	NUS	2017_NE_MEA	56	MI14R0008	48.2	NA	NA
2017	NUS	2017_NE_MEA	57	MI14R0009	35.2	NA	NA
2017	NUS	2017_NE_MEA	58	MI14R0421	20.8	NA	NA
2017	NUS	2017_NE_MEA	59	MI14R0267	25.9	NA	NA
2017	NUS	2017_NE_MEA	60	OH09-207-68	44.9	NA	NA
2018	NUS	2018_IL_URB	1	TRUMAN	7.0	1.3	0.4
2018	NUS	2018_IL_URB	2	ERNIE	60.0	7.7	0.6
2018	NUS	2018_IL_URB	3	FREEDOM	37.0	28.3	1.5
2018	NUS	2018_IL_URB	4	PIONEER2545	63.0	36.7	4.3
2018	NUS	2018_IL_URB	5	IL13-20616	13.0	5.7	0.7
2018	NUS	2018_IL_URB	6	IL14-11718	40.0	11.7	1.1
2018	NUS	2018_IL_URB	7	IL14-11848	7.0	12.0	0.6
2018	NUS	2018_IL_URB	8	IL14-28462	43.0	18.4	0.2
2018	NUS	2018_IL_URB	9	IL14-DC-64-95-118	27.0	11.0	0.8
2018	NUS	2018_IL_URB	10	VA15W-68	57.0	33.3	3.1
2018	NUS	2018_IL_URB	11	VA16W-29	57.0	20.0	2.6
2018	NUS	2018_IL_URB	12	VA16W-148	27.0	36.7	2.5
2018	NUS	2018_IL_URB	13	VA16W-149	50.0	26.0	0.8
2018	NUS	2018_IL_URB	14	13VA-FHB-DH252	77.0	8.3	0.6
2018	NUS	2018_IL_URB	15	KY06C-1195-37-2-5	37.0	18.3	1.5
2018	NUS	2018_IL_URB	16	X08C-1181-61-15-5	47.0	40.0	1.7
2018	NUS	2018_IL_URB	17	KY06C-1178-16-10-3	33.0	11.0	1.3
2018	NUS	2018_IL_URB	18	KY07C-1145-94-12-5	17.0	9.3	0.9
2018	NUS	2018_IL_URB	19	X08C-1077-11-18-3	20.0	9.3	0.9
2018	NUS	2018_IL_URB	20	NY11013-10-72-1314	40.0	35.0	0.7
2018	NUS	2018_IL_URB	21	NY11013-10-15-1312	70.0	30.0	0.6
2018	NUS	2018_IL_URB	22	NY02008-807	30.0	18.3	1.8
2018	NUS	2018_IL_URB	23	NY09095-16-928	10.0	10.0	1.3
2018	NUS	2018_IL_URB	24	NY02007-1206	40.0	16.0	3.1
2018	NUS	2018_IL_URB	25	KWS152	53.0	45.0	1.7
2018	NUS	2018_IL_URB	26	KWS149	57.0	9.3	2.0
2018	NUS	2018_IL_URB	27	KWS192	33.0	12.7	1.5
2018	NUS	2018_IL_URB	28	KWS191	43.0	35.0	2.7
2018	NUS	2018_IL_URB	29	KWS193	43.0	6.3	1.0
2018	NUS	2018_IL_URB	30	MI14R0267	40.0	6.0	1.5
2018	NUS	2018_IL_URB	31	MI14W0190	13.0	2.7	0.8

2018	NUS	2018_IL_URB	32	MI14W0906	57.0	73.3	2.4
2018	NUS	2018_IL_URB	33	MI14R1145	17.0	23.3	1.7
2018	NUS	2018_IL_URB	34	MO131753	30.0	9.7	0.3
2018	NUS	2018_IL_URB	35	MO151163	20.0	1.4	0.4
2018	NUS	2018_IL_URB	36	MO151126	33.0	8.3	1.0
2018	NUS	2018_IL_URB	37	MO160132	30.0	1.0	0.5
2018	NUS	2018_IL_URB	38	MO160140	43.0	5.3	0.6
2018	NUS	2018_IL_URB	39	0566A1-3-1-1-63	30.0	56.7	1.1
2018	NUS	2018_IL_URB	40	04620A1-1-7-4-10	60.0	20.0	2.4
2018	NUS	2018_IL_URB	41	0527A1-9-14-4-3-3	7.0	14.9	0.3
2018	NUS	2018_IL_URB	42	0762A1-2-8	10.0	8.7	0.2
2018	NUS	2018_IL_URB	43	07419A1-16-1-1-16-1-1	57.0	26.7	1.5
2018	NUS	2018_IL_URB	44	OH12-194-24	47.0	13.3	2.2
2018	NUS	2018_IL_URB	45	OH12-195-22	33.0	20.0	0.9
2018	NUS	2018_IL_URB	46	OH13-314-18	67.0	25.0	1.8
2018	NUS	2018_IL_URB	47	NE13604	40.0	10.7	1.1
2018	NUS	2018_IL_URB	48	NW13493	77.0	23.3	2.0
2018	NUS	2018_IL_URB	49	NE13515	63.0	33.3	2.1
2018	NUS	2018_IL_URB	50	NE14494	50.0	17.7	1.4
2018	NUS	2018_IL_URB	51	NE14696	43.0	21.0	0.6
2018	NUS	2018_IL_URB	52	LES168062	57.0	7.3	1.3
2018	NUS	2018_IL_URB	53	LES167062	57.0	16.7	1.5
2018	NUS	2018_IL_URB	54	LES167906	47.0	31.7	2.4
2018	NUS	2018_IL_URB	55	LES167499	67.0	26.7	2.1
2018	NUS	2018_IL_URB	500	PIONEER25R47	50.0	28.3	0.9
2018	NUS	2018_IL_URB	501	IL07-4415	10.0	3.7	1.3
2018	NUS	2018_IL_URB	502	IL02-18228	10.0	1.0	42.3
2018	NUS	2018_IN_LAY	1	TRUMAN	25.0	NA	NA
2018	NUS	2018_IN_LAY	2	ERNIE	40.0	NA	NA
2018	NUS	2018_IN_LAY	3	FREEDOM	30.0	NA	NA
2018	NUS	2018_IN_LAY	4	PIONEER2545	50.0	NA	NA
2018	NUS	2018_IN_LAY	5	IL13-20616	15.0	NA	NA
2018	NUS	2018_IN_LAY	6	IL14-11718	15.0	NA	NA
2018	NUS	2018_IN_LAY	7	IL14-11848	10.0	NA	NA
2018	NUS	2018_IN_LAY	8	IL14-28462	10.0	NA	NA
2018	NUS	2018_IN_LAY	9	IL14-DC-64-95-118	20.0	NA	NA
2018	NUS	2018_IN_LAY	10	VA15W-68	50.0	NA	NA
2018	NUS	2018_IN_LAY	11	VA16W-29	55.0	NA	NA
2018	NUS	2018_IN_LAY	12	VA16W-148	30.0	NA	NA
2018	NUS	2018_IN_LAY	13	VA16W-149	40.0	NA	NA
2018	NUS	2018_IN_LAY	14	13VA-FHB-DH252	25.0	NA	NA
2018	NUS	2018_IN_LAY	15	KY06C-1195-37-2-5	55.0	NA	NA
2018	NUS	2018_IN_LAY	16	X08C-1181-61-15-5	30.0	NA	NA
2018	NUS	2018_IN_LAY	17	KY06C-1178-16-10-3	45.0	NA	NA
2018	NUS	2018_IN_LAY	18	KY07C-1145-94-12-5	20.0	NA	NA
2018	NUS	2018_IN_LAY	19	X08C-1077-11-18-3	30.0	NA	NA
2018	NUS	2018_IN_LAY	20	NY11013-10-72-1314	30.0	NA	NA
2018	NUS	2018_IN_LAY	21	NY11013-10-15-1312	65.0	NA	NA
2018	NUS	2018_IN_LAY	22	NY02008-807	20.0	NA	NA

2018	NUS	2018_IN_LAY	23	NY09095-16-928	25.0	NA	NA
2018	NUS	2018_IN_LAY	24	NY02007-1206	60.0	NA	NA
2018	NUS	2018_IN_LAY	25	KWS152	55.0	NA	NA
2018	NUS	2018_IN_LAY	26	KWS149	35.0	NA	NA
2018	NUS	2018_IN_LAY	27	KWS192	50.0	NA	NA
2018	NUS	2018_IN_LAY	28	KWS191	40.0	NA	NA
2018	NUS	2018_IN_LAY	29	KWS193	25.0	NA	NA
2018	NUS	2018_IN_LAY	30	MI14R0267	30.0	NA	NA
2018	NUS	2018_IN_LAY	31	MI14W0190	20.0	NA	NA
2018	NUS	2018_IN_LAY	32	MI14W0906	60.0	NA	NA
2018	NUS	2018_IN_LAY	33	MI14R1145	25.0	NA	NA
2018	NUS	2018_IN_LAY	34	MO131753	25.0	NA	NA
2018	NUS	2018_IN_LAY	35	MO151163	15.0	NA	NA
2018	NUS	2018_IN_LAY	36	MO151126	20.0	NA	NA
2018	NUS	2018_IN_LAY	37	MO160132	15.0	NA	NA
2018	NUS	2018_IN_LAY	38	MO160140	20.0	NA	NA
2018	NUS	2018_IN_LAY	39	0566A1-3-1-1-63	25.0	NA	NA
2018	NUS	2018_IN_LAY	40	04620A1-1-7-4-10	30.0	NA	NA
2018	NUS	2018_IN_LAY	41	0527A1-9-14-4-3-3	10.0	NA	NA
2018	NUS	2018_IN_LAY	42	0762A1-2-8	10.0	NA	NA
2018	NUS	2018_IN_LAY	43	07419A1-16-1-1-16-1-1	40.0	NA	NA
2018	NUS	2018_IN_LAY	44	OH12-194-24	50.0	NA	NA
2018	NUS	2018_IN_LAY	45	OH12-195-22	30.0	NA	NA
2018	NUS	2018_IN_LAY	46	OH13-314-18	50.0	NA	NA
2018	NUS	2018_IN_LAY	47	NE13604	50.0	NA	NA
2018	NUS	2018_IN_LAY	48	NW13493	65.0	NA	NA
2018	NUS	2018_IN_LAY	49	NE13515	50.0	NA	NA
2018	NUS	2018_IN_LAY	50	NE14494	60.0	NA	NA
2018	NUS	2018_IN_LAY	51	NE14696	50.0	NA	NA
2018	NUS	2018_IN_LAY	52	LES168062	25.0	NA	NA
2018	NUS	2018_IN_LAY	53	LES167062	55.0	NA	NA
2018	NUS	2018_IN_LAY	54	LES167906	55.0	NA	NA
2018	NUS	2018_IN_LAY	55	LES167499	60.0	NA	NA
2018	NUS	2018_IN_WLA	1	TRUMAN	15.0	NA	2.8
2018	NUS	2018_IN_WLA	2	ERNIE	12.0	NA	1.7
2018	NUS	2018_IN_WLA	3	FREEDOM	20.0	NA	8.5
2018	NUS	2018_IN_WLA	4	PIONEER2545	54.0	NA	4.2
2018	NUS	2018_IN_WLA	5	IL13-20616	8.0	NA	1.1
2018	NUS	2018_IN_WLA	6	IL14-11718	5.0	NA	2.7
2018	NUS	2018_IN_WLA	7	IL14-11848	5.0	NA	0.7
2018	NUS	2018_IN_WLA	8	IL14-28462	17.0	NA	4.2
2018	NUS	2018_IN_WLA	9	IL14-DC-64-95-118	11.0	NA	1.9
2018	NUS	2018_IN_WLA	10	VA15W-68	44.0	NA	4.4
2018	NUS	2018_IN_WLA	11	VA16W-29	49.0	NA	1.5
2018	NUS	2018_IN_WLA	12	VA16W-148	32.0	NA	10.8
2018	NUS	2018_IN_WLA	13	VA16W-149	50.0	NA	6.1
2018	NUS	2018_IN_WLA	14	13VA-FHB-DH252	27.0	NA	2.8
2018	NUS	2018_IN_WLA	15	KY06C-1195-37-2-5	24.0	NA	1.5
2018	NUS	2018_IN_WLA	16	X08C-1181-61-15-5	27.0	NA	2.1

2018	NUS	2018_IN_WLA	17	KY06C-1178-16-10-3	12.0	NA	5.1
2018	NUS	2018_IN_WLA	18	KY07C-1145-94-12-5	17.0	NA	1.0
2018	NUS	2018_IN_WLA	19	X08C-1077-11-18-3	14.0	NA	2.7
2018	NUS	2018_IN_WLA	20	NY11013-10-72-1314	4.0	NA	0.6
2018	NUS	2018_IN_WLA	21	NY11013-10-15-1312	5.0	NA	0.8
2018	NUS	2018_IN_WLA	22	NY02008-807	15.0	NA	1.7
2018	NUS	2018_IN_WLA	23	NY09095-16-928	27.0	NA	6.3
2018	NUS	2018_IN_WLA	24	NY02007-1206	4.0	NA	1.5
2018	NUS	2018_IN_WLA	25	KWS152	29.0	NA	1.6
2018	NUS	2018_IN_WLA	26	KWS149	39.0	NA	2.2
2018	NUS	2018_IN_WLA	27	KWS192	20.0	NA	0.7
2018	NUS	2018_IN_WLA	28	KWS191	8.0	NA	3.4
2018	NUS	2018_IN_WLA	29	KWS193	25.0	NA	4.3
2018	NUS	2018_IN_WLA	30	MI14R0267	9.0	NA	3.6
2018	NUS	2018_IN_WLA	31	MI14W0190	8.0	NA	1.2
2018	NUS	2018_IN_WLA	32	MI14W0906	82.0	NA	1.6
2018	NUS	2018_IN_WLA	33	MI14R1145	24.0	NA	3.7
2018	NUS	2018_IN_WLA	34	MO131753	35.0	NA	2.4
2018	NUS	2018_IN_WLA	35	MO151163	7.0	NA	1.2
2018	NUS	2018_IN_WLA	36	MO151126	15.0	NA	2.2
2018	NUS	2018_IN_WLA	37	MO160132	11.0	NA	0.9
2018	NUS	2018_IN_WLA	38	MO160140	9.0	NA	1.6
2018	NUS	2018_IN_WLA	39	0566A1-3-1-1-63	32.0	NA	2.1
2018	NUS	2018_IN_WLA	40	04620A1-1-7-4-10	28.0	NA	6.7
2018	NUS	2018_IN_WLA	41	0527A1-9-14-4-3-3	13.0	NA	4.3
2018	NUS	2018_IN_WLA	42	0762A1-2-8	21.0	NA	2.3
2018	NUS	2018_IN_WLA	43	07419A1-16-1-1-16-1-1	18.0	NA	1.4
2018	NUS	2018_IN_WLA	44	OH12-194-24	46.0	NA	5.6
2018	NUS	2018_IN_WLA	45	OH12-195-22	44.0	NA	11.6
2018	NUS	2018_IN_WLA	46	OH13-314-18	71.0	NA	3.9
2018	NUS	2018_IN_WLA	47	NE13604	39.0	NA	5.1
2018	NUS	2018_IN_WLA	48	NW13493	33.0	NA	3.3
2018	NUS	2018_IN_WLA	49	NE13515	46.0	NA	7.4
2018	NUS	2018_IN_WLA	50	NE14494	43.0	NA	7.2
2018	NUS	2018_IN_WLA	51	NE14696	26.0	NA	6.7
2018	NUS	2018_IN_WLA	52	LES168062	27.0	NA	1.9
2018	NUS	2018_IN_WLA	53	LES167062	44.0	NA	2.3
2018	NUS	2018_IN_WLA	54	LES167906	50.0	NA	8.6
2018	NUS	2018_IN_WLA	55	LES167499	45.0	NA	3.8
2018	NUS	2018_KY_LEX	1	TRUMAN	NA	12.5	6.0
2018	NUS	2018_KY_LEX	2	ERNIE	NA	10.0	4.0
2018	NUS	2018_KY_LEX	3	FREEDOM	NA	12.5	7.2
2018	NUS	2018_KY_LEX	4	PIONEER2545	NA	15.0	7.2
2018	NUS	2018_KY_LEX	5	IL13-20616	NA	12.5	3.0
2018	NUS	2018_KY_LEX	6	IL14-11718	NA	12.5	3.9
2018	NUS	2018_KY_LEX	7	IL14-11848	NA	7.5	1.4
2018	NUS	2018_KY_LEX	8	IL14-28462	NA	5.0	3.0
2018	NUS	2018_KY_LEX	9	IL14-DC-64-95-118	NA	5.0	2.7
2018	NUS	2018_KY_LEX	10	VA15W-68	NA	22.5	10.3

2018	NUS	2018_KY_LEX	11	VA16W-29	NA	15.0	7.0
2018	NUS	2018_KY_LEX	12	VA16W-148	NA	22.5	9.5
2018	NUS	2018_KY_LEX	13	VA16W-149	NA	35.0	5.1
2018	NUS	2018_KY_LEX	14	13VA-FHB-DH252	NA	25.0	8.8
2018	NUS	2018_KY_LEX	15	KY06C-1195-37-2-5	NA	10.0	6.5
2018	NUS	2018_KY_LEX	16	X08C-1181-61-15-5	NA	20.0	5.2
2018	NUS	2018_KY_LEX	17	KY06C-1178-16-10-3	NA	5.0	3.9
2018	NUS	2018_KY_LEX	18	KY07C-1145-94-12-5	NA	12.5	4.1
2018	NUS	2018_KY_LEX	19	X08C-1077-11-18-3	NA	5.0	3.5
2018	NUS	2018_KY_LEX	20	NY11013-10-72-1314	NA	30.0	6.1
2018	NUS	2018_KY_LEX	21	NY11013-10-15-1312	NA	17.5	6.9
2018	NUS	2018_KY_LEX	22	NY02008-807	NA	15.0	13.1
2018	NUS	2018_KY_LEX	23	NY09095-16-928	NA	5.0	4.3
2018	NUS	2018_KY_LEX	24	NY02007-1206	NA	22.5	13.2
2018	NUS	2018_KY_LEX	25	KWS152	NA	25.0	9.3
2018	NUS	2018_KY_LEX	26	KWS149	NA	12.5	3.6
2018	NUS	2018_KY_LEX	27	KWS192	NA	7.5	2.7
2018	NUS	2018_KY_LEX	28	KWS191	NA	15.0	6.9
2018	NUS	2018_KY_LEX	29	KWS193	NA	7.5	5.3
2018	NUS	2018_KY_LEX	30	MI14R0267	NA	7.5	4.2
2018	NUS	2018_KY_LEX	31	MI14W0190	NA	5.0	7.1
2018	NUS	2018_KY_LEX	32	MI14W0906	NA	40.0	8.5
2018	NUS	2018_KY_LEX	33	MI14R1145	NA	10.0	6.3
2018	NUS	2018_KY_LEX	34	MO131753	NA	7.5	4.0
2018	NUS	2018_KY_LEX	35	MO151163	NA	10.0	2.6
2018	NUS	2018_KY_LEX	36	MO151126	NA	5.0	0.6
2018	NUS	2018_KY_LEX	37	MO160132	NA	5.0	4.4
2018	NUS	2018_KY_LEX	38	MO160140	NA	10.0	6.6
2018	NUS	2018_KY_LEX	39	0566A1-3-1-1-63	NA	37.5	5.8
2018	NUS	2018_KY_LEX	40	04620A1-1-7-4-10	NA	10.0	5.1
2018	NUS	2018_KY_LEX	41	0527A1-9-14-4-3-3	NA	7.5	3.7
2018	NUS	2018_KY_LEX	42	0762A1-2-8	NA	22.5	4.0
2018	NUS	2018_KY_LEX	43	07419A1-16-1-1-16-1-1	NA	17.5	5.7
2018	NUS	2018_KY_LEX	44	OH12-194-24	NA	15.0	7.5
2018	NUS	2018_KY_LEX	45	OH12-195-22	NA	17.5	5.7
2018	NUS	2018_KY_LEX	46	OH13-314-18	NA	35.0	7.0
2018	NUS	2018_KY_LEX	47	NE13604	NA	25.0	7.8
2018	NUS	2018_KY_LEX	48	NW13493	NA	7.5	5.0
2018	NUS	2018_KY_LEX	49	NE13515	NA	22.5	5.1
2018	NUS	2018_KY_LEX	50	NE14494	NA	20.0	6.7
2018	NUS	2018_KY_LEX	51	NE14696	NA	10.0	7.0
2018	NUS	2018_KY_LEX	52	LES168062	NA	7.5	2.1
2018	NUS	2018_KY_LEX	53	LES167062	NA	12.5	3.9
2018	NUS	2018_KY_LEX	54	LES167906	NA	12.5	8.6
2018	NUS	2018_KY_LEX	55	LES167499	NA	40.0	6.6
2018	NUS	2018_MI_ELA	1	TRUMAN	13.3	NA	NA
2018	NUS	2018_MI_ELA	2	ERNIE	26.7	NA	NA
2018	NUS	2018_MI_ELA	3	FREEDOM	21.7	NA	NA
2018	NUS	2018_MI_ELA	4	PIONEER2545	58.3	NA	NA

2018	NUS	2018_MI_ELA	5	IL13-20616	26.7	NA	NA
2018	NUS	2018_MI_ELA	6	IL14-11718	33.3	NA	NA
2018	NUS	2018_MI_ELA	7	IL14-11848	23.3	NA	NA
2018	NUS	2018_MI_ELA	8	IL14-28462	18.3	NA	NA
2018	NUS	2018_MI_ELA	9	IL14-DC-64-95-118	43.3	NA	NA
2018	NUS	2018_MI_ELA	10	VA15W-68	40.0	NA	NA
2018	NUS	2018_MI_ELA	11	VA16W-29	20.0	NA	NA
2018	NUS	2018_MI_ELA	12	VA16W-148	33.3	NA	NA
2018	NUS	2018_MI_ELA	13	VA16W-149	23.3	NA	NA
2018	NUS	2018_MI_ELA	14	13VA-FHB-DH252	30.0	NA	NA
2018	NUS	2018_MI_ELA	15	KY06C-1195-37-2-5	25.0	NA	NA
2018	NUS	2018_MI_ELA	16	X08C-1181-61-15-5	41.7	NA	NA
2018	NUS	2018_MI_ELA	17	KY06C-1178-16-10-3	23.3	NA	NA
2018	NUS	2018_MI_ELA	18	KY07C-1145-94-12-5	28.3	NA	NA
2018	NUS	2018_MI_ELA	19	X08C-1077-11-18-3	25.0	NA	NA
2018	NUS	2018_MI_ELA	20	NY11013-10-72-1314	50.0	NA	NA
2018	NUS	2018_MI_ELA	21	NY11013-10-15-1312	45.0	NA	NA
2018	NUS	2018_MI_ELA	22	NY02008-807	18.3	NA	NA
2018	NUS	2018_MI_ELA	23	NY09095-16-928	21.7	NA	NA
2018	NUS	2018_MI_ELA	24	NY02007-1206	68.3	NA	NA
2018	NUS	2018_MI_ELA	25	KWS152	63.3	NA	NA
2018	NUS	2018_MI_ELA	26	KWS149	38.3	NA	NA
2018	NUS	2018_MI_ELA	27	KWS192	40.0	NA	NA
2018	NUS	2018_MI_ELA	28	KWS191	40.0	NA	NA
2018	NUS	2018_MI_ELA	29	KWS193	41.7	NA	NA
2018	NUS	2018_MI_ELA	30	MI14R0267	10.0	NA	NA
2018	NUS	2018_MI_ELA	31	MI14W0190	18.3	NA	NA
2018	NUS	2018_MI_ELA	32	MI14W0906	46.7	NA	NA
2018	NUS	2018_MI_ELA	33	MI14R1145	36.7	NA	NA
2018	NUS	2018_MI_ELA	34	MO131753	15.0	NA	NA
2018	NUS	2018_MI_ELA	35	MO151163	15.0	NA	NA
2018	NUS	2018_MI_ELA	36	MO151126	15.0	NA	NA
2018	NUS	2018_MI_ELA	37	MO160132	20.0	NA	NA
2018	NUS	2018_MI_ELA	38	MO160140	33.3	NA	NA
2018	NUS	2018_MI_ELA	39	0566A1-3-1-1-63	33.3	NA	NA
2018	NUS	2018_MI_ELA	40	04620A1-1-7-4-10	73.3	NA	NA
2018	NUS	2018_MI_ELA	41	0527A1-9-14-4-3-3	21.7	NA	NA
2018	NUS	2018_MI_ELA	42	0762A1-2-8	25.0	NA	NA
2018	NUS	2018_MI_ELA	43	07419A1-16-1-1-16-1-1	18.3	NA	NA
2018	NUS	2018_MI_ELA	44	OH12-194-24	46.7	NA	NA
2018	NUS	2018_MI_ELA	45	OH12-195-22	35.0	NA	NA
2018	NUS	2018_MI_ELA	46	OH13-314-18	71.7	NA	NA
2018	NUS	2018_MI_ELA	47	NE13604	35.0	NA	NA
2018	NUS	2018_MI_ELA	48	NW13493	35.0	NA	NA
2018	NUS	2018_MI_ELA	49	NE13515	40.0	NA	NA
2018	NUS	2018_MI_ELA	50	NE14494	28.3	NA	NA
2018	NUS	2018_MI_ELA	51	NE14696	23.3	NA	NA
2018	NUS	2018_MI_ELA	52	LES168062	31.7	NA	NA
2018	NUS	2018_MI_ELA	53	LES167062	55.0	NA	NA

2018	NUS	2018_MI_ELA	54	LES167906	58.3	NA	NA
2018	NUS	2018_MI_ELA	55	LES167499	83.3	NA	NA
2018	NUS	2018_MO_COL	1	TRUMAN	7.4	NA	NA
2018	NUS	2018_MO_COL	2	ERNIE	25.0	NA	NA
2018	NUS	2018_MO_COL	3	FREEDOM	27.1	NA	NA
2018	NUS	2018_MO_COL	4	PIONEER2545	35.3	NA	NA
2018	NUS	2018_MO_COL	5	IL13-20616	23.8	NA	NA
2018	NUS	2018_MO_COL	6	IL14-11718	41.3	NA	NA
2018	NUS	2018_MO_COL	7	IL14-11848	11.3	NA	NA
2018	NUS	2018_MO_COL	8	IL14-28462	66.8	NA	NA
2018	NUS	2018_MO_COL	9	IL14-DC-64-95-118	32.5	NA	NA
2018	NUS	2018_MO_COL	10	VA15W-68	25.1	NA	NA
2018	NUS	2018_MO_COL	11	VA16W-29	21.7	NA	NA
2018	NUS	2018_MO_COL	12	VA16W-148	19.6	NA	NA
2018	NUS	2018_MO_COL	13	VA16W-149	20.9	NA	NA
2018	NUS	2018_MO_COL	14	13VA-FHB-DH252	16.2	NA	NA
2018	NUS	2018_MO_COL	15	KY06C-1195-37-2-5	33.4	NA	NA
2018	NUS	2018_MO_COL	16	X08C-1181-61-15-5	26.5	NA	NA
2018	NUS	2018_MO_COL	17	KY06C-1178-16-10-3	16.3	NA	NA
2018	NUS	2018_MO_COL	18	KY07C-1145-94-12-5	14.9	NA	NA
2018	NUS	2018_MO_COL	19	X08C-1077-11-18-3	44.1	NA	NA
2018	NUS	2018_MO_COL	20	NY11013-10-72-1314	31.6	NA	NA
2018	NUS	2018_MO_COL	21	NY11013-10-15-1312	39.6	NA	NA
2018	NUS	2018_MO_COL	22	NY02008-807	36.6	NA	NA
2018	NUS	2018_MO_COL	23	NY09095-16-928	11.6	NA	NA
2018	NUS	2018_MO_COL	24	NY02007-1206	27.1	NA	NA
2018	NUS	2018_MO_COL	25	KWS152	34.6	NA	NA
2018	NUS	2018_MO_COL	26	KWS149	33.3	NA	NA
2018	NUS	2018_MO_COL	27	KWS192	22.8	NA	NA

2018	NUS	2018_MO_COL	28	KWS191	34.1	NA	NA
2018	NUS	2018_MO_COL	29	KWS193	32.6	NA	NA
2018	NUS	2018_MO_COL	30	MI14R0267	15.6	NA	NA
2018	NUS	2018_MO_COL	31	MI14W0190	20.7	NA	NA
2018	NUS	2018_MO_COL	32	MI14W0906	31.6	NA	NA
2018	NUS	2018_MO_COL	33	MI14R1145	13.7	NA	NA
2018	NUS	2018_MO_COL	34	MO131753	25.9	NA	NA
2018	NUS	2018_MO_COL	35	MO151163	20.6	NA	NA
2018	NUS	2018_MO_COL	36	MO151126	22.8	NA	NA
2018	NUS	2018_MO_COL	37	MO160132	38.9	NA	NA
2018	NUS	2018_MO_COL	38	MO160140	32.3	NA	NA
2018	NUS	2018_MO_COL	39	0566A1-3-1-1-63	22.3	NA	NA
2018	NUS	2018_MO_COL	40	04620A1-1-7-4-10	54.4	NA	NA
2018	NUS	2018_MO_COL	41	0527A1-9-14-4-3-3	33.7	NA	NA
2018	NUS	2018_MO_COL	42	0762A1-2-8	16.3	NA	NA
2018	NUS	2018_MO_COL	43	07419A1-16-1-1-16-1-1	13.6	NA	NA
2018	NUS	2018_MO_COL	44	OH12-194-24	17.8	NA	NA
2018	NUS	2018_MO_COL	45	OH12-195-22	17.5	NA	NA
2018	NUS	2018_MO_COL	46	OH13-314-18	19.8	NA	NA
2018	NUS	2018_MO_COL	47	NE13604	33.4	NA	NA
2018	NUS	2018_MO_COL	48	NW13493	19.0	NA	NA
2018	NUS	2018_MO_COL	49	NE13515	36.3	NA	NA
2018	NUS	2018_MO_COL	50	NE14494	34.2	NA	NA
2018	NUS	2018_MO_COL	51	NE14696	49.6	NA	NA
2018	NUS	2018_MO_COL	52	LES168062	8.4	NA	NA
2018	NUS	2018_MO_COL	53	LES167062	18.9	NA	NA
2018	NUS	2018_MO_COL	54	LES167906	41.2	NA	NA
2018	NUS	2018_MO_COL	55	LES167499	20.8	NA	NA

2018	NUS	2018_NY_ITH	1	TRUMAN	9.5	35.0	NA
2018	NUS	2018_NY_ITH	2	ERNIE	15.5	50.0	NA
2018	NUS	2018_NY_ITH	3	FREEDOM	19.5	70.0	NA
2018	NUS	2018_NY_ITH	4	PIONEER2545	24.5	85.0	NA
2018	NUS	2018_NY_ITH	5	IL13-20616	10.3	40.0	NA
2018	NUS	2018_NY_ITH	6	IL14-11718	7.5	45.0	NA
2018	NUS	2018_NY_ITH	7	IL14-11848	7.3	35.0	NA
2018	NUS	2018_NY_ITH	8	IL14-28462	9.0	60.0	NA
2018	NUS	2018_NY_ITH	9	IL14-DC-64-95-118	11.0	55.0	NA
2018	NUS	2018_NY_ITH	10	VA15W-68	16.5	70.0	NA
2018	NUS	2018_NY_ITH	11	VA16W-29	24.0	80.0	NA
2018	NUS	2018_NY_ITH	12	VA16W-148	15.8	65.0	NA
2018	NUS	2018_NY_ITH	13	VA16W-149	15.3	75.0	NA
2018	NUS	2018_NY_ITH	14	13VA-FHB-DH252	17.8	35.0	NA
2018	NUS	2018_NY_ITH	15	KY06C-1195-37-2-5	22.3	55.0	NA
2018	NUS	2018_NY_ITH	16	X08C-1181-61-15-5	24.5	75.0	NA
2018	NUS	2018_NY_ITH	17	KY06C-1178-16-10-3	11.3	75.0	NA
2018	NUS	2018_NY_ITH	18	KY07C-1145-94-12-5	10.0	45.0	NA
2018	NUS	2018_NY_ITH	19	X08C-1077-11-18-3	14.0	60.0	NA
2018	NUS	2018_NY_ITH	20	NY11013-10-72-1314	16.0	75.0	NA
2018	NUS	2018_NY_ITH	21	NY11013-10-15-1312	32.0	70.0	NA
2018	NUS	2018_NY_ITH	22	NY02008-807	22.0	45.0	NA
2018	NUS	2018_NY_ITH	23	NY09095-16-928	9.5	45.0	NA
2018	NUS	2018_NY_ITH	24	NY02007-1206	38.8	75.0	NA
2018	NUS	2018_NY_ITH	25	KWS152	16.5	70.0	NA
2018	NUS	2018_NY_ITH	26	KWS149	13.3	55.0	NA
2018	NUS	2018_NY_ITH	27	KWS192	12.8	50.0	NA
2018	NUS	2018_NY_ITH	28	KWS191	21.5	90.0	NA
2018	NUS	2018_NY_ITH	29	KWS193	17.0	75.0	NA
2018	NUS	2018_NY_ITH	30	MI14R0267	20.8	80.0	NA
2018	NUS	2018_NY_ITH	31	MI14W0190	12.8	55.0	NA
2018	NUS	2018_NY_ITH	32	MI14W0906	26.5	90.0	NA
2018	NUS	2018_NY_ITH	33	MI14R1145	27.0	65.0	NA
2018	NUS	2018_NY_ITH	34	MO131753	7.0	55.0	NA
2018	NUS	2018_NY_ITH	35	MO151163	13.8	45.0	NA
2018	NUS	2018_NY_ITH	36	MO151126	6.0	20.0	NA
2018	NUS	2018_NY_ITH	37	MO160132	12.8	55.0	NA
2018	NUS	2018_NY_ITH	38	MO160140	17.8	55.0	NA
2018	NUS	2018_NY_ITH	39	0566A1-3-1-1-63	14.0	90.0	NA
2018	NUS	2018_NY_ITH	40	04620A1-1-7-4-10	15.3	85.0	NA
2018	NUS	2018_NY_ITH	41	0527A1-9-14-4-3-3	17.5	85.0	NA
2018	NUS	2018_NY_ITH	42	0762A1-2-8	12.0	80.0	NA
2018	NUS	2018_NY_ITH	43	07419A1-16-1-1-16-1-1	12.0	75.0	NA
2018	NUS	2018_NY_ITH	44	OH12-194-24	15.3	70.0	NA
2018	NUS	2018_NY_ITH	45	OH12-195-22	11.8	70.0	NA
2018	NUS	2018_NY_ITH	46	OH13-314-18	15.8	65.0	NA
2018	NUS	2018_NY_ITH	47	NE13604	14.3	60.0	NA
2018	NUS	2018_NY_ITH	48	NW13493	13.5	35.0	NA
2018	NUS	2018_NY_ITH	49	NE13515	17.5	75.0	NA

2018	NUS	2018_NY_ITH	50	NE14494	20.5	90.0	NA
2018	NUS	2018_NY_ITH	51	NE14696	8.3	75.0	NA
2018	NUS	2018_NY_ITH	52	LES168062	19.0	75.0	NA
2018	NUS	2018_NY_ITH	53	LES167062	12.8	75.0	NA
2018	NUS	2018_NY_ITH	54	LES167906	23.5	75.0	NA
2018	NUS	2018_NY_ITH	55	LES167499	26.5	75.0	NA
2018	NUS	2018_VA_MTH	1	TRUMAN	31.0	20.0	13.2
2018	NUS	2018_VA_MTH	2	ERNIE	39.0	22.5	12.8
2018	NUS	2018_VA_MTH	3	FREEDOM	49.3	30.0	13.7
2018	NUS	2018_VA_MTH	4	PIONEER2545	75.0	47.5	26.1
2018	NUS	2018_VA_MTH	5	IL13-20616	33.5	11.5	12.9
2018	NUS	2018_VA_MTH	6	IL14-11718	53.8	7.5	12.4
2018	NUS	2018_VA_MTH	7	IL14-11848	26.3	7.5	4.0
2018	NUS	2018_VA_MTH	8	IL14-28462	74.8	12.5	11.0
2018	NUS	2018_VA_MTH	9	IL14-DC-64-95-118	62.3	7.5	9.4
2018	NUS	2018_VA_MTH	10	VA15W-68	52.3	25.0	25.0
2018	NUS	2018_VA_MTH	11	VA16W-29	65.8	32.5	19.7
2018	NUS	2018_VA_MTH	12	VA16W-148	54.5	60.0	19.1
2018	NUS	2018_VA_MTH	13	VA16W-149	65.8	32.5	11.2
2018	NUS	2018_VA_MTH	14	13VA-FHB-DH252	42.3	30.0	13.2
2018	NUS	2018_VA_MTH	15	KY06C-1195-37-2-5	39.3	42.5	13.7
2018	NUS	2018_VA_MTH	16	X08C-1181-61-15-5	52.8	32.5	10.3
2018	NUS	2018_VA_MTH	17	KY06C-1178-16-10-3	28.5	37.5	13.8
2018	NUS	2018_VA_MTH	18	KY07C-1145-94-12-5	52.5	27.5	8.6
2018	NUS	2018_VA_MTH	19	X08C-1077-11-18-3	31.0	35.0	10.1
2018	NUS	2018_VA_MTH	20	NY11013-10-72-1314	57.5	50.0	17.3
2018	NUS	2018_VA_MTH	21	NY11013-10-15-1312	46.0	60.0	14.7
2018	NUS	2018_VA_MTH	22	NY02008-807	59.0	45.0	16.9
2018	NUS	2018_VA_MTH	23	NY09095-16-928	52.5	20.0	18.9
2018	NUS	2018_VA_MTH	24	NY02007-1206	74.0	52.5	23.8

2018	NUS	2018_VA_MTH	25	KWS152	46.8	37.5	12.0
2018	NUS	2018_VA_MTH	26	KWS149	51.3	45.0	10.8
2018	NUS	2018_VA_MTH	27	KWS192	44.0	21.5	12.1
2018	NUS	2018_VA_MTH	28	KWS191	51.5	35.0	20.5
2018	NUS	2018_VA_MTH	29	KWS193	44.8	30.0	18.6
2018	NUS	2018_VA_MTH	30	MI14R0267	38.5	11.5	15.3
2018	NUS	2018_VA_MTH	31	MI14W0190	26.3	10.0	21.1
2018	NUS	2018_VA_MTH	32	MI14W0906	81.0	57.5	16.0
2018	NUS	2018_VA_MTH	33	MI14R1145	57.5	27.5	11.7
2018	NUS	2018_VA_MTH	34	MO131753	28.0	22.5	12.9
2018	NUS	2018_VA_MTH	35	MO151163	26.5	15.0	10.1
2018	NUS	2018_VA_MTH	36	MO151126	28.5	11.5	9.4
2018	NUS	2018_VA_MTH	37	MO160132	34.8	12.5	13.0
2018	NUS	2018_VA_MTH	38	MO160140	30.3	12.5	13.3
2018	NUS	2018_VA_MTH	39	0566A1-3-1-1-63	59.8	40.0	16.9
2018	NUS	2018_VA_MTH	40	04620A1-1-7-4-10	65.5	20.0	18.1
2018	NUS	2018_VA_MTH	41	0527A1-9-14-4-3-3	26.8	14.0	7.9
2018	NUS	2018_VA_MTH	42	0762A1-2-8	21.3	6.5	6.6
2018	NUS	2018_VA_MTH	43	07419A1-16-1-1-16-1-1	34.8	25.0	12.1
2018	NUS	2018_VA_MTH	44	OH12-194-24	62.3	40.0	21.3
2018	NUS	2018_VA_MTH	45	OH12-195-22	63.5	35.0	15.6
2018	NUS	2018_VA_MTH	46	OH13-314-18	66.8	47.5	15.4
2018	NUS	2018_VA_MTH	47	NE13604	51.3	62.5	21.0
2018	NUS	2018_VA_MTH	48	NW13493	37.3	30.0	14.5
2018	NUS	2018_VA_MTH	49	NE13515	55.8	45.0	15.0
2018	NUS	2018_VA_MTH	50	NE14494	59.3	50.0	15.7
2018	NUS	2018_VA_MTH	51	NE14696	51.8	67.5	17.0
2018	NUS	2018_VA_MTH	52	LES168062	43.8	20.0	8.4

2018	NUS	2018_VA_MTH	53	LES167062	77.8	27.5	9.5
2018	NUS	2018_VA_MTH	54	LES167906	75.5	55.0	14.5
2018	NUS	2018_VA_MTH	55	LES167499	89.0	60.0	17.0
2018	SUS	NC18	1	ERNIE	40.0	25.0	7.4
2018	SUS	NC18	2	COKER9835	85.0	85.0	24.3
2018	SUS	NC18	3	BESS	25.0	25.0	8.9
2018	SUS	NC18	4	JAMESTOWN	40.0	12.5	4.5
2018	SUS	NC18	5	NC13-21213	65.0	32.5	8.1
2018	SUS	NC18	6	NC14-23372	30.0	20.0	7.3
2018	SUS	NC18	7	NC14-23373	25.0	17.5	6.7
2018	SUS	NC18	8	AR09006-10-2	35.0	35.0	18.8
2018	SUS	NC18	9	AR09009-8-3	25.0	27.5	20.2
2018	SUS	NC18	10	AR09045-4-2	35.0	40.0	21.4
2018	SUS	NC18	11	ARLA09218C-5-2	50.0	30.0	10.9
2018	SUS	NC18	12	ARLA09238C-6-3	45.0	30.0	10.7
2018	SUS	NC18	13	ARLA09179UC-9-3	50.0	10.0	8.3
2018	SUS	NC18	14	ARLA09137UC-17-2	50.0	30.0	8.3
2018	SUS	NC18	15	ARLW08160D-20-1	50.0	12.5	6.8
2018	SUS	NC18	16	GA13VA-FHB-DH83-17EL53	30.0	2.5	3.4
2018	SUS	NC18	17	GA091034-17EL44	80.0	42.5	20.0
2018	SUS	NC18	18	GA10654-17LE46	45.0	25.0	3.2
2018	SUS	NC18	19	GA10389-17LE56	35.0	15.0	7.5
2018	SUS	NC18	20	GA111005-17A3	55.0	40.0	11.2
2018	SUS	NC18	21	GA121086-LDH20-17A24	35.0	10.0	4.4
2018	SUS	NC18	22	GA091537-17A29	25.0	25.0	5.8
2018	SUS	NC18	23	GA1035-DH49-17LE52	15.0	7.5	2.6
2018	SUS	NC18	24	KWS154	35.0	20.0	5.1
2018	SUS	NC18	25	KWS192	35.0	20.0	8.0
2018	SUS	NC18	26	KWS193	35.0	22.5	12.7
2018	SUS	NC18	27	L11815	50.0	35.0	4.6
2018	SUS	NC18	28	L11820	50.0	25.0	3.0
2018	SUS	NC18	29	L11811	60.0	70.0	20.2
2018	SUS	NC18	30	LA14066DH-147	45.0	50.0	9.6
2018	SUS	NC18	31	LA08277C-P5-3-1	35.0	17.5	8.5
2018	SUS	NC18	32	LA11289C-57-4	45.0	40.0	11.9
2018	SUS	NC18	33	LA12120SB-56-4	35.0	27.5	7.1
2018	SUS	NC18	34	LA14076-LDH6	25.0	15.0	6.1
2018	SUS	NC18	35	LA14066DH-172	55.0	25.0	5.9
2018	SUS	NC18	36	NC14-20369	45.0	35.0	11.3
2018	SUS	NC18	37	NC14-22588	50.0	17.5	6.0
2018	SUS	NC18	38	NC11546-14	30.0	10.0	5.2
2018	SUS	NC18	39	NC15-23047	45.0	27.5	8.0
2018	SUS	NC18	40	NC15-21787	45.0	30.0	10.7
2018	SUS	NC18	41	NC11331-6	30.0	7.5	3.9
2018	SUS	NC18	42	DH12SRW057-081	40.0	15.0	6.9
2018	SUS	NC18	43	13VA-FHB-DH131	45.0	22.5	11.7

2018	SUS	NC18	44	VA15W-70	40.0	35.0	13.8
2018	SUS	NC18	45	VA16W-31	50.0	50.0	22.4
2018	SUS	NC18	46	VA16W-202	50.0	27.5	6.7
2018	SUS	NC18	47	12VTK10-156	40.0	17.5	10.6
2018	SUS	NC18	48	DH13SRW023-201	45.0	27.5	10.9
2018	SUS	NC18	49	DH13SRW025-14	45.0	15.0	16.5
2018	SUS	LA18	1	ERNIE	NA	7.5	5.5
2018	SUS	LA18	2	COKER9835	NA	60.0	16.2
2018	SUS	LA18	3	BESS	NA	5.0	3.5
2018	SUS	LA18	4	JAMESTOWN	NA	15.0	5.3
2018	SUS	LA18	5	NC13-21213	NA	37.5	13.1
2018	SUS	LA18	6	NC14-23372	NA	5.0	5.7
2018	SUS	LA18	7	NC14-23373	NA	22.5	8.5
2018	SUS	LA18	8	AR09006-10-2	NA	20.0	6.6
2018	SUS	LA18	9	AR09009-8-3	NA	17.5	7.8
2018	SUS	LA18	10	AR09045-4-2	NA	12.5	6.5
2018	SUS	LA18	11	ARLA09218C-5-2	NA	30.0	11.4
2018	SUS	LA18	12	ARLA09238C-6-3	NA	12.5	6.7
2018	SUS	LA18	13	ARLA09179UC-9-3	NA	25.0	12.8
2018	SUS	LA18	14	ARLA09137UC-17-2	NA	32.5	13.9
2018	SUS	LA18	15	ARLW08160D-20-1	NA	15.0	4.1
2018	SUS	LA18	16	GA13VA-FHB-DH83-17EL53	NA	5.0	3.1
2018	SUS	LA18	17	GA091034-17EL44	NA	67.5	27.9
2018	SUS	LA18	18	GA10654-17LE46	NA	7.5	3.6
2018	SUS	LA18	19	GA10389-17LE56	NA	17.5	4.3
2018	SUS	LA18	20	GA111005-17A3	NA	35.0	16.2
2018	SUS	LA18	21	GA121086-LDH20-17A24	NA	17.5	3.7
2018	SUS	LA18	22	GA091537-17A29	NA	10.0	3.6
2018	SUS	LA18	23	GA1035-DH49-17LE52	NA	5.0	5.4
2018	SUS	LA18	24	KWS154	NA	5.0	3.1
2018	SUS	LA18	25	KWS192	NA	20.0	3.9
2018	SUS	LA18	26	KWS193	NA	NA	2.8
2018	SUS	LA18	27	L11815	NA	10.0	3.6
2018	SUS	LA18	28	L11820	NA	12.5	4.7
2018	SUS	LA18	29	L11811	NA	30.0	13.9
2018	SUS	LA18	30	LA14066DH-147	NA	32.5	8.5
2018	SUS	LA18	31	LA08277C-P5-3-1	NA	17.5	6.5
2018	SUS	LA18	32	LA11289C-57-4	NA	30.0	7.8
2018	SUS	LA18	33	LA12120SB-56-4	NA	7.5	6.2
2018	SUS	LA18	34	LA14076-LDH6	NA	27.5	5.3
2018	SUS	LA18	35	LA14066DH-172	NA	35.0	8.6
2018	SUS	LA18	36	NC14-20369	NA	7.5	3.1
2018	SUS	LA18	37	NC14-22588	NA	15.0	3.8
2018	SUS	LA18	38	NC11546-14	NA	7.5	3.2
2018	SUS	LA18	39	NC15-23047	NA	7.5	2.1
2018	SUS	LA18	40	NC15-21787	NA	22.5	8.1
2018	SUS	LA18	41	NC11331-6	NA	7.5	3.2
2018	SUS	LA18	42	DH12SRW057-081	NA	5.0	2.4

2018	SUS	LA18	43	13VA-FHB-DH131	NA	9.0	5.1
2018	SUS	LA18	44	VA15W-70	NA	25.0	10.7
2018	SUS	LA18	45	VA16W-31	NA	15.0	10.6
2018	SUS	LA18	46	VA16W-202	NA	8.5	7.0
2018	SUS	LA18	47	12VTK10-156	NA	35.0	11.3
2018	SUS	LA18	48	DH13SRW023-201	NA	32.5	7.4
2018	SUS	LA18	49	DH13SRW025-14	NA	10.0	3.9
2018	SUS	VA18	1	ERNIE	NA	22.0	16.8
2018	SUS	VA18	2	COKER9835	NA	72.5	34.8
2018	SUS	VA18	3	BESS	NA	22.5	16.3
2018	SUS	VA18	4	JAMESTOWN	NA	41.0	13.4
2018	SUS	VA18	5	NC13-21213	NA	50.0	16.0
2018	SUS	VA18	6	NC14-23372	NA	35.0	12.1
2018	SUS	VA18	7	NC14-23373	NA	20.0	22.1
2018	SUS	VA18	8	AR09006-10-2	NA	39.0	21.8
2018	SUS	VA18	9	AR09009-8-3	NA	46.0	21.7
2018	SUS	VA18	10	AR09045-4-2	NA	54.0	33.6
2018	SUS	VA18	11	ARLA09218C-5-2	NA	35.0	20.9
2018	SUS	VA18	12	ARLA09238C-6-3	NA	41.0	12.1
2018	SUS	VA18	13	ARLA09179UC-9-3	NA	49.0	18.2
2018	SUS	VA18	14	ARLA09137UC-17-2	NA	47.0	25.2
2018	SUS	VA18	15	ARLW08160D-20-1	NA	38.0	19.0
2018	SUS	VA18	16	GA13VA-FHB-DH83-17EL53	NA	29.0	8.8
2018	SUS	VA18	17	GA091034-17EL44	NA	62.0	30.5
2018	SUS	VA18	18	GA10654-17LE46	NA	43.5	16.3
2018	SUS	VA18	19	GA10389-17LE56	NA	35.0	16.4
2018	SUS	VA18	20	GA111005-17A3	NA	57.0	23.5
2018	SUS	VA18	21	GA121086-LDH20-17A24	NA	46.5	12.7
2018	SUS	VA18	22	GA091537-17A29	NA	21.5	10.9
2018	SUS	VA18	23	GA1035-DH49-17LE52	NA	31.5	19.9
2018	SUS	VA18	24	KWS154	NA	31.5	12.3
2018	SUS	VA18	25	KWS192	NA	32.0	17.5
2018	SUS	VA18	26	KWS193	NA	29.5	16.7
2018	SUS	VA18	27	L11815	NA	37.0	14.9
2018	SUS	VA18	28	L11820	NA	40.0	15.6
2018	SUS	VA18	29	L11811	NA	41.5	21.3
2018	SUS	VA18	30	LA14066DH-147	NA	52.5	30.9
2018	SUS	VA18	31	LA08277C-P5-3-1	NA	35.5	24.4
2018	SUS	VA18	32	LA11289C-57-4	NA	73.0	17.8
2018	SUS	VA18	33	LA12120SB-56-4	NA	37.5	11.2
2018	SUS	VA18	34	LA14076-LDH6	NA	52.0	17.9
2018	SUS	VA18	35	LA14066DH-172	NA	56.5	22.1
2018	SUS	VA18	36	NC14-20369	NA	30.5	10.7
2018	SUS	VA18	37	NC14-22588	NA	35.5	17.4
2018	SUS	VA18	38	NC11546-14	NA	25.5	15.1
2018	SUS	VA18	39	NC15-23047	NA	43.0	21.3
2018	SUS	VA18	40	NC15-21787	NA	39.5	26.1
2018	SUS	VA18	41	NC11331-6	NA	26.0	11.2

2018	SUS	VA18	42	DH12SRW057-081	NA	34.5	18.0
2018	SUS	VA18	43	13VA-FHB-DH131	NA	25.0	18.9
2018	SUS	VA18	44	VA15W-70	NA	44.0	21.6
2018	SUS	VA18	45	VA16W-31	NA	37.5	32.8
2018	SUS	VA18	46	VA16W-202	NA	37.5	24.2
2018	SUS	VA18	47	12VTK10-156	NA	50.0	24.1
2018	SUS	VA18	48	DH13SRW023-201	NA	34.0	20.5
2018	SUS	VA18	49	DH13SRW025-14	NA	30.0	26.0
2018	SUS	NAR18	1	ERNIE	NA	4.0	6.8
2018	SUS	NAR18	2	COKER9835	NA	73.3	16.0
2018	SUS	NAR18	3	BESS	NA	9.3	11.1
2018	SUS	NAR18	4	JAMESTOWN	NA	40.0	14.7
2018	SUS	NAR18	5	NC13-21213	NA	41.7	16.9
2018	SUS	NAR18	6	NC14-23372	NA	33.3	19.8
2018	SUS	NAR18	7	NC14-23373	NA	28.3	20.2
2018	SUS	NAR18	8	AR09006-10-2	NA	43.3	28.4
2018	SUS	NAR18	9	AR09009-8-3	NA	43.3	27.1
2018	SUS	NAR18	10	AR09045-4-2	NA	56.7	27.7
2018	SUS	NAR18	11	ARLA09218C-5-2	NA	33.3	16.6
2018	SUS	NAR18	12	ARLA09238C-6-3	NA	55.0	19.5
2018	SUS	NAR18	13	ARLA09179UC-9-3	NA	36.7	19.0
2018	SUS	NAR18	14	ARLA09137UC-17-2	NA	48.3	23.8
2018	SUS	NAR18	15	ARLW08160D-20-1	NA	25.0	9.5
2018	SUS	NAR18	16	GA13VA-FHB-DH83-17EL53	NA	26.7	9.5
2018	SUS	NAR18	17	GA091034-17EL44	NA	79.3	19.9
2018	SUS	NAR18	18	GA10654-17LE46	NA	38.3	12.5
2018	SUS	NAR18	19	GA10389-17LE56	NA	23.3	12.2
2018	SUS	NAR18	20	GA111005-17A3	NA	61.7	22.6
2018	SUS	NAR18	21	GA121086-LDH20-17A24	NA	33.3	8.8
2018	SUS	NAR18	22	GA091537-17A29	NA	16.0	13.6
2018	SUS	NAR18	23	GA1035-DH49-17LE52	NA	16.7	7.5
2018	SUS	NAR18	24	KWS154	NA	26.7	15.9
2018	SUS	NAR18	25	KWS192	NA	28.3	14.1
2018	SUS	NAR18	26	KWS193	NA	40.0	18.1
2018	SUS	NAR18	27	L11815	NA	25.0	11.9
2018	SUS	NAR18	28	L11820	NA	15.0	8.4
2018	SUS	NAR18	29	L11811	NA	58.3	19.2
2018	SUS	NAR18	30	LA14066DH-147	NA	81.7	20.7
2018	SUS	NAR18	31	LA08277C-P5-3-1	NA	40.0	14.2
2018	SUS	NAR18	32	LA11289C-57-4	NA	65.0	21.2
2018	SUS	NAR18	33	LA12120SB-56-4	NA	31.7	12.7
2018	SUS	NAR18	34	LA14076-LDH6	NA	18.3	6.8
2018	SUS	NAR18	35	LA14066DH-172	NA	35.0	9.5
2018	SUS	NAR18	36	NC14-20369	NA	20.0	5.3
2018	SUS	NAR18	37	NC14-22588	NA	25.0	8.2
2018	SUS	NAR18	38	NC11546-14	NA	26.7	8.3
2018	SUS	NAR18	39	NC15-23047	NA	13.7	10.2
2018	SUS	NAR18	40	NC15-21787	NA	36.0	14.2

2018	SUS	NAR18	41	NC11331-6	NA	30.0	10.7
2018	SUS	NAR18	42	DH12SRW057-081	NA	22.7	6.9
2018	SUS	NAR18	43	13VA-FHB-DH131	NA	33.3	21.6
2018	SUS	NAR18	44	VA15W-70	NA	45.0	23.4
2018	SUS	NAR18	45	VA16W-31	NA	78.3	28.4
2018	SUS	NAR18	46	VA16W-202	NA	35.0	9.1
2018	SUS	NAR18	47	12VTK10-156	NA	53.3	20.4
2018	SUS	NAR18	48	DH13SRW023-201	NA	38.3	21.3
2018	SUS	NAR18	49	DH13SRW025-14	NA	41.7	18.0
2018	SUS	FAR18	1	ERNIE	NA	7.7	2.7
2018	SUS	FAR18	2	COKER9835	NA	42.7	9.0
2018	SUS	FAR18	3	BESS	NA	2.7	4.2
2018	SUS	FAR18	4	JAMESTOWN	NA	5.0	3.3
2018	SUS	FAR18	5	NC13-21213	NA	14.0	3.5
2018	SUS	FAR18	6	NC14-23372	NA	14.0	8.1
2018	SUS	FAR18	7	NC14-23373	NA	12.3	3.8
2018	SUS	FAR18	8	AR09006-10-2	NA	13.0	8.9
2018	SUS	FAR18	9	AR09009-8-3	NA	7.7	5.8
2018	SUS	FAR18	10	AR09045-4-2	NA	20.7	10.0
2018	SUS	FAR18	11	ARLA09218C-5-2	NA	7.3	3.9
2018	SUS	FAR18	12	ARLA09238C-6-3	NA	3.7	3.7
2018	SUS	FAR18	13	ARLA09179UC-9-3	NA	11.7	5.7
2018	SUS	FAR18	14	ARLA09137UC-17-2	NA	6.0	4.1
2018	SUS	FAR18	15	ARLW08160D-20-1	NA	7.7	5.6
2018	SUS	FAR18	16	GA13VA-FHB-DH83-17EL53	NA	6.0	2.7
2018	SUS	FAR18	17	GA091034-17EL44	NA	14.3	3.5
2018	SUS	FAR18	18	GA10654-17LE46	NA	5.0	3.2
2018	SUS	FAR18	19	GA10389-17LE56	NA	4.3	2.1
2018	SUS	FAR18	20	GA111005-17A3	NA	26.7	5.4
2018	SUS	FAR18	21	GA121086-LDH20-17A24	NA	10.7	2.4
2018	SUS	FAR18	22	GA091537-17A29	NA	9.7	2.6
2018	SUS	FAR18	23	GA1035-DH49-17LE52	NA	6.0	2.7
2018	SUS	FAR18	24	KWS154	NA	5.0	3.9
2018	SUS	FAR18	25	KWS192	NA	9.3	3.5
2018	SUS	FAR18	26	KWS193	NA	8.0	6.1
2018	SUS	FAR18	27	L11815	NA	6.0	2.2
2018	SUS	FAR18	28	L11820	NA	2.7	3.7
2018	SUS	FAR18	29	L11811	NA	24.3	7.3
2018	SUS	FAR18	30	LA14066DH-147	NA	46.7	7.8
2018	SUS	FAR18	31	LA08277C-P5-3-1	NA	6.3	2.6
2018	SUS	FAR18	32	LA11289C-57-4	NA	17.7	5.2
2018	SUS	FAR18	33	LA12120SB-56-4	NA	17.3	2.9
2018	SUS	FAR18	34	LA14076-LDH6	NA	4.7	2.5
2018	SUS	FAR18	35	LA14066DH-172	NA	7.3	4.2
2018	SUS	FAR18	36	NC14-20369	NA	4.3	2.5
2018	SUS	FAR18	37	NC14-22588	NA	7.0	2.8
2018	SUS	FAR18	38	NC11546-14	NA	4.3	2.1
2018	SUS	FAR18	39	NC15-23047	NA	3.7	2.7

2018	SUS	FAR18	40	NC15-21787	NA	45.0	7.4
2018	SUS	FAR18	41	NC11331-6	NA	11.7	2.4
2018	SUS	FAR18	42	DH12SRW057-081	NA	3.7	3.2
2018	SUS	FAR18	43	13VA-FHB-DH131	NA	9.3	7.9
2018	SUS	FAR18	44	VA15W-70	NA	16.7	9.7
2018	SUS	FAR18	45	VA16W-31	NA	21.7	9.2
2018	SUS	FAR18	46	VA16W-202	NA	6.7	3.8
2018	SUS	FAR18	47	12VTK10-156	NA	8.7	4.9
2018	SUS	FAR18	48	DH13SRW023-201	NA	4.3	4.6
2018	SUS	FAR18	49	DH13SRW025-14	NA	8.7	6.8
2017	SUS	NC17	1	ERNIE	14.5	NA	0.2
2017	SUS	NC17	2	COKER9835	35.0	NA	1.8
2017	SUS	NC17	3	BESS	18.5	NA	0.4
2017	SUS	NC17	4	JAMESTOWN	49.0	NA	NA
2017	SUS	NC17	5	NC13-20076	10.5	NA	0.2
2017	SUS	NC17	6	VA13W-38	13.5	NA	0.3
2017	SUS	NC17	7	ARLA07133C-19-4	31.0	NA	0.9
2017	SUS	NC17	8	ARLA07133C-3-4	17.5	NA	0.4
2017	SUS	NC17	9	ARLA06146E-1-4	12.5	NA	0.7
2017	SUS	NC17	10	AR08109-17-2	18.5	NA	0.7
2017	SUS	NC17	11	AR08015-17-4	10.0	NA	2.0
2017	SUS	NC17	12	AR08057-5-1	30.0	NA	1.1
2017	SUS	NC17	13	LES15-5369	20.0	NA	0.5
2017	SUS	NC17	14	LES15-5499	23.5	NA	1.6
2017	SUS	NC17	15	LES15-5605	37.8	NA	0.4
2017	SUS	NC17	16	GA09343-16ES3	12.0	NA	1.6
2017	SUS	NC17	17	GA09410-16ES22	30.8	NA	NA
2017	SUS	NC17	18	GA09129-16EL56	13.5	NA	1.1
2017	SUS	NC17	19	GA121176-16JS49	40.0	NA	NA
2017	SUS	NC17	20	GA09163-16ES19	11.0	NA	1.1
2017	SUS	NC17	21	GA09144-16ES23	13.0	NA	0.2
2017	SUS	NC17	22	GA05450-16ES8	32.5	NA	1.6
2017	SUS	NC17	23	GA09054-16ES25	12.5	NA	0.4
2017	SUS	NC17	24	KWS095	17.5	NA	1.2
2017	SUS	NC17	25	KWS103	21.0	NA	0.9
2017	SUS	NC17	26	KWS114	13.5	NA	2.3
2017	SUS	NC17	27	KWS122	37.8	NA	0.1
2017	SUS	NC17	28	KWS133	27.5	NA	1.8
2017	SUS	NC17	29	KWS141	20.0	NA	1.7
2017	SUS	NC17	30	LA08265C-50	17.5	NA	0.3
2017	SUS	NC17	31	LA09101UB-48-3-5	15.0	NA	0.2
2017	SUS	NC17	32	LW08049C-74-2-5	31.5	NA	0.4
2017	SUS	NC17	33	LA10081C-18	22.5	NA	0.6
2017	SUS	NC17	34	LA11309GS-16	17.0	NA	NA
2017	SUS	NC17	35	NC13-23443	15.0	NA	0.3
2017	SUS	NC17	36	NC13-21213	7.8	NA	1.0
2017	SUS	NC17	37	NC13-20332	22.5	NA	1.8
2017	SUS	NC17	38	NC14-23372	22.5	NA	0.4
2017	SUS	NC17	39	NC14-23373	21.5	NA	0.3

2017	SUS	NC17	40	VA13W-174	11.0	NA	0.4
2017	SUS	NC17	41	VA09MAS2-131-6-2	20.0	NA	0.7
2017	SUS	NC17	42	VA14W-32	12.8	NA	NA
2017	SUS	NC17	43	VA07MAS1-7047-1-1-4-2	17.5	NA	0.3
2017	SUS	NC17	44	DH12SRW056-058	10.0	NA	0.7
2017	SUS	NC17	45	DH11SRW061-16	27.8	NA	2.3
2017	SUS	NC17	46	VA09MAS2-131-6-2-4	7.5	NA	0.9
2017	SUS	NAR17	1	ERNIE	42.0	11.0	1.7
2017	SUS	NAR17	2	COKER9835	100.0	97.0	5.7
2017	SUS	NAR17	3	BESS	7.0	7.0	2.2
2017	SUS	NAR17	4	JAMESTOWN	5.0	6.0	0.9
2017	SUS	NAR17	5	NC13-20076	3.0	4.0	0.7
2017	SUS	NAR17	6	VA13W-38	12.0	58.0	1.8
2017	SUS	NAR17	7	ARLA07133C-19-4	5.0	13.0	1.7
2017	SUS	NAR17	8	ARLA07133C-3-4	15.0	8.0	1.6
2017	SUS	NAR17	9	ARLA06146E-1-4	7.0	24.0	1.9
2017	SUS	NAR17	10	AR08109-17-2	13.0	7.0	2.1
2017	SUS	NAR17	11	AR08015-17-4	18.0	35.0	4.8
2017	SUS	NAR17	12	AR08057-5-1	20.0	65.0	5.0
2017	SUS	NAR17	13	LES15-5369	15.0	30.0	2.1
2017	SUS	NAR17	14	LES15-5499	15.0	28.0	2.9
2017	SUS	NAR17	15	LES15-5605	18.0	40.0	3.5
2017	SUS	NAR17	16	GA09343-16ES3	22.0	60.0	3.9
2017	SUS	NAR17	17	GA09410-16ES22	37.0	33.0	2.3
2017	SUS	NAR17	18	GA09129-16EL56	10.0	17.0	1.1
2017	SUS	NAR17	19	GA121176-16JS49	12.0	22.0	1.0
2017	SUS	NAR17	20	GA09163-16ES19	5.0	15.0	2.6
2017	SUS	NAR17	21	GA09144-16ES23	10.0	23.0	1.1
2017	SUS	NAR17	22	GA05450-16ES8	25.0	57.0	3.8
2017	SUS	NAR17	23	GA09054-16ES25	13.0	15.0	1.1
2017	SUS	NAR17	24	KWS095	8.0	47.0	4.0
2017	SUS	NAR17	25	KWS103	7.0	12.0	2.2
2017	SUS	NAR17	26	KWS114	7.0	37.0	5.0
2017	SUS	NAR17	27	KWS122	7.0	5.0	1.1
2017	SUS	NAR17	28	KWS133	12.0	47.0	4.9
2017	SUS	NAR17	29	KWS141	7.0	22.0	2.9
2017	SUS	NAR17	30	LA08265C-50	12.0	10.0	1.5
2017	SUS	NAR17	31	LA09101UB-48-3-5	8.0	22.0	1.4
2017	SUS	NAR17	32	LW08049C-74-2-5	7.0	5.0	0.6
2017	SUS	NAR17	33	LA10081C-18	3.0	7.0	1.5
2017	SUS	NAR17	34	LA11309GS-16	18.0	13.0	0.6
2017	SUS	NAR17	35	NC13-23443	80.0	47.0	2.5
2017	SUS	NAR17	36	NC13-21213	17.0	37.0	2.3
2017	SUS	NAR17	37	NC13-20332	18.0	35.0	2.9
2017	SUS	NAR17	38	NC14-23372	12.0	17.0	3.1
2017	SUS	NAR17	39	NC14-23373	5.0	8.0	1.5
2017	SUS	NAR17	40	VA13W-174	17.0	62.0	1.2
2017	SUS	NAR17	41	VA09MAS2-131-6-2	20.0	35.0	3.0
2017	SUS	NAR17	42	VA14W-32	20.0	12.0	2.3

2017	SUS	NAR17	43	VA07MAS1-7047-1-1-4-2	15.0	58.0	1.4
2017	SUS	NAR17	44	DH12SRW056-058	8.0	8.0	2.2
2017	SUS	NAR17	45	DH11SRW061-16	17.0	8.0	1.4
2017	SUS	NAR17	46	VA09MAS2-131-6-2-4	20.0	48.0	4.4
2017	SUS	KY17	1	ERNIE	23.0	30.0	17.1
2017	SUS	KY17	2	COKER9835	41.0	63.0	23.8
2017	SUS	KY17	3	BESS	48.0	18.0	17.5
2017	SUS	KY17	4	JAMESTOWN	31.0	30.0	14.9
2017	SUS	KY17	5	NC13-20076	54.0	25.0	13.7
2017	SUS	KY17	6	VA13W-38	48.0	25.0	14.8
2017	SUS	KY17	7	ARLA07133C-19-4	40.0	35.0	22.7
2017	SUS	KY17	8	ARLA07133C-3-4	27.0	45.0	18.8
2017	SUS	KY17	9	ARLA06146E-1-4	44.0	28.0	19.2
2017	SUS	KY17	10	AR08109-17-2	58.0	38.0	18.0
2017	SUS	KY17	11	AR08015-17-4	29.0	50.0	20.4
2017	SUS	KY17	12	AR08057-5-1	34.0	45.0	18.2
2017	SUS	KY17	13	LES15-5369	47.0	43.0	16.4
2017	SUS	KY17	14	LES15-5499	45.0	38.0	13.5
2017	SUS	KY17	15	LES15-5605	26.0	40.0	22.1
2017	SUS	KY17	16	GA09343-16ES3	43.0	70.0	29.1
2017	SUS	KY17	17	GA09410-16ES22	39.0	53.0	26.8
2017	SUS	KY17	18	GA09129-16EL56	42.0	33.0	21.0
2017	SUS	KY17	19	GA121176-16JS49	39.0	30.0	21.3
2017	SUS	KY17	20	GA09163-16ES19	48.0	35.0	32.1
2017	SUS	KY17	21	GA09144-16ES23	49.0	35.0	17.5
2017	SUS	KY17	22	GA05450-16ES8	31.0	68.0	33.0
2017	SUS	KY17	23	GA09054-16ES25	47.0	55.0	24.0
2017	SUS	KY17	24	KWS095	33.0	38.0	16.3
2017	SUS	KY17	25	KWS103	37.0	45.0	14.0
2017	SUS	KY17	26	KWS114	61.0	45.0	16.5
2017	SUS	KY17	27	KWS122	51.0	28.0	8.9
2017	SUS	KY17	28	KWS133	35.0	40.0	19.3
2017	SUS	KY17	29	KWS141	57.0	28.0	14.5
2017	SUS	KY17	30	LA08265C-50	57.0	30.0	14.6
2017	SUS	KY17	31	LA09101UB-48-3-5	29.0	25.0	17.7
2017	SUS	KY17	32	LW08049C-74-2-5	61.0	35.0	18.4
2017	SUS	KY17	33	LA10081C-18	43.0	58.0	29.3
2017	SUS	KY17	34	LA11309GS-16	42.0	28.0	12.9
2017	SUS	KY17	35	NC13-23443	52.0	20.0	14.7
2017	SUS	KY17	36	NC13-21213	35.0	50.0	14.6
2017	SUS	KY17	37	NC13-20332	42.0	63.0	32.2
2017	SUS	KY17	38	NC14-23372	52.0	38.0	16.2
2017	SUS	KY17	39	NC14-23373	34.0	28.0	22.9
2017	SUS	KY17	40	VA13W-174	32.0	25.0	16.1
2017	SUS	KY17	41	VA09MAS2-131-6-2	30.0	25.0	24.9
2017	SUS	KY17	42	VA14W-32	56.0	55.0	27.3
2017	SUS	KY17	43	VA07MAS1-7047-1-1-4-2	40.0	23.0	24.6
2017	SUS	KY17	44	DH12SRW056-058	44.0	35.0	21.5

2017	SUS	KY17	45	DH11SRW061-16	62.0	20.0	14.4
2017	SUS	KY17	46	VA09MAS2-131-6-2-4	30.0	43.0	25.9
2017	SUS	LA17	1	ERNIE	10.0	10.0	11.0
2017	SUS	LA17	2	COKER9835	50.0	85.0	13.0
2017	SUS	LA17	3	BESS	28.0	10.0	6.0
2017	SUS	LA17	4	JAMESTOWN	15.0	15.0	6.0
2017	SUS	LA17	5	NC13-20076	18.0	35.0	8.0
2017	SUS	LA17	6	VA13W-38	20.0	25.0	14.0
2017	SUS	LA17	7	ARLA07133C-19-4	5.0	35.0	16.0
2017	SUS	LA17	8	ARLA07133C-3-4	23.0	35.0	13.0
2017	SUS	LA17	9	ARLA06146E-1-4	15.0	25.0	8.0
2017	SUS	LA17	10	AR08109-17-2	18.0	30.0	16.0
2017	SUS	LA17	11	AR08015-17-4	18.0	40.0	25.0
2017	SUS	LA17	12	AR08057-5-1	28.0	35.0	11.0
2017	SUS	LA17	13	LES15-5369	5.0	35.0	5.0
2017	SUS	LA17	14	LES15-5499	NA	35.0	16.0
2017	SUS	LA17	15	LES15-5605	20.0	30.0	21.0
2017	SUS	LA17	16	GA09343-16ES3	23.0	65.0	23.0
2017	SUS	LA17	17	GA09410-16ES22	18.0	35.0	26.0
2017	SUS	LA17	18	GA09129-16EL56	15.0	30.0	13.0
2017	SUS	LA17	19	GA121176-16JS49	33.0	75.0	15.0
2017	SUS	LA17	20	GA09163-16ES19	25.0	45.0	26.0
2017	SUS	LA17	21	GA09144-16ES23	18.0	30.0	27.0
2017	SUS	LA17	22	GA05450-16ES8	23.0	40.0	23.0
2017	SUS	LA17	23	GA09054-16ES25	28.0	35.0	17.0
2017	SUS	LA17	24	KWS095	15.0	35.0	8.0
2017	SUS	LA17	25	KWS103	10.0	20.0	9.0
2017	SUS	LA17	26	KWS114	60.0	30.0	25.0
2017	SUS	LA17	27	KWS122	18.0	30.0	17.0
2017	SUS	LA17	28	KWS133	15.0	40.0	22.0
2017	SUS	LA17	29	KWS141	33.0	30.0	19.0
2017	SUS	LA17	30	LA08265C-50	23.0	40.0	13.0
2017	SUS	LA17	31	LA09101UB-48-3-5	13.0	25.0	5.0
2017	SUS	LA17	32	LW08049C-74-2-5	15.0	25.0	12.0
2017	SUS	LA17	33	LA10081C-18	20.0	40.0	20.0
2017	SUS	LA17	34	LA11309GS-16	18.0	40.0	9.0
2017	SUS	LA17	35	NC13-23443	18.0	25.0	7.0
2017	SUS	LA17	36	NC13-21213	23.0	30.0	12.0
2017	SUS	LA17	37	NC13-20332	23.0	20.0	8.0
2017	SUS	LA17	38	NC14-23372	18.0	40.0	17.0
2017	SUS	LA17	39	NC14-23373	15.0	35.0	27.0
2017	SUS	LA17	40	VA13W-174	18.0	20.0	16.0
2017	SUS	LA17	41	VA09MAS2-131-6-2	35.0	40.0	18.0
2017	SUS	LA17	42	VA14W-32	50.0	70.0	36.0
2017	SUS	LA17	43	VA07MAS1-7047-1-1-4-2	15.0	40.0	28.0
2017	SUS	LA17	44	DH12SRW056-058	20.0	30.0	25.0
2017	SUS	LA17	45	DH11SRW061-16	40.0	40.0	21.0
2017	SUS	LA17	46	VA09MAS2-131-6-2-4	18.0	45.0	41.0
2017	SUS	MO17	1	ERNIE	13.0	8.0	NA

2017	SUS	MO17	2	COKER9835	22.0	30.0	NA
2017	SUS	MO17	3	BESS	9.0	10.0	NA
2017	SUS	MO17	4	JAMESTOWN	9.0	8.0	NA
2017	SUS	MO17	5	NC13-20076	14.0	8.0	NA
2017	SUS	MO17	6	VA13W-38	10.0	8.0	NA
2017	SUS	MO17	7	ARLA07133C-19-4	8.0	8.0	NA
2017	SUS	MO17	8	ARLA07133C-3-4	17.0	10.0	NA
2017	SUS	MO17	9	ARLA06146E-1-4	15.0	18.0	NA
2017	SUS	MO17	10	AR08109-17-2	13.0	10.0	NA
2017	SUS	MO17	11	AR08015-17-4	15.0	10.0	NA
2017	SUS	MO17	12	AR08057-5-1	10.0	5.0	NA
2017	SUS	MO17	13	LES15-5369	10.0	13.0	NA
2017	SUS	MO17	14	LES15-5499	11.0	10.0	NA
2017	SUS	MO17	15	LES15-5605	14.0	5.0	NA
2017	SUS	MO17	16	GA09343-16ES3	13.0	15.0	NA
2017	SUS	MO17	17	GA09410-16ES22	11.0	25.0	NA
2017	SUS	MO17	18	GA09129-16EL56	10.0	10.0	NA
2017	SUS	MO17	19	GA121176-16JS49	8.0	20.0	NA
2017	SUS	MO17	20	GA09163-16ES19	8.0	8.0	NA
2017	SUS	MO17	21	GA09144-16ES23	18.0	13.0	NA
2017	SUS	MO17	22	GA05450-16ES8	12.0	10.0	NA
2017	SUS	MO17	23	GA09054-16ES25	13.0	25.0	NA
2017	SUS	MO17	24	KWS095	13.0	20.0	NA
2017	SUS	MO17	25	KWS103	10.0	8.0	NA
2017	SUS	MO17	26	KWS114	10.0	10.0	NA
2017	SUS	MO17	27	KWS122	12.0	5.0	NA
2017	SUS	MO17	28	KWS133	13.0	18.0	NA
2017	SUS	MO17	29	KWS141	11.0	5.0	NA
2017	SUS	MO17	30	LA08265C-50	14.0	15.0	NA
2017	SUS	MO17	31	LA09101UB-48-3-5	10.0	8.0	NA
2017	SUS	MO17	32	LW08049C-74-2-5	10.0	8.0	NA
2017	SUS	MO17	33	LA10081C-18	17.0	15.0	NA
2017	SUS	MO17	34	LA11309GS-16	16.0	13.0	NA
2017	SUS	MO17	35	NC13-23443	9.0	8.0	NA
2017	SUS	MO17	36	NC13-21213	17.0	40.0	NA
2017	SUS	MO17	37	NC13-20332	18.0	13.0	NA
2017	SUS	MO17	38	NC14-23372	15.0	10.0	NA
2017	SUS	MO17	39	NC14-23373	16.0	5.0	NA
2017	SUS	MO17	40	VA13W-174	7.0	8.0	NA
2017	SUS	MO17	41	VA09MAS2-131-6-2	10.0	10.0	NA
2017	SUS	MO17	42	VA14W-32	19.0	8.0	NA
2017	SUS	MO17	43	VA07MAS1-7047-1-1-4-2	10.0	10.0	NA
2017	SUS	MO17	44	DH12SRW056-058	14.0	5.0	NA
2017	SUS	MO17	45	DH11SRW061-16	10.0	13.0	NA
2017	SUS	MO17	46	VA09MAS2-131-6-2-4	21.0	13.0	NA
2017	SUS	VA17	1	ERNIE	12.0	18.0	0.6
2017	SUS	VA17	2	COKER9835	44.0	46.0	3.2
2017	SUS	VA17	3	BESS	18.0	18.0	1.1
2017	SUS	VA17	4	JAMESTOWN	10.0	25.0	0.8

2017	SUS	VA17	5	NC13-20076	11.0	18.0	0.4
2017	SUS	VA17	6	VA13W-38	16.0	27.0	1.4
2017	SUS	VA17	7	ARLA07133C-19-4	33.0	27.5	1.7
2017	SUS	VA17	8	ARLA07133C-3-4	40.0	31.0	2.2
2017	SUS	VA17	9	ARLA06146E-1-4	9.0	15.5	0.8
2017	SUS	VA17	10	AR08109-17-2	38.0	26.5	1.8
2017	SUS	VA17	11	AR08015-17-4	36.0	32.0	2.2
2017	SUS	VA17	12	AR08057-5-1	35.0	28.0	2.9
2017	SUS	VA17	13	LES15-5369	16.0	23.0	1.3
2017	SUS	VA17	14	LES15-5499	52.0	18.5	1.8
2017	SUS	VA17	15	LES15-5605	40.0	24.5	0.9
2017	SUS	VA17	16	GA09343-16ES3	39.0	51.0	3.1
2017	SUS	VA17	17	GA09410-16ES22	29.0	38.5	5.3
2017	SUS	VA17	18	GA09129-16EL56	21.0	29.5	1.6
2017	SUS	VA17	19	GA121176-16JS49	27.0	49.0	3.4
2017	SUS	VA17	20	GA09163-16ES19	30.0	19.5	5.6
2017	SUS	VA17	21	GA09144-16ES23	21.0	40.0	1.6
2017	SUS	VA17	22	GA05450-16ES8	26.0	33.0	2.6
2017	SUS	VA17	23	GA09054-16ES25	18.0	31.0	1.1
2017	SUS	VA17	24	KWS095	34.0	33.0	1.6
2017	SUS	VA17	25	KWS103	38.0	38.5	1.7
2017	SUS	VA17	26	KWS114	47.0	37.0	2.8
2017	SUS	VA17	27	KWS122	24.0	16.0	0.8
2017	SUS	VA17	28	KWS133	46.0	23.5	2.2
2017	SUS	VA17	29	KWS141	17.0	20.5	1.5
2017	SUS	VA17	30	LA08265C-50	15.0	31.0	0.9
2017	SUS	VA17	31	LA09101UB-48-3-5	19.0	25.0	1.0
2017	SUS	VA17	32	LW08049C-74-2-5	47.0	21.5	1.6
2017	SUS	VA17	33	LA10081C-18	14.0	25.5	1.3
2017	SUS	VA17	34	LA11309GS-16	10.0	29.0	1.1
2017	SUS	VA17	35	NC13-23443	21.0	27.5	0.8
2017	SUS	VA17	36	NC13-21213	42.0	29.0	1.4
2017	SUS	VA17	37	NC13-20332	29.0	34.0	1.6
2017	SUS	VA17	38	NC14-23372	13.0	23.0	0.7
2017	SUS	VA17	39	NC14-23373	13.0	27.0	0.7
2017	SUS	VA17	40	VA13W-174	12.0	34.5	0.8
2017	SUS	VA17	41	VA09MAS2-131-6-2	22.0	35.5	1.5
2017	SUS	VA17	42	VA14W-32	31.0	33.0	1.7
2017	SUS	VA17	43	VA07MAS1-7047-1-1-4-2	11.0	27.5	1.2
2017	SUS	VA17	44	DH12SRW056-058	18.0	31.0	2.5
2017	SUS	VA17	45	DH11SRW061-16	16.0	27.0	2.4
2017	SUS	VA17	46	VA09MAS2-131-6-2-4	26.0	29.5	1.1
2016	SUS	VA16	1	ERNIE	9.7	48.0	1.8
2016	SUS	VA16	2	COKER9835	39.1	63.0	7.7
2016	SUS	VA16	3	BESS	7.0	31.0	2.3
2016	SUS	VA16	4	JAMESTOWN	14.6	37.0	2.1
2016	SUS	VA16	5	AR06024-7-2	8.2	24.0	1.4
2016	SUS	VA16	6	ARS10-389	6.2	38.0	0.7
2016	SUS	VA16	7	AR07010-7-1	11.4	32.0	3.3

2016	SUS	VA16	8	AR07053-13-1	11.7	28.0	3.1
2016	SUS	VA16	9	AR07078-7-4	24.8	30.0	3.8
2016	SUS	VA16	10	AR07108-6-1	8.4	25.0	1.7
2016	SUS	VA16	11	ARLA06146E-20-1	10.5	31.0	1.8
2016	SUS	VA16	12	ARLA07084C-10-1	9.3	39.0	3.4
2016	SUS	VA16	13	ARS11-2086	15.9	45.0	4.3
2016	SUS	VA16	14	ARS12-201	10.7	32.0	2.9
2016	SUS	VA16	15	ARS13-159	9.3	56.0	1.1
2016	SUS	VA16	16	ARS13-215	39.1	49.0	13.4
2016	SUS	VA16	17	ARS14W0539	12.5	52.0	5.7
2016	SUS	VA16	18	ARS14W0623	14.3	61.0	4.6
2016	SUS	VA16	19	ARS14W1012	11.4	72.0	3.0
2016	SUS	VA16	20	ES14-0057	11.8	25.0	1.8
2016	SUS	VA16	21	ES14-0528	10.5	42.0	1.3
2016	SUS	VA16	22	ES14-1293	18.4	27.0	1.7
2016	SUS	VA16	23	ES14-1350	14.5	24.0	0.9
2016	SUS	VA16	24	GA08250-15ES14	11.0	21.0	2.2
2016	SUS	VA16	25	GA08293-15ES3	22.9	50.0	4.4
2016	SUS	VA16	26	GA09361-15ES38	31.7	52.0	5.6
2016	SUS	VA16	27	GA091252-15ES35	14.0	39.0	7.0
2016	SUS	VA16	28	GA08281-15ES1	20.4	38.0	5.1
2016	SUS	VA16	29	GANC9337-15ES27	7.0	23.0	1.5
2016	SUS	VA16	30	GA09343-15ES33	17.8	43.0	5.9
2016	SUS	VA16	31	GANC10014-15ES24	20.1	63.0	4.8
2016	SUS	VA16	32	KWS053	9.9	26.0	3.4
2016	SUS	VA16	33	KWS060	20.7	54.0	0.9
2016	SUS	VA16	34	KWS074	15.4	35.0	2.9
2016	SUS	VA16	35	KWS081	10.5	47.0	1.7
2016	SUS	VA16	36	KWS083	13.0	14.0	3.6
2016	SUS	VA16	37	KWS087	9.8	35.0	2.2
2016	SUS	VA16	38	LA06146E-P4	10.6	42.0	5.8
2016	SUS	VA16	39	LA08090C-9-2	15.1	28.0	4.7
2016	SUS	VA16	40	LA08265C-50	24.9	44.0	3.1
2016	SUS	VA16	41	LA09011UB-2	20.6	51.0	4.0
2016	SUS	VA16	42	LA09225C-33	33.3	55.0	4.6
2016	SUS	VA16	43	NC10435-11	15.8	19.0	2.9
2016	SUS	VA16	44	NC12-22225	11.1	24.0	1.9
2016	SUS	VA16	45	NC13-20076	8.5	12.0	1.0
2016	SUS	VA16	46	NC13-22350	9.5	31.0	1.3
2016	SUS	VA16	47	NC13-23449	14.0	18.0	2.9
2016	SUS	VA16	48	VA12W-68	17.8	44.0	7.3
2016	SUS	VA16	49	VA13W-38	15.0	26.0	1.7
2016	SUS	VA16	50	VA09MAS6-122-7-1	25.0	28.0	2.6
2016	SUS	VA16	51	VA08MAS1-188-6-4-1	13.4	42.0	2.1
2016	SUS	VA16	52	VA13FHB-26	18.3	32.0	3.4
2016	SUS	VA16	53	VA14FHB-14	12.2	20.0	1.4
2016	SUS	VA16	54	VA14FHB-13	10.9	26.0	2.6
2016	SUS	VA16	55	VA14FHB-28	32.5	40.0	4.6
2016	SUS	FAR16	1	ERNIE	43.3	26.7	1.9

2016	SUS	FAR16	2	COKER9835	95.0	45.0	2.8
2016	SUS	FAR16	3	BESS	6.7	1.0	2.0
2016	SUS	FAR16	4	JAMESTOWN	NA	1.3	1.4
2016	SUS	FAR16	5	AR06024-7-2	1.7	1.3	2.1
2016	SUS	FAR16	6	ARS10-389	1.7	1.3	1.0
2016	SUS	FAR16	7	AR07010-7-1	1.7	2.3	5.7
2016	SUS	FAR16	8	AR07053-13-1	5.0	13.3	7.6
2016	SUS	FAR16	9	AR07078-7-4	3.3	8.0	6.9
2016	SUS	FAR16	10	AR07108-6-1	3.3	2.7	5.6
2016	SUS	FAR16	11	ARLA06146E-20-1	6.7	14.0	4.2
2016	SUS	FAR16	12	ARLA07084C-10-1	3.3	1.3	3.3
2016	SUS	FAR16	13	ARS11-2086	98.3	78.3	3.0
2016	SUS	FAR16	14	ARS12-201	98.3	76.7	4.1
2016	SUS	FAR16	15	ARS13-159	46.7	83.3	1.5
2016	SUS	FAR16	16	ARS13-215	1.7	2.7	7.7
2016	SUS	FAR16	17	ARS14W0539	NA	1.7	3.4
2016	SUS	FAR16	18	ARS14W0623	16.7	66.7	10.5
2016	SUS	FAR16	19	ARS14W1012	3.3	6.3	2.0
2016	SUS	FAR16	20	ES14-0057	21.7	37.7	3.6
2016	SUS	FAR16	21	ES14-0528	43.3	60.0	1.7
2016	SUS	FAR16	22	ES14-1293	1.7	4.0	3.1
2016	SUS	FAR16	23	ES14-1350	78.3	85.0	2.0
2016	SUS	FAR16	24	GA08250-15ES14	NA	0.7	1.0
2016	SUS	FAR16	25	GA08293-15ES3	NA	1.7	0.8
2016	SUS	FAR16	26	GA09361-15ES38	NA	2.7	2.6
2016	SUS	FAR16	27	GA091252-15ES35	20.0	5.7	1.6
2016	SUS	FAR16	28	GA08281-15ES1	5.0	4.0	1.9
2016	SUS	FAR16	29	GANC9337-15ES27	NA	1.0	0.5
2016	SUS	FAR16	30	GA09343-15ES33	5.0	2.3	2.9
2016	SUS	FAR16	31	GANC10014-15ES24	3.3	6.0	2.7
2016	SUS	FAR16	32	KWS053	1.7	3.7	0.6
2016	SUS	FAR16	33	KWS060	5.0	11.0	1.6
2016	SUS	FAR16	34	KWS074	16.7	63.3	2.4
2016	SUS	FAR16	35	KWS081	3.3	26.7	1.4
2016	SUS	FAR16	36	KWS083	10.0	31.7	4.3
2016	SUS	FAR16	37	KWS087	8.3	20.7	2.5
2016	SUS	FAR16	38	LA06146E-P4	NA	1.0	1.0
2016	SUS	FAR16	39	LA08090C-9-2	3.3	5.0	2.7
2016	SUS	FAR16	40	LA08265C-50	1.7	0.7	0.9
2016	SUS	FAR16	41	LA09011UB-2	1.7	5.0	2.1
2016	SUS	FAR16	42	LA09225C-33	5.0	6.7	3.5
2016	SUS	FAR16	43	NC10435-11	NA	3.0	1.7
2016	SUS	FAR16	44	NC12-22225	35.0	50.0	2.3
2016	SUS	FAR16	45	NC13-20076	NA	0.3	0.9
2016	SUS	FAR16	46	NC13-22350	21.7	37.7	1.5
2016	SUS	FAR16	47	NC13-23449	93.3	77.5	2.8
2016	SUS	FAR16	48	VA12W-68	NA	4.0	2.6
2016	SUS	FAR16	49	VA13W-38	33.3	36.7	1.4
2016	SUS	FAR16	50	VA09MAS6-122-7-1	1.7	1.0	2.0

2016	SUS	FAR16	51	VA08MAS1-188-6-4-1	8.3	7.7	2.0
2016	SUS	FAR16	52	VA13FHB-26	50.0	76.7	1.9
2016	SUS	FAR16	53	VA14FHB-14	96.7	80.0	1.3
2016	SUS	FAR16	54	VA14FHB-13	96.7	85.0	1.6
2016	SUS	FAR16	55	VA14FHB-28	3.3	6.7	2.1
2016	SUS	NAR16	1	ERNIE	13.3	34.0	7.0
2016	SUS	NAR16	2	COKER9835	38.3	80.0	14.0
2016	SUS	NAR16	3	BESS	3.3	3.0	4.0
2016	SUS	NAR16	4	JAMESTOWN	5.0	33.7	8.0
2016	SUS	NAR16	5	AR06024-7-2	5.0	19.0	5.0
2016	SUS	NAR16	6	ARS10-389	3.3	17.7	5.0
2016	SUS	NAR16	7	AR07010-7-1	5.0	26.0	10.0
2016	SUS	NAR16	8	AR07053-13-1	8.3	41.7	13.0
2016	SUS	NAR16	9	AR07078-7-4	6.7	30.0	11.0
2016	SUS	NAR16	10	AR07108-6-1	NA	6.0	6.0
2016	SUS	NAR16	11	ARLA06146E-20-1	NA	35.0	7.0
2016	SUS	NAR16	12	ARLA07084C-10-1	5.0	18.3	9.0
2016	SUS	NAR16	13	ARS11-2086	5.0	63.3	11.0
2016	SUS	NAR16	14	ARS12-201	5.0	48.3	11.0
2016	SUS	NAR16	15	ARS13-159	3.3	46.7	8.0
2016	SUS	NAR16	16	ARS13-215	6.7	43.3	26.0
2016	SUS	NAR16	17	ARS14W0539	3.3	53.3	9.0
2016	SUS	NAR16	18	ARS14W0623	NA	73.3	26.0
2016	SUS	NAR16	19	ARS14W1012	5.0	85.0	9.0
2016	SUS	NAR16	20	ES14-0057	3.3	10.0	6.0
2016	SUS	NAR16	21	ES14-0528	6.7	30.0	6.0
2016	SUS	NAR16	22	ES14-1293	3.3	9.3	5.0
2016	SUS	NAR16	23	ES14-1350	NA	21.7	4.0
2016	SUS	NAR16	24	GA08250-15ES14	1.7	9.3	7.0
2016	SUS	NAR16	25	GA08293-15ES3	16.7	48.7	8.0
2016	SUS	NAR16	26	GA09361-15ES38	13.3	36.7	14.0
2016	SUS	NAR16	27	GA091252-15ES35	6.7	43.3	10.0
2016	SUS	NAR16	28	GA08281-15ES1	6.7	21.7	7.0
2016	SUS	NAR16	29	GANC9337-15ES27	3.3	18.3	6.0
2016	SUS	NAR16	30	GA09343-15ES33	8.3	48.3	9.0
2016	SUS	NAR16	31	GANC10014-15ES24	3.3	53.3	10.0
2016	SUS	NAR16	32	KWS053	1.7	15.7	4.0
2016	SUS	NAR16	33	KWS060	5.0	8.0	5.0
2016	SUS	NAR16	34	KWS074	3.3	18.3	9.0
2016	SUS	NAR16	35	KWS081	3.3	5.7	3.0
2016	SUS	NAR16	36	KWS083	3.3	16.7	8.0
2016	SUS	NAR16	37	KWS087	1.7	26.7	4.0
2016	SUS	NAR16	38	LA06146E-P4	10.0	51.7	10.0
2016	SUS	NAR16	39	LA08090C-9-2	8.3	48.3	14.0
2016	SUS	NAR16	40	LA08265C-50	6.7	36.7	8.0
2016	SUS	NAR16	41	LA09011UB-2	18.3	61.7	13.0
2016	SUS	NAR16	42	LA09225C-33	8.3	40.0	11.0
2016	SUS	NAR16	43	NC10435-11	15.0	33.3	5.0
2016	SUS	NAR16	44	NC12-22225	3.3	10.0	5.0

2016	SUS	NAR16	45	NC13-20076	5.0	6.7	3.0
2016	SUS	NAR16	46	NC13-22350	3.3	10.0	3.0
2016	SUS	NAR16	47	NC13-23449	8.3	31.7	7.0
2016	SUS	NAR16	48	VA12W-68	6.7	56.7	16.0
2016	SUS	NAR16	49	VA13W-38	11.7	18.3	6.0
2016	SUS	NAR16	50	VA09MAS6-122-7-1	11.7	7.7	6.0
2016	SUS	NAR16	51	VA08MAS1-188-6-4-1	13.3	28.3	7.0
2016	SUS	NAR16	52	VA13FHB-26	3.3	76.7	7.0
2016	SUS	NAR16	53	VA14FHB-14	63.3	85.0	6.0
2016	SUS	NAR16	54	VA14FHB-13	65.0	80.3	7.0
2016	SUS	NAR16	55	VA14FHB-28	25.0	58.3	9.0
2016	SUS	IL16	1	ERNIE	53.4	16.7	2.2
2016	SUS	IL16	2	COKER9835	80.9	46.7	5.0
2016	SUS	IL16	3	BESS	49.3	21.7	0.5
2016	SUS	IL16	4	JAMESTOWN	37.2	33.3	2.6
2016	SUS	IL16	5	AR06024-7-2	47.9	70.0	1.5
2016	SUS	IL16	6	ARS10-389	15.8	26.7	1.2
2016	SUS	IL16	7	AR07010-7-1	51.1	36.7	1.2
2016	SUS	IL16	8	AR07053-13-1	45.5	13.3	2.1
2016	SUS	IL16	9	AR07078-7-4	74.5	50.0	2.7
2016	SUS	IL16	10	AR07108-6-1	62.3	30.0	1.2
2016	SUS	IL16	11	ARLA06146E-20-1	71.0	30.0	1.0
2016	SUS	IL16	12	ARLA07084C-10-1	NA	NA	NA
2016	SUS	IL16	13	ARS11-2086	30.7	63.3	1.3
2016	SUS	IL16	14	ARS12-201	37.6	35.0	1.5
2016	SUS	IL16	15	ARS13-159	67.5	20.0	0.8
2016	SUS	IL16	16	ARS13-215	NA	NA	NA
2016	SUS	IL16	17	ARS14W0539	15.7	66.7	2.2
2016	SUS	IL16	18	ARS14W0623	50.3	6.1	1.5
2016	SUS	IL16	19	ARS14W1012	67.4	36.7	3.8
2016	SUS	IL16	20	ES14-0057	54.4	26.7	1.1
2016	SUS	IL16	21	ES14-0528	20.2	10.0	1.7
2016	SUS	IL16	22	ES14-1293	33.2	50.0	0.8
2016	SUS	IL16	23	ES14-1350	71.8	60.0	2.0
2016	SUS	IL16	24	GA08250-15ES14	49.9	23.3	2.1
2016	SUS	IL16	25	GA08293-15ES3	62.7	66.7	6.1
2016	SUS	IL16	26	GA09361-15ES38	75.5	18.4	2.1
2016	SUS	IL16	27	GA091252-15ES35	70.6	68.6	4.2
2016	SUS	IL16	28	GA08281-15ES1	68.6	48.8	2.6
2016	SUS	IL16	29	GANC9337-15ES27	47.6	20.6	4.2
2016	SUS	IL16	30	GA09343-15ES33	73.3	30.8	5.3
2016	SUS	IL16	31	GANC10014-15ES24	44.6	36.7	2.9
2016	SUS	IL16	32	KWS053	22.3	33.6	2.2
2016	SUS	IL16	33	KWS060	57.5	13.3	2.0
2016	SUS	IL16	34	KWS074	33.7	8.3	1.0
2016	SUS	IL16	35	KWS081	60.6	30.0	0.7
2016	SUS	IL16	36	KWS083	43.6	30.0	1.1
2016	SUS	IL16	37	KWS087	55.4	30.0	2.2
2016	SUS	IL16	38	LA06146E-P4	NA	NA	NA

2016	SUS	IL16	39	LA08090C-9-2	64.4	70.0	2.6
2016	SUS	IL16	40	LA08265C-50	57.8	36.7	3.1
2016	SUS	IL16	41	LA09011UB-2	32.9	70.0	6.8
2016	SUS	IL16	42	LA09225C-33	69.3	25.8	2.2
2016	SUS	IL16	43	NC10435-11	64.2	30.0	3.1
2016	SUS	IL16	44	NC12-22225	40.6	26.7	0.9
2016	SUS	IL16	45	NC13-20076	36.0	16.7	3.0
2016	SUS	IL16	46	NC13-22350	31.0	46.7	1.0
2016	SUS	IL16	47	NC13-23449	61.0	50.0	2.1
2016	SUS	IL16	48	VA12W-68	37.2	20.8	6.6
2016	SUS	IL16	49	VA13W-38	17.2	21.7	1.0
2016	SUS	IL16	50	VA09MAS6-122-7-1	48.0	53.3	5.3
2016	SUS	IL16	51	VA08MAS1-188-6-4-1	57.8	76.7	2.0
2016	SUS	IL16	52	VA13FHB-26	27.2	26.7	2.3
2016	SUS	IL16	53	VA14FHB-14	42.3	43.3	1.6
2016	SUS	IL16	54	VA14FHB-13	24.8	36.7	3.5
2016	SUS	IL16	55	VA14FHB-28	62.1	40.0	3.8
2016	SUS	KY16	1	ERNIE	30.0	33.0	15.5
2016	SUS	KY16	2	COKER9835	62.8	43.0	23.1
2016	SUS	KY16	3	BESS	15.3	14.0	4.6
2016	SUS	KY16	4	JAMESTOWN	34.4	27.0	10.2
2016	SUS	KY16	5	AR06024-7-2	17.6	9.0	2.2
2016	SUS	KY16	6	ARS10-389	31.1	16.0	7.8
2016	SUS	KY16	7	AR07010-7-1	31.3	27.0	9.8
2016	SUS	KY16	8	AR07053-13-1	17.9	29.0	8.1
2016	SUS	KY16	9	AR07078-7-4	40.3	42.0	9.1
2016	SUS	KY16	10	AR07108-6-1	28.6	31.0	8.6
2016	SUS	KY16	11	ARLA06146E-20-1	20.4	28.0	8.9
2016	SUS	KY16	12	ARLA07084C-10-1	18.8	31.0	5.0
2016	SUS	KY16	13	ARS11-2086	32.8	31.0	12.6
2016	SUS	KY16	14	ARS12-201	35.0	22.0	9.2
2016	SUS	KY16	15	ARS13-159	24.3	15.0	8.2
2016	SUS	KY16	16	ARS13-215	42.7	27.0	8.0
2016	SUS	KY16	17	ARS14W0539	36.8	11.0	12.6
2016	SUS	KY16	18	ARS14W0623	19.0	21.0	8.9
2016	SUS	KY16	19	ARS14W1012	44.0	36.0	17.3
2016	SUS	KY16	20	ES14-0057	21.3	25.0	7.6
2016	SUS	KY16	21	ES14-0528	25.5	24.0	7.4
2016	SUS	KY16	22	ES14-1293	16.3	16.0	4.6
2016	SUS	KY16	23	ES14-1350	21.1	12.0	8.0
2016	SUS	KY16	24	GA08250-15ES14	22.3	23.0	7.2
2016	SUS	KY16	25	GA08293-15ES3	40.7	47.0	21.6
2016	SUS	KY16	26	GA09361-15ES38	45.9	38.0	11.9
2016	SUS	KY16	27	GA091252-15ES35	32.6	31.0	15.5
2016	SUS	KY16	28	GA08281-15ES1	30.3	28.0	8.3
2016	SUS	KY16	29	GANC9337-15ES27	30.1	17.0	7.6
2016	SUS	KY16	30	GA09343-15ES33	45.6	24.0	11.2
2016	SUS	KY16	31	GANC10014-15ES24	40.1	32.0	13.2
2016	SUS	KY16	32	KWS053	26.9	23.0	10.0

2016	SUS	KY16	33	KWS060	27.2	23.0	10.6
2016	SUS	KY16	34	KWS074	19.9	47.0	9.2
2016	SUS	KY16	35	KWS081	18.4	16.0	6.8
2016	SUS	KY16	36	KWS083	23.4	31.0	9.1
2016	SUS	KY16	37	KWS087	24.7	18.0	6.8
2016	SUS	KY16	38	LA06146E-P4	35.5	27.0	10.7
2016	SUS	KY16	39	LA08090C-9-2	31.9	19.0	5.7
2016	SUS	KY16	40	LA08265C-50	29.8	22.0	7.6
2016	SUS	KY16	41	LA09011UB-2	31.7	31.0	13.2
2016	SUS	KY16	42	LA09225C-33	32.8	36.0	12.0
2016	SUS	KY16	43	NC10435-11	29.1	23.0	9.4
2016	SUS	KY16	44	NC12-22225	25.4	18.0	8.8
2016	SUS	KY16	45	NC13-20076	24.8	17.0	7.5
2016	SUS	KY16	46	NC13-22350	21.8	12.0	10.9
2016	SUS	KY16	47	NC13-23449	29.9	25.0	9.2
2016	SUS	KY16	48	VA12W-68	33.7	40.0	13.3
2016	SUS	KY16	49	VA13W-38	35.9	27.0	5.9
2016	SUS	KY16	50	VA09MAS6-122-7-1	28.9	31.0	12.7
2016	SUS	KY16	51	VA08MAS1-188-6-4-1	25.6	22.0	10.4
2016	SUS	KY16	52	VA13FHB-26	21.6	25.0	10.2
2016	SUS	KY16	53	VA14FHB-14	23.2	21.0	7.9
2016	SUS	KY16	54	VA14FHB-13	27.1	41.0	10.3
2016	SUS	KY16	55	VA14FHB-28	38.3	35.0	11.4
2016	SUS	MO16	1	ERNIE	15.0	32.5	9.0
2016	SUS	MO16	2	COKER9835	34.0	80.0	16.0
2016	SUS	MO16	3	BESS	15.0	25.0	9.0
2016	SUS	MO16	4	JAMESTOWN	19.0	27.5	13.0
2016	SUS	MO16	5	AR06024-7-2	13.0	17.5	10.0
2016	SUS	MO16	6	ARS10-389	19.0	35.0	16.0
2016	SUS	MO16	7	AR07010-7-1	17.0	22.5	23.0
2016	SUS	MO16	8	AR07053-13-1	24.0	12.5	17.0
2016	SUS	MO16	9	AR07078-7-4	23.0	27.5	33.0
2016	SUS	MO16	10	AR07108-6-1	17.0	25.0	16.0
2016	SUS	MO16	11	ARLA06146E-20-1	17.0	35.0	19.0
2016	SUS	MO16	12	ARLA07084C-10-1	12.0	37.5	32.0
2016	SUS	MO16	13	ARS11-2086	10.0	80.0	18.0
2016	SUS	MO16	14	ARS12-201	15.0	82.5	17.0
2016	SUS	MO16	15	ARS13-159	23.0	80.0	9.3
2016	SUS	MO16	16	ARS13-215	13.0	25.0	27.8
2016	SUS	MO16	17	ARS14W0539	15.0	30.0	32.7
2016	SUS	MO16	18	ARS14W0623	19.0	52.5	16.4
2016	SUS	MO16	19	ARS14W1012	24.0	35.0	28.1
2016	SUS	MO16	20	ES14-0057	8.0	22.5	2.7
2016	SUS	MO16	21	ES14-0528	15.0	30.0	13.6
2016	SUS	MO16	22	ES14-1293	10.0	10.0	6.5
2016	SUS	MO16	23	ES14-1350	25.0	87.5	14.9
2016	SUS	MO16	24	GA08250-15ES14	13.0	12.5	14.6
2016	SUS	MO16	25	GA08293-15ES3	20.0	30.0	21.3
2016	SUS	MO16	26	GA09361-15ES38	29.0	32.5	17.2

2016	SUS	MO16	27	GA091252-15ES35	11.0	22.5	18.8
2016	SUS	MO16	28	GA08281-15ES1	22.0	35.0	16.3
2016	SUS	MO16	29	GANC9337-15ES27	21.0	17.5	19.8
2016	SUS	MO16	30	GA09343-15ES33	12.0	30.0	18.6
2016	SUS	MO16	31	GANC10014-15ES24	24.0	40.0	15.6
2016	SUS	MO16	32	KWS053	9.0	12.5	8.7
2016	SUS	MO16	33	KWS060	12.0	28.0	9.0
2016	SUS	MO16	34	KWS074	21.0	35.0	19.0
2016	SUS	MO16	35	KWS081	10.0	38.0	15.0
2016	SUS	MO16	36	KWS083	9.0	18.0	10.0
2016	SUS	MO16	37	KWS087	25.0	50.0	14.0
2016	SUS	MO16	38	LA06146E-P4	28.0	45.0	17.0
2016	SUS	MO16	39	LA08090C-9-2	33.0	40.0	28.0
2016	SUS	MO16	40	LA08265C-50	40.0	40.0	15.0
2016	SUS	MO16	41	LA09011UB-2	54.0	30.0	36.0
2016	SUS	MO16	42	LA09225C-33	43.0	40.0	34.0
2016	SUS	MO16	43	NC10435-11	41.0	35.0	14.0
2016	SUS	MO16	44	NC12-22225	22.0	30.0	8.0
2016	SUS	MO16	45	NC13-20076	29.0	45.0	16.0
2016	SUS	MO16	46	NC13-22350	14.0	18.0	8.0
2016	SUS	MO16	47	NC13-23449	13.0	45.0	13.0
2016	SUS	MO16	48	VA12W-68	11.0	13.0	21.0
2016	SUS	MO16	49	VA13W-38	21.0	40.0	30.0
2016	SUS	MO16	50	VA09MAS6-122-7-1	19.0	23.0	27.0
2016	SUS	MO16	51	VA08MAS1-188-6-4-1	17.0	23.0	12.0
2016	SUS	MO16	52	VA13FHB-26	28.0	93.0	18.0
2016	SUS	MO16	53	VA14FHB-14	24.0	95.0	31.0
2016	SUS	MO16	54	VA14FHB-13	18.0	88.0	19.0
2016	SUS	MO16	55	VA14FHB-28	32.0	25.0	25.0
2015	SUS	NC15	1	ERNIE	7.0	4.2	4.4
2015	SUS	NC15	2	COKER9835	53.5	24.9	16.7
2015	SUS	NC15	3	BESS	17.5	3.2	5.5
2015	SUS	NC15	4	JAMESTOWN	7.0	4.1	2.1
2015	SUS	NC15	5	LA07085CW-P4	14.0	5.7	3.0
2015	SUS	NC15	6	LANC8170-41-2	17.5	4.0	2.2
2015	SUS	NC15	7	NC11-22289	7.0	2.9	1.2
2015	SUS	NC15	8	AR06024-7-2	7.0	3.2	2.2
2015	SUS	NC15	9	AR06037-17-2	33.0	11.6	7.0
2015	SUS	NC15	10	AR06045-2-4	14.0	2.6	3.2
2015	SUS	NC15	11	AR06045-16-4	21.0	7.1	3.9
2015	SUS	NC15	12	AR06046-10-3	30.5	6.4	9.3
2015	SUS	NC15	13	AR06061-11-1	14.0	4.0	2.4
2015	SUS	NC15	14	LW08190C-57-3	10.5	4.9	2.5
2015	SUS	NC15	15	ARGE08-1398	10.5	1.4	1.0
2015	SUS	NC15	16	B12-1792	14.0	4.3	5.0
2015	SUS	NC15	17	B12-2180NC	27.0	5.7	6.3
2015	SUS	NC15	18	GA071171-14ES8	29.0	5.0	5.3
2015	SUS	NC15	19	GA071092-14ES11	21.0	5.7	5.0
2015	SUS	NC15	20	GA071092-14ES13	28.5	6.4	6.5

2015	SUS	NC15	21	GA081129-14ES16	14.0	3.6	2.5
2015	SUS	NC15	22	GA08250-14ES7	14.0	3.9	3.6
2015	SUS	NC15	23	GA08250-14ES5	31.0	8.3	5.2
2015	SUS	NC15	24	GA071171-14ES19	14.0	7.9	8.9
2015	SUS	NC15	25	GA081562-14ES14	17.5	7.3	8.9
2015	SUS	NC15	26	KWS054	7.0	4.4	5.4
2015	SUS	NC15	27	LA06146E-P4	7.0	6.3	7.1
2015	SUS	NC15	28	LA08265C-50	17.5	6.5	8.0
2015	SUS	NC15	29	LA09144C-6	17.5	8.2	18.8
2015	SUS	NC15	30	LANC8248-1	17.5	8.4	10.9
2015	SUS	NC15	31	ES13-1591	14.0	2.5	4.5
2015	SUS	NC15	32	ES13-3423	10.5	3.8	5.2
2015	SUS	NC15	33	ES12-3030	14.0	2.5	3.7
2015	SUS	NC15	34	M11-2024	14.0	6.5	8.5
2015	SUS	NC15	35	M12-3301	17.5	9.0	4.1
2015	SUS	NC15	36	M12-2036	27.5	4.3	6.9
2015	SUS	NC15	37	NC11-23084	27.0	8.3	10.0
2015	SUS	NC15	38	NC12-23576	33.0	10.3	11.9
2015	SUS	NC15	39	NC12-23219	14.0	4.7	2.2
2015	SUS	NC15	40	NC12-20662	10.5	4.4	2.3
2015	SUS	NC15	41	NC9305-7	27.0	4.4	5.6
2015	SUS	NC15	42	VA11W-106	21.0	11.1	17.0
2015	SUS	NC15	43	VA11W-313	7.0	4.7	5.0
2015	SUS	NC15	44	VA12W-72	16.0	3.6	4.1
2015	SUS	NC15	45	VA12W-54	27.0	5.0	4.8
2015	SUS	NC15	46	VA12FHB-53	33.0	4.5	4.7
2015	SUS	NC15	47	VA12FHB-4	27.0	3.2	4.0
2015	SUS	NC15	48	VA13W-177	7.0	1.2	1.2
2015	SUS	NC15	49	VA08MAS5-39-6-4	17.5	4.9	6.7
2015	SUS	KY15	1	ERNIE	32.3	18.8	11.7
2015	SUS	KY15	2	COKER9835	52.3	30.7	10.8
2015	SUS	KY15	3	BESS	42.3	16.7	5.5
2015	SUS	KY15	4	JAMESTOWN	45.5	26.2	5.8
2015	SUS	KY15	5	LA07085CW-P4	28.6	13.9	8.6
2015	SUS	KY15	6	LANC8170-41-2	45.6	17.2	5.8
2015	SUS	KY15	7	NC11-22289	16.4	4.6	2.6
2015	SUS	KY15	8	AR06024-7-2	29.9	5.2	2.9
2015	SUS	KY15	9	AR06037-17-2	46.4	20.4	9.5
2015	SUS	KY15	10	AR06045-2-4	25.1	12.2	6.7
2015	SUS	KY15	11	AR06045-16-4	30.1	12.0	7.1
2015	SUS	KY15	12	AR06046-10-3	32.7	15.8	5.4
2015	SUS	KY15	13	AR06061-11-1	44.1	14.7	4.0
2015	SUS	KY15	14	LW08190C-57-3	33.7	18.5	3.7
2015	SUS	KY15	15	ARGE08-1398	17.3	5.7	2.7
2015	SUS	KY15	16	B12-1792	53.8	16.0	15.9
2015	SUS	KY15	17	B12-2180NC	51.6	21.4	9.8
2015	SUS	KY15	18	GA071171-14ES8	46.8	13.7	7.5
2015	SUS	KY15	19	GA071092-14ES11	31.3	17.6	8.4
2015	SUS	KY15	20	GA071092-14ES13	33.8	16.1	8.8

2015	SUS	KY15	21	GA081129-14ES16	16.7	12.5	9.3
2015	SUS	KY15	22	GA08250-14ES7	35.3	14.2	8.7
2015	SUS	KY15	23	GA08250-14ES5	43.0	20.0	13.3
2015	SUS	KY15	24	GA071171-14ES19	27.6	12.2	9.6
2015	SUS	KY15	25	GA081562-14ES14	44.8	25.9	16.5
2015	SUS	KY15	26	KWS054	22.1	17.0	8.7
2015	SUS	KY15	27	LA06146E-P4	43.4	20.3	14.6
2015	SUS	KY15	28	LA08265C-50	42.6	17.2	7.5
2015	SUS	KY15	29	LA09144C-6	40.5	33.6	20.1
2015	SUS	KY15	30	LANC8248-1	68.6	28.4	11.3
2015	SUS	KY15	31	ES13-1591	28.3	15.1	3.2
2015	SUS	KY15	32	ES13-3423	38.1	12.6	4.5
2015	SUS	KY15	33	ES12-3030	35.8	12.5	6.0
2015	SUS	KY15	34	M11-2024	53.4	13.7	6.7
2015	SUS	KY15	35	M12-3301	36.0	15.9	5.8
2015	SUS	KY15	36	M12-2036	36.5	13.7	7.6
2015	SUS	KY15	37	NC11-23084	43.9	19.2	7.2
2015	SUS	KY15	38	NC12-23576	39.3	23.1	13.7
2015	SUS	KY15	39	NC12-23219	29.8	14.8	4.9
2015	SUS	KY15	40	NC12-20662	18.6	7.8	6.3
2015	SUS	KY15	41	NC9305-7	27.5	13.6	4.2
2015	SUS	KY15	42	VA11W-106	44.9	18.6	9.1
2015	SUS	KY15	43	VA11W-313	33.7	17.6	7.6
2015	SUS	KY15	44	VA12W-72	39.5	15.0	9.0
2015	SUS	KY15	45	VA12W-54	57.4	20.9	16.1
2015	SUS	KY15	46	VA12FHB-53	49.2	18.1	4.2
2015	SUS	KY15	47	VA12FHB-4	30.6	11.2	5.5
2015	SUS	KY15	48	VA13W-177	16.9	6.5	4.1
2015	SUS	KY15	49	VA08MAS5-39-6-4	33.2	18.2	7.4
2015	SUS	MO15	1	ERNIE	17.0	35.0	6.2
2015	SUS	MO15	2	COKER9835	51.5	80.0	7.1
2015	SUS	MO15	3	BESS	3.1	17.5	4.3
2015	SUS	MO15	4	JAMESTOWN	18.1	40.0	5.6
2015	SUS	MO15	5	LA07085CW-P4	7.7	62.5	6.6
2015	SUS	MO15	6	LANC8170-41-2	30.7	87.5	4.3
2015	SUS	MO15	7	NC11-22289	13.3	20.0	2.3
2015	SUS	MO15	8	AR06024-7-2	14.4	17.5	2.9
2015	SUS	MO15	9	AR06037-17-2	35.9	85.0	10.9
2015	SUS	MO15	10	AR06045-2-4	4.9	37.5	3.3
2015	SUS	MO15	11	AR06045-16-4	11.0	35.0	4.3
2015	SUS	MO15	12	AR06046-10-3	29.5	72.5	9.3
2015	SUS	MO15	13	AR06061-11-1	23.7	42.5	8.9
2015	SUS	MO15	14	LW08190C-57-3	13.5	62.5	5.0
2015	SUS	MO15	15	ARGE08-1398	5.9	45.0	0.8
2015	SUS	MO15	16	B12-1792	33.1	55.0	7.8
2015	SUS	MO15	17	B12-2180NC	10.1	37.5	6.3
2015	SUS	MO15	18	GA071171-14ES8	33.3	50.0	5.3
2015	SUS	MO15	19	GA071092-14ES11	14.6	67.5	4.9
2015	SUS	MO15	20	GA071092-14ES13	26.9	55.0	4.1

2015	SUS	MO15	21	GA081129-14ES16	22.7	65.0	5.5
2015	SUS	MO15	22	GA08250-14ES7	33.2	62.5	8.3
2015	SUS	MO15	23	GA08250-14ES5	8.9	40.0	5.0
2015	SUS	MO15	24	GA071171-14ES19	26.5	55.0	8.4
2015	SUS	MO15	25	GA081562-14ES14	38.2	60.0	10.6
2015	SUS	MO15	26	KWS054	25.5	57.5	8.6
2015	SUS	MO15	27	LA06146E-P4	9.3	80.0	7.2
2015	SUS	MO15	28	LA08265C-50	13.2	60.0	5.3
2015	SUS	MO15	29	LA09144C-6	63.4	90.0	7.6
2015	SUS	MO15	30	LANC8248-1	13.1	50.0	6.7
2015	SUS	MO15	31	ES13-1591	16.5	57.5	5.4
2015	SUS	MO15	32	ES13-3423	14.3	77.5	9.8
2015	SUS	MO15	33	ES12-3030	14.3	57.5	7.6
2015	SUS	MO15	34	M11-2024	23.3	25.0	7.4
2015	SUS	MO15	35	M12-3301	23.3	50.0	3.6
2015	SUS	MO15	36	M12-2036	7.9	87.5	17.2
2015	SUS	MO15	37	NC11-23084	9.8	32.5	2.9
2015	SUS	MO15	38	NC12-23576	20.4	85.0	7.2
2015	SUS	MO15	39	NC12-23219	24.2	60.0	11.6
2015	SUS	MO15	40	NC12-20662	8.0	32.5	4.8
2015	SUS	MO15	41	NC9305-7	27.4	17.5	6.5
2015	SUS	MO15	42	VA11W-106	10.1	72.5	6.6
2015	SUS	MO15	43	VA11W-313	24.3	90.0	12.1
2015	SUS	MO15	44	VA12W-72	16.4	75.0	13.2
2015	SUS	MO15	45	VA12W-54	26.1	87.5	16.0
2015	SUS	MO15	46	VA12FHB-53	18.5	67.5	3.7
2015	SUS	MO15	47	VA12FHB-4	13.4	37.5	5.3
2015	SUS	MO15	48	VA13W-177	11.3	35.0	6.0
2015	SUS	MO15	49	VA08MAS5-39-6-4	21.7	60.0	6.9
2015	SUS	VA15	1	ERNIE	7.0	NA	0.1
2015	SUS	VA15	2	COKER9835	30.5	NA	1.0
2015	SUS	VA15	3	BESS	13.0	NA	0.2
2015	SUS	VA15	4	JAMESTOWN	7.0	NA	0.1
2015	SUS	VA15	5	LA07085CW-P4	24.3	NA	0.5
2015	SUS	VA15	6	LANC8170-41-2	19.8	NA	0.4
2015	SUS	VA15	7	NC11-22289	9.3	NA	0.2
2015	SUS	VA15	8	AR06024-7-2	13.6	NA	0.1
2015	SUS	VA15	9	AR06037-17-2	19.1	NA	0.8
2015	SUS	VA15	10	AR06045-2-4	29.6	NA	0.1
2015	SUS	VA15	11	AR06045-16-4	21.6	NA	0.1
2015	SUS	VA15	12	AR06046-10-3	15.2	NA	0.1
2015	SUS	VA15	13	AR06061-11-1	8.6	NA	0.2
2015	SUS	VA15	14	LW08190C-57-3	15.1	NA	0.1
2015	SUS	VA15	15	ARGE08-1398	9.8	NA	NA
2015	SUS	VA15	16	B12-1792	12.6	NA	0.6
2015	SUS	VA15	17	B12-2180NC	19.9	NA	0.9
2015	SUS	VA15	18	GA071171-14ES8	18.1	NA	0.7
2015	SUS	VA15	19	GA071092-14ES11	17.7	NA	0.3
2015	SUS	VA15	20	GA071092-14ES13	11.3	NA	0.3

2015	SUS	VA15	21	GA081129-14ES16	NA	NA	NA
2015	SUS	VA15	22	GA08250-14ES7	12.1	NA	0.4
2015	SUS	VA15	23	GA08250-14ES5	11.8	NA	0.4
2015	SUS	VA15	24	GA071171-14ES19	23.6	NA	0.4
2015	SUS	VA15	25	GA081562-14ES14	21.1	NA	0.8
2015	SUS	VA15	26	KWS054	10.3	NA	0.6
2015	SUS	VA15	27	LA06146E-P4	22.7	NA	1.0
2015	SUS	VA15	28	LA08265C-50	21.7	NA	1.0
2015	SUS	VA15	29	LA09144C-6	31.5	NA	1.3
2015	SUS	VA15	30	LANC8248-1	25.1	NA	0.4
2015	SUS	VA15	31	ES13-1591	10.5	NA	0.1
2015	SUS	VA15	32	ES13-3423	11.1	NA	0.2
2015	SUS	VA15	33	ES12-3030	13.3	NA	0.1
2015	SUS	VA15	34	M11-2024	14.9	NA	0.5
2015	SUS	VA15	35	M12-3301	13.0	NA	0.2
2015	SUS	VA15	36	M12-2036	15.8	NA	0.4
2015	SUS	VA15	37	NC11-23084	37.7	NA	0.1
2015	SUS	VA15	38	NC12-23576	35.0	NA	0.6
2015	SUS	VA15	39	NC12-23219	8.2	NA	0.1
2015	SUS	VA15	40	NC12-20662	26.6	NA	0.1
2015	SUS	VA15	41	NC9305-7	16.0	NA	0.2
2015	SUS	VA15	42	VA11W-106	14.3	NA	0.5
2015	SUS	VA15	43	VA11W-313	16.4	NA	0.6
2015	SUS	VA15	44	VA12W-72	16.9	NA	0.6
2015	SUS	VA15	45	VA12W-54	25.3	NA	0.4
2015	SUS	VA15	46	VA12FHB-53	10.9	NA	0.2
2015	SUS	VA15	47	VA12FHB-4	31.3	NA	0.1
2015	SUS	VA15	48	VA13W-177	9.3	NA	0.1
2015	SUS	VA15	49	VA08MAS5-39-6-4	37.3	NA	0.2
2015	SUS	IL15	1	ERNIE	69.3	30.0	NA
2015	SUS	IL15	2	COKER9835	74.8	55.0	NA
2015	SUS	IL15	3	BESS	47.5	15.0	NA
2015	SUS	IL15	4	JAMESTOWN	46.2	23.3	NA
2015	SUS	IL15	5	LA07085CW-P4	55.0	50.0	NA
2015	SUS	IL15	6	LANC8170-41-2	65.9	40.0	NA
2015	SUS	IL15	7	NC11-22289	21.6	25.0	NA
2015	SUS	IL15	8	AR06024-7-2	22.2	28.3	NA
2015	SUS	IL15	9	AR06037-17-2	43.2	31.7	NA
2015	SUS	IL15	10	AR06045-2-4	25.0	13.3	NA
2015	SUS	IL15	11	AR06045-16-4	32.6	26.7	NA
2015	SUS	IL15	12	AR06046-10-3	66.6	23.3	NA
2015	SUS	IL15	13	AR06061-11-1	34.0	10.0	NA
2015	SUS	IL15	14	LW08190C-57-3	27.1	51.7	NA
2015	SUS	IL15	15	ARGE08-1398	13.9	24.6	NA
2015	SUS	IL15	16	B12-1792	76.8	20.0	NA
2015	SUS	IL15	17	B12-2180NC	53.3	26.7	NA
2015	SUS	IL15	18	GA071171-14ES8	66.7	33.3	NA
2015	SUS	IL15	19	GA071092-14ES11	30.4	38.3	NA
2015	SUS	IL15	20	GA071092-14ES13	26.1	41.7	NA

2015	SUS	IL15	21	GA081129-14ES16	60.1	51.7	NA
2015	SUS	IL15	22	GA08250-14ES7	42.3	23.3	NA
2015	SUS	IL15	23	GA08250-14ES5	43.9	50.0	NA
2015	SUS	IL15	24	GA071171-14ES19	44.6	28.3	NA
2015	SUS	IL15	25	GA081562-14ES14	14.0	73.3	NA
2015	SUS	IL15	26	KWS054	47.3	53.3	NA
2015	SUS	IL15	27	LA06146E-P4	38.6	53.3	NA
2015	SUS	IL15	28	LA08265C-50	89.0	46.7	NA
2015	SUS	IL15	29	LA09144C-6	67.7	33.3	NA
2015	SUS	IL15	30	LANC8248-1	75.6	19.6	NA
2015	SUS	IL15	31	ES13-1591	30.7	21.7	NA
2015	SUS	IL15	32	ES13-3423	33.4	41.7	NA
2015	SUS	IL15	33	ES12-3030	77.5	26.7	NA
2015	SUS	IL15	34	M11-2024	72.6	20.0	NA
2015	SUS	IL15	35	M12-3301	36.9	15.0	NA
2015	SUS	IL15	36	M12-2036	37.5	15.0	NA
2015	SUS	IL15	37	NC11-23084	46.3	33.3	NA
2015	SUS	IL15	38	NC12-23576	64.0	38.3	NA
2015	SUS	IL15	39	NC12-23219	62.8	28.3	NA
2015	SUS	IL15	40	NC12-20662	17.7	33.3	NA
2015	SUS	IL15	41	NC9305-7	58.7	17.1	NA
2015	SUS	IL15	42	VA11W-106	64.5	40.0	NA
2015	SUS	IL15	43	VA11W-313	78.9	56.7	NA
2015	SUS	IL15	44	VA12W-72	46.4	70.0	NA
2015	SUS	IL15	45	VA12W-54	53.7	43.3	NA
2015	SUS	IL15	46	VA12FHB-53	32.6	47.1	NA
2015	SUS	IL15	47	VA12FHB-4	20.2	13.3	NA
2015	SUS	IL15	48	VA13W-177	33.4	20.0	NA
2015	SUS	IL15	49	VA08MAS5-39-6-4	44.1	33.3	NA
2015	SUS	HIL15	1	ERNIE	30.0	25.0	NA
2015	SUS	HIL15	2	COKER9835	65.0	60.0	NA
2015	SUS	HIL15	3	BESS	25.0	60.0	NA
2015	SUS	HIL15	4	JAMESTOWN	40.0	10.0	NA
2015	SUS	HIL15	5	LA07085CW-P4	55.0	60.0	NA
2015	SUS	HIL15	6	LANC8170-41-2	45.0	20.0	NA
2015	SUS	HIL15	7	NC11-22289	35.0	50.0	NA
2015	SUS	HIL15	8	AR06024-7-2	45.0	12.5	NA
2015	SUS	HIL15	9	AR06037-17-2	25.0	20.0	NA
2015	SUS	HIL15	10	AR06045-2-4	10.0	22.5	NA
2015	SUS	HIL15	11	AR06045-16-4	20.0	6.5	NA
2015	SUS	HIL15	12	AR06046-10-3	15.0	20.0	NA
2015	SUS	HIL15	13	AR06061-11-1	10.0	22.5	NA
2015	SUS	HIL15	14	LW08190C-57-3	40.0	10.0	NA
2015	SUS	HIL15	15	ARGE08-1398	15.0	4.0	NA
2015	SUS	HIL15	16	B12-1792	70.0	22.5	NA
2015	SUS	HIL15	17	B12-2180NC	35.0	12.5	NA
2015	SUS	HIL15	18	GA071171-14ES8	65.0	22.5	NA
2015	SUS	HIL15	19	GA071092-14ES11	35.0	30.0	NA
2015	SUS	HIL15	20	GA071092-14ES13	40.0	30.0	NA

2015	SUS	HIL15	21	GA081129-14ES16	55.0	55.0	NA
2015	SUS	HIL15	22	GA08250-14ES7	20.0	47.5	NA
2015	SUS	HIL15	23	GA08250-14ES5	40.0	15.0	NA
2015	SUS	HIL15	24	GA071171-14ES19	35.0	50.0	NA
2015	SUS	HIL15	25	GA081562-14ES14	55.0	42.5	NA
2015	SUS	HIL15	26	KWS054	55.0	65.0	NA
2015	SUS	HIL15	27	LA06146E-P4	55.0	75.0	NA
2015	SUS	HIL15	28	LA08265C-50	55.0	45.0	NA
2015	SUS	HIL15	29	LA09144C-6	45.0	57.5	NA
2015	SUS	HIL15	30	LANC8248-1	65.0	22.5	NA
2015	SUS	HIL15	31	ES13-1591	40.0	47.5	NA
2015	SUS	HIL15	32	ES13-3423	35.0	40.0	NA
2015	SUS	HIL15	33	ES12-3030	40.0	45.0	NA
2015	SUS	HIL15	34	M11-2024	45.0	22.5	NA
2015	SUS	HIL15	35	M12-3301	20.0	22.5	NA
2015	SUS	HIL15	36	M12-2036	50.0	70.0	NA
2015	SUS	HIL15	37	NC11-23084	15.0	7.5	NA
2015	SUS	HIL15	38	NC12-23576	30.0	22.5	NA
2015	SUS	HIL15	39	NC12-23219	55.0	15.0	NA
2015	SUS	HIL15	40	NC12-20662	40.0	30.0	NA
2015	SUS	HIL15	41	NC9305-7	20.0	11.5	NA
2015	SUS	HIL15	42	VA11W-106	25.0	35.0	NA
2015	SUS	HIL15	43	VA11W-313	75.0	31.5	NA
2015	SUS	HIL15	44	VA12W-72	60.0	75.0	NA
2015	SUS	HIL15	45	VA12W-54	70.0	57.5	NA
2015	SUS	HIL15	46	VA12FHB-53	35.0	22.5	NA
2015	SUS	HIL15	47	VA12FHB-4	35.0	3.0	NA
2015	SUS	HIL15	48	VA13W-177	30.0	35.0	NA
2015	SUS	HIL15	49	VA08MAS5-39-6-4	75.0	57.5	NA
2015	SUS	GA15	1	ERNIE	25.0	NA	2.8
2015	SUS	GA15	2	COKER9835	80.0	NA	7.0
2015	SUS	GA15	3	BESS	26.0	NA	1.7
2015	SUS	GA15	4	JAMESTOWN	22.0	NA	0.6
2015	SUS	GA15	5	LA07085CW-P4	33.0	NA	6.5
2015	SUS	GA15	6	LANC8170-41-2	28.0	NA	1.0
2015	SUS	GA15	7	NC11-22289	18.0	NA	0.6
2015	SUS	GA15	8	AR06024-7-2	13.0	NA	0.5
2015	SUS	GA15	9	AR06037-17-2	40.0	NA	3.8
2015	SUS	GA15	10	AR06045-2-4	22.0	NA	2.1
2015	SUS	GA15	11	AR06045-16-4	30.0	NA	3.0
2015	SUS	GA15	12	AR06046-10-3	35.0	NA	5.2
2015	SUS	GA15	13	AR06061-11-1	25.0	NA	4.6
2015	SUS	GA15	14	LW08190C-57-3	30.0	NA	4.5
2015	SUS	GA15	15	ARGE08-1398	22.0	NA	0.1
2015	SUS	GA15	16	B12-1792	35.0	NA	2.7
2015	SUS	GA15	17	B12-2180NC	33.0	NA	3.5
2015	SUS	GA15	18	GA071171-14ES8	35.0	NA	3.6
2015	SUS	GA15	19	GA071092-14ES11	40.0	NA	7.7
2015	SUS	GA15	20	GA071092-14ES13	45.0	NA	4.1

2015	SUS	GA15	21	GA081129-14ES16	35.0	NA	2.3
2015	SUS	GA15	22	GA08250-14ES7	33.0	NA	5.7
2015	SUS	GA15	23	GA08250-14ES5	40.0	NA	4.2
2015	SUS	GA15	24	GA071171-14ES19	40.0	NA	6.7
2015	SUS	GA15	25	GA081562-14ES14	28.0	NA	9.4
2015	SUS	GA15	26	KWS054	30.0	NA	3.7
2015	SUS	GA15	27	LA06146E-P4	28.0	NA	2.2
2015	SUS	GA15	28	LA08265C-50	35.0	NA	2.2
2015	SUS	GA15	29	LA09144C-6	40.0	NA	5.0
2015	SUS	GA15	30	LANC8248-1	33.0	NA	5.8
2015	SUS	GA15	31	ES13-1591	35.0	NA	2.7
2015	SUS	GA15	32	ES13-3423	50.0	NA	3.4
2015	SUS	GA15	33	ES12-3030	40.0	NA	3.1
2015	SUS	GA15	34	M11-2024	45.0	NA	2.5
2015	SUS	GA15	35	M12-3301	40.0	NA	3.3
2015	SUS	GA15	36	M12-2036	35.0	NA	2.9
2015	SUS	GA15	37	NC11-23084	55.0	NA	9.8
2015	SUS	GA15	38	NC12-23576	45.0	NA	3.0
2015	SUS	GA15	39	NC12-23219	25.0	NA	0.9
2015	SUS	GA15	40	NC12-20662	40.0	NA	2.4
2015	SUS	GA15	41	NC9305-7	30.0	NA	3.6
2015	SUS	GA15	42	VA11W-106	40.0	NA	10.2
2015	SUS	GA15	43	VA11W-313	25.0	NA	2.5
2015	SUS	GA15	44	VA12W-72	22.0	NA	8.3
2015	SUS	GA15	45	VA12W-54	33.0	NA	5.4
2015	SUS	GA15	46	VA12FHB-53	35.0	NA	1.8
2015	SUS	GA15	47	VA12FHB-4	45.0	NA	3.4
2015	SUS	GA15	48	VA13W-177	22.0	NA	1.0
2015	SUS	GA15	49	VA08MAS5-39-6-4	40.0	NA	3.4
2015	SUS	FAR15	1	ERNIE	95.0	90.0	17.0
2015	SUS	FAR15	2	COKER9835	100.0	95.0	13.6
2015	SUS	FAR15	3	BESS	35.0	46.7	9.7
2015	SUS	FAR15	4	JAMESTOWN	33.3	45.0	11.1
2015	SUS	FAR15	5	LA07085CW-P4	41.7	71.7	17.1
2015	SUS	FAR15	6	LANC8170-41-2	38.3	53.3	7.3
2015	SUS	FAR15	7	NC11-22289	33.3	56.7	11.5
2015	SUS	FAR15	8	AR06024-7-2	25.0	45.0	8.3
2015	SUS	FAR15	9	AR06037-17-2	50.0	73.3	12.9
2015	SUS	FAR15	10	AR06045-2-4	41.7	75.0	14.0
2015	SUS	FAR15	11	AR06045-16-4	41.7	61.7	12.6
2015	SUS	FAR15	12	AR06046-10-3	43.3	75.0	14.4
2015	SUS	FAR15	13	AR06061-11-1	38.3	48.3	10.6
2015	SUS	FAR15	14	LW08190C-57-3	35.0	76.7	7.7
2015	SUS	FAR15	15	ARGE08-1398	13.3	26.7	2.1
2015	SUS	FAR15	16	B12-1792	53.3	81.7	22.1
2015	SUS	FAR15	17	B12-2180NC	45.0	48.3	8.8
2015	SUS	FAR15	18	GA071171-14ES8	41.7	55.0	12.2
2015	SUS	FAR15	19	GA071092-14ES11	51.7	63.3	12.5
2015	SUS	FAR15	20	GA071092-14ES13	53.3	66.7	12.3

2015	SUS	FAR15	21	GA081129-14ES16	36.7	48.3	13.6
2015	SUS	FAR15	22	GA08250-14ES7	35.0	51.7	15.5
2015	SUS	FAR15	23	GA08250-14ES5	46.7	68.3	11.2
2015	SUS	FAR15	24	GA071171-14ES19	40.0	48.3	11.9
2015	SUS	FAR15	25	GA081562-14ES14	38.3	65.0	18.5
2015	SUS	FAR15	26	KWS054	40.0	76.7	14.2
2015	SUS	FAR15	27	LA06146E-P4	31.7	56.7	15.9
2015	SUS	FAR15	28	LA08265C-50	28.3	48.3	7.1
2015	SUS	FAR15	29	LA09144C-6	78.3	75.0	21.7
2015	SUS	FAR15	30	LANC8248-1	45.0	46.7	12.2
2015	SUS	FAR15	31	ES13-1591	43.3	81.7	10.1
2015	SUS	FAR15	32	ES13-3423	100.0	95.0	13.4
2015	SUS	FAR15	33	ES12-3030	55.0	68.3	12.6
2015	SUS	FAR15	34	M11-2024	26.7	53.3	14.3
2015	SUS	FAR15	35	M12-3301	90.0	88.3	10.7
2015	SUS	FAR15	36	M12-2036	25.0	56.7	17.6
2015	SUS	FAR15	37	NC11-23084	91.7	93.3	14.1
2015	SUS	FAR15	38	NC12-23576	50.0	83.3	22.6
2015	SUS	FAR15	39	NC12-23219	26.7	58.3	13.8
2015	SUS	FAR15	40	NC12-20662	96.7	81.7	12.7
2015	SUS	FAR15	41	NC9305-7	35.0	53.3	11.7
2015	SUS	FAR15	42	VA11W-106	55.0	83.3	17.1
2015	SUS	FAR15	43	VA11W-313	56.7	91.7	15.9
2015	SUS	FAR15	44	VA12W-72	40.0	91.7	26.3
2015	SUS	FAR15	45	VA12W-54	55.0	80.0	12.7
2015	SUS	FAR15	46	VA12FHB-53	43.3	90.0	16.4
2015	SUS	FAR15	47	VA12FHB-4	90.0	88.3	12.3
2015	SUS	FAR15	48	VA13W-177	18.3	38.3	9.6
2015	SUS	FAR15	49	VA08MAS5-39-6-4	88.3	88.3	9.1
2015	SUS	NAR15	1	ERNIE	63.3	38.3	13.0
2015	SUS	NAR15	2	COKER9835	90.0	75.0	21.9
2015	SUS	NAR15	3	BESS	23.3	20.0	9.4
2015	SUS	NAR15	4	JAMESTOWN	60.0	31.7	12.2
2015	SUS	NAR15	5	LA07085CW-P4	75.0	55.0	16.1
2015	SUS	NAR15	6	LANC8170-41-2	66.7	46.7	10.9
2015	SUS	NAR15	7	NC11-22289	31.7	18.3	6.7
2015	SUS	NAR15	8	AR06024-7-2	21.7	15.0	6.8
2015	SUS	NAR15	9	AR06037-17-2	28.3	30.0	19.4
2015	SUS	NAR15	10	AR06045-2-4	21.7	25.0	16.2
2015	SUS	NAR15	11	AR06045-16-4	23.3	28.3	17.3
2015	SUS	NAR15	12	AR06046-10-3	28.3	50.0	28.2
2015	SUS	NAR15	13	AR06061-11-1	25.0	18.3	10.4
2015	SUS	NAR15	14	LW08190C-57-3	46.7	38.3	8.9
2015	SUS	NAR15	15	ARGE08-1398	6.7	4.0	3.5
2015	SUS	NAR15	16	B12-1792	55.0	48.3	26.3
2015	SUS	NAR15	17	B12-2180NC	33.3	33.3	19.8
2015	SUS	NAR15	18	GA071171-14ES8	50.0	48.3	19.7
2015	SUS	NAR15	19	GA071092-14ES11	66.7	48.3	24.9
2015	SUS	NAR15	20	GA071092-14ES13	68.3	50.0	24.6

2015	SUS	NAR15	21	GA081129-14ES16	70.0	45.0	17.9
2015	SUS	NAR15	22	GA08250-14ES7	43.3	50.0	18.1
2015	SUS	NAR15	23	GA08250-14ES5	45.0	40.0	22.1
2015	SUS	NAR15	24	GA071171-14ES19	53.3	40.0	22.0
2015	SUS	NAR15	25	GA081562-14ES14	31.7	38.3	27.2
2015	SUS	NAR15	26	KWS054	33.3	53.3	18.8
2015	SUS	NAR15	27	LA06146E-P4	45.0	43.3	20.2
2015	SUS	NAR15	28	LA08265C-50	38.3	33.3	11.0
2015	SUS	NAR15	29	LA09144C-6	73.3	50.0	22.6
2015	SUS	NAR15	30	LANC8248-1	73.3	23.3	10.0
2015	SUS	NAR15	31	ES13-1591	46.7	38.3	11.1
2015	SUS	NAR15	32	ES13-3423	73.3	46.7	16.8
2015	SUS	NAR15	33	ES12-3030	50.0	41.7	12.2
2015	SUS	NAR15	34	M11-2024	36.7	28.3	15.8
2015	SUS	NAR15	35	M12-3301	56.7	48.3	16.0
2015	SUS	NAR15	36	M12-2036	15.0	36.7	16.8
2015	SUS	NAR15	37	NC11-23084	71.7	50.0	14.7
2015	SUS	NAR15	38	NC12-23576	63.3	46.7	20.1
2015	SUS	NAR15	39	NC12-23219	41.7	21.7	11.4
2015	SUS	NAR15	40	NC12-20662	80.0	35.0	14.7
2015	SUS	NAR15	41	NC9305-7	31.7	21.7	11.8
2015	SUS	NAR15	42	VA11W-106	28.3	46.7	28.4
2015	SUS	NAR15	43	VA11W-313	68.3	78.3	16.5
2015	SUS	NAR15	44	VA12W-72	55.0	75.0	31.4
2015	SUS	NAR15	45	VA12W-54	75.0	60.0	18.0
2015	SUS	NAR15	46	VA12FHB-53	26.7	46.7	17.1
2015	SUS	NAR15	47	VA12FHB-4	33.3	33.3	10.7
2015	SUS	NAR15	48	VA13W-177	25.0	26.7	10.9
2015	SUS	NAR15	49	VA08MAS5-39-6-4	80.0	33.3	15.3
2014	SUS	NC14	1	ERNIE	11.0	4.3	4.4
2014	SUS	NC14	2	COKER9835	78.0	24.5	35.6
2014	SUS	NC14	3	BESS	27.0	8.1	8.3
2014	SUS	NC14	4	JAMESTOWN	13.0	2.9	2.5
2014	SUS	NC14	5	M10-1615	16.5	8.2	9.5
2014	SUS	NC14	6	AR00179-2-2	19.5	3.9	6.6
2014	SUS	NC14	7	AR00334-5-2	13.5	5.6	5.4
2014	SUS	NC14	8	AR01136-3-2	32.5	7.5	7.9
2014	SUS	NC14	9	AR04001-3	23.0	3.6	2.5
2014	SUS	NC14	10	AR04084-1-3	15.0	5.7	10.2
2014	SUS	NC14	11	ARGE07-1347-6-7-9	19.0	2.0	2.8
2014	SUS	NC14	12	ARGE07-1354-2-6-1	17.0	2.0	3.2
2014	SUS	NC14	13	ARGE07-1355-16-6-6	17.5	5.9	6.7
2014	SUS	NC14	14	ARS09-228	34.0	3.3	4.9
2014	SUS	NC14	15	ARS10-028	17.5	12.6	22.3
2014	SUS	NC14	16	ARS10-038	73.0	30.8	89.1
2014	SUS	NC14	17	ARS10-043	80.0	21.2	54.9
2014	SUS	NC14	18	ARS10-172	60.0	23.8	30.6
2014	SUS	NC14	19	ARS10-389	10.0	2.6	3.4
2014	SUS	NC14	20	B09-0002	10.5	1.3	3.1

2014	SUS	NC14	21	B09-900256	63.0	17.7	17.6
2014	SUS	NC14	22	B08-91993	31.0	4.8	7.2
2014	SUS	NC14	23	GA04494-13ES1	65.0	11.4	19.4
2014	SUS	NC14	24	GA051477-13ES2	65.0	10.4	13.0
2014	SUS	NC14	25	GA051477-13ES4	35.5	6.0	5.1
2014	SUS	NC14	26	GA051207-13ES11	27.5	9.4	13.6
2014	SUS	NC14	27	GA061050-13ES18	30.0	14.8	20.8
2014	SUS	NC14	28	GA06586-13ES21	56.5	23.2	31.5
2014	SUS	NC14	29	GA06390-13ES24	30.5	14.3	17.3
2014	SUS	NC14	30	GA061050-13ES17	45.0	10.7	22.4
2014	SUS	NC14	31	KWS013	45.0	11.5	8.5
2014	SUS	NC14	32	KWS026	19.0	6.9	7.3
2014	SUS	NC14	33	KWS027	47.5	17.6	25.0
2014	SUS	NC14	34	LA06149C-P7	26.5	9.7	12.3
2014	SUS	NC14	35	LA08201C-57	18.5	7.4	11.0
2014	SUS	NC14	36	LANC8170-41-1	34.0	7.8	6.9
2014	SUS	NC14	37	LA07085CW-P4	19.0	3.2	1.9
2014	SUS	NC14	38	LANC8170-41-2	19.0	4.7	4.5
2014	SUS	NC14	39	LCS08577-4	51.5	15.2	23.3
2014	SUS	NC14	40	LCS229	50.0	10.6	21.9
2014	SUS	NC14	41	M09-9547	24.5	5.5	17.9
2014	SUS	NC14	42	M11-1027	32.0	11.0	15.0
2014	SUS	NC14	43	M11-2298	15.5	4.3	6.0
2014	SUS	NC14	44	MD08-22-22-13-4	30.0	1.5	3.5
2014	SUS	NC14	45	MD26-H2-23-13-1	10.5	1.5	3.3
2014	SUS	NC14	46	MD09W272-8-4-13-3	12.0	4.8	6.9
2014	SUS	NC14	47	MDC07026-F2-19-13-4	27.5	6.5	8.3
2014	SUS	NC14	48	NC11-21401	7.0	0.9	3.2
2014	SUS	NC14	49	NC11-22289	20.0	2.0	2.4
2014	SUS	NC14	50	NC11-22291	8.5	1.2	1.2
2014	SUS	NC14	51	NC8170-45-17	22.0	9.9	5.5
2014	SUS	NC14	52	NC8170-86-2	18.0	5.6	9.3
2014	SUS	NC14	53	NC9305-7	18.5	7.6	8.5
2014	SUS	NC14	54	NC09-21916	35.0	3.2	5.4
2014	SUS	NC14	55	VA10W-96	26.5	8.6	18.0
2014	SUS	NC14	56	VA11W-108	27.0	6.5	11.2
2014	SUS	NC14	57	VA11W-230	22.5	5.0	6.5
2014	SUS	NC14	58	VA11W-278	20.0	7.7	6.5
2014	SUS	NC14	59	VA12W-102	61.0	11.8	19.1
2014	SUS	NC14	60	VA12W-150	22.5	8.5	6.1
2014	SUS	NC14	61	VA12FHB-37	37.5	11.8	27.1
2014	SUS	NC14	62	VA12FHB-85	26.5	6.6	13.5
2014	SUS	KY14	1	ERNIE	38.6	23.6	12.3
2014	SUS	KY14	2	COKER9835	60.8	32.5	10.4
2014	SUS	KY14	3	BESS	32.8	14.1	10.0
2014	SUS	KY14	4	JAMESTOWN	31.6	26.3	17.2
2014	SUS	KY14	5	M10-1615	19.9	18.2	9.4
2014	SUS	KY14	6	AR00179-2-2	32.8	18.7	11.9
2014	SUS	KY14	7	AR00334-5-2	21.8	21.4	11.4

2014	SUS	KY14	8	AR01136-3-2	31.8	25.2	10.6
2014	SUS	KY14	9	AR04001-3	31.2	19.0	12.8
2014	SUS	KY14	10	AR04084-1-3	43.8	24.8	22.0
2014	SUS	KY14	11	ARGE07-1347-6-7-9	18.4	15.4	10.4
2014	SUS	KY14	12	ARGE07-1354-2-6-1	31.6	20.9	13.6
2014	SUS	KY14	13	ARGE07-1355-16-6-6	30.4	21.5	16.4
2014	SUS	KY14	14	ARS09-228	46.1	35.7	8.9
2014	SUS	KY14	15	ARS10-028	36.0	26.3	22.9
2014	SUS	KY14	16	ARS10-038	58.3	40.0	14.1
2014	SUS	KY14	17	ARS10-043	31.6	23.9	14.3
2014	SUS	KY14	18	ARS10-172	43.8	37.7	11.9
2014	SUS	KY14	19	ARS10-389	15.3	17.7	6.1
2014	SUS	KY14	20	B09-0002	29.9	19.7	12.3
2014	SUS	KY14	21	B09-900256	47.0	30.9	12.9
2014	SUS	KY14	22	B08-91993	47.3	22.9	12.7
2014	SUS	KY14	23	GA04494-13ES1	65.5	40.1	24.1
2014	SUS	KY14	24	GA051477-13ES2	43.2	28.8	15.8
2014	SUS	KY14	25	GA051477-13ES4	40.6	35.6	15.5
2014	SUS	KY14	26	GA051207-13ES11	31.9	23.4	15.8
2014	SUS	KY14	27	GA061050-13ES18	46.2	36.9	19.9
2014	SUS	KY14	28	GA06586-13ES21	55.4	37.9	26.1
2014	SUS	KY14	29	GA06390-13ES24	36.6	28.6	24.4
2014	SUS	KY14	30	GA061050-13ES17	51.8	34.7	18.1
2014	SUS	KY14	31	KWS013	47.0	36.8	14.6
2014	SUS	KY14	32	KWS026	22.1	12.0	7.0
2014	SUS	KY14	33	KWS027	35.4	21.0	15.1
2014	SUS	KY14	34	LA06149C-P7	42.8	31.7	23.8
2014	SUS	KY14	35	LA08201C-57	39.4	22.4	19.2
2014	SUS	KY14	36	LANC8170-41-1	43.5	25.7	11.9
2014	SUS	KY14	37	LA07085CW-P4	40.7	23.9	12.9
2014	SUS	KY14	38	LANC8170-41-2	37.1	25.2	9.3
2014	SUS	KY14	39	LCS08577-4	45.4	36.8	10.3
2014	SUS	KY14	40	LCS229	29.0	27.9	11.2
2014	SUS	KY14	41	M09-9547	35.5	23.8	17.2
2014	SUS	KY14	42	M11-1027	36.4	24.8	8.1
2014	SUS	KY14	43	M11-2298	28.3	20.5	12.2
2014	SUS	KY14	44	MD08-22-22-13-4	19.2	4.3	5.3
2014	SUS	KY14	45	MD26-H2-23-13-1	33.1	19.1	12.9
2014	SUS	KY14	46	MD09W272-8-4-13-3	34.8	23.9	12.5
2014	SUS	KY14	47	MDC07026-F2-19-13-4	32.1	20.3	9.2
2014	SUS	KY14	48	NC11-21401	23.2	22.7	13.7
2014	SUS	KY14	49	NC11-22289	21.9	14.2	9.0
2014	SUS	KY14	50	NC11-22291	22.4	17.5	9.3
2014	SUS	KY14	51	NC8170-45-17	25.5	16.6	11.6
2014	SUS	KY14	52	NC8170-86-2	47.7	23.7	10.8
2014	SUS	KY14	53	NC9305-7	34.2	24.1	15.3
2014	SUS	KY14	54	NC09-21916	33.6	17.1	9.2
2014	SUS	KY14	55	VA10W-96	31.2	19.4	11.3
2014	SUS	KY14	56	VA11W-108	46.6	29.2	16.1

2014	SUS	KY14	57	VA11W-230	39.7	27.3	7.0
2014	SUS	KY14	58	VA11W-278	55.3	33.4	14.7
2014	SUS	KY14	59	VA12W-102	39.0	25.6	6.6
2014	SUS	KY14	60	VA12W-150	42.5	23.6	10.3
2014	SUS	KY14	61	VA12FHB-37	33.9	29.9	17.1
2014	SUS	KY14	62	VA12FHB-85	22.5	19.6	13.9
2014	SUS	VA14	1	ERNIE	13.4	14.8	2.0
2014	SUS	VA14	2	COKER9835	85.9	35.9	7.3
2014	SUS	VA14	3	BESS	16.2	22.3	5.2
2014	SUS	VA14	4	JAMESTOWN	12.3	6.9	0.6
2014	SUS	VA14	5	M10-1615	11.6	5.7	1.4
2014	SUS	VA14	6	AR00179-2-2	15.4	8.4	6.9
2014	SUS	VA14	7	AR00334-5-2	14.0	5.5	0.8
2014	SUS	VA14	8	AR01136-3-2	12.1	19.7	2.0
2014	SUS	VA14	9	AR04001-3	10.3	11.0	0.6
2014	SUS	VA14	10	AR04084-1-3	19.2	11.5	2.0
2014	SUS	VA14	11	ARGE07-1347-6-7-9	11.1	27.6	12.6
2014	SUS	VA14	12	ARGE07-1354-2-6-1	12.8	23.7	3.5
2014	SUS	VA14	13	ARGE07-1355-16-6-6	19.0	8.0	1.0
2014	SUS	VA14	14	ARS09-228	8.8	12.4	0.5
2014	SUS	VA14	15	ARS10-028	22.9	14.6	2.4
2014	SUS	VA14	16	ARS10-038	72.7	26.9	12.2
2014	SUS	VA14	17	ARS10-043	17.8	13.8	2.3
2014	SUS	VA14	18	ARS10-172	57.9	23.4	5.4
2014	SUS	VA14	19	ARS10-389	8.2	13.0	0.1
2014	SUS	VA14	20	B09-0002	10.4	8.8	0.1
2014	SUS	VA14	21	B09-900256	27.6	29.2	6.6
2014	SUS	VA14	22	B08-91993	22.9	4.7	0.5
2014	SUS	VA14	23	GA04494-13ES1	26.5	23.5	2.6
2014	SUS	VA14	24	GA051477-13ES2	22.6	19.7	0.9
2014	SUS	VA14	25	GA051477-13ES4	16.1	8.2	1.1
2014	SUS	VA14	26	GA051207-13ES11	44.6	14.9	1.2
2014	SUS	VA14	27	GA061050-13ES18	21.0	18.1	4.4
2014	SUS	VA14	28	GA06586-13ES21	47.1	12.6	3.8
2014	SUS	VA14	29	GA06390-13ES24	19.1	32.8	2.0
2014	SUS	VA14	30	GA061050-13ES17	19.6	18.9	1.8
2014	SUS	VA14	31	KWS013	8.8	10.6	0.9
2014	SUS	VA14	32	KWS026	20.3	14.0	0.8
2014	SUS	VA14	33	KWS027	16.4	12.4	1.6
2014	SUS	VA14	34	LA06149C-P7	17.8	18.5	0.8
2014	SUS	VA14	35	LA08201C-57	34.7	12.3	0.9
2014	SUS	VA14	36	LANC8170-41-1	54.3	10.9	2.1
2014	SUS	VA14	37	LA07085CW-P4	21.0	20.8	1.1
2014	SUS	VA14	38	LANC8170-41-2	9.0	14.2	0.9
2014	SUS	VA14	39	LCS08577-4	37.0	21.3	2.2
2014	SUS	VA14	40	LCS229	26.4	14.0	1.3
2014	SUS	VA14	41	M09-9547	19.5	17.2	2.2
2014	SUS	VA14	42	M11-1027	19.8	25.6	2.0
2014	SUS	VA14	43	M11-2298	13.6	16.0	1.8

2014	SUS	VA14	44	MD08-22-22-13-4	17.4	11.5	2.2
2014	SUS	VA14	45	MD26-H2-23-13-1	7.9	6.3	0.1
2014	SUS	VA14	46	MD09W272-8-4-13-3	22.7	16.4	1.6
2014	SUS	VA14	47	MDC07026-F2-19-13-4	10.9	19.7	1.0
2014	SUS	VA14	48	NC11-21401	9.9	20.0	1.5
2014	SUS	VA14	49	NC11-22289	7.5	18.6	2.6
2014	SUS	VA14	50	NC11-22291	18.3	10.3	NA
2014	SUS	VA14	51	NC8170-45-17	11.3	18.2	2.6
2014	SUS	VA14	52	NC8170-86-2	14.7	10.4	0.4
2014	SUS	VA14	53	NC9305-7	26.2	14.2	1.2
2014	SUS	VA14	54	NC09-21916	13.9	11.1	0.3
2014	SUS	VA14	55	VA10W-96	22.5	14.4	0.4
2014	SUS	VA14	56	VA11W-108	39.6	25.6	4.6
2014	SUS	VA14	57	VA11W-230	12.1	9.1	0.8
2014	SUS	VA14	58	VA11W-278	44.3	17.9	2.8
2014	SUS	VA14	59	VA12W-102	16.7	21.0	3.7
2014	SUS	VA14	60	VA12W-150	46.2	13.6	4.3
2014	SUS	VA14	61	VA12FHB-37	23.5	29.1	2.1
2014	SUS	VA14	62	VA12FHB-85	25.8	27.5	2.1
2014	SUS	FAR14	1	ERNIE	7.5	14.0	9.0
2014	SUS	FAR14	2	COKER9835	50.0	45.0	11.9
2014	SUS	FAR14	3	BESS	5.0	12.0	6.9
2014	SUS	FAR14	4	JAMESTOWN	5.0	26.5	3.0
2014	SUS	FAR14	5	M10-1615	12.5	29.0	5.9
2014	SUS	FAR14	6	AR00179-2-2	7.5	8.0	1.8
2014	SUS	FAR14	7	AR00334-5-2	7.5	25.0	6.9
2014	SUS	FAR14	8	AR01136-3-2	10.0	57.5	3.2
2014	SUS	FAR14	9	AR04001-3	15.0	30.0	6.5
2014	SUS	FAR14	10	AR04084-1-3	22.5	24.0	7.2
2014	SUS	FAR14	11	ARGE07-1347-6-7-9	5.0	23.5	4.8
2014	SUS	FAR14	12	ARGE07-1354-2-6-1	5.0	15.0	4.2
2014	SUS	FAR14	13	ARGE07-1355-16-6-6	10.0	15.0	6.4
2014	SUS	FAR14	14	ARS09-228	20.0	32.5	7.1
2014	SUS	FAR14	15	ARS10-028	10.0	27.5	6.4
2014	SUS	FAR14	16	ARS10-038	32.5	90.0	18.3
2014	SUS	FAR14	17	ARS10-043	30.0	60.0	8.6
2014	SUS	FAR14	18	ARS10-172	35.0	55.0	9.1
2014	SUS	FAR14	19	ARS10-389	7.5	19.0	4.4
2014	SUS	FAR14	20	B09-0002	7.5	6.0	2.4
2014	SUS	FAR14	21	B09-900256	30.0	75.0	10.8
2014	SUS	FAR14	22	B08-91993	30.0	47.5	11.4
2014	SUS	FAR14	23	GA04494-13ES1	40.0	67.5	13.2
2014	SUS	FAR14	24	GA051477-13ES2	32.5	32.5	7.7
2014	SUS	FAR14	25	GA051477-13ES4	30.0	40.0	6.3
2014	SUS	FAR14	26	GA051207-13ES11	25.0	50.0	7.1
2014	SUS	FAR14	27	GA061050-13ES18	70.0	52.5	14.9
2014	SUS	FAR14	28	GA06586-13ES21	57.5	70.0	23.9
2014	SUS	FAR14	29	GA06390-13ES24	25.0	67.5	9.5
2014	SUS	FAR14	30	GA061050-13ES17	37.5	30.0	12.0

2014	SUS	FAR14	31	KWS013	NA	45.0	7.1
2014	SUS	FAR14	32	KWS026	22.5	12.5	2.6
2014	SUS	FAR14	33	KWS027	25.0	70.0	17.6
2014	SUS	FAR14	34	LA06149C-P7	10.0	35.0	4.3
2014	SUS	FAR14	35	LA08201C-57	22.5	31.0	8.4
2014	SUS	FAR14	36	LANC8170-41-1	37.5	37.5	5.2
2014	SUS	FAR14	37	LA07085CW-P4	12.5	37.5	6.2
2014	SUS	FAR14	38	LANC8170-41-2	30.0	17.5	3.0
2014	SUS	FAR14	39	LCS08577-4	65.0	34.0	7.6
2014	SUS	FAR14	40	LCS229	32.5	36.0	8.6
2014	SUS	FAR14	41	M09-9547	22.5	35.0	7.6
2014	SUS	FAR14	42	M11-1027	20.0	31.5	6.6
2014	SUS	FAR14	43	M11-2298	45.0	30.0	7.4
2014	SUS	FAR14	44	MD08-22-22-13-4	2.5	13.5	1.5
2014	SUS	FAR14	45	MD26-H2-23-13-1	7.5	31.5	5.3
2014	SUS	FAR14	46	MD09W272-8-4-13-3	17.5	35.0	4.2
2014	SUS	FAR14	47	MDC07026-F2-19-13-4	22.5	50.0	7.2
2014	SUS	FAR14	48	NC11-21401	25.0	52.5	3.1
2014	SUS	FAR14	49	NC11-22289	15.0	32.5	2.9
2014	SUS	FAR14	50	NC11-22291	12.5	30.0	3.5
2014	SUS	FAR14	51	NC8170-45-17	5.0	25.0	3.3
2014	SUS	FAR14	52	NC8170-86-2	17.5	65.0	5.5
2014	SUS	FAR14	53	NC9305-7	5.0	27.5	6.9
2014	SUS	FAR14	54	NC09-21916	20.0	27.5	3.2
2014	SUS	FAR14	55	VA10W-96	20.0	50.0	5.1
2014	SUS	FAR14	56	VA11W-108	7.5	62.5	6.8
2014	SUS	FAR14	57	VA11W-230	10.0	57.5	4.5
2014	SUS	FAR14	58	VA11W-278	32.5	60.0	7.9
2014	SUS	FAR14	59	VA12W-102	27.5	45.0	7.6
2014	SUS	FAR14	60	VA12W-150	32.5	47.5	8.3
2014	SUS	FAR14	61	VA12FHB-37	20.0	77.5	8.6
2014	SUS	FAR14	62	VA12FHB-85	30.0	70.0	10.8
2014	SUS	FUN14	1	ERNIE	33.2	48.0	NA
2014	SUS	FUN14	2	COKER9835	43.5	83.7	NA
2014	SUS	FUN14	3	BESS	27.2	31.4	NA
2014	SUS	FUN14	4	JAMESTOWN	19.6	23.5	NA
2014	SUS	FUN14	5	M10-1615	15.7	17.5	NA
2014	SUS	FUN14	6	AR00179-2-2	18.5	18.7	NA
2014	SUS	FUN14	7	AR00334-5-2	24.0	27.1	NA
2014	SUS	FUN14	8	AR01136-3-2	19.2	21.8	NA
2014	SUS	FUN14	9	AR04001-3	35.2	64.6	NA
2014	SUS	FUN14	10	AR04084-1-3	27.4	55.2	NA
2014	SUS	FUN14	11	ARGE07-1347-6-7-9	16.3	13.7	NA
2014	SUS	FUN14	12	ARGE07-1354-2-6-1	22.3	29.1	NA
2014	SUS	FUN14	13	ARGE07-1355-16-6-6	34.5	42.1	NA
2014	SUS	FUN14	14	ARS09-228	28.5	51.4	NA
2014	SUS	FUN14	15	ARS10-028	11.3	19.4	NA
2014	SUS	FUN14	16	ARS10-038	64.4	43.5	NA
2014	SUS	FUN14	17	ARS10-043	25.2	31.2	NA

2014	SUS	FUN14	18	ARS10-172	42.6	50.1	NA
2014	SUS	FUN14	19	ARS10-389	25.9	48.5	NA
2014	SUS	FUN14	20	B09-0002	17.9	30.6	NA
2014	SUS	FUN14	21	B09-900256	22.6	42.6	NA
2014	SUS	FUN14	22	B08-91993	39.7	55.1	NA
2014	SUS	FUN14	23	GA04494-13ES1	55.7	74.6	NA
2014	SUS	FUN14	24	GA051477-13ES2	23.6	50.9	NA
2014	SUS	FUN14	25	GA051477-13ES4	26.0	51.3	NA
2014	SUS	FUN14	26	GA051207-13ES11	46.8	54.5	NA
2014	SUS	FUN14	27	GA061050-13ES18	35.7	58.2	NA
2014	SUS	FUN14	28	GA06586-13ES21	60.6	59.0	NA
2014	SUS	FUN14	29	GA06390-13ES24	26.6	56.0	NA
2014	SUS	FUN14	30	GA061050-13ES17	25.8	40.5	NA
2014	SUS	FUN14	31	KWS013	NA	NA	NA
2014	SUS	FUN14	32	KWS026	NA	NA	NA
2014	SUS	FUN14	33	KWS027	NA	NA	NA
2014	SUS	FUN14	34	LA06149C-P7	45.1	40.0	NA
2014	SUS	FUN14	35	LA08201C-57	NA	NA	NA
2014	SUS	FUN14	36	LANC8170-41-1	53.4	62.0	NA
2014	SUS	FUN14	37	LA07085CW-P4	18.2	49.0	NA
2014	SUS	FUN14	38	LANC8170-41-2	50.0	43.0	NA
2014	SUS	FUN14	39	LCS08577-4	NA	NA	NA
2014	SUS	FUN14	40	LCS229	NA	NA	NA
2014	SUS	FUN14	41	M09-9547	35.5	41.7	NA
2014	SUS	FUN14	42	M11-1027	23.2	37.7	NA
2014	SUS	FUN14	43	M11-2298	22.7	38.5	NA
2014	SUS	FUN14	44	MD08-22-22-13-4	13.4	27.2	NA
2014	SUS	FUN14	45	MD26-H2-23-13-1	14.2	20.3	NA
2014	SUS	FUN14	46	MD09W272-8-4-13-3	13.1	24.3	NA
2014	SUS	FUN14	47	MDC07026-F2-19-13-4	20.2	36.2	NA
2014	SUS	FUN14	48	NC11-21401	11.6	34.5	NA
2014	SUS	FUN14	49	NC11-22289	17.0	28.4	NA
2014	SUS	FUN14	50	NC11-22291	16.2	22.3	NA
2014	SUS	FUN14	51	NC8170-45-17	16.0	40.9	NA
2014	SUS	FUN14	52	NC8170-86-2	29.0	36.2	NA
2014	SUS	FUN14	53	NC9305-7	42.9	58.8	NA
2014	SUS	FUN14	54	NC09-21916	41.0	63.7	NA
2014	SUS	FUN14	55	VA10W-96	27.7	45.1	NA
2014	SUS	FUN14	56	VA11W-108	27.4	28.4	NA
2014	SUS	FUN14	57	VA11W-230	32.0	49.2	NA
2014	SUS	FUN14	58	VA11W-278	37.8	62.4	NA
2014	SUS	FUN14	59	VA12W-102	26.3	39.8	NA
2014	SUS	FUN14	60	VA12W-150	35.3	38.2	NA
2014	SUS	FUN14	61	VA12FHB-37	14.2	24.2	NA
2014	SUS	FUN14	62	VA12FHB-85	33.8	44.5	NA
2014	SUS	LA14	1	ERNIE	20.0	12.5	NA
2014	SUS	LA14	2	COKER9835	50.0	47.5	NA
2014	SUS	LA14	3	BESS	20.0	7.5	NA
2014	SUS	LA14	4	JAMESTOWN	15.0	5.0	NA

2014	SUS	LA14	5	M10-1615	30.0	42.5	NA
2014	SUS	LA14	6	AR00179-2-2	25.0	22.5	NA
2014	SUS	LA14	7	AR00334-5-2	40.0	30.0	NA
2014	SUS	LA14	8	AR01136-3-2	30.0	20.0	NA
2014	SUS	LA14	9	AR04001-3	25.0	5.0	NA
2014	SUS	LA14	10	AR04084-1-3	30.0	10.0	NA
2014	SUS	LA14	11	ARGE07-1347-6-7-9	15.0	5.0	NA
2014	SUS	LA14	12	ARGE07-1354-2-6-1	20.0	5.0	NA
2014	SUS	LA14	13	ARGE07-1355-16-6-6	20.0	5.0	NA
2014	SUS	LA14	14	ARS09-228	25.0	15.0	NA
2014	SUS	LA14	15	ARS10-028	25.0	25.0	NA
2014	SUS	LA14	16	ARS10-038	55.0	60.0	NA
2014	SUS	LA14	17	ARS10-043	50.0	55.0	NA
2014	SUS	LA14	18	ARS10-172	45.0	30.0	NA
2014	SUS	LA14	19	ARS10-389	15.0	5.0	NA
2014	SUS	LA14	20	B09-0002	10.0	5.0	NA
2014	SUS	LA14	21	B09-900256	45.0	42.5	NA
2014	SUS	LA14	22	B08-91993	30.0	40.0	NA
2014	SUS	LA14	23	GA04494-13ES1	25.0	30.0	NA
2014	SUS	LA14	24	GA051477-13ES2	25.0	7.5	NA
2014	SUS	LA14	25	GA051477-13ES4	15.0	5.0	NA
2014	SUS	LA14	26	GA051207-13ES11	30.0	10.0	NA
2014	SUS	LA14	27	GA061050-13ES18	20.0	10.0	NA
2014	SUS	LA14	28	GA06586-13ES21	45.0	55.0	NA
2014	SUS	LA14	29	GA06390-13ES24	30.0	20.0	NA
2014	SUS	LA14	30	GA061050-13ES17	20.0	10.0	NA
2014	SUS	LA14	31	KWS013	40.0	32.5	NA
2014	SUS	LA14	32	KWS026	35.0	10.0	NA
2014	SUS	LA14	33	KWS027	35.0	32.5	NA
2014	SUS	LA14	34	LA06149C-P7	35.0	7.5	NA
2014	SUS	LA14	35	LA08201C-57	30.0	7.5	NA
2014	SUS	LA14	36	LANC8170-41-1	35.0	22.5	NA
2014	SUS	LA14	37	LA07085CW-P4	20.0	5.0	NA
2014	SUS	LA14	38	LANC8170-41-2	20.0	7.5	NA
2014	SUS	LA14	39	LCS08577-4	50.0	55.0	NA
2014	SUS	LA14	40	LCS229	50.0	37.5	NA
2014	SUS	LA14	41	M09-9547	30.0	47.5	NA
2014	SUS	LA14	42	M11-1027	30.0	12.5	NA
2014	SUS	LA14	43	M11-2298	10.0	5.0	NA
2014	SUS	LA14	44	MD08-22-22-13-4	25.0	5.0	NA
2014	SUS	LA14	45	MD26-H2-23-13-1	35.0	20.0	NA
2014	SUS	LA14	46	MD09W272-8-4-13-3	25.0	7.5	NA
2014	SUS	LA14	47	MDC07026-F2-19-13-4	25.0	10.0	NA
2014	SUS	LA14	48	NC11-21401	25.0	7.5	NA
2014	SUS	LA14	49	NC11-22289	12.5	5.0	NA
2014	SUS	LA14	50	NC11-22291	15.0	5.0	NA
2014	SUS	LA14	51	NC8170-45-17	15.0	5.0	NA
2014	SUS	LA14	52	NC8170-86-2	15.0	7.5	NA
2014	SUS	LA14	53	NC9305-7	25.0	10.0	NA

2014	SUS	LA14	54	NC09-21916	35.0	10.0	NA
2014	SUS	LA14	55	VA10W-96	40.0	37.5	NA
2014	SUS	LA14	56	VA11W-108	45.0	42.5	NA
2014	SUS	LA14	57	VA11W-230	30.0	10.0	NA
2014	SUS	LA14	58	VA11W-278	45.0	25.0	NA
2014	SUS	LA14	59	VA12W-102	40.0	37.5	NA
2014	SUS	LA14	60	VA12W-150	45.0	15.0	NA
2014	SUS	LA14	61	VA12FHB-37	40.0	32.5	NA
2014	SUS	LA14	62	VA12FHB-85	35.0	17.5	NA
2014	SUS	NAR14	1	ERNIE	33.3	35.0	12.0
2014	SUS	NAR14	2	COKER9835	80.0	91.0	36.9
2014	SUS	NAR14	3	BESS	21.7	28.3	16.8
2014	SUS	NAR14	4	JAMESTOWN	41.7	32.7	16.2
2014	SUS	NAR14	5	M10-1615	25.0	30.0	19.1
2014	SUS	NAR14	6	AR00179-2-2	31.7	32.7	17.0
2014	SUS	NAR14	7	AR00334-5-2	30.0	37.3	18.6
2014	SUS	NAR14	8	AR01136-3-2	36.7	54.0	14.1
2014	SUS	NAR14	9	AR04001-3	43.3	45.0	13.6
2014	SUS	NAR14	10	AR04084-1-3	61.7	60.0	19.4
2014	SUS	NAR14	11	ARGE07-1347-6-7-9	25.0	43.3	10.5
2014	SUS	NAR14	12	ARGE07-1354-2-6-1	38.3	48.3	20.2
2014	SUS	NAR14	13	ARGE07-1355-16-6-6	31.7	29.3	15.9
2014	SUS	NAR14	14	ARS09-228	61.7	76.7	29.6
2014	SUS	NAR14	15	ARS10-028	56.7	85.0	36.0
2014	SUS	NAR14	16	ARS10-038	85.0	96.7	32.2
2014	SUS	NAR14	17	ARS10-043	68.3	68.3	25.5
2014	SUS	NAR14	18	ARS10-172	71.7	81.7	26.8
2014	SUS	NAR14	19	ARS10-389	33.3	33.3	11.2
2014	SUS	NAR14	20	B09-0002	23.3	23.3	11.3
2014	SUS	NAR14	21	B09-900256	66.7	83.3	24.2
2014	SUS	NAR14	22	B08-91993	45.0	50.0	17.0
2014	SUS	NAR14	23	GA04494-13ES1	76.7	88.3	23.5
2014	SUS	NAR14	24	GA051477-13ES2	75.0	58.3	21.7
2014	SUS	NAR14	25	GA051477-13ES4	68.3	51.7	21.2
2014	SUS	NAR14	26	GA051207-13ES11	46.7	60.0	22.4
2014	SUS	NAR14	27	GA061050-13ES18	61.7	68.3	30.4
2014	SUS	NAR14	28	GA06586-13ES21	70.0	76.7	34.3
2014	SUS	NAR14	29	GA06390-13ES24	68.3	81.7	29.8
2014	SUS	NAR14	30	GA061050-13ES17	70.0	81.7	33.0
2014	SUS	NAR14	31	KWS013	56.7	56.7	14.9
2014	SUS	NAR14	32	KWS026	38.3	23.3	9.9
2014	SUS	NAR14	33	KWS027	48.3	80.0	37.9
2014	SUS	NAR14	34	LA06149C-P7	56.7	61.7	29.2
2014	SUS	NAR14	35	LA08201C-57	58.3	46.7	9.8
2014	SUS	NAR14	36	LANC8170-41-1	65.0	58.3	8.2
2014	SUS	NAR14	37	LA07085CW-P4	53.3	66.7	13.4
2014	SUS	NAR14	38	LANC8170-41-2	55.0	48.3	6.1
2014	SUS	NAR14	39	LCS08577-4	66.7	61.7	13.8
2014	SUS	NAR14	40	LCS229	60.0	61.7	20.6

2014	SUS	NAR14	41	M09-9547	58.3	53.3	24.0
2014	SUS	NAR14	42	M11-1027	46.7	53.3	19.3
2014	SUS	NAR14	43	M11-2298	38.3	40.0	15.6
2014	SUS	NAR14	44	MD08-22-22-13-4	33.3	30.0	6.6
2014	SUS	NAR14	45	MD26-H2-23-13-1	26.7	38.3	12.8
2014	SUS	NAR14	46	MD09W272-8-4-13-3	48.3	71.7	16.5
2014	SUS	NAR14	47	MDC07026-F2-19-13-4	43.3	66.7	10.3
2014	SUS	NAR14	48	NC11-21401	38.3	38.3	9.5
2014	SUS	NAR14	49	NC11-22289	33.3	30.0	9.8
2014	SUS	NAR14	50	NC11-22291	28.3	26.0	9.7
2014	SUS	NAR14	51	NC8170-45-17	40.0	40.0	9.1
2014	SUS	NAR14	52	NC8170-86-2	55.0	45.0	12.5
2014	SUS	NAR14	53	NC9305-7	36.7	45.0	15.5
2014	SUS	NAR14	54	NC09-21916	46.7	56.7	18.5
2014	SUS	NAR14	55	VA10W-96	70.0	43.3	13.7
2014	SUS	NAR14	56	VA11W-108	58.3	70.0	17.5
2014	SUS	NAR14	57	VA11W-230	60.0	60.0	17.0
2014	SUS	NAR14	58	VA11W-278	63.3	71.7	15.8
2014	SUS	NAR14	59	VA12W-102	46.7	58.3	21.2
2014	SUS	NAR14	60	VA12W-150	56.7	58.3	26.3
2014	SUS	NAR14	61	VA12FHB-37	56.7	78.3	36.6
2014	SUS	NAR14	62	VA12FHB-85	48.3	65.0	26.9
